# Cassava pullulanase and its synergistic debranching action with isoamylase 3 in starch catabolism

**DOI:** 10.3389/fpls.2023.1114215

**Published:** 2023-01-27

**Authors:** Karan Wangpaiboon, Thanapon Charoenwongpaiboon, Methus Klaewkla, Robert A. Field, Pawinee Panpetch

**Affiliations:** ^1^ Center of Excellence in Structural and Computational Biology, Department of Biochemistry, Faculty of Science, Chulalongkorn University, Bangkok, Thailand; ^2^ Department of Chemistry, Faculty of Science, Silpakorn University, Nakhon Pathom, Thailand; ^3^ Department of Chemistry and Manchester Institute of Biotechnology, The University of Manchester, Manchester, United Kingdom; ^4^ Center of Excellence in Molecular Crop, Department of Biochemistry, Faculty of Science, Chulalongkorn University, Bangkok, Thailand

**Keywords:** cassava (*Manihot esculenta* Crantz), debranching enzyme, pullulan, pullulanase, starch, starch degradation, synergistic debranching

## Abstract

Pullulanase (EC 3.2.1.41, PUL), a debranching enzyme belonging to glycoside hydrolase family 13 subfamily 13, catalyses the cleavage of α-1,6 linkages of pullulan and β-limit dextrin. The present work studied PUL from cassava *Manihot esculenta* Crantz (*Me*PUL) tubers, an important economic crop. The *Mepul* gene was successfully cloned and expressed in *E. coli* and r*Me*PUL was biochemically characterised. *Me*PUL was present as monomer and homodimer, as judged by apparent mass of ~ 84 - 197 kDa by gel permeation chromatography analysis. Optimal pH and temperature were at pH 6.0 and 50 °C, and enzyme activity was enhanced by the addition of Ca^2+^ ions. Pullulan is the most favourable substrate for r*Me*PUL, followed by β-limit dextrin. Additionally, maltooligosaccharides were potential allosteric modulators of r*Me*PUL. Interestingly, short-chain maltooligosaccharides (DP 2 - 4) were significantly revealed at a higher level when r*Me*PUL was mixed with cassava isoamylase 3 (r*Me*ISA3), compared to that of each single enzyme reaction. This suggests that *Me*PUL and *Me*ISA3 debranch β-limit dextrin in a synergistic manner, which represents a major starch catabolising process in dicots. Additionally, subcellular localisation suggested the involvement of *Me*PUL in starch catabolism, which normally takes place in plastids.

## 1 Introduction

Starch is a vital carbohydrate reserve in plants and serves as a key component of the food chain for animals. It is synthesised in plastids, including chloroplasts in leaves and amyloplasts in tubers and grain. Starch consists of two α-glucans: amylose and amylopectin. The amylose mainly presents as a linear glucan structure comprised of α-1,4 glycosidic bond with a limited α-1,6-linked branching (less than 1%). This glucan is synthesised by the concerted action of ADP-glucose pyrophosphorylase (AGPase) and granule-bound starch synthase I (GBSSI). In contrast, amylopectin is much more complex since it harbours highly branched structures generated by the action of AGPase, soluble starch synthases (SSs), starch branching enzymes (SBEs), and starch debranching enzymes (DBEs) (Ball and Morell, 2003; Tappiban et al., 2019). In addition, the starch degrading process in chloroplasts also relies on several enzymes, including β-amylases (BAMs), disproportionation enzyme 1 (D-enzyme1 or DPE1) and debranching enzymes (DBEs), which generate residual sugars, glucose and maltose, which are exported to the cytosol to be further metabolised (Zeeman et al., 2010).

Debranching enzymes (DBEs), members of glycoside hydrolase family 13 (GH13), are found in plants and microorganisms. They could be categorised into two major types: isoamylase (EC 3.2.1.68, ISA) and pullulanase (PUL, limit dextrinase, R-enzyme, EC 3.2.1.41) according to their peptide sequences and substrate specificities. Although they both can act on α-1,6 glucosidic linkages (Panpetch et al., 2018b), only PUL shows high specific activity against pullulan, α-1,6-linked maltotriose units, and liberates maltotriose final product. DBEs are required for both starch synthesis and degradation in plants. Generally, plants contain 3 isoforms of ISA (ISA1, ISA2, and ISA3) but only 1 pullulanase (Zeeman et al., 2010). The ISA isoforms 1 and 2 (ISA1 and ISA2) exist as either heterooligo- or homooligo-meric structures, depending on the plant species (Hussain et al., 2003; Panpetch et al., 2018b). They have been proposed to play important roles mainly in starch synthesis by the removal of redundant branches of immature amylopectin. This produces an appropriate structure that is competent to form highly structured granular starch, based on “the glucan trimming model” (Myers et al., 2000; Nakamura, 2002). Loss of DBEs in starch biosynthesis results in the accumulation of phytoglycogen (Delatte et al., 2005), while in starch degradation, a starch-excess or *sex* phenotype arises (Caspar et al., 1991). Further, PUL together with ISA3 participates in the debranching of so-called β-limit dextrin, starch residues arising from the action of BAM (Zeeman et al., 2010). Deficiency of these debranching enzymes promotes the accumulation of branch-maltooliogosaccharides and sex phenotypes, resulting in a slow growth rate (Delatte et al., 2006). To date, plant starch metabolism has been mainly evaluated in model plants such as *Arabidopsis thaliana*; additional information on starch metabolism in other plants, however, is also required.

Currently, only plant PUL from barley (*Hordeum vulgare*) (*Hv*LD), a monocotyledon, has been biochemically and structurally characterised (Møller et al., 2015). The *Hv*LD was classified into GH13 subfamily 13. In addition to the 3 major domains, including N-, C-, and catalytic domains, *Hv*LD structure also haboured an additional carbohydrate-binding module 48 (CBM48) located between N-terminal domain and catalytic domain (Stam et al., 2006; Machovič and Janeček, 2008; Vester-Christensen et al., 2010b; Drula et al., 2022). Here is less data for PUL, especially from dicotyledonous plants. Only partially purified PUL from potato (*Solarium tuberosum* L.) (*St*PUL) (Ishizaki et al., 1983) and purified PUL from spinach (*Spinacia oleracea L.*) (*So*PUL) (Renz et al., 1998) were biochemically characterised to date. Moreover, the PUL from cassava, a tropical dicotyledonous plant, has not been studied before (Tappiban et al., 2019).

Cassava (*Manihot esculenta* Crantz), one of the most important economic crops in Asia, Africa and South America, in particular, possesses a very high starch content in its tubers (Tappiban et al., 2019). Although cassava starch has been widely used for many years, its starch-modifying enzymes have been little studied. Some of these enzymes have been cloned, expressed, and characterised (Tappiban et al., 2019), such as DPE1 (Tantanarat et al., 2014), ISA1/ISA2 (Panpetch et al., 2018b), and ISA3 (Panpetch et al., 2018a). Notably, most studies of plant pullulanases have been conducted *in vivo* (Dinges et al., 2003; Fujita et al., 2009; Li et al., 2019), which is complicated by the concerted action of several other enzymes and, as a result, specific action information about individual enzymes has rarely been investigated. This study aimed to biochemically characterise pullulanase from *Manihot esculenta* Crantz (*Me*PUL), with a view to determining its biological role in starch metabolism which remained unclear.

## 2 Materials and methods

### 2.1 *Mepul* gene cloning

Total RNA was extracted from nine-month-old cassava *Manihot esculenta* Crantz cultivar ‘KU50’ tubers harvested from the National Research Centre of Millet and Corn, Thailand, using PureLink™ Plant RNA Reagent (Invitrogen™). The RNA was converted into cDNA using a SMARTer^®^ RACE 5′/3′ Kit (Clontech^®^) followed the manufacturer’s manual. Full length *MePUL* was amplified by PrimeSTAR^®^ HS DNA Polymerase (Clontech^®^) using the 5′ RACE cDNA as a template. 5′ RACE forward primer provided in the RACE kit and a gene specific reverse primer (5′ GTAAGCTTAAATTTTCCTAGGCTCAACAAACACAG 3′) were used for PCR. The PCR product was cloned into a pJET1.2/blunt (Thermo Fisher Scientific™) and the putative gene was confirmed by DNA sequencing (1^st^ BASE DNA sequencing). A new forward primer (5′ CTCTA GCTAGCGGTTCCACTCCCACTTCTGAGTTGC 3′) excluding a transit peptide sequence predicted by ChloroP1.1 server and a new reverse primer (5′ AAGGAAAAAATGCGGCCGCAATTTTCCTAGGCTCAACAAACAC 3′) were specifically designed. The gene excluding a stop codon was subcloned into a pET21b vector *via Nhe*I and *Not*I restriction sites in-framed with C-terminal 6x His sequence of the plasmid. The recombinant plasmid (pET21b-*Mepul*) was transformed into *E. coli* TOP10 (Invitrogen™). The plasmid was extracted and subjected to DNA sequencing.

### 2.2 *Mepul* gene expression and purification of the recombinant *Me*PUL (r*Me*PUL)

The pET21b-*Mepul* was transformed into *E. coli* SoluBL21 (DE3) and grown on an LB agar plate containing 100 μg/mL ampicillin. Then, the transformant was cultured in LB broth containing 100 μg/mL ampicillin until OD_600_ reached ~ 0.6. Cells were induced for protein production by adding 0.4 mM IPTG and continuously cultured at 16°C with shaking at 250 rpm for overnight. Cells were collected by centrifugation at 8,000 x g for 10 min at 4°C. After that, the cell pellet was suspended in buffer A (25 mM phosphate buffer + 500 mM NaCl + 25 mM imidazole, pH 7.2) and lysed by ultrasonication. After that, cell debris was removed by centrifugation at 10,000 x g for 10 min at 4°C. The supernatant was loaded onto a Histrap™ FF column equilibrated with the buffer A. The column was washed by the same buffer and the r*Me*PUL was eluted with a linear gradient of buffer B (25 mM phosphate buffer + 500 mM NaCl + 50 – 300 mM imidazole, pH 7.2). Finally, the purified fractions containing *Me*PUL activity were pooled and subjected to dialysis against 25 mM phosphate buffer pH 7.2 at 4°C for overnight. SDS-PAGE and western blot analysis were used for evaluating protein purity.

### 2.3 r*Me*PUL oligomer state analysis

The molecular size of purified r*Me*PUL was analysed with an MAbPac™ SEC-1 column (4 x 300 mm, Thermo Fisher Scientific™). The system was run in 25 mM phosphate buffer pH 6.8 containing 300 mM NaCl with a flowrate of 0.2 mL/min at 30°C. The signal was monitored by measuring A_280_. Gel Filtration Standards (Bio-Rad) were used for molecular weight calibration. To investigate the multimeric state of r*Me*PUL/r*Me*ISA3 (Panpetch et al., 2018a), ~ 0.26 nmol of each enzyme was mixed and incubated in 25 mM phosphate buffer pH 7.2 in 0.1 mL total volume at 4°C overnight prior to analysis.

### 2.4 r*Me*PUL activity assay

The purified r*Me*PUL was incubated with 0.75% (w/v) pullulan (Megazyme^®^) in 25 mM acetate buffer pH 6.0 in the total volume of 0.2 mL at 50°C to determine debranching activity. The reaction was terminated by adding an equal volume of 2,4-dinitrosalicylic (DNS) acid reagent (Miller, 1959) and then boiled for 10 min. The colour development of the reactions was monitored by measuring A_540_. One unit of *Me*PUL was defined as the amount of enzyme that release 1 μmol of reducing sugar from pullulan substrate in a minute. Standard glucose in range of 0, 0.5, 1, 1.5, 2, 4, 6, and 8 mM was used for a standard curve.

### 2.5 Biochemical characterisations of r*Me*PUL

The substrate specificity of r*Me*PUL and the effect of pH, temperature, and metal ions and chemicals, and enzyme kinetics were studied. The characterisation was monitored by relative debranching activity of the purified enzyme determined by modified DNS method as described in the enzymatic assay. All experiments were performed in triplicate.

#### 2.5.1 Optimal pH

The purified r*Me*PUL was assayed in the reaction containing 0.75% (w/v) pullulan in 25 mM buffer at various pH values [acetate buffer (pH 4.0 – 6.0) and phosphate buffer (pH 6.0 – 7.5)] at 30°C.

#### 2.5.2 Optimal temperature

The purified r*Me*PUL was assayed in reactions containing 0.75% (w/v) pullulan in 25 mM acetate buffer pH 6.0 incubated at various temperatures (30 - 60°C).

#### 2.5.3 Effect of metal ions

To evaluate the effect of EDTA, the activity of purified r*Me*PUL was measured in the reaction containing 0.75% (w/v) pullulan in 25 mM acetate buffer pH 6.0 and 1 mM EDTA at 50°C. The activity was compared with the reaction without EDTA.

The effect of various metal ions was observed. The purified r*Me*PUL was dialysed against 1 mM EDTA in 25 mM phosphate buffer pH 7.2 at 4°C overnight. Next, the pretreated enzyme was incubated with 0.75% (w/v) pullulan substrate in 25 mM acetate buffer pH 6.0 at 50°C with the presence of 10 mM metal ion [CaCl_2_, CoCl_2_, CuSO_4_, FeCl_2_, MnCl_2_, MgCl_2_, NiSO_4_, and ZnCl_2_].

#### 2.5.4 Substrate specificity

The purified r*Me*PUL was incubated with 0.75% (w/v) of different types of substrates [pullulan, β-limit dextrin, amylopectin, potato starch, cassava starch, glycogen type II, and maltodextrin] in 25 mM acetate buffer pH 6.0 at 50°C.

#### 2.5.5 Kinetic studies

To assess enzyme kinetics, the purified r*Me*PUL was incubated with pullulan or β-limit dextrin in 25 mM acetate buffer pH 6.0 at 50°C in 0.1-mL reactions under enzyme initial velocity of 15 min. The reactions were initiated by addition of 0.75 μg of the purified r*Me*PUL and then analysed by DNS method. To determine the effect of some maltooligosaccharides on enzyme activity, 1 - 3 mM maltose or maltotriose were mixed in the reactions. The kinetic parameters were fitted and determined by the OriginPro 2017 software.

#### 2.5.6 Effect of β-cyclodextrin on *Me*PUL activity

The r*Me*PUL was incubated with 0.75% (w/v) pullulan and 0.01% (w/v) β-CD in 25 mM acetate buffer pH 6.0 in the total volume of 0.2 mL at 50°C. The activity was assayed by DNS method. Relative activity was compared with the control reaction without adding β-CD.

### 2.6 Product analysis

#### 2.6.1 Thin-layer chromatography (TLC)

To determine the substrate specificity of *Me*PUL, purified r*Me*PUL was incubated with 2.5 mg/mL various oligosaccharides [maltohexaose (G6, Wako^®^), maltotriosyl maltotriose (G3G3, Megazyme^®^), glucosyl maltotriose (G1G3), panose (Megazyme^®^), isopanose (Megazyme^®^), isomaltotriose (IMO3, TCI^®^), β-CD (Sigma^®^), maltosyl β-CD (β-CD-G2, Ensuiko Sugar Reffining Co., Ltd.), glucosyl β-CD (β-CD-G1, Bio Research Coporation of Yokohama), and acarbose (Wako^®^)] in 25 mM acetate buffer pH 6.0 at 50°C for 2.5 hr.

In addition, to confirm the specificity of pullulan cleavage, products produced by *Kp*PUL (commercial pullulanase, Sigma^®^) and the purified r*Me*PUL were also compared. 0.2 U/mL of both PULs were incubated with 0.5% (w/v) pullulan in 25 mM acetate buffer pH 6.0 at 25°C and at 50°C for *Kp*PUL and r*Me*PUL, respectively. The products were analysed at 0, 0.5, 1, 3.5, 6, and 24 hr after incubation.

The products from the enzymatic reactions were spotted onto TLC plates (TLC Silica gel 60 F_254_, Merck). The TLC was run in a TLC tank equilibrated with acetonitrile:ethylacetate:1-propanol:water (85:20:50:60). Finally, the result was visualised by heating at 110°C for 10 min after staining with orcinol solution orcinol solution (sulphuric acid:ethanol:water (5:27:13) containing 0.2%(w/v) orcinol) (Waksmundzka-Hajnos et al., 2008; Wangpaiboon et al., 2021).

#### 2.6.2 High-performance anion-exchange chromatography with pulsed amperometric detection analysis (HPAEC-PAD)

To analyse the product pattern according to the debranching activity of PUL on pullulan substrate, The 0.2 U/mL of both PULs were incubated with 0.5% (w/v) pullulan in 25 mM acetate buffer pH 6.0 at 25°C and at 50°C for *Kp*PUL and r*Me*PUL, respectively. The products were analysed at 0.5 hr after incubation.

The enzymatic reactions were analysed by HPAEC-PAD using a CarboPac™ PA-100 column with a flowrate of 1 mL/min at 30°C. The products were eluted with a linear gradient of 0 – 0.5 M sodium acetate in 150 mM NaOH solution for 35 min, followed by 0.5 M sodium acetate in 150 mM NaOH for 5 min.

#### 2.6.3 Matrix-assisted laser desorption ionization time-of-flight mass spectrometry (MALDI-TOF MS)

Each sample obtained from PUL reactions (section 2.6.2) was desalted by using a mixed bed resin (AG 501-X8, Bio-Rad). The samples were diluted 10-fold prior to mixing with 10 mg/mL of 2,5-dihydroxybenzoic acid with the ratio of 1:1 and spotted onto a MALDI target plate. Masses were analysed with a JEOL SpiralTOF MALDI Imaging-TOF/TOF Mass Spectrometer using spiral mode.

### 2.7 Synergistic action between cassava r*Me*PUL and r*Me*ISA3

To investigate the possible relationship between PUL and ISA3 in starch degradation process, r*Me*ISA3 and r*Me*PUL activities were defined under the same condition; 0.75% (w/v) β-limit dextrin (Megazyme^®^) in 25 mM acetate buffer pH 6.0 at 37°C. For the individual enzyme reactions, 0.2 U/mL of either r*Me*ISA3 or r*Me*PUL was incubated with 0.5% (w/v) β-limit dextrin in 25 mM acetate buffer pH 6.0 at 37°C. For the mixed-enzyme reaction, 0.1 U/mL of each enzyme was added to the reaction and then incubated under the same conditions. The reactions were collected at the time points of 0, 0.5, 1, 3.5, 6, and 24 hr and analysed by HPAEC-PAD and MALDI-TOF MS.

### 2.8 Computational structural analysis by molecular dynamics simulation (MD)

SWISS-MODEL server was employed to constructed homology model of mature *Me*PUL using crystal structure of barley limit dextrinase (E510A mutant) in complex with a 6^II^-O-maltotriosylmaltotriose (branched maltohexaose, PDB ID: 4J3W) (Møller et al., 2015) as a template. Structure of branched maltohexaose in complex with *Me*PUL homology model was also extracted from this crystal structure. H^++^ server was used to protonated all amino acids of this model at pH 6.0 (Gordon et al., 2005; Myers et al., 2006; Aguilar et al., 2012). LEaP module of AMBER20 was employed to solvated the complex in an isomeric truncated octahedral box of TIP3P water model (Jorgensen et al., 1983) with the buffer distance of 13 Å. Sodium ions (Na^+^) were added to neutralise the systems. PMEMD module of AMBER 20 was then used to minimise and simulate the system under the periodic boundary condition as previously described (Wangpaiboon et al., 2022). In the production run, the systems were simulated at 323 K for 50 ns. The Cpptraj module (Roe and Cheatham, 2013) of AMBER 20 was employed to calculated for Root Mean Square Deviation (RMSD) of the complex, and percentage of Hydrogen bond (H-bond) occupation (%H-bond_oc_). RMSD value of all atoms of the protein or ligands were calculated with respect to the minimised structure. The structure was found to be stable in the range of 40 to 50 ns. Therefore, this trajectory was used for analysis. Hydrogen bonds will present if the following conditions are met: (i) a donor-acceptor distance of less than 3.5, and (ii) a donor H-acceptor bond angle of less than 120°. H-bond occupations > 50% were taken into account (Klaewkla et al., 2021). The binding affinity of each residue to the ligand was calculated based on molecular mechanics/Generalised Born surface area (MM/GBSA) method (Genheden et al., 2012) using MMPBSA.py modules (Genheden et al., 2012) of AMBER20.

### 2.9 Subcellular localisation of *Me*PUL expressed in *Nicotiana benthamiana* leaves

The full-length *Mepul* was cloned into a pCR™8/GW/TOPO^®^ TA vector (Invitrogen™) based on circular polymerase extension cloning technique (Aslanidis and De Jong, 1990; Quan and Tian, 2011). The gene with LR sequences was then subcloned into a pGWB5 vector fused with the C-terminal green fluorescent protein (GFP) (Nakagawa et al., 2007) using Gateway^®^ LR Clonase^®^ II (Invitrogen™). The pGWB5-*Mepul* was transformed into an *Agrobacterium tumefaciens* GV3101 by electroporation.

*A. tumefaciens* harbouring pGWB5-*Mepul* and *A. tumefaciens* containing the silencing suppressor *p19* gene were co-infiltrated into 4-week *Nicotiana benthamiana* leaves (Lindbo, 2007; Panpetch and Sirikantaramas, 2021). A pGWB2 bearing *GFP* gene was used as a control for infiltration experiments. After 3 days, localisation of *Me*PUL was observed under a FluoView^®^ FV10i-DOC confocal laser scanning microscope (Olympus Corp.). GFP fluorescence signal was monitored at 488/510 nm of excitation/emission, while chloroplast autofluorescence was observed at 633/664 nm.

## 3 Results

### 3.1 Sequence analysis, gene expression, and protein production

**Figure 1:**
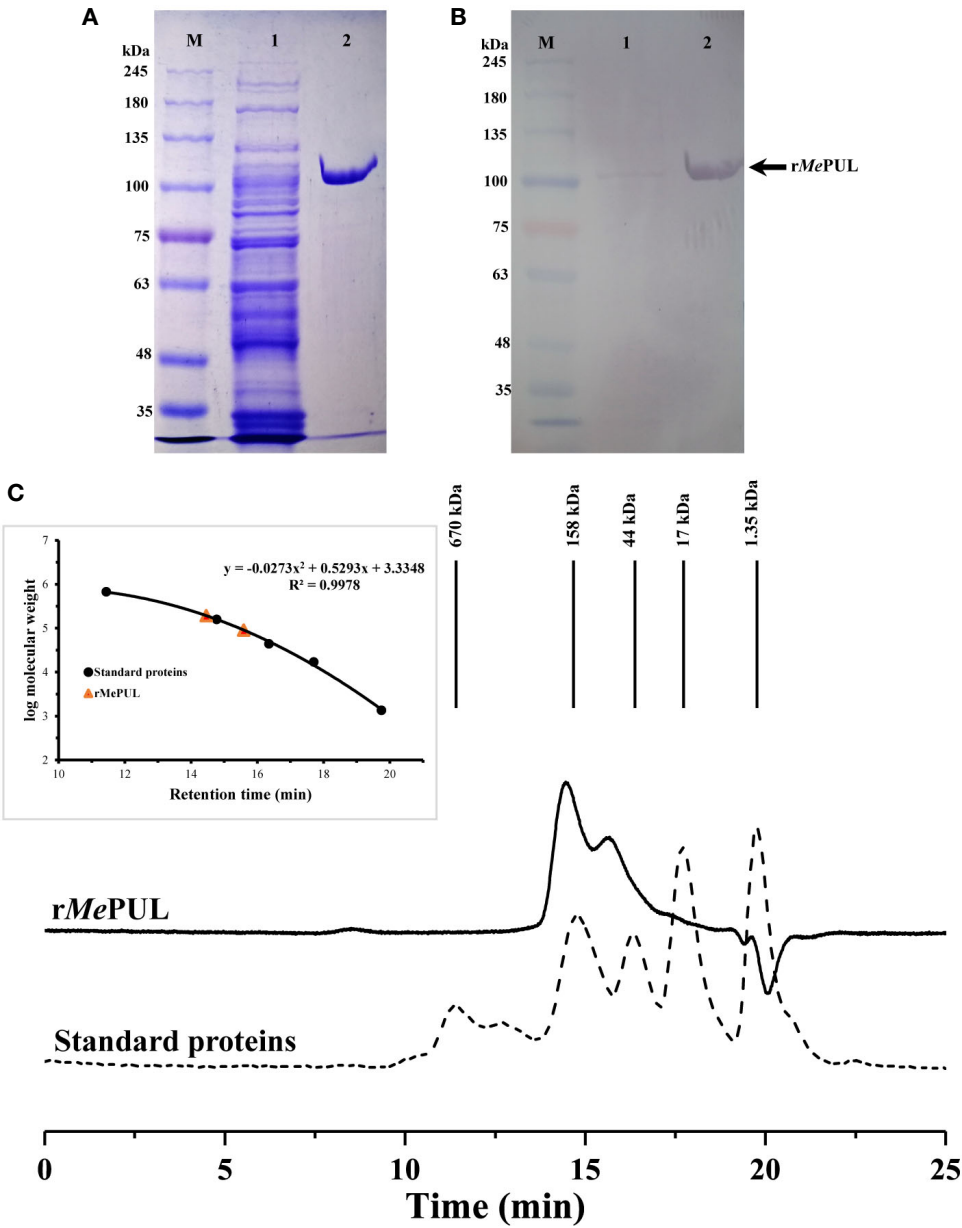
Molecular weight analysis of r*Me*PUL. **(A, B)** SDS-PAGE and western blot analyses. Crude extract and pooled activity fractions of r*Me*PUL purified by a Histrap™ column were analysed and visualised by SDS-PAGE and Western blot analysis. Lane M: TriColour Broad Protein Ladder (3.5 – 245 kDa) (BiotechRabbit); lane 1: crude protein; lane 2: purified r*Me*PUL (~ 100 kDa). **(C)** Non-denaturing gel filtration analysis. Concentrated pooled purified fraction was loaded onto a gel filtration column (MAbPac™ SEC-1). Protein standards (shown by dashed line) are as follows; (1) vitamin B12 (1.35 kDa), (2) myoglobin (17 kDa), (3) ovalbumin (44 kDa), (4) γ-globulin (158 kDa), and (5) thyroglobulin (670 kDa).

The full-length *Mepul* sequence with an open reading frame of 2,904 bp (**Supplementary Figure S1**) was successfully obtained from 5′ RACE and encoded 967 amino acid residues. According to the full peptide sequence, the first 74 residues belonged to a transit peptide, as predicted by the ChloroP1.1 server (**Supplementary Figure S2**). For gene expression in *E. coli*, mature *Mepul* without the transit peptide was directly cloned into a pET21b expression vector and successfully expressed in soluble form in *E. coli* SoluBL (DE3) transformant. After purification by a metal ion affinity column, SDS-PAGE and western blot analyses showed a single band of the purified r*Me*PUL. The apparent molecular mass of ~ 100 kDa corresponded to that calculated for monomeric protein the by Expasy Compute pI/Mw tool (https://web.expasy.org/compute.pi/) which was 100776.98 Da (including 6x His). Nevertheless, r*Me*PUL exhibited two protein peaks by gel filtration chromatography with the calculated sizes of 197 and 84 kDa, as shown in **Figure 1**: the higher MW peak was a major form.

### 3.2 Biochemical characterisation

**Figure 2:**
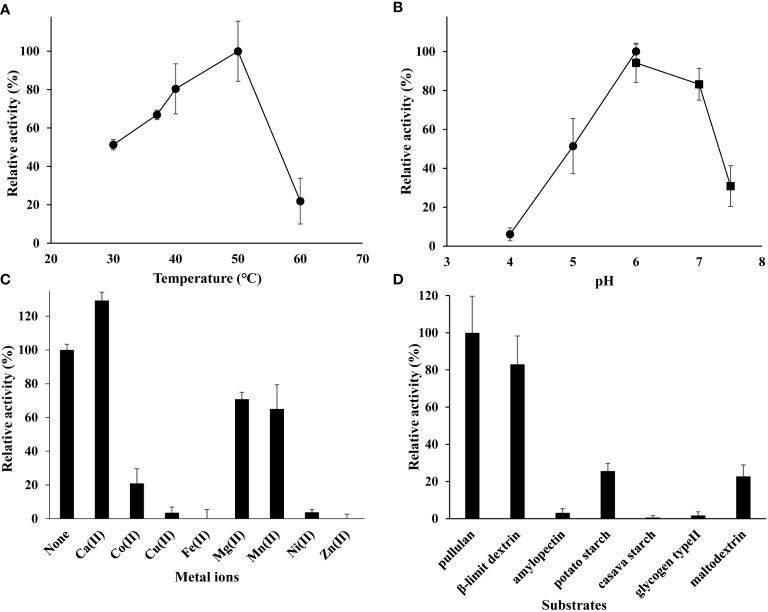
Biochemical characterisations of purified r*Me*PUL. **(A)** Optimum temperature for debranching activity of r*Me*PUL determined at temperatures ranging from 30 – 60°C. **(B)** Optimum pH for debranching activity of r*Me*PUL measured at pH 4.0 – 7.5 [acetate buffer (●) (pH 4.0, 5.0, 6.0); potassium phosphate buffer (▪) (pH 6.0, 7.0, 7.5)]. **(C)** The effect of metal ions [10 mM of Ca^2+^, Co^2+^, Cu^2+^, Fe^2+^, Mg^2+^, Mn^2+^, Ni^2+^, and Zn^2+^] on debranching activity of r*Me*PUL. **(D)** Substrate specificity of the r*Me*PUL on various types of branched substrates (pullulan, β-limit dextrin, potato amylopectin, potato starch, cassava starch, glycogen type II, and maltodextrin). Enzyme activity was analysed by DNS assay.

The r*Me*PUL exhibited optimal temperature and pH at 50 °C and pH 6.0, respectively (**Figure 2A, B**). In addition, EDTA treatment could increase enzyme activity of around 30%. Only Ca^2+^ ion could promote r*Me*PUL activity ~ 30% higher than the control reaction. Conversely, Co^2+^, Cu^2+^, Zn^2+^, Mg^2+^, and Ni^2+^ ions could apparently inhibit *Me*PUL activity (**Figure 2C**). Under assay conditions with different branched substrates, r*Me*PUL exhibited the highest activity against pullulan, followed by β-limit dextrin with ~ 80% activity (**Figure 2D**). r*Me*PUL showed little hydrolysis activity on potato starch and maltodextrin, at ~ 20% compared with that of pullulan. In contrast, amylopectin, cassava starch, and glycogen type II were barely cleaved by r*Me*PUL.

**Figure 3:**
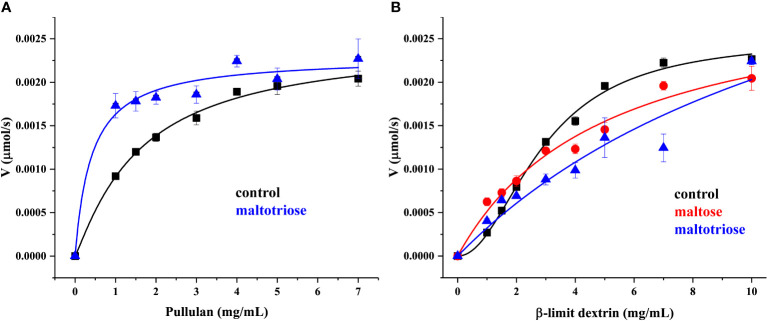
Kinetics study of r*Me*PUL. **(A, B)** Pullulan and β-limit dextrin were used as substrates, respectively. Black lines represent kinetics without addition of maltooligosaccharide (control) and meanwhile red and blue lines show enzyme kinetics with the presence of 1 mM maltose and maltotriose, respectively. The reactions were carried out in 25 mM acetate buffer pH 6.0 at 50°C. The debranching activity was determined by DNS assay.

**Figure 4:**
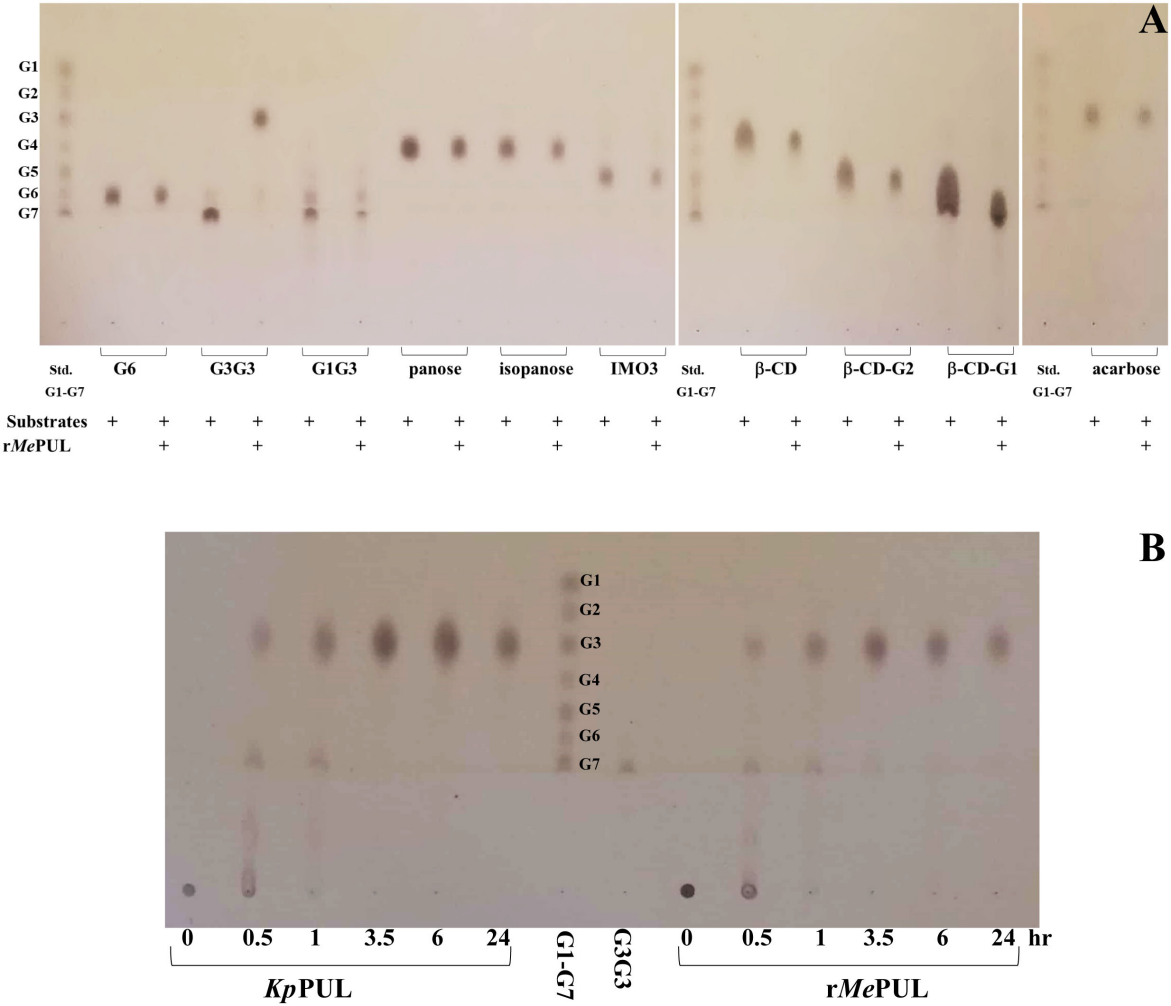
Product analysis by TLC of PUL action. **(A)** TLC analysis of r*Me*PUL activity on 2.5 mg/mL various oligosaccharides with different linkages after incubating in 25 mM acetate buffer pH 6.0 at 50°C for 2.5 hr. **(B)** TLC analysis of products produced from *Kp*PUL (commercial pullulanase, Sigma) and r*Me*PUL. The 0.2 U/mL of both PULs were incubated with 5 mg/mL pullulan in 25 mM acetate buffer pH 6.0 at 25°C for *Kp*PUL and at 50°C for r*Me*PUL. The products were analysed at 0, 0.5, 1, 3.5, 6, and 24 hr after incubation.

In addition, the kinetics study showed that r*Me*PUL catalysis could be fitted well with Hill plots, with both pullulan and β-limit dextrin substrates (represent in black lines in **Figure 3A, B**). In contrast, pullulan and β-limit dextrin kinetics in the presence of maltose or maltotriose could not be fitted with the same kinetic model. Notably, addition of maltotriose could obviously reduce the *k* value when pullulan was used as substrate (**Figure 3A**), while both *k_cat_* and *K_m_* values of β-limit dextrin kinetics in the presence of maltose or maltotriose were highly increased (**Figure 3B**; **Supplementary Figure S3**, and **Table 1**).To further investigate the linkage-specificity of substrates for r*Me*PUL, the enzyme was incubated with different oligosaccharides harbouring various types of linkages. Among those substrates, maltotriosyl-maltotriose was the only substrate that was completely hydrolysed, releasing maltotriose. On the other hand, for other oligosaccharide substrates including maltohexose, β-cyclodextrin, maltosyl-β-cyclodextrin, glucosyl-β-cyclodextrin, panose, isopanose, glucosyl-maltotriose, isomaltotriose, and acarbose, no catalysis was apparently observed even after 2.5-hr incubation with the enzyme (**Figure 4A**). Additionally, the effect of β-CD on the inhibition of r*Me*PUL in pullulan hydrolysis was also investigated. The result clearly showed that the pullulan cleavage was almost completely inhibited which only the relative activity of 2.4% was shown compared to the control reaction.

**Table 1:** kinetic parameters of r*Me*PUL.

Substrate	*k* or *K_m_* (mg/mL)	V_max_ (x 10^-3^) (μmol s^-1^)	*n*	*k_cat_* (s^-1^)	Kinetic model
pullulan	1.6 ± 0.3	2.5 ± 0.3	1.1 ± 0.2	333.2 ± 35	Hill
pullulan +	0.4 ± 0.1	2.3 ± 0.1		309 ± 15	MM^ǂ^
1 mM maltotriose					
β-limit dextrin	2.9 ± 0.1	2.5 ± 0.7	2.1 ± 0.1	335.9 ± 10	Hill
β-limit dextrin +	5.1 ± 0.7	3.1 ±0.2		417.9 ± 31	MM
1 mM maltose					
β-limit dextrin +	6.3 ± 1.3	3.7 ± 0.4		499.9 ± 54	MM
3 mM maltose					
β-limit dextrin +	14.2 ± 5.8	4.9 ± 0.1		659.8 ± 19	MM
1 mM maltotriose					

^ǂ^Mechaelis-Menten model.

**Figure 5:**
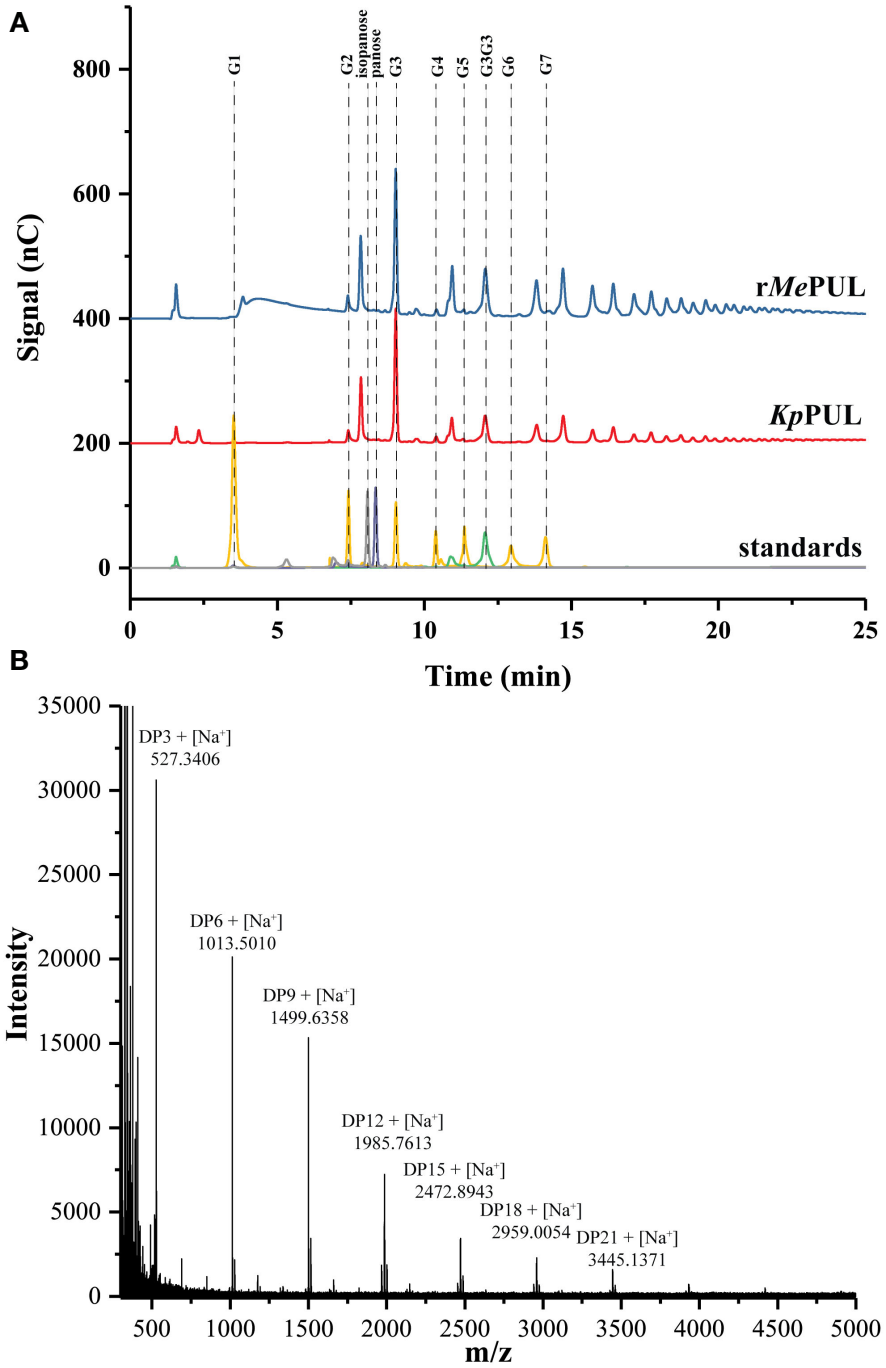
Pullulan hydrolysis. **(A)** HPAEC-PAD analysis and **(B)** MALDI-TOF MS analyses of products synthesised from r*Me*PUL. The 0.2 U/mL of r*Me*PUL and *Kp*PUL were incubated with 5 mg/mL pullulan in 25 mM acetate buffer pH 6.0 at 25°C for *Kp*PUL and at 50°C for r*Me*PUL for 0.5 hr.

When pullulan was used as substrate, the patterns of products released from both r*Me*PUL and a commercial pullulanase, *Klebsiella pneumoniae* pullulanase (*Kp*PUL), were as expected rather similar, as shown in the TLC image (**Figure 4B**). At 0.5 hr of incubation, series of oligosaccharides harbouring masses of maltotriose building block were apparently released. Notably, at 3.5 h onwards, maltotriose was apparently obtained as a main product (**Figure 4B**). In addition, the product patterns were also confirmed by HPAEC-PAD and MALDI-TOF MS, where the main peaks corresponded to maltotriose and maltotriosyl-maltotriose (**Figures 5A, B**).

### 3.3 Prediction of a catalytically competent binding conformation

**Figure 6:**
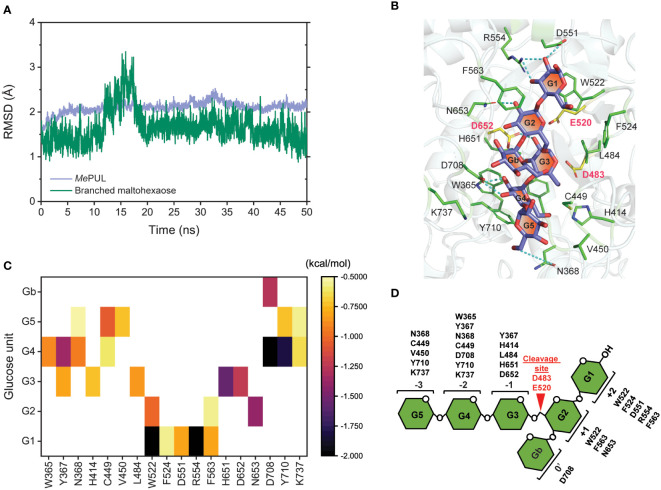
Molecular dynamics simulation analysis of mature *Me*PUL/branched maltohexaose complex. **(A)** Time evolution of all-atom RMSD. **(B)** The binding conformation and H-bond network of branched maltohexaose in *Me*PUL binding site. **(C)** The per-residue decomposition free energy of residues within the substrate-binding track. **(D)** The proposed oligosaccharide binding subsites of *Me*PUL.

Determination of a catalytically competent binding conformation of the enzyme is important to describe how the enzyme acts on its specific substrate. Although r*Me*PUL exhibited the highest activity on pullulan (**Figure 2D**), this substrate is not found in plants but generally obtained from fungi such as *Aureobasidium pullulans* (Pandey et al., 2021). Additionally, the *Me*PUL active site could not be fully occupied by pullulan substrate since the pullulan structure lacks glucosyl branch point at the position of 0′ subsite in the active site. Thus, 6^II^-O-maltotriosylmaltotriose or branched maltohexaose which is an interior structure of β-limit dextrin, a more close-to-nature substrate, was chosen instead of pullulan to evaluate the interaction and conformation of mature *Me*PUL when binding to its substrate by using molecular dynamics (MD) simulation analysis. The RMSD plots showed that *Me*PUL was likely stable at 10 ns of simulation, while branched maltohexaose was stable after 20 ns of simulation (**Figure 6A**). High fluctuation of RMSD value was observed for branched maltohexaose due to its flexibility. Therefore, the last 10 ns trajectories of simulations were selected for further analysis including H-bond network and binding free energy. The binding conformation and H-bond network of branched maltohexaose in *Me*PUL binding site were illustrated in **Figure 6B**. Branched maltohexaose substrate laid along the *Me*PUL binding track formed H-bonds with N368, D551, R554, D652, N653, and D708. The per-residue decomposition free energy of crucial residues within the substrate-binding track was calculated using the MM/GBSA method (**Figure 6C**). The result revealed that W365, Y367, N368, H414, C449, V450, L484, W522, F524, D551, R554, F563, H651, D652, N653, D708, Y710, and K737 of *Me*PUL significantly interacted with branched oligosaccharides. Additionally, residue conservation analysis with searching 149 related sequences, such as pullulanases, α-1,6-glucosidases, etc. (**Supplementary data II**), with the ConSurf server (Ashkenazy et al., 2016) showed that the best predicted binding residues are highly conserved (> 80%), except F563, D708, and K737 (**Supplementary Figure S4**) which exhibited a lower cutoff (<80%).

### 3.4 Synergistic activity between *Me*PUL and *Me*ISA3

**Figure 7:**
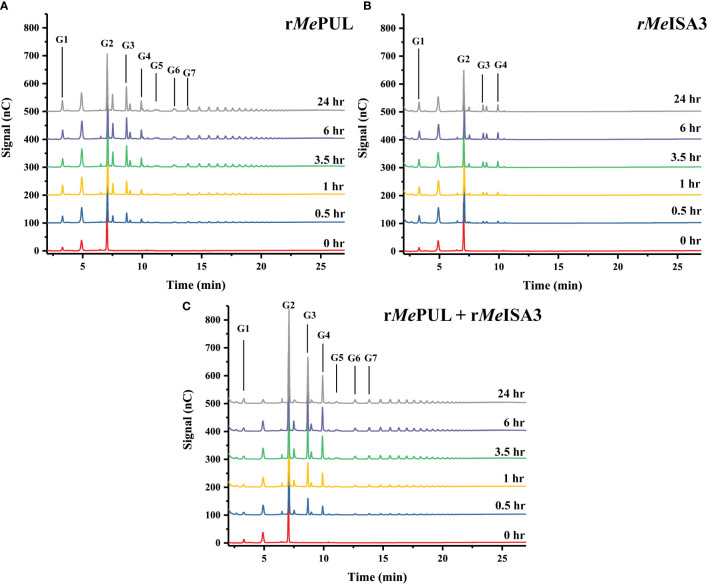
Synergistic activity between r*Me*PUL and r*Me*ISA3 on β-limit dextrin. 0.2 U/mL of **(A)** r*Me*PUL or **(B)** r*Me*ISA3 as well as **(C)** mixture of 0.2 U/mL r*Me*PUL + 0.2 U/mL r*Me*ISA3 were incubated with 0.5% (w/v) β-limit dextrin in 25 mM acetate buffer pH 6.0 at 37°C for 24 hr and products were analysed by HPAEC-PAD.

r*Me*PUL could release a broad range of maltooligosaccharides from β-limit dextrin with degree of polymerisation (DP) longer than 7, while products of r*Me*ISA3 were mainly short maltooligosaccharides (DP <5) as shown by HPAEC-PAD (**Figure 7**) and MALDI-TOF MS (**Supplementary Figure S5**). When r*Me*PUL and r*Me*ISA3 were co-incubated, the product pattern was similar to that of single r*Me*PUL, however, the peak areas of DP 2 - 4 were obviously greater (>2 times) than a summary of the peak area obtained from each single r*Me*PUL and r*Me*ISA3 reaction (**Supplementary Figure S6**).

### 3.5 Subcellular localisation of *Me*PUL

**Figure 8:**
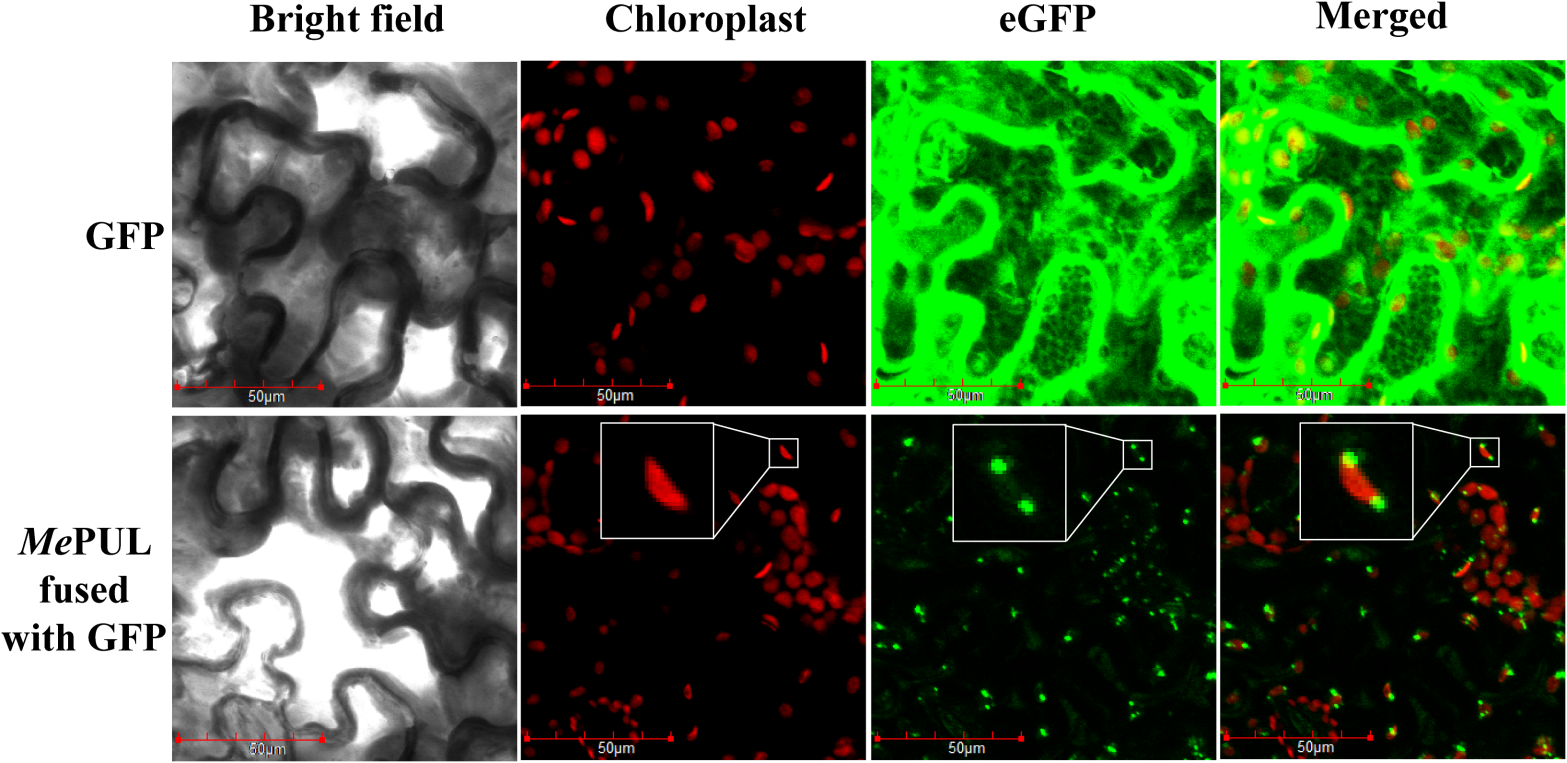
Subcellular GFP-tagged *Me*PUL localisation in *Nicotiana benthamiana* leaves. Confocal microscopic images of *N. benthamiana* leaf epidermal cells infiltrated with pGWB2-*GFP* (control; upper panel) and pGWB5-*Mepul* (lower panel). Chloroplast autofluorescence (chloroplast), GFP fluorescence (eGFP), and merged images are shown. Plastidial *Me*PUL-GFP localisation is shown as enlargements in the insets. Scale bars: 50 μm.

The pGWB5-*Mepul* and the silencing suppressor *p19* were co-expressed in *Agrobacterium tumefaciens* GV3101 infiltrated in 4-wk *N. benthamiana* leaves. The in-planta assay showed that GFP-tagged *Me*PUL fluorescence signals were detected in the chloroplasts (**Figure 8**, bottom panel, inset). This corresponded to the presence of transit peptide sequence predicted *in silico*. Conversely, the eGFP protein, as control, was overexpressed and localised throughout the plant cells.

## 4 Discussion

The protein sequence of r*Me*PUL was identical to the cassava pullulanase in the Phytozome database [Manes.10G051700.1.p]. Comparison of the *Me*PUL to those of other plants and bacteria showed some sequecne similarity - around 37 – 74%, including to *Hordeum vulgare* pullulanase (*Hv*LD; GenBank AAD04189.1, 65%), *Oryza sativa* pullulanase (*Os*PUL; GenBank BAA09167.1, 60%), *Solanum tuberosum* pullulanase (*St*PUL; GenBank XP_006361707.1, 71%), *Arabidopsis thaliana* pullulanase (*At*PUL; GenBank NP_196056.2, 72%) *Zea mays* pullanase *(Zm*PUL; GenBank NP_001104920.1, 74%), and *Spinacia oleracea* pullulanase (*So*PUL; GenBank CAA58803.1, 71%), and *Klebsiella pneumoniae* pullulanase, a bacterial pullulanase (*Kp*PUL: PDB 2FGZ, 37%). The sequence alignments are presented in **Figures S2, S7**. However, *Me*PUL showed very low similarity compared with other types of *Manihot esculenta* debranching enzymes (*Me*ISA1: GenBank AUZ20772.1, 23%; *Me*ISA2: GenBank AUZ20773.1, 24%; and *Me*ISA3: GenBank AUZ20774.1, 29%) shown in **Figure S8**. Although they are members of the same Glycoside hydrolase (GH) 13 family, ISAs are subcategorised into subfamily 11. The *Me*PUL belongs to GH 13 subfamily 13 (pullulanase subfamily) based on sequence similarity (Machovič and Janeček, 2008). The *Me*PUL also comprised 4 domains of N-terminal domain, CBM48, catalytic domain, and C-terminal domain similar to that of *Hv*PUL as shown in **Figure S2** (Vester-Christensen et al., 2010b).

Fortunately, although the *Me*PUL comprised a total of 14 cysteine residues in the mature protein sequence, it was successfully expressed in soluble form in *E. coli* SoluBL (DE3) transformant. Noticeably, protein MW obtained from gel filtration chromatography under non-denaturing condition was ~ 2-fold higher than the MW shown by SDS-PAGE analysis (**Figure 1**), which implied that *Me*PUL could be naturally present as a homodimer. Unlike other debranching enzymes from cassava, the *Me*ISA1 and *Me*ISA2 essentially exist as catalytically active hetermeric enzymes with various molar ratios (Panpetch et al., 2018b), while *Me*ISA3 is a monomeric enzyme (Panpetch et al., 2018a). Additionally, *So*PUL was reported as a monomeric protein (Renz et al., 1998) and partially purified *St*PUL from potato tuber present as a dimer analysed under nondenaturing condition however 3 protein bands were shown on SDS-PAGE gel (Ishizaki et al., 1983).

An *in vitro* biochemical assay of r*Me*PUL disclosed that r*Me*PUL has pH optimum in the slightly acidic pH range and is a metal ion-dependent enzyme (**Figure 2**). Surprisingly, the optimum temperature of r*Me*PUL was at 50°C (**Figure 2A**), while for r*Me*ISA3 it was at only at 37°C (Panpetch et al., 2018a) even though they both originate from the same organism. r*Me*PUL exhibited the highest activity in acetate buffer pH at 6.0 (**Figure 2B**) like r*Me*ISA3. The activity dropped by up to 50% at pH 5.0 and 7.0, with a sharp drop of activity below 5 or above 7, similar to the reported optimum pH of isoamylases from potato and maize (Ishizaki et al., 1983; Doehlert and Knutson, 1991; Rahman et al., 1998). Nevertheless, both optimal pH and temperature between *Me*PUL and *St*PUL were similarly (Ishizaki et al., 1983). Moreover, only Ca^2+^ ion could promote the activity of r*Me*PUL (**Figure 2C**) corresponded to the presence of embedding of Ca^2+^ in *Hv*PUL crystal structure (Vester-Christensen et al., 2010b), while the opposite was true for *St*PUL where Ca^2+^ ion lowered the enzyme activity by around 40%. However, enzyme activity was completely inhibited by Cu^2+^ and Zn^2+^ (**Figure 2C**), correlating with previous work of Iwaki and Fuwa (1981) (Iwaki and Fuwa, 1981), which reported that 1 mM Cu^2+^, Zn^2+^, Ag^2+^, or Cd^2+^ inhibited rice endosperm debranching enzyme. Moreover, we also found that r*Me*PUL activity could be enhanced after treating with EDTA. Possibly, there was contamination of some metal ion inhibitors in gene expression and protein purification processes

To evaluate the substrate specificity of r*Me*PUL, the enzyme was incubated with various substrates (**Figure 2**). r*Me*PUL exhibited the highest activity toward pullulan (**Figure 2A**). This confirmed that this debranching enzyme was pullulanase-type, while isoamylase-type debranching enzyme clearly showed no activity on pullulan at all (Panpetch et al., 2018b; Panpetch et al., 2018a). Notably, r*Me*PUL was unable to cleave substrates harbouring highly branched structures, especially amylopectin, cassava starch, and glycogen. However, compared to no activity on cassava starch, r*Me*PUL could digest potato starch weakly, with ~20% of its activity on pullulan. This possibly resulted from less complexity of the intrinsic structure of potato starch than that of cassava. Hence, *in vitro* results could support the trimming model hypothesis of starch granule synthesis, proposing that PUL could partially substitute for ISA1 activity (Dinges et al., 2003; Wattebled et al., 2005; Fujita et al., 2009). Importantly, r*Me*PUL also preferentially digested β-limit dextrin at a high level as shown in **Figure 2D**. This might be because its structure is rather similar to that of α-limit dextrin or pullulan-like polymers found in plants, which are both produced by amylase action in starch degradation. Moreover, the results from protein localisation indicated that *Me*PUL was specifically localised to the plastid organelles (**Figure 8**) such as chloroplast and amyloplast, where starch degradation occurs in plant cells (Zeeman et al., 2010). Taken together, these data along with some previous reports on target localisation to plastid organelles support the notion that *Me*PUL principally participates in starch degradation processes (Zeeman et al., 2010). Additionally, unlike other PULs from cereals, such as rice (Yamasaki et al., 2008) and barley (Mcdougall et al., 2004), transglycosylation activity could not be observed in r*Me*PUL.

Enzyme kinetic analysis with r*Me*PUL acting on pullulan and β-limit dextrin was fitted with Hill plots, indicating the cooperative manner of the enzyme. Conversely, it was still unclear why addition of some maltooligosaccharides such as maltose and maltotriose could obviously change the kinetic pattern of r*Me*PUL (from Hill to Michaleis-Menten). Notably, maltotriose, the main product of pullulan hydrolysis, was a potential positive effector of the r*Me*PUL action, since it could clearly promote binding affinity (*k* or *K_m_*) of the enzyme to pullulan substrate. However, maltose and maltotriose could also increase *k_cat_* around 1.2 - 1.7 folds for β-limit dextrin hydrolysis (**Figure 3B**, **Supplementary Figure S3**, and **Table 1**). It might be that these maltooligosaccharides produced during starch degradation (Zeeman et al., 2010) act as allosteric modulators to some enzymes participating in the starch degradation process.

Although *Me*PUL exhibited very low sequence similarity to *Kp*PUL (Mikami et al., 2006) (**Supplementary Figure S7**), their product patterns of on pullulan degradation were rather similar, mainly reflecting the release of maltotriose and maltotriose-containing oligosaccharides (**Figure 4B**). However, pullulan does not entirely consist of maltotriose building blocks (Catley and Whelan, 1971; Carolan et al., 1983); it also contains maltooligosaccharides with different DPs, such as maltose (DP2) and maltotetraose (DP4), which could also be detected in HPAEC analysis from r*Me*PUL and *Kp*PUL reactions. r*Me*PUL specifically hydrolysed α-1,6 linkage between maltoriose units but no hydrolytic activity on linear α-1,4-linked oligosaccharides was observed (**Figure 4A**), strongly indicating that *Me*PUL is a pullulanase type I (Møller et al., 2016). Cyclic oligosaccharides such as β-cyclodextrin (β-CD) and α-1,6 branched β-cyclodextrins (β-CD-G1 and β-CD-G2) were not cleaved by r*Me*PUL. Furthermore, the pullulan cleavage was almost completely inhibited by β-CD as judged by DNS assay. This corresponded to the competitive inhibition of debranching activities of *Kp*PUL and *Hv*LD by CDs, where the cyclic structure of cyclic oligosaccharides can productively bind at subsites +1, +2, and 0′, as shown in superimposition structures of *Me*PUL and *Hv*LD (Vester-Christensen et al., 2010a; Møller et al., 2015) (**Supplementary Figure S9**). Although β-CD-G1 and β-CD-G2 contained an α-1,6 branch, the glucosyl and maltosyl branches were not appropriately positioned at catalytic cleavage site (**Supplementary Figure S9**), corresponding to complex structure of *Hv*LD with 6-S-(α-D-maltosyl)-6-deoxy-6-thiocyclomaltoheptaose (Møller et al., 2015). Therefore, β-CD-G1 and β-CD-G2 could not be hydrolysed by r*Me*PUL. Interestingly, r*Me*PUL could not digest the α-1,6 linkage of glucosyl-maltotriose (G1G3), panose or isopanose. The experimental data demonstrated that the active site of r*Me*PUL required longer substrates in order to make productive contacts with the longer binding site. This information acorrelated with computational analysis, which showed that the *Me*PUL-branched maltohexaose complex exhibited high binding energy at +2, +1, -1, -2, and -3 sites (**Figure 6C**). The computational and bioinformatic data revealed that the residues W365, Y367, N368, H414, C449, V450, L484, W522, F524, D551, R554, F563, H651, D652, N653, D708, Y710, and K737 were potentially essential for substrate binding based on the results of the MM/GBSA method and conserved sequence analysis. Most of these residues, which bound with ligands (**Figure 6B**), were highly conserved at the same positions in the 3D-structures of *Me*PUL, *Hv*LD, (Møller et al., 2015), and *Kp*PUL (Mikami et al., 2006) even though they did not show high similarity of overall protein sequences. Notably, two out of those residues, F563 and D708 located near the branch point of branched maltohexaose, showed 80% lower score of the cutoff conservation. Perhaps these variable residues might play a role in imposing the substrate specificity. Proposed oligosaccharide binding subsites of *Me*PUL are shown in **Figure 6D**.

To analyse the relationship between *Me*PUL and *Me*ISA3 debranching activities in starch degradation, mixed-enzyme reaction was performed on β-limit dextrin substrate (**Figure 7**). The results suggested that r*Me*PUL preferentially cleave longer chains in broad ranges of maltooligosaccharides and meanwhile r*Me*ISA3 liberated only short-chain maltooligosaccharides (**Figure 7** and **Figure S4**). This suggested that *Me*PUL might play a major role in starch debranching of cassava starch catabolism. Interestingly, when r*Me*ISA3 and r*Me*PUL were present together in the same reaction, the debranching activity of short-chain oligosaccharides (DP 2 - 4) was apparently increased (**Figure 7** and **Figure S6**). This result implied that *Me*ISA3 and *Me*PUL worked synergistically, possibly by providing favourable substrates for each other by removal of some unfavourable branched points due to their different substrate preferences. Importantly, considering the prospect of an heterocomplex between r*Me*ISA3 and r*Me*PUL, no multimeric r*Me*ISA3/r*Me*PUL complex was observed by gel filtration chromatography (**Supplementary Figure S10**). This demonstrated that they should work separately for their debranching activities.

Some previous studies *in vivo* revealed that deficiency of PUL in rice (Fujita et al., 2009) and maize (Wong et al., 2003) slightly effected on an accumulation of water-soluble glucan. Although *Me*ISA3 and *Me*PUL seemed to exhibit overlapping activities in debranching processes, cassava starch catabolism may not be efficient when only either PUL or ISA3 are present, which contrasts with some previous reports from other plant species (Wong et al., 2003; Delatte et al., 2006; Fujita et al., 2009). Importantly, both PUL and ISA3 have been conserved in most plants. Previous *in vivo* studies showed the relationship between these two enzymes, but they did not clearly unravel the reason for the conservation of both enzymes. Nevertheless, our results show that both PUL and ISA3 are necessary for efficient starch degradation *in vitro*: their synergistic action may account for why these two types of debranching enzymes are typically present in plant cells.

## 5 Conclusion

Pullulanase from *Manihot esculenta* Crantz ‘KU50’ was successfully cloned, expressed, and biochemically characterised. This debranching enzyme was clearly categorised as pullulanase type I due to its preference for cleaving α-1,6 linkages of pullulan and β-limit dextrin. The debranching action of PUL is conserved in most plants, both mono- and di-cotyledons, and is located in plastids. The present work demonstrates the synergistic action between *Me*PUL and *Me*ISA3, which is important for efficient starch degradation in cassava and which may have implications for biotechnology innovation in relation to harnessing starch for food and materials applications.

## Data availability statement

The original contributions presented in the study are included in the article/**Supplementary Material**. Further inquiries can be directed to the corresponding author.

## Author contributions

KW, TC, and PP: conceptualization. KW, TC, MK, and PP: methodology. KW, TC, and PP: formal analysis. KW, TC, MK, RF, and PP: investigation. TC and PP: resources. TC and PP: data curation. KW, TC, and MK: writing - original draft. KW, TC, RF, and PP: writing - review & editing. KW, TC, MK, and PP: visualization. KW and PP: supervision. PP: funding acquisition. PP: Project Administration. All authors contributed to the article and approved the submitted version.
