# Supplementary material for: Cassava pullulanase and its synergistic debranching action with isoamylase 3 in starch catabolism
Source: Front Plant Sci. 2023 Jan 27;14:1114215. doi: 10.3389/fpls.2023.1114215 (PMC9911869; doi:10.3389/fpls.2023.1114215)
Supplement: Supplementary file 1 [file DataSheet_1.docx]

**Supplementary data I**

Full length open reading frame

ATGTCTATAGTAGTTTCCAAATCGCTTCCATCAGTTTCTCCTCCTTCGATTCCCAATATTCACTTTTCCACGTTCTCTTCACCATCTTCTTCAAGAAGTCGTTTAATTCTGAGACAATATCAATGCCACCACCTCCCTTTTCCCCTTTGTTTTAGAACTTTTCATAGATGCCCAATTCGCTGCTGTTCTTCCTCCTCCTCTATGGCTCTTCATGAAGTTTCCGGTTCCACTCCCACTTCTGAGTTGCAAGATTGCTTGTTGTACTCGAGGGCCTTCTGGGTTACAAAATCTATAATTGCATGGAATGTAGATGTTGGAACTGATGGTTCTTGCTTTTTGTATGCTAGTCAGAATGCTGCATTATCCATTACTGAATCTGGCGTACAAGGCCATGACGTGAAATTTAAGCTTGAGCAAGATGATGGTGGGCTTCCAGAAAAAGTAGTTGCAAAATTTCCTCATATTCGGGGTTATAGAGCTTTCAAAGTGCCGCCTTTTGTTGATACCATATCTCTTCTCAAATGCCAATTAGCAGTTGCAGCATTTAACTTTGATGGAAAGTGCAGCAATGCTACTGGTCTGCAGTTACCAGGTATTTTGGATGAATTGTTCTCATATGATGGTCCCCTTGGCGCACATTTTGCCAAAGATGCTGTATCCCTTTATCTTTGGGCTCCCACTGCTCAAGCAGTTCGGCTATGCATTTATAAGGATTCGTTTACCAGCAATCCTACAGAAACTGTTCAGCTCATGGAGGTTAATGGAGTTTGGAGTACTAAAGGGCCAAAAGATTGGGAAGGTTGTTATTATGTGTATGAAGTATCAGTCTACCATCCATCCACCTTACACATTGAAAAATACTTTGCAAATGATCCATATGCTAGAGGGCTCTCATCAGATGGCAAGCGGACATTATTGGTGAATCTTGATTCTGATACTTTAAAACCTGAAAGATGGGACATATTAGCAGATGAAAAACCCACTTTACTTTCTTTCTCTGATATAAGTATTTATGAGTTGCATATAAGAGATTTTAGTGCCAATGATGATACTGTGCATTCTGACCTTCGGGGTGGCTATCTAGCCTTCACTGTAGAGGATTCAGCAGGTGTACTTCATCTAAAGAAATTATCAAGTGCTGGCCTTACTCATGTTCATTTGCTTCCAGCCTTTCAATTTGCTGGTGTTGACGATGTAAGGGAGAACTGGAAGTCTGTAGATAACAGCATGCTAGAAAAATTACCGCCAGATTCAGCTGAGCAACAAGCTCATATTACTGCATTCCAAAATGATGATGGGTATAATTGGGGGTATAATCCTGTCCTATGGGGGGTCCCTAAAGGAAGTTATGCAAGTAATCCAAATGGTTCTTGCCGCACCATTGAGTTCAGAAAGATGGTTCAGGCAATTAACCGCATTGGTCTCCGGGTTGTGTTGGATGTTGTCTACAATCATTTGCATGGAAGTGGGCCTTTTGATGAGAATTCTGTTCTTGATAAGGTTGTTCCAGGTTATTATTTACGAAGGAACAGTGATGGCTTTATAGAAAATAGTACATGTGTGAACAACACTGCCAGTGAGCATTATATGGTTGAGCGCTTGATCGTTGATGATCTTTTAAGCTGGGCAGTTAACTACAAGATTGATGGATTCCGGTTTGACCTTATGGGCCATATGATGAAAAGTACAATGGTGAAAGCAAAAGATGCACTGCTCAGCCTAACAAAGGAAAGGAATGGAGTTGATGGTTCAAGTATCTACTTATATGGTGAAGGTTGGGACTTTGGTGAGGTTGCTAAAAATGCTCGTGGGATAAATGCTTCCCAGTTCAATCTTGGTGGAACTGGAATTGGGAGTTTTAATGATCGAATACGCGATGCGATGCATGGTGGATCCCCATTTGGCCATCCTCTTCAGCAAGGATTTGTGACTGGTCTTATGTTGCAGCCTAATGGTCATGACCATGGAGGAAAAGATGTTGAAGAACACATGCTTACGATAGCGAAGGATCACATCCAGGCTGGGATGGCTGCAAATTTGAGGGAGTTTGTTCTAATCAATAGCGAAGGGAAAGAGGTGAAAGGATCAGAAATTTTGACTTATGGTGGAGAACCCCTTGCGTATGCTTTATGTCCTACTGAAACAATTAATTATGTTTCTGCTCATGACAATGAAACGCTGTTCGACGTTGTGTGCATGAAGACTCCTATGCAAATATCTGTGGATGAGAGATGCAGATTAAATTATTTGGCAACGAATATGATAGCATTAGCACAGGGAATACCATTTTTTCATGCTGGCGATGAAATGCTACGTTCAAAATCGCTTGATCGTGATTCATACAACTCTGGAGACTGGTTCAACAGACTAGATTTCTCGTACAATTCTAACAATTGGGGTGTTGGCCTTCCACCAAAAGGGAAAAATGAAGGAAACTGGCCATTAATGAAACCCAGATTAGCAGATCCATCCTTCAAGCCTCAAAAATCTCACATCCTTGCTACTGTTGACAATTTTTTAGATGTATTACAAATTCGATATTCTTCTCCACTTTTCCGTTTGACAACAGCTAATGCCATCCAGGAGCGAGTAAGGTTTCATAATACCGGTCCTTCATGGGTCCCTGGCGTCATAGTTATGAGCATTGAAGACGGTCATGAAGGTTTCCCTGGGTTATCTCAGCTGGATCCCATCTACTCGTACATCGTGGTTATATTCAACACTTGTCCCAATGAGATATTGTTTGCCTGCCCTCCGTTGCGTGCAAGAGGCTTTCAGCTACATCCTGTTCAGGTTAAGTCGACTGACAAAGTTGTGAAGAAATCTGCATATGAGCCATTGTCGGGGTGTTTCACTGTTCCACCAATGACAACATCTGTGTTTGTTGAGCCTAGGAAAATTTAA

**Figure S1. Open reading frame of full length *Mepul* gene.** The open reading frame of *Mepul* contains 2,904 bp.

OsPUL MQMLLHANSLLLLAPTTSR-----L--SASASPGRSGTARPLPPPQGTRIPPAPPLAGHG 53

HvPUL ------------------------------------------------------------ 0

ZmPUL --MLLHAGPSFLLAPPPRF-----AAAPSSASPRRSRTPQSSPPTSHFAR-PADPVAQ-R 51

SoPUL MSSLYNPIA----LASSFHHHYPNLRFLPFNFNFITK--LPVS--NS-----FA--IGSS 45

AtPUL MALTLTPTSSVHLLSSI-SVARPRIFAADFNLRSRWRRRRPVTSIS-----------NFR 48

MePUL MSIVVS-KS----LPSVSPPSIPNIHFSTFSSPSSSR--SRLI-LRQYQCHHLP--FPLC 50

StPUL MSALLSSTP----FSTIFAP----SELTRFLLPTQSR--LQLSSLTRYR-------SGFK 43

OsPUL GRPPSPQPR-------R----GRDGV--GEECAAAVASQGFVTDARAYWVTRSLIAWNVN 100

HvPUL ---------MA-----V----GETGASVSAAEAEAEATQAFMPDARAYWVTSDLIAWNVG 42

ZmPUL VRPVAPRPPMA-----T----AEEGASSDVGVAVAESAQGFLLDARAYWVTKSLIAWNIS 102

SoPUL SRSFHSSPLKKDSSCFCCSMAVEVG---SASSVSQSELQGSLNSCRAYWPSKYTFAWNVD 102

AtPUL LRL----PSKTSLHCLCSSSSASSPM-SLEVSSPNSQFLDCLIYSRAYWVTQGVIAWNVD 103

MePUL FRT----FHRCPIRC-CSSSSSMALH-EVSGSTPTSELQDCLLYSRAFWVTKSIIAWNVD 104

StPUL SGT----SRSLNLRC-CSTMPVQD------QSENPSCSQDSLFYSRAFWVTKSIIAWNVG 92

: .**:* : :***:.

OsPUL DQD-TSLFLYASRDATMHVS--DGAIHGYDSKIELEPEHASLPDNVAEKFPFIRSYRTFR 157

HvPUL ELEAQSVCLYASRAAAMSLSPSNGGIQGYDSKVELQPESAGLPETVTQKFPFISSYRAFK 102

ZmPUL DQK-TSLFLYASRNATMCMS--SQDMKGYDSKVELQPENDGLPSSVTQKFPFISSYRAFR 159

SoPUL IG-NGSYYLFASKTAALKFT--DAGIEGYDVKIKLDKDQGGLPANVTEKFPHIRGYSAFK 159

AtPUL VG-EGSCYFYASKSAGLSFS--EDGIDGYDLRIKLEAESGSLPADVIEKFPHIRNYKSFK 160

MePUL VGTDGSCFLYASQNAALSIT--ESGVQGHDVKFKLEQDDGGLPEKVVAKFPHIRGYRAFK 162

StPUL AG-EGECYLYASRKAELCVA--GDGIQGHDVKIKLERTNYGLPQQVIEKFPHIRDYAAFE 149

. ::**: * : .: :.*:* :.:*: .** * ***.* .* :*.

OsPUL VPSSVDVASLVKCQLAVASYDAHGRHQDVTGLQLPGVLDDMFAYTGPLGAVFSDKDVDLY 217

HvPUL VPSSVDVASLVKCQLVVASFGADGKHVDVTGLQLPGVLDDMFAYTGPLGAVFSEDSVSLH 162

ZmPUL IPSSVDVATLVKCQLAVASFDAHGNRQDVTGLQLPGVLDDMFAYTGPLGTIFSEEAVSMY 219

SoPUL APATLDVDSLLKCQLAVAAFSADGACRNATGLQLPGVIDELYSYDGPLGAVFSENTISLY 219

AtPUL VPKDLDIRDLVKSQLAVVCFDAEGRLIQGTGLQLPGVLDELFSYDGPLGAHFTPEGVSLH 220

MePUL VPPFVDTISLLKCQLAVAAFNFDGKCSNATGLQLPGILDELFSYDGPLGAHFAKDAVSLY 222

StPUL VPATLDFQSLVKCQLAVGIFNSYGKCASATGLQLPGILDELFSYTGPLGAVFATEAVSLY 209

* :* *:*.**.* :. * . *******::*::::* ****: *: . :.::

OsPUL LWAPTAQDVRVCFYDGPAG-PLLQTVQLKELNGVWSVTVPRYPENQYYLYEVKVYHPSTS 276

HvPUL LWAPTAQGVSVCFFDGPAG-PALETVQLKESNGVWSVTGPREWENRYYLYEVDVYHPTKA 221

ZmPUL LWAPTAQDVSVSFYDGPAG-PLLETVQLNELNGVWSVTGPRNWENRYYLYEVTVYHQTTG 278

SoPUL LWAPTAQAVSASIFKDPSGGEPLQTVQLIESNGVWSAVGPRTWEGCYYVYEITVYHHSTL 279

AtPUL LWAPTAQAVSVCIYKNPLDKSPMEICPLKEANGVWSTEGACSWGGCYYVYKVSVYHPSTM 280

MePUL LWAPTAQAVRLCIYKDSFTSNPTETVQLMEVNGVWSTKGPKDWEGCYYVYEVSVYHPSTL 282

StPUL LWAPTAQAVEALIYKSPSEADPVEIVQLKELDGVWSAKGPMHWEGCYYVYEVSVYHPSTL 269

******* * ::.. : * * :****. . **:*:: *** :.

OsPUL QVEKCLADDPYARGLSANGTRTWLVDINSETLKPASWDELSDEEPNLESFSDISIYELHI 336

HvPUL QVLKCLAGDPYARSLSANGARTWLVDINNETLKPASWDELADEKPKLDSFSDITIYELHI 281

ZmPUL NIEKCLAADPYARGLSANSTRTWLVDINNETLKPLAWDGLAAEKPRLDSFSDISIYELHI 338

SoPUL RIEKSFAIDPYARGISADVKRTLLADLSSETLKPEGWENLADEKPHLLSPSDISLYELHI 339

AtPUL KLETCYANDPYARGLSADGRKTFLVNLDSDDLKPEGWDNLADKKPCLRSFSDISIYELHV 340

MePUL HIEKYFANDPYARGLSSDGKRTLLVNLDSDTLKPERWDILADEKPTLLSFSDISIYELHI 342

StPUL RIEKCVSNDPYARGLSADGKRTLLVNLDSDDVKPEGWDNLQDEKPNLLSFSDVSIYELHV 329

.: . : *****.:*:: :* *.::..: :** *: * ::* * * **:::****:

OsPUL RDFSAHDSTVDCNSRGGFVHLHFRLFRLNLLNDFCSPPITKHPGRIMETVMQDSAGIRHL 396

HvPUL RDFSAHDGTVDSDSRGGFRAFA----------------------------YQASAGMEHL 313

ZmPUL RDFSAHDSTVDCPFRGGFCAFT----------------------------FQDSVGIEHL 370

SoPUL RDFSAYDLTVHPDLRGGYLAFT----------------------------SQDSAGVNHL 371

AtPUL RDFSANDETVEPENRGGYLAFT----------------------------SKDSAGVKHL 372

MePUL RDFSANDDTVHSDLRGGYLAFT----------------------------VEDSAGVLHL 374

StPUL RDFSASDPTVSHEFQGGYLAFT----------------------------SQDSAGVQHL 361

***** * ** :**: : : *.*: **

OsPUL RKLSAAGLTHVHLLPSFHFASVDDNKSNWKFVDEAQLAKLPPGSDEQQAAIVSIQQEDPY 456

HvPUL RKLSDAGLTHVHLLPSFHFAGVDDIKSNWKFVDECELATFPPGSDMQQAAVVAIQEEDPY 373

ZmPUL KKLSDAGLTHVHLLPSFQFGGVDDIKSNWKCVDEIELSKLPPGSDLQQAAIVAIQEEDPY 430

SoPUL EKLSAAGLTHVHLLPSFQFAEVDDDKKKWKFVDTKRFETLPPDSEEQQAQITAIRDEDGY 431

AtPUL QKLVDAGLTHLHLLPTFQFGDVDDEKENWKSVDTSLLEGLRPDSTEAQARITEIQNDDGY 432

MePUL KKLSSAGLTHVHLLPAFQFAGVDDVRENWKSVDNSMLEKLPPDSAEQQAHITAFQNDDGY 434

StPUL KRLSSAGITHVHLLPTYQFAGVEDEKHKWKYTDIEKLNSFPPDSEEQQALITAIQDEDGY 421

.:* **:**:****:::*. *:* : :** .* : : *.* ** :. ::::* *

OsPUL NWGYDPVLWGVPKGSYASNPDGPSRIIEYRQMVQALNRIGLRVVMDVVYNHLDSSGPFGV 516

HvPUL NWGYNPVLWGVPKGSYASDPDGPSRIIEYRQMVQALNRIGLRVVMDVVYNHLDSSGPCGI 433

ZmPUL NWGYNPVVWGVPKGSYASNPDGPSRIIEYRLMVQALNRLGLRVVMDVVYNHLYSSGPFAI 490

SoPUL NWGYNPVLWGTPKGSYATDPNGPCRIIEFRKMVQALNRIGLRVVLDVVYNHLNSSGPSDD 491

AtPUL NWGYNPVLWGVPKGSYASDPTGPCRIIEFRKMVQALNCTGLNVVLDVVYNHLHASGPHDK 492

MePUL NWGYNPVLWGVPKGSYASNPNGSCRTIEFRKMVQAINRIGLRVVLDVVYNHLHGSGPFDE 494

StPUL NWGYNPVLWGVPKGSYAGNANGPCRIVEFRKMVQALNRIGLRVVLDVVYNHLHASGPFSD 481

****:**:**.****** : * .* :*:* ****:* **.**:******* .***

OsPUL SSVLDKIVPGYYLRRNVNGQIENSAAMNNTASEHFMVDRLTVDDLLNWAINYKVDGFRFD 576

HvPUL SSVLDKIVPGYYVRRDTNGQIENSAAMNNTASEHFMVDRLIVDDLLNWAVNYKVDGFRFD 493

ZmPUL TSVLDKIVPGYYLRRDSNGQTENSAAVNNTASEHFMVDRLIVDDLLNWAVNYKVDGFRFD 550

SoPUL NSVLDKIVPGYYLRRDNDGAIENSTCVNDTASEHFMVERLILDDLKHWAVNYKVDGFRFD 551

AtPUL ESVLDKIVPGYYLRRNSDGFIENSTCVNNTASEHYMVDRLIRDDLLNWVVNYKVDGFRFD 552

MePUL NSVLDKVVPGYYLRRNSDGFIENSTCVNNTASEHYMVERLIVDDLLSWAVNYKIDGFRF**D** 554

StPUL FSVLDKIVPGYFLRRNADGGIENSTCVNNTASEHFMVERLILDDLKCWAVHYKIDGFRFD 541

*****:****::**: :* ***:.:*:*****:**:** *** *.::**:******

OsPUL LMGHIMKSTMIRAKSAIRSLTRDVHGVYGSKIYLYGEGWDFGEVAQNKRGINASQINMSG 636

HvPUL LMGHIMKRTMVT-KSALQSLTTDAHGVDGSKIYLYGEGWDFAEVARNQRGINGSQLNMSG 552

ZmPUL LMGHIMKKTMIRAKSALQSLTIDEHGVDGSKIYLYGEGWNFGEVAENQRGINGSQLNMSG 610

SoPUL LMGHIMKHTMVKATNMLQGLSKNIDGVEGSSIYLYGEGWDFGEVANNARGVNASQLNLGG 611

AtPUL LMGHIMKATIVNAKSAIGSLRKETDGVDGSRIYLYGEGWNFGEVAENGRGINASQFNLGG 612

MePUL LMGHMMKSTMVKAKDALLSLTKERNGVDGSSIYLYG**E**GWDFGEVAKNARGINASQFNLGG 614

StPUL LMGHIMKRTMLKAKSLLNSLSTEENGVDGSSIYIYGEGWDFGEVAKNGRGINASQFNLFG 601

****:** *:: .. : .* : .** ** **:*****:*.***.* **:*.**:*: *

OsPUL TGIGSFNDRIRDSVNGGNPFGNPLQQGFSTGLFLEPNGYYQGNEADTRRELATYADHIQI 696

HvPUL TGIGSFNDRIRDAINGGNPFGNPLQQGFNTGLFLEPNGFYQGNEADTRRSLATYADQIQI 612

ZmPUL TGIGSFNDRIRDAINGGSPFGNPLQQGFSTGLFLEPNGFYQGNETETRLTLATYADHIQI 670

SoPUL TGIGSFNDRIRDAVLGGGPFGPPLQQGYVTGLSLQPNDHDHSGKANADRMLAVAKDHIQV 671

AtPUL TGIGSFNDRIRDATLGGSPFGHPLQQGFITGLLLQPNAHDHGSEATQELMLSTAKNHIQT 672

MePUL TGIGSFNDRIRDAMHGGSPFGHPLQQGFVTGLMLQPNGHDHGGKDVEEHMLTIAKDHIQA 674

StPUL AGIGSFNDRIRDALLGGSPFGHPLHQGFVTGLYLEPNGHDLGDKANVERMLTVSKDHIQV 661

:***********: **.*** **:**: *** *:** . ..: *: ::**

OsPUL GLAGNLKDYVLRTHTGEAKKGSDIYTFDGSPVGYTSSPVETINYVSAHDNETLFDIVSIK 756

HvPUL GLAGNLRDYVLISHTGEAKKGSEIHTFDGLPVGYTASPIETINYVSAHDNETLFDVISVK 672

ZmPUL GLAGNLKDYVVISHTGEARKGSEIRTFDGSPVGYASSPIETINYASAHDNETLFDIISLK 730

SoPUL GMAGNLRDYILTNCDGKQVKGSEVYTYGGTPVGYAMQPIETINYVSAHDNETLFDIVSLK 731

AtPUL GMAANLKDYMLTNHEGKEVKGSEVLMHDATPVAYASLPTETINYVSAHDNETLFDIISLK 732

MePUL GMAANLREFVLINSEGKEVKGSEILTYGGEPLAYALCPTETINYVSAH**D**NETLFDVVCMK 734

StPUL GMAANLKDFVLTNCDGQEVKGSEVLMYDGKPVGYASSPVETVNYVSAHDNETLFDIISLK 721

*:*.**::::: . *: ***:: ... *:.*: * **:**.**********::.:*

OsPUL TPIGLSIDGECRINHLASSMIALSQGIPFFHAGDEILRSKSLDRDSYNSGDWFKKLDLHM 816

HvPUL TPMILSVDERCRINHLASSMMALSQGIPFFHAGDEILRSKSIDRDSYNSGDWFNKLDFTY 732

ZmPUL TPMDLSIDERCRINHLSTSMIALSQGIPFFHAGDEILRSKSLDRDSYDSGDWFNKIDFTY 790

SoPUL TPTYITVDERCRVNHLATSILALSQGIPFFHAGDELLRSKSLDRDSYNSGDWFNRLDFSY 791

AtPUL TPMEISVDERCRINHLASSMIALSQGIPFFHAGDEILRSKSLDRDSYNSGDWFNRLDFSY 792

MePUL TPMQISVDERCRLNYLATNMIALAQGIPFFHAGDEMLRSKSLDRDSYNSGDWFNRLDFSY 794

StPUL TPKDISVEERCRMNHLATSVIALSQGIPFFHAGDEMLRSKSIDRDSYNSGDWFNRLDFSY 781

** :::: .**:*:*::.::**:***********:*****:*****:*****:::*:

OsPUL NQPI-GCRL-LQEIRMKNMHLIKPRLENPSFRPLKNHILSCFDNFVDILKIRYSSPLFRL 874

HvPUL ETNNWGVGLPPSEKNEDNWPLMKPRLENPSFKPAKGHILAALDSFVDILKIRYSSPLFRL 792

ZmPUL ETNNWGVGLPPREKNEGSWPLMKPRLENPSFKPAKHDIIAALDKFIDILKIRYSSPLFRL 850

SoPUL NSNNWGVGLPPKDHNESNWPLIKKRLANPSYKPDKNHIIAAVENFTNLLQIRYSSPLFRL 851

AtPUL SSNNWGVGLPPKGKNEHNWPLIKPRLQDPSFKPKSSHIVATLHNFLDLLRIRYSSPLFRL 852

MePUL NSNNWGVGLPPKGKNEGNWPLMKPRLADPSFKPQKSHILATVDNFLDVLQIRYSSPLFRL 854

StPUL NSNNWGVGIPPKEKNERNWPLIKPRLADPSYKPQKSHILAAVENFLNLMQIRYSSPLFRL 841

. * : . . *:* ** :**::* . .*:: ...* ::::**********

OsPUL STASDIEQRVRFHNTGPSMVPGVIVMSIKDAQNEKCKMAQLDKNFSYVVTIFNVCPHEVS 934

HvPUL STANDIKQRVRFHNTGPSLVPGVIVMGIEDARGESPEMAQLDTNFSYVVTVFNVCPHEVS 852

ZmPUL TTASDIVQRVHFHNTGPSLVPGVIVMSIEDARNDRHDMAQIDETFSCVVTVFNVCPYEVS 910

SoPUL RSAKDIEDRVRFHNNVPSWIPGLIAMSIEDGHAGAPGLSQIDPKFQYIVVIINVQPTETK 911

AtPUL DTARAIQERVRFHNTGPSSIPGAIVMSIEDGHRGIPSVSQIDPIYSLIVVIFNARPSEFS 912

MePUL TTANAIQERVRFHNTGPSWVPGVIVMSIEDGHEGFPGLSQLDPIYSYIVVIFNTCPNEIL 914

StPUL KTANAIQERVRFHNTGPSWIPGLIVMSIEDGHQGVPGLSQLDPIYSYIVVIINPCPTDVS 901

:* * :**:***. ** :** *.*.*:*.: ::*:* :. :*.::* * :

OsPUL IEIHDLASLGLELHPIQVNSSDALVRQSAYEASKGRFTVPRRTTAVFVQPRC- 986

HvPUL MDIPALASMGFELHPVQVNSSDTLVRKSAYEAATGRFTVPGRTVSVFVEPRC- 904

ZmPUL IEIPDLASLRLQLHPVQVNSSDALARQSAYDTATGRFTVPKRTAAVFVEPRC- 962

SoPUL FVNPDLRAKSLQLHPVQSTSGDTVVKESKYEPSTGCFTIPPKSTAVFVEPRHV 964

AtPUL YPSPALKDRKLELHPVQVMSADEIVKKSVYDSFSGGFTVPARTTTVFVESRNG 965

MePUL FACPPLRARGFQLHPVQVKSTDKVVKKSAYEPLSGCFTVPPMTTSVFVEPRKI 967

StPUL FANLALKAKSLQLHPVQMNSTD-VVKNSTYDASSGCFNVPARTTSVFVQPR-- 951

* ::***:* * * :.::* *: .* *.:* :.:***: *

**Figure S2. Multiple alignment of PULs from plants.** *Me*PULs from *Manihot esculenta* (this work), *Hordeum vulgare* (*Hv*LD; GenBank AAD04189.1), *Oryza sativa* (*Os*PUL; GenBank BAA09167.1), *Solanum tuberosum* (*St*PUL; GenBank XP_006361707.1), *Arabidopsis thaliana* (*At*PUL; GenBank NP_196056.2) *Zea mays* (*Zm*PUL; GenBank NP_001104920.1), and *Spinacia oleracea* (*So*PUL; GenBank CAA58803.1) were aligned by Clustal Omega software. The green colour presents a *Me*PUL transit peptide. The yellow represents the conserved sequences of GH13 while the catalytic triad is shown in red. The sequences highlighted by red, grey, and cyan presents the sequence of *N*-terminal domain, carbohydrate-binding module 48 (CMB48), and *C*-terminal domain, respectively. Meanwhile, the underline sequence indicates catalytic domain sequence of *Me*PUL based on the 3D structure of *Hv*PUL (Vester-Christensen et al., 2010)


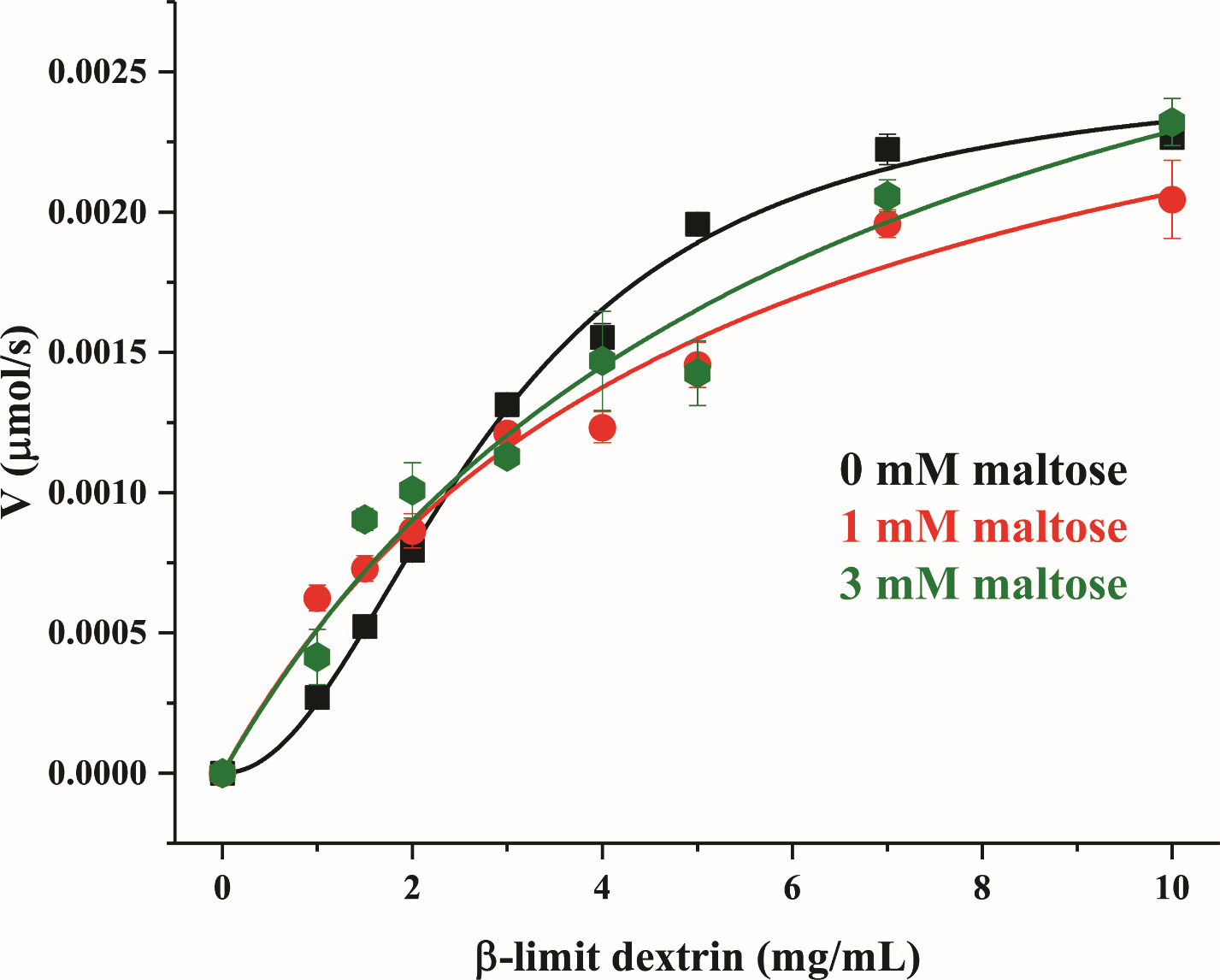


**Figure S3. Effect of maltose concentration on r*Me*PUL kinetics.** β-limit dextrin was used as substrate. The reactions were carried out in 25 mM acetate buffer pH 6.0 at 50 °C with the presence of 0, 1, and 3 mM maltose (shown in black, red, and green, respectively). The reactions were monitored by DNS assay.


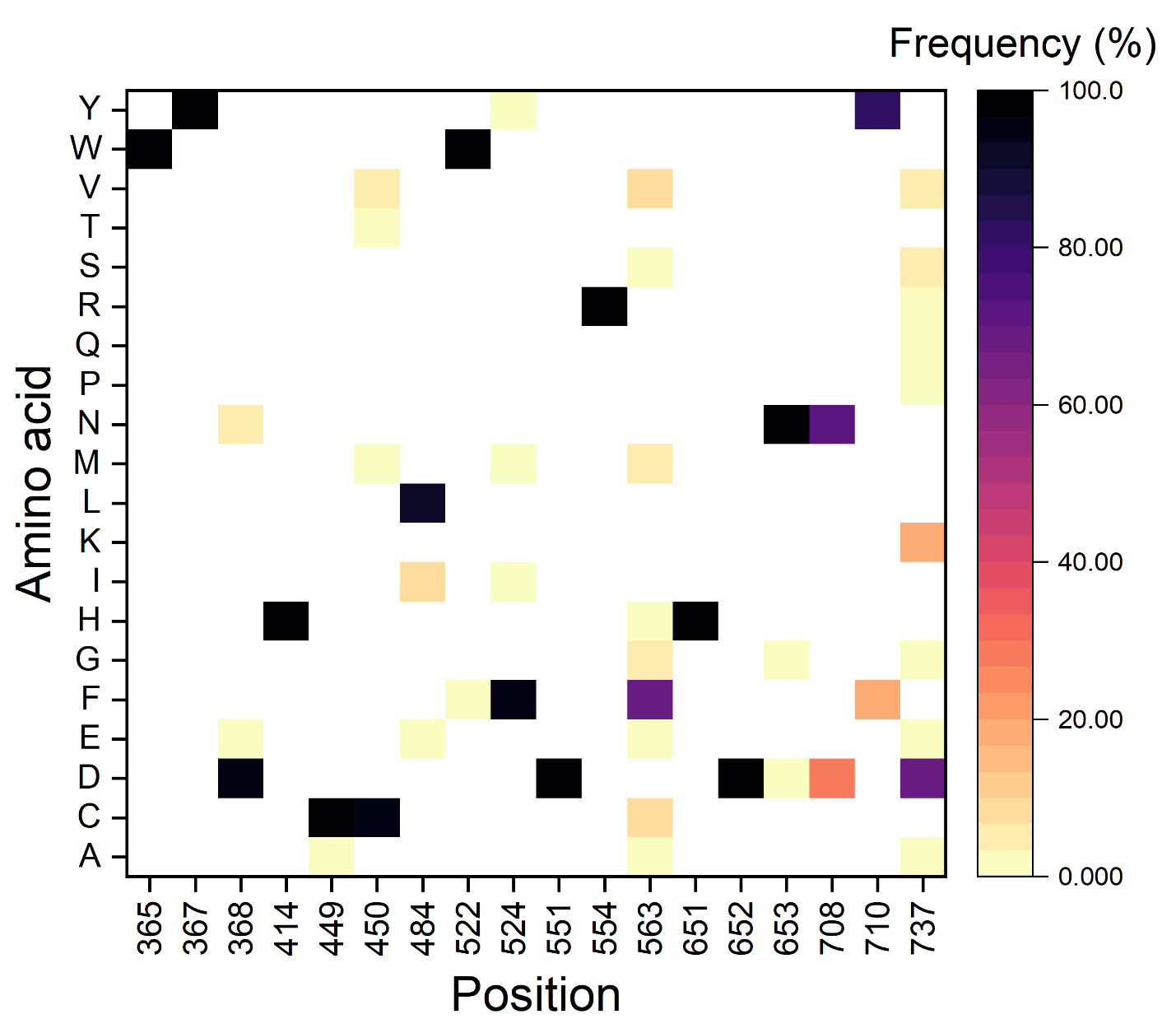


**Figure S4. Conserved binding residue analysis.** Proposed residues binding with branched maltohexaose were analysed by ConSurf server.


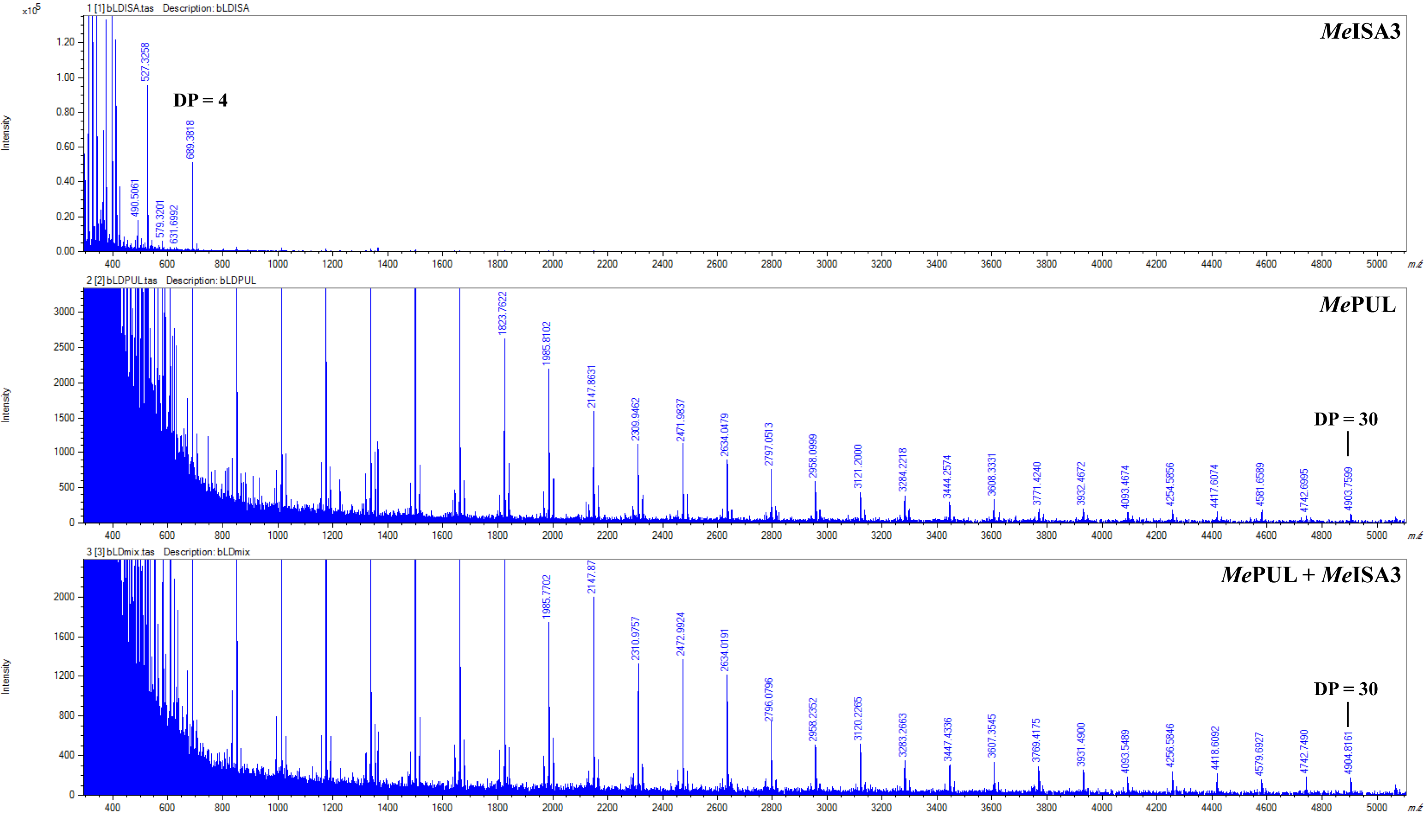


**Figure S5. Mass analysis of β-limit dextrin hydrolysis by *Me*ISA3, *Me*PUL, and mixed *Me*PUL + *Me*ISA3 reaction.** Individual *Me*ISA3 and *Me*PUL, as well as mixture of *Me*PUL + *Me*ISA3 were incubated with β-limit dextrin for 24 hr and then products were analysed by MALDI-TOF.


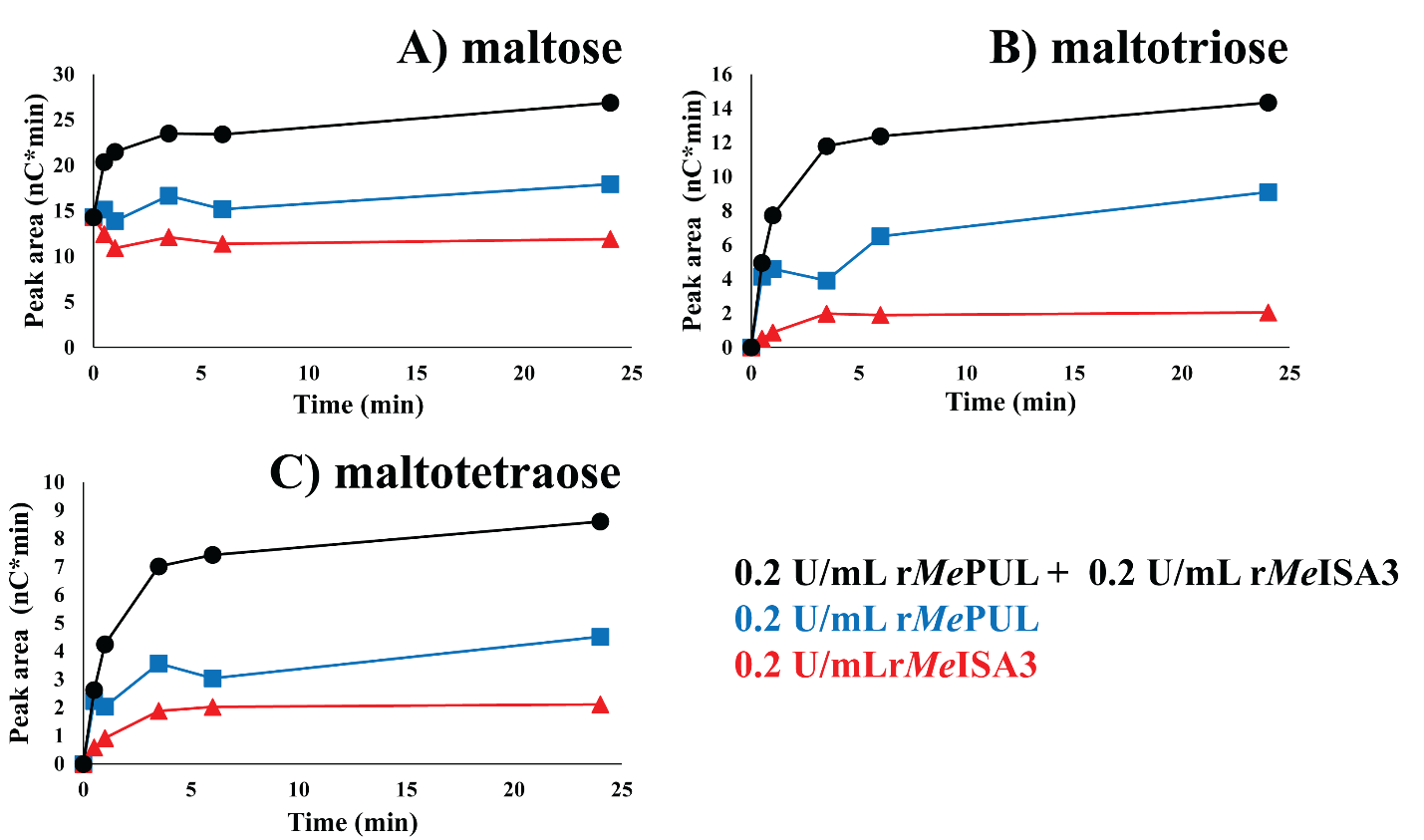


**Figure S6. Peak area between r*Me*PUL and r*Me*ISA3 on β-limit dextrin.** Single 0.2 U/mL r*Me*PUL (blue) or r*Me*ISA3 (red) as well as mixture (black) of 0.2 U/mL r*Me*PUL + 0.2 U/mL r*Me*ISA3 were incubated with 0.5 % (w/v) β-limit dextrin in 25 mM acetate buffer pH 6.0 at 37 ℃ for 24 hrs and products were analysed by HPAEC-PAD. The peak areas of maltose (A), maltotriose (B), and maltotetraose (C) were plotted and compared.

KpPUL CDNSSSSSTSGSPGSPGNPGNPGTPGTPDPQDVVVRLPDVAVPGEAVQASARQAVIHLVD 60

MePUL ------------------------------------------------------------ 0

HvPUL ------------------------------------------------------------ 0

KpPUL IAGITSSTPADYATKNLYLWNNETCDALSAPVADWNDVSTTPTGSDKYGPYWVIPLTKES 120

MePUL ------------------------------------------------------------ 0

HvPUL ------------------------------------------------------------ 0

KpPUL GCINVIVRDGTNKLIDSDLRVSFSDFTDRTVSVIAGNSA--VYDSRADAFRAAFGVALAD 178

MePUL --------------------------------------------GSTPTSELQDCLLYSR 16

HvPUL --------------------------------MAVGETGASVSAAEAEAEATQAFMPDAR 28

. : : : :

KpPUL AHWVDKTTLLWPGGENKPIVRLYYSH-SSKVAA-DSNGEFS--DKYVKL--TPTTVNQQV 232

MePUL AFWVTKSIIAWNVDVGTDGSCFLYASQNAALSI--TESGVQGHDVKFKLEQDDGGLPEKV 74

HvPUL AYWVTSDLIAWNVGELEAQSVCLYASRAAAMSLSPSNGGIQGYDSKVELQPESAGLPETV 88

*.** . : * . *: : :: ::. .. * .:* : : *

KpPUL SMRFPHLASYPAFKLPDDVNVDELLQGETVAIAAESDGILSSATQVQTAGVLDDTYAAAA 292

MePUL VAKFPHIRGYRAFKVPPFVDTISLLKCQLAVAAFNFDGKCSNATGLQLPGILDELFSYDG 134

HvPUL TQKFPFISSYRAFKVPSSVDVASLVKCQLVVASFGADGKHVDVTGLQLPGVLDDMFAYTG 148

:**.: .* ***:* *:. .*:: : .. : ** ..* :* *:**: :: .

KpPUL EALSYGAQLTDSGVTFRVWAPTAQQVELVIYSADKKVIASHPMTRDSASGAWSWQGGSDL 352

MePUL ---PLGAHFAKDAVSLYLWAPTAQAVRLCIYKDSFTSNPTETVQLMEVNGVWSTKGPKDW 191

HvPUL ---PLGAVFSEDSVSLHLWAPTAQGVSVCFFDGPA-GPALETVQLKESNGVWSVTGPREW 204

** ::...*:: :****** * : ::. . : . .*.** * :

KpPUL KGAFYRYAMTVYHPQSRKVEQYEVTDPYAHSLSTNSEYSQVVDLNDSALKPEGWDGLTMP 412

MePUL EGCYYVYEVSVYHPSTLHIEKYFANDPYARGLSSDGKRTLLVNLDSDTLKPERWDILADE 251

HvPUL ENRYYLYEVDVYHPTKAQVLKCLAGDPYARSLSANGARTWLVDINNETLKPASWDELADE 264

:. :* * : **** . :: : . ****:.**::. : :*:::..:*** ** *:

KpPUL HAQKTKADLAKMTIHESHIRDLSAWDQTVPAELRGKYLALTAQESNMVQHLKQLSASGVT 472

MePUL K--PTLLSFSDISIYELHIRDFSANDDTVHSDLRGGYLAFTVEDSAGVLHLKKLSSAGLT 309

HvPUL K--PKLDSFSDITIYELHIRDFSAHDGTVDSDSRGGFRAFAYQASAGMEHLRKLSDAGLT 322

: . .::.::*:* ****:** * ** :: ** : *:: : * : **::** :*:*

KpPUL HIELLPVFDLATVNEFSDKVADIQQPFSRLCEVNSAVKSSEFAGYCDSGSTVEEVLTQLK 532

MePUL HVHLLPAFQFAGVDDVRENWKSVD-----NSMLE-----------------------KL- 340

HvPUL HVHLLPSFHFAGVDDIKSNWKFVD-----ECELA-----------------------TF- 353

*:.*** *.:* *::. .: :: . : :

KpPUL QNDSKDNPQVQALNTLVAQTDSYNWGYDPFHYTVPEGSYATDPEGTARIKEFRTMIQAIK 592

MePUL ---PPDSAEQQAHITAFQNDDGYNWGYNPVLWGVPKGSYASNPNGSCRTIEFRKMVQAIN 397

HvPUL ---PPGSDMQQAAVVAIQEEDPYNWGYNPVLWGVPKGSYASDPDGPSRIIEYRQMVQALN 410

.. ** . . : * *****:*. : **:****::*:* .* *:* *:**::

KpPUL QDLGMNVIMDVVYNHTNAAGPTDRTSVLDKIVPWYYQRLNETTGSVESATCCSDSAPEHR 652

MePUL -RIGLRVVLDVVYNHLHGSGPFDENSVLDKVVPGYYLRRN-SDGFIENSTCVNNTASEHY 455

HvPUL -RIGLRVVMDVVYNHLDSSGPCGISSVLDKIVPGYYVRRD-TNGQIENSAAMNNTASEHF 468

:*:.*::****** ..:** . .*****:** ** * : : * :*.::. .::* **

KpPUL MFAKLIADSLAVWTTDYKIDGFRFDLMLYHPKAQILSAWERI--------KALNPDIYFF 704

MePUL MVERLIVDDLLSWAVNYKIDGFRF**D**LMGHMMKSTMVKAKDALLSLTKERNGVDGSSIYLY 515

HvPUL MVDRLIVDDLLNWAVNYKVDGFRFDLMGHIMKRTMVT-KSALQSLTTDAHGVDGSKIYLY 527

*. :**.*.* *:.:**:******** : * ::. . : . . .**::

KpPUL GEGWDSNQSD---RFEIASQINLKGTGIGTFSDRLRDAVRGGGPFDSGDALRQNQGVGSG 761

MePUL G**E**GWDFGEVAKNARGINASQFNLGGTGIGSFNDRIRDAMHGGSPFGHPL----QQGFVTG 571

HvPUL GEGWDFAEVARNQRGINGSQLNMSGTGIGSFNDRIRDAINGGNPFGNPL----QQGFNTG 583

***** : * .**:*: *****:*.**:***:.**.**. :**. :*

KpPUL AGVLPNELTTLSDDQA----RHLADLTRLGMAGNLADFVLIDKDGAVKRGSEI-DYNGAP 816

MePUL LMLQPNGHDHGGKDVEEHMLTIAKDHIQAGMAANLREFVLINSEGKEVKGSEILTYGGEP 631

HvPUL LFLEPNGFYQGNEADTRRSLATYADQIQIGLAGNLRDYVLISHTGEAKKGSEIHTFDGLP 643

: ** .. * : *:*.** ::***. * :**** :.* *

KpPUL GGYAADPTEVVNYVSKHDNQTLWDMISYKAAQEADLDTRVRMQAVSLATVMLGQGIAFDQ 876

MePUL LAYALCPTETINYVSAHDNETLFDVVCMKTPMQISVDERCRLNYLATNMIALAQGIPFFH 691

HvPUL VGYTASPIETINYVSAHDNETLFDVISVKTPMILSVDERCRINHLASSMMALSQGIPFFH 703

.*: * *.:**** ***:**:*::. *: .:* * *:: :: : *.*** * :

KpPUL QGSELLRSKSFTRDSYDSGDWFNRVDYSLQDNNYNVGMPRSSDDGSNYDIIA-RVKDAVA 935

MePUL AGDEMLRSKSLDRDSYNSGDWFNRLDFSYNSNNWGVGLPPKGKNEGNWPLMKPRLADPSF 751

HvPUL AGDEILRSKSIDRDSYNSGDWFNKLDFTYETNNWGVGLPPSEKNEDNWPLMKPRLENPSF 763

*.*:*****: ****:******::*:: : **:.**:* . .: .*: :: *: :

KpPUL TPGETELKQMTAFYQELTALRKSSPLFTLGDGATVMKRVDFRNTGADQQTGLLVMTIDDG 995

MePUL KPQKSHILATVDNFLDVLQIRYSSPLFRLTTANAIQERVRFHNTGPSWVPGVIVMSIEDG 811

HvPUL KPAKGHILAALDSFVDILKIRYSSPLFRLSTANDIKQRVRFHNTGPSLVPGVIVMGIEDA 823

.* : .: : :: :* ***** * . : :** *:*** . *::** *:*.

KpPUL MQAGA---SLDSRVDGIVVAINAAPESRTL--QDFAGTSLQLSAIQQAAGDRSLASGVQV 1050

MePUL HEGFPGLSQLDPIYSYIVVIFNTCPNEILFACPPLRARGFQLHPVQVKSTDKVVKKSAYE 871

HvPUL RGESPEMAQLDTNFSYVVTVFNVCPHEVSMDIPALASMGFELHPVQVNSSDTLVRKSAYE 883

.** . :*. :*..*.. : : . .::* :* : * : ...

KpPUL AADGSVTLPAWSVAVLELPQGESQGAGLPVSSK 1083

MePUL PLSGCFTVPPMTTSVFVEPRKI----------- 893

HvPUL AATGRFTVPGRTVSVFVEPRC------------ 904

* .*:* :.:*: *:

**Figure S7. Protein sequence alignment of *Kp*PUL, *Me*PUL, and *Hv*PUL.** The sequences of *Me*PUL, *Kp*PUL [PDB 2FGZ], and *Hv*PUL [GenBank AAD04189.1] were aligned using Clustal Omegar software.

MePUL MSIVVSKSLPSVSPPSIPNIHFSTFSSPSSSRSRLILRQYQCHHLPFPLCFRTFHRCPIR 60

MeISA2 ----MATLLP----------------------------------------SFAISRCCYS 16

MeISA1 ------------------------------------------------------------ 0

MeISA3 ------------------------------------------------------------ 0

MePUL CCSSSSS-MALHE--VSGST-----PTSELQDCLLYSRAFWVTKSIIAWNVDVG------ 106

MeISA2 CGAVESSKLTLTTRYTSGKKMELGFGRTDVEKRLLVGEVAQNVRSTLHWNHNSGVFAAAR 76

MeISA1 ------------------------------------------------------------ 0

MeISA3 ------------------------------------------------------------ 0

MePUL ------------------------------------------------------------ 106

MeISA2 VPVQETEQILSTITEVDELQKVSSYLFRTQIGGNVKVSVRKKNAKYAVYIEVSSLELGNS 136

MeISA1 -----------------------MDLI------------------------QSS--A--- 8

MeISA3 --------MLRPLLPCDSTSAAKMRLFASPFSNNYTIA-----------IVPSS--AGH- 38

MePUL ------------TDGSCFLYASQNAALSITESGVQGHDVKFKLEQDDGGL-PEKVVAKFP 153

MeISA2 DYRLVLAWGIYRSDSSCFMPLDSQRL-----------------DPVARTMETPFVQNAFA 179

MeISA1 ---------------SRFLSLPF-HI-----------------PPR------NKDAAEFL 29

MeISA3 ---------------SHVLDMGL-KL-----------------SKQ------ASSSSGLR 59

* .: :

MePUL HIRGYRAFKVPPFVDTISLLKCQLAVAAFNFDGKCSNATGLQ----------LPGILDEL 203

MeISA2 IFSLELEFEAKQTPFSLSFLLK----SMFN---T--DSSGSEIRNH--KKANFSVPIGFS 228

MeISA1 IFRQRKCFSHSQSLSTV--RTS----PLIE---ATRRDGGSELETAVVVDKPRLGKYQVS 80

MeISA3 IFSQGRD--------KH--RTP----SVYG---RGARERVLE-EKEASLMSETSPSFKIF 101

: . :

MePUL FSYDGPLGAHF-AKDAVSLYLWAPTAQAVRLCIYKDSFTSNPTE-----TVQLM----EV 253

MeISA2 SGYPDPLGLSFSTDGSMNFAFFSRNAEGVVLCLYDDSTTD-----KPALELDLDPYVNRS 283

MeISA1 EGHPAPFGATV-RDGGVNFSVYSANAVSASLCLISLDDLAENRV---TEEIPLDPLANKT 136

MeISA3 PGQASPLGVSE-VDKGINFAIFSQHATSVTLCLSLPQRGVHERLVGNVIELALDPHVNKT 160

. *:* . .:.: .:: * .. **: . : * .

MePUL NGVWSTKGPKDW-EGCYYVYEVSV---YHPSTLHIEKYFANDPYARGLSSDGKRTLLV-- 307

MeISA2 GDVWHASLEGAC-TFSSYGYRCMGGILQGETGKDYVERVLLDPYARIIVNFTADH--GS- 339

MeISA1 GYIWHVFLKGEF-RDVLYGYRFDGKFSRGEGHYFDSSKIVLDPYAKAVISRGEFGVLGPD 195

MeISA3 GDIWHICIEDLPRSNVLYGYRVDGPQNWNQGHRFDSNIILVDPYAKLVEGRRYFGDASLK 220

. :* * *. . . ****: : .

MePUL ------NLDSDTLKPERWDILADEKPTLLSFSDISIYELHIRDFSANDDT-VHSDLRGGY 360

MeISA2 -HSSLKYLGRL-CKEPAFEWSDEV-YPNLDMEKLVVYRLNVKRFTEHKSSQLYSDIAGTF 396

MeISA1 DNCWPQMAGMIPAAQDKFDWQGDLPLR-YSQKDLVIYEMHVRGFTRHESSR--TEFPGTY 252

MeISA3 ---LSKFLGTYDFDSSLFDWGDNYKLPNIPEKDLVIYEMNVRAFTADKSSGLDPKIRGSY 277

. :: : ..: :*.:::: *: ...: .: * :

MePUL LAFTVEDSAGVLHLKKLSSAGLTHVHLLPAFQFAGVDDVRENWKSVDNSMLEKLPPDSAE 420

MeISA2 AGLTE-------KLNHIKNLGVNAVLLEPIFPFDEEKGP--------------------- 428

MeISA1 LGIVE-------KLDHLKELGVNCIELMPCHEFNELEYFSYNS----------------- 288

MeISA3 LGVIE-------KIPHLLELGVNAVELLPVFEFDEFEFQR-RP----------------- 312

.. :: :: . *:. : * * . * .

MePUL QQAHITAFQNDDGYNWGYNPVLWGVPKGSYASNPNGSCRT---IEFRKMVQAINRIGLRV 477

MeISA2 -----------------FFPRHFFSPSNIYGPSGGSI---SAITSMKEMVKQFHANGIEV 468

MeISA1 ------VLGDHRMNFWGYSTVNYFSPMTRYSSAGTRNCGRDAINEFKFLVREAHKRGIEV 342

MeISA3 ------NPRDHMINTWGYSTINFFAPMSRYASGGGGPCNA--SREFKEMVKALHGAGIEV 364

: : * *. .:: :*: : *:.*

MePUL VLDVVYNHLHGSGPFDEN---SVLDKVVPGYYLRRNSDGFIENSTCVNNTASEHYMVERL 534

MeISA2 LLEVVFTHTAEGG-------SLQGIDDFSYYYANRAV-----ELESRNALNCNYPIVQRM 516

MeISA1 IMDVVFNHTAEGNEKGPI-LSFRGIDNSVYYMLAPK-GEFYNYSGCGNTFNCNHPVVRQL 400

MeISA3 ILDVVYNHTNEADDQNPYTTSFRGIDNKVYYMLDPNSGQLLNFSGCGNTLNCNHPVVMEL 424

:::**:.* .. . * . * .:: :* .:

MePUL IVDDLLSWAVNYKIDGFRF**D**LMGHMMKSTMVKAKDALLSLTKE----------------- 577

MeISA2 ILDSLRHWVTEYHIDGFCF**I**NASFLQRGF-------------------HGEILSRPPLVE 557

MeISA1 ILDCLRYWVIEMHVDGFRF**D**LASIMTRGSSLWDPVNVFGKPIEGDLLTTGSPLGSPPLID 460

MeISA3 ILDSLRHWVTEYHVDGFRF**D**LASVLCRGT-------------------DGTPLSAPPVIR 465

*:* * *. : ::*** * . : :.

MePUL --RNGVDGSSIYLYG**E**GWDFGEVAKNARGINASQFNLGGTGIGSFNDRIRDAMHGGSPFG 635

MeISA2 AIAFDPLLSKTKIIA**D**CWDPEDVIPKDTCFP--HWKRWAEMNAKFCFDVRNFLRGESL-L 614

MeISA1 MISNDPILREVKLIA**E**AWDAGGLYQ-VGTFP--HWQIWSEWNGKFRDIVRQFIKGTDGFA 517

MeISA3 AIAKEPILSRCKIIS**E**PWDCGGLYL-VGKFP--NWDRWAEWNGKYRDDMRRFIKGDSGMK 522

: .: ** : : ::. . ..: :* ::* .

MePUL HPLQQGFVTGLMLQPNGHDHGGKDVEEHMLTIAKDHIQAGMAANLREFVLINSEGKEVKG 695

MeISA2 SDLATR---------------------------------------------------LCG 623

MeISA1 GAFAEC---------------------------------------------------LCG 526

MeISA3 GSFATR---------------------------------------------------VAG 531

: : *

MePUL SE-ILTYGGEPLAYALCPTETINYVSAH**D**NETLFDVVCMKTPMQIS-------------- 740

MeISA2 SGDIFSS-------GRGPAFSFNYVARN**S**GLPLVDLVSFSSSE-----------LASELS 665

MeISA1 SPNLYQE------GGRKPWNSINFVCAH**D**GFTLADLVTYNNKNNLANGEDNNDGENHNNS 580

MeISA3 SADLYSA------NKRKPCHSINFVIAH**D**GFTLYDLVSYNFKHNDANGEGGNDGSNDNFS 585

* : * ::*:* :.. * *:* .

MePUL ----------------VDERCRLNYLATNMIALAQGIPFFHAGDEMLRSKSLDRDSYNSG 784

MeISA2 WNCGEEGATNKTPVLERRLKQIRNYLF--ILYVSLGVPVLNMGDECGQSSNGST-SYGDR 722

MeISA1 WNCGQEGEFASILVKKLRKRQMRNFFV--CLMVSQGIPMIYMGDEYGHTKGGNNNTYCHD 638

MeISA3 WNCGFEGETDDPSIKALRSRQMKNFHL--ALMISQGTPMMLMGDEYGHTRYGNNNSYGHD 643

: *: : :: * *.: *** :: . :*

MePUL DWFNRLDFSYNSNNWGVGLPPKGKNEGNWPLMKPRLADPSFKPQKSHILATVDNFLDVLQ 844

MeISA2 ---KPFDWNALSMGFGIQMTRFISF------------MSSLRRRRSD-VLQKRNFM---- 762

MeISA1 NYINYFRWDKKEESSS-DFYRFCCQ------------MTKF-RHECK-SLGLNDFP---- 679

MeISA3 TSINNFQWGFLDKQRS-SHFRFFSE------------VIKF-RLMHQ-VFRHENFL---- 684

: : :. . . .: . :*

MePUL IRYSSPLFRLTTANAIQERVRFHNTGP---SWVPG---VIVMSIEDGHEGFPGLSQLDPI 898

MeISA2 ---------------KEENIDWHGSGQSPPRWEDRSCKFLAMTLKTEKTENKLSPESSNI 807

MeISA1 ---------------TAERLQWHGHYPCVPDWS-ETSRFVAFTQI------------DSA 711

MeISA3 ---------------SNNEVTWHED-----NWDNYESKFLAFTLH------------DSI 712

:.: :* * .:.:: .

MePUL YSYIVVIFNTCPNEILFACPPLRARGFQLHPVQVK---STDKVVKKS--AYEPLS---GC 950

MeISA2 KGDLFMAFNAYPHSESVILPPVPE-GMTWHRLVDTSLPFPGFFSEDGEPVFEQMAGL--- 863

MeISA1 KGEIYVAFNANHLPVTVTLPERPG--YRWEPLVDTGKPAPYDFLYSDVPERDAAVKQYGH 769

MeISA3 GADIYLAFNAHNYYVKVSIPPPPS-KRRWFRVADTNLASPDDFVPEGVPGIENS------ 765

. : : **: . * : . . .. :

MePUL ------FTVPPMTTSVFV-EPRKI- 967

MeISA2 ----IAYEMKSHSCTLFEATSLGG- 883

MeISA1 FLDANFYPMHSYSSIILILSPDEIA 794

MeISA3 ------YNVAPYSSILLEAKLT--- 781

: : : ::

**Figure S8. Multiple sequence alignment of debranching enzymes from *Manihot esculenta* Crantz ‘KU50’.** The sequences of *Me*PUL, *Me*ISA1 [GenBank AUZ20772.1], *Me*ISA2 [GenBank AUZ20773.1], and *Me*ISA3 [GenBank AUZ20774.1] were aligned by using Clustal Omega software. The yellow shows 4 conserved motifs of GH13, while the catalytic triad is shown in red.


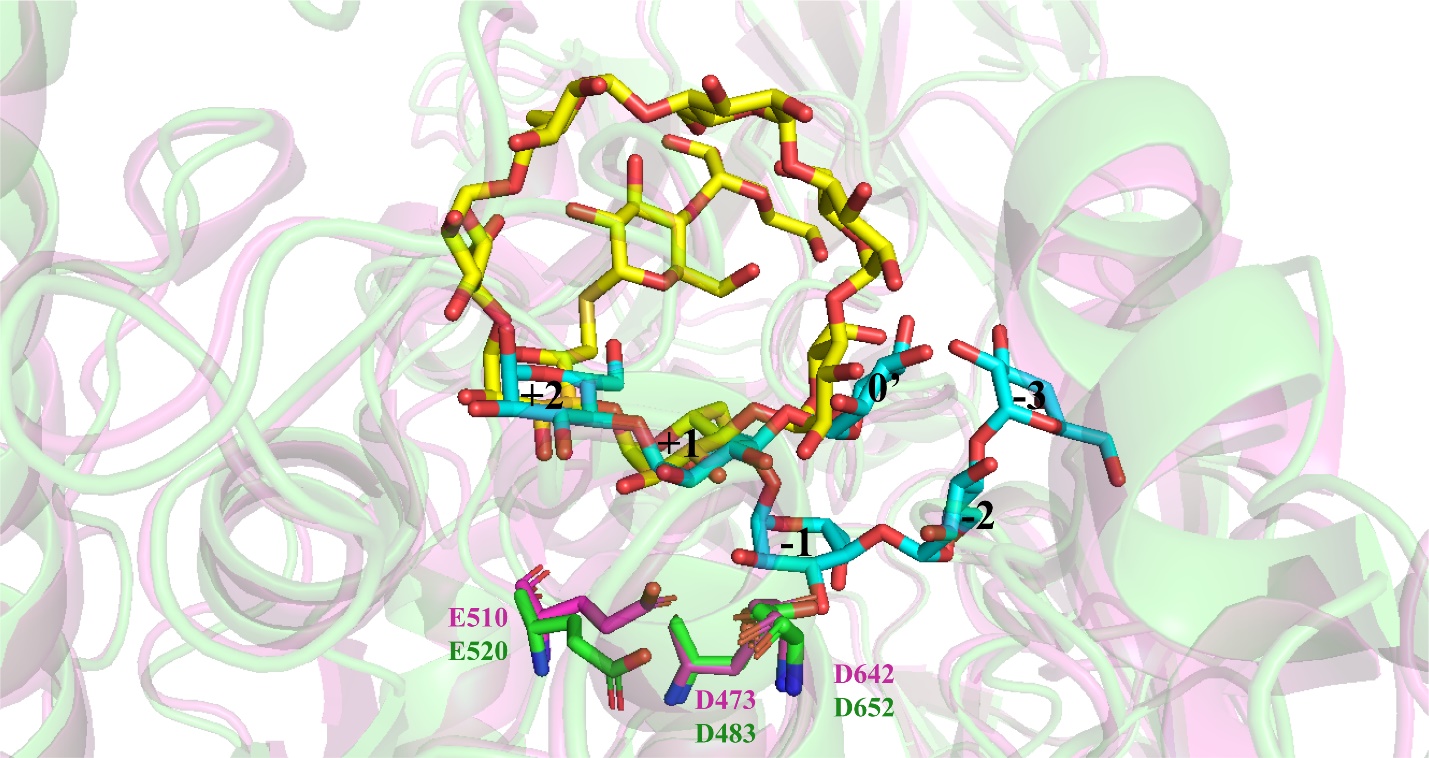


**Figure S9. Superimposition of *Me*PUL complexed with branched hexasaccharide and *Hv*LD complexed with 6-S-(α-D-maltosyl)-6-deoxy-6-thiocyclomaltoheptaose (PDB: 4J3U).** The green and magenta colours present 3D structures of *Me*PUL and *Hv*LD. Branched hexasaccharide and 6-S-(α-D-maltosyl)-6-deoxy-6-thiocyclomaltoheptaose (G2SβCD) are in cyan and yellow.


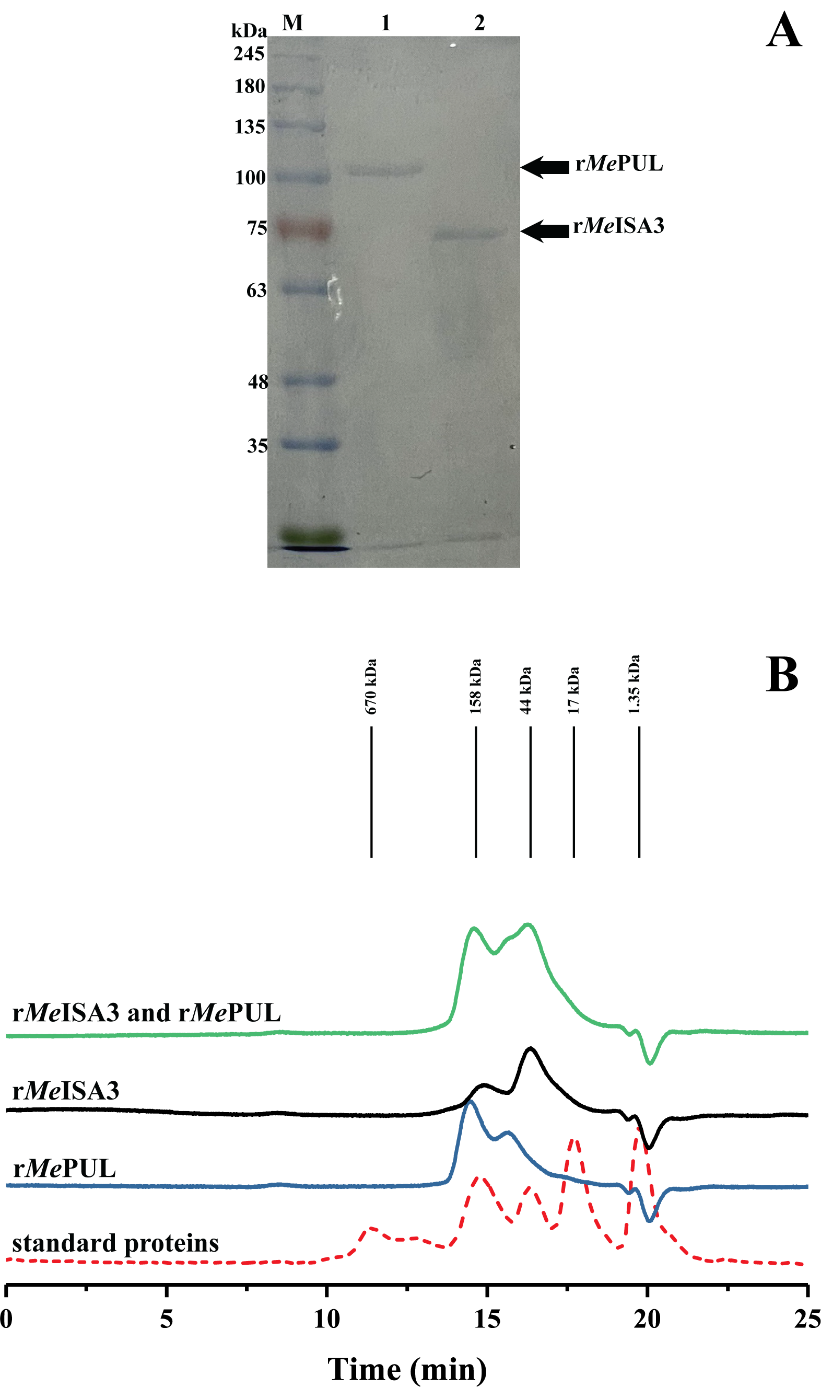


**Figure S10. Heterocomplex analysis between r*Me*ISA3 and r*Me*PUL. A)** SDS-PAGE analysis of r*Me*ISA3 and r*Me*PUL. **B)** Gel filtration chromatography of r*Me*ISA3 and r*Me*PUL. ~ 0.26 nmol of r*Me*PUL and r*Me*ISA3 was mixed in 25 mM phosphate buffer pH 7.2 in 0.1-mL total volume at 4 ℃ overnight before analysis with gel filtration chromatography.
