# Supplementary material for: Cassava pullulanase and its synergistic debranching action with isoamylase 3 in starch catabolism
Source: Front Plant Sci. 2023 Jan 27;14:1114215. doi: 10.3389/fpls.2023.1114215 (PMC9911869; doi:10.3389/fpls.2023.1114215)
Supplement: Supplementary file 2 [file DataSheet_2.docx]

**Supplementary data II**

**Sequences searching and comparison by ConSurf server**

**>Input_protein_seq (*Me*PUL)**

MASGSTPTSELQDCLLYSRAFWVTKSIIAWN-VDV----------GTD-GSCFLYASQNA

A-LSITES---GVQ---GH-D-----VKFKLEQD-DGG--LPEKVVAKFPHIR--GYRAF

KVPP--------FVDTI-----SLLKCQLAVAAFN-----F----------DGK-CSNAT

GLQLPGILDELFS------Y---DGPLGA-------HF----A-K----------D----

-----------AVSLYLWAPTA-QAVRLCIYKDSFT------------SNPT--ETVQLM

EV---NGVWST------KG-PK-D-----WE--GCYYVYEVSVYHP-----STLHIEKYF

ANDPYARGLSSD-----GKRTLLVNL---DSDTLKPERWDILADEKP-TL-LSFSDISIY

ELHIRDFSANDDTVHSDLRGGYLAFTVED---------SAGVLHLKKLSSAGLTHVHLLP

AFQFAG--VDDVRE---------------NWKSVDN------------------------

--SMLEKLP---PDSAEQQAHITAFQNDDGYNWGYNPVLWGVPKGSYASNP---NGSCRT

IEFRKMVQAINRIGLRVVLDVVYNHLHGSGPFDENSVLDKVVPGYYLRRN-SDGFIENST

C--VNNTASEHYMVERLIVDDLLSWAVNYKIDGFRFDLMGHMMKSTMVKAKDALLSLTKE

RNGVDGSSIYLYGEGWDFG-EVAKNARGI----NASQFNLG-GTGIGSFNDRIRDAMHGG

SPF-GHP----LQQGFVTGLMLQPNGHD---------HGGKD-VEEHMLTIAKDHIQAGM

AANLREFVLIN-S-EGK-EVKGSEILTY-GG-EPLAYALCPTETINYVSAHDNETLFDVV

CMKT--------P---------------MQISVDERCRLNYLATNMIALAQGIPFFHAGD

EMLRSKSLDRDSYNSGDWFNRLDFSY---------N-SNNWG-VGLPPKGKN----EGNW

PLMKPRLA-D-PS-FK--PQKSHILATVDNFLDVLQIRYSSPLFRLTTANAIQERVRFHN

T--G-PS--WVPGVIVMSIEDGHEGF-PGLS-QLDPIYSYIVVIFNTCPNEILFACPPLR

--------ARGFQLHPVQVK--S-T--DKV-VKKSAYEPLS--GCFTVPPMTTSVFVEPR

KIAAALE

>UniRef90_J3LVK7_17_897 | Uncharacterized protein n=3 Tax=Oryza brachyantha TaxID=4533 RepID=J3LVK7_ORYBR | E_val=0

-----------QEFLSDARAYWVTRSLIAWN-VN-----------DKE-TSLYLYASRDA

R-MHVSDG---VIE---GY-D-----EKIELQSE-HAS--LPASEAQKFPFIS--SYRIF

RVPS--------SVDVA-----SLVKCQLAIASFD-----A----------HGR-RQDIT

GLQLPGVLDDMFA------Y---TGPLGA-------IF----S-E----------E----

-----------DVSLYLWAPTA-QDVSVCFYDGPSG--------------PLL-QTVQLK

EL---NGVWSV------TV-PR-N-----WE--NQYYLYEVKIYHP-----STSQVEKCL

AADPYARGLSAN-----GTRTWLVDI---NSGTLKPASWDELSDEKP-NL-DSFSDISIY

ELHIRDFSAHDCTVDCNSRGGFRAFTCQD---------SAGIRHLRKLAVAGLTHVHLLP

SFHFAS--VDDNKS---------------NWKSVDE------------------------

--AELAKLP---PGSDEQQAAIVSIQQEDPYNWGYDPVLWGVPKGSYASNP---DGPSRI

IEYREMVQALNRIGLRVVMDVVYNHLDSSGPFGVSSVLDKIVPGYYLRRD-INGQIENSA

A--MNNTASEHFMVDRLIVDDLLNWAINYKVDGFRFDLMGHIMRSTMIRAKSALQSLRRD

VHGVDGSKIYLYGEGWDFG-EVAQNKRGI----NASQINMS-GTGIGSFNDRIRDAVNGG

NPF-GNP----LQQGFSTGLFLEPNGYY---------QGNEA-DTRRALATYADHIQIGL

AGNLKDYVLTT-H-TGQ-AKKGSEIHTF-DG-SPVGYTSSPVETINYISAHDNETLFDIV

SLKT--------P---------------VGLSIDERCRINHLASSMVALSQGIPFFHAGD

EILRSKSLDRDSYNSGDWFNKLDFTY---------E-TNNWG-VGLPPRDKN----EENW

HLIKPRLE-N-PS-FR--PLKNHILSVLDNFIDILKIRYSSPLFRLSTASDIEQRVRFHN

T--G-PS--LLPGVIVMSIEDARTVR-PEIA-QLDKNFSYVVTIFNVCPYEVSIEIHDLA

--------SLGLELHPVQVN--S-S--DAL-VRQSAYQAST--GRFTVPRRTTAVFVQPR

-------

>UniRef90_A0A7N0RGW6_72_954 | Uncharacterized protein n=1 Tax=Kalanchoe fedtschenkoi TaxID=63787 RepID=A0A7N0RGW6_KALFE | E_val=0

-------LSDLKDCLSYSRAYWVTESIIAWN-VDI----------G-D-GLCYLYASKNA

N-LLLSEN---EIT---GQ-D-----VKVRLIED-QGQ--LPGDVIEKFPQVR--GYRAF

VVPP--------AQDIK-----SLLKCQLAVAWFN-----S----------DGI-CRGAT

CLQLPGVLDDLFS------Y---NGPLGA-------IF----T-Q----------E----

-----------AVSLYLWAPTA-QGVRAFIYTEPSG------------GDPV--DVVQLE

EI---AGVWRI------RG-PR-N-----WE--GCYYVYEVSVYHY-----STMQIDKCY

ANDPYARGISTD-----SRRTLLVNL---DSDLLKPDGWDELASEKP-PL-NSFSDISIY

ELHVRDFSATDKTVQPDFQGCYLAFTSQD---------SSGVNHLRRLSSAGLTHIHLLP

TFQFAE--VDDDKE---------------HWMSVDF------------------------

--DMLERLP---PDSDEQQAHINTIKDMDGYNWGYNPVLWGVPKGSYASNP---SGPSRT

SEFRRMVQALNRMGLRVVLDVVYNHLHGNGPFDANSVLDKIVPGYYLRRN-SDGHIENST

C--VNNTASEHFMVERMILDDLLSWAIQYKVDGFRFDLMGHIMKRTMLKAKSALHNLSRE

RDGVDGSSIFLYGEGWDFG-EVANNGRGI----NASQFNLS-GSGIGSFNDRIRDAMLGG

SPF-GHP----LQQGFVTGLSLQPNDYN---------HGDKD-AVEYMLAASMDHIQIGL

AANLKDYVLTN-H-EGK-KVKGSEIYSY-GG-VPVAYAQGPSETINYVSAHDNETLFDIV

SLKT--------P---------------IQISVQERCRNNHLATSLIALSQGIPFFHAGD

DMLRSKSIDRDSYNSGDWFNRLDFSY---------N-HNNWG-VGLPPKEKN----EKNW

PLIKPRLT-N-PS-FK--PQKVHILAAVENFLSLLQIRYSSPLFRLQTANMIQERVRFLN

S--G-PS--SVPGVIVMSIDDG---L-PGLS-QLDQNYSHIVVIFNASPTTISFAAKALR

--------NMAFKLHPVQLT--S-R--DEV-VKTSYYEASS--GCFNVPAKTTSVFVEPR

-------

>UniRef90_O81638_79_961 | Pullulanase-type starch debranching enzyme n=15 Tax=Andropogoneae TaxID=147429 RepID=O81638_MAIZE | E_val=0

---------SAQGFLLDARAYWVTKSLIAWN-IS-----------DQK-TSLFLYASRNA

T-MCMSSQ---DMK---GY-D-----SKVELQPE-NDG--LPSSVTQKFPFIS--SYRAF

RIPS--------SVDVA-----TLVKCQLAVASFD-----A----------HGN-RQDVT

GLQLPGVLDDMFA------Y---TGPLGT-------IF----S-E----------E----

-----------AVSMYLWAPTA-QDVSVSFYDGPAG--------------PLL-ETVQLN

EL---NGVWSV------TG-PR-N-----WE--NRYYLYEVTVYHQ-----TTGNIEKCL

AADPYARGLSAN-----STRTWLVDI---NNETLKPLAWDGLAAEKP-RL-DSFSDISIY

ELHIRDFSAHDSTVDCPFRGGFCAFTFQD---------SVGIEHLKKLSDAGLTHVHLLP

SFQFGG--VDDIKS---------------NWKCVDE------------------------

--IELSKLP---PGSDLQQAAIVAIQEEDPYNWGYNPVVWGVPKGSYASNP---DGPSRI

IEYRLMVQALNRLGLRVVMDVVYNHLYSSGPFAITSVLDKIVPGYYLRRD-SNGQTENSA

A--VNNTASEHFMVDRLIVDDLLNWAVNYKVDGFRFDLMGHIMKKTMIRAKSALQSLTID

EHGVDGSKIYLYGEGWNFG-EVAENQRGI----NGSQLNMS-GTGIGSFNDRIRDAINGG

SPF-GNP----LQQGFSTGLFLEPNGFY---------QGNET-ETRLTLATYADHIQIGL

AGNLKDYVVIS-H-TGE-ARKGSEIRTF-DG-SPVGYASSPIETINYASAHDNETLFDII

SLKT--------P---------------MDLSIDERCRINHLSTSMIALSQGIPFFHAGD

EILRSKSLDRDSYDSGDWFNKIDFTY---------E-TNNWG-VGLPPREKN----EGSW

PLMKPRLE-N-PS-FK--PAKHDIIAALDKFIDILKIRYSSPLFRLTTASDIVQRVHFHN

T--G-PS--LVPGVIVMSIEDARNDR-HDMA-QIDETFSCVVTVFNVCPYEVSIEIPDLA

--------SLRLQLHPVQVN--S-S--DAL-ARQSAYDTAT--GRFTVPKRTAAVFVEPR

-------

>UniRef90_A0A0E0ICV6_85_960 | Uncharacterized protein n=11 Tax=Oryzinae TaxID=1648021 RepID=A0A0E0ICV6_ORYNI | E_val=0

----------------DARAYWVTRSLIAWN-VN-----------DQD-TSLFLYASRDA

T-MHVSDG---AIH---GY-D-----SKIELEPE-HAS--LPDNVAEKFPFIR--SYRTF

RVPS--------SVDVA-----SLVKCQLAVASYD-----A----------HGR-HQDVT

GLQLPGVLDDMFA------Y---TGPLGA-------VF----S-D----------K----

-----------DVDLYLWAPTA-QDVRVCFYDGPAG--------------PLL-QTVQLK

EL---NGVWSV------TV-PR-Y-----RE--NQYYLYEVKVYHP-----STSQVEKCL

ADDPYARGLSAN-----GTRTWLVDI---NSETLKPASWDELSDEKP-NL-ESFSDISIY

ELHIRDFSAHDSTVDCNSRGGFRAFTFQD---------SAGIRHLRKLSAAGLTHVHLLP

SFHFAS--VDDNKS---------------NWKFVDE------------------------

--AQLAKLP---PGSDEQQAAIVSIQQEDPYNWGYDPVLWGVPKGSYASNP---DGPSRI

IEYRQMVQALNRIGLRVVMDVVYNHLDSSGPFGVSSVLDKIVPGYYLRRN-VNGQIENSA

A--MNNTASEHFMVDRLIVDDLLNWAINYKVDGFRFDLMGHIMKSTMIRAKSAIRSLTRD

VHGVDGSKIYLYGEGWDFG-EVAQNKRGI----NASQINMS-GTGIGSFNDRIRDSVNGG

NPF-GNP----LQQGFSTGLFLEPNGYY---------QGNEA-DTRRELATYADHIQIGL

AGNLKDYVLRT-H-TGE-AKKGSDIYTF-DG-SPVGYTSSPVETINYVSAHDNETLFDIV

SIKT--------P---------------IGLSIDEKCRINHLASSMIALSQGIPFFHAGD

EILRSKSLDRDSYNSGDWFNKLDFTY---------E-TNNWG-VGLPPRDKN----EENW

HLIKPRLE-N-PS-FR--PLKNHILSVFDNFVDILKIRYSSPLFRLSTASDIEQRVRFHN

T--G-PS--MVPGVIVMSIKDAQNEK-CKMA-QLDKNFSYVVTIFNVCPHEVSIEIHDLA

--------SLGLELHPIQVN--S-S--DAL-VRQSAYEASK--GRFTVPRRTTAVFVQPR

-------

>UniRef90_A0A803NWK7_70_962 | Uncharacterized protein n=2 Tax=Cannabis sativa TaxID=3483 RepID=A0A803NWK7_CANSA | E_val=0

--SSSPVTSQLKDSLMYSRAYWVSESIIAWD-VDV----------GIN-GSLYLFASETA

S-LSITDGD-YGIQ---GH-D-----VKFKLEED-THG--LPQNVMEKFPHIK--DYRAF

KVPP--------SLDAK-----VLLKCQLAVAAFD-----D----------VGK-CSNIT

GLQLPGILDELFS------Y---SGPLGA-------VY----S-T----------E----

-----------AIALFLWAPTA-QAVRACIYKDSSG------------GSPL--EIVQLE

ET---KGVWST------KG-PK-S-----WD--GCYYVYEVSVYHP-----STLQIVKCH

ANDPYARGLSAN-----GGRTLFVDL---DSDTLKPDGWEKLALEKP-DL-LSFSDTSIY

ELHVRDFSASDLTVDNEFRGGYLAFTLQD---------SAGIRHLKKLSDAGLTHVHLLP

AFQFAD--VDDEKE---------------KWKSVDT------------------------

--EILEKFS---PDSAEQQALITAIQNSDGYNWGYNPVLWGVPKGSYASNP---NGSCRV

IEFRRMVKALNHIGLRVVLDVVYNHLHASGPYDENSVLDKIVPGYYLRRN-TDGFIENST

C--VNNTASEHFMVERLIIDDLLHWVVNYKVDGFRFDLMGHIMKRTMMKAKDAISSLTKE

KNGVDGSSVYIYGEGWDFG-EVARNGRGV----NASQVNLS-GTGIGSFNDRIRDAILGG

SPF-GHP----LQQGFVTGLLLQPNDHD---------HG--T-AAGEMLAASKDHIQVGM

AANLRDFVLTN-C-LGK-EVKGSEVLTH-GS-APVGFASNPTETVNYVSAHDNETLFDII

SLKT--------P---------------MEISVNERCWINHLATSIIALSQGIPFFHAGD

ELLRSKSMDRDSYNSGDWFNRLDFTY---------N-SNNWG-VGLPPKQKN----EKSW

PLIKPRLA-D-PS-FK--PQRSHILAAVENFSNLLRIRYSSPLFRLRTATAIQERVRFHN

T--G-PS--SIPGVIVMSIEDGHESQ-HGLS-QLDPIYSYIVVVINARPTEVSFTSPALR

--------AKHFELHPVQLM--S-T--DEV-IKASTYEEST--GCFVVPSRRTSVFVEPR

K------

>UniRef90_B9T3G1_75_964 | Pullulanase, putative n=1 Tax=Ricinus communis TaxID=3988 RepID=B9T3G1_RICCO | E_val=0

----STSTSQFQDSLLYSRAFWVSKTIIAWN-VDV----------GDN-GSCFLYASDTG

S-LSVSNA---GIQ---GH-D-----VEVKLEKY-NGG--LPENVVVKFPHIR--DYRAF

KAPP--------TLDAK-----SLLKCQLAVASYE-----A----------DGK-CRSAT

GLQLPGILDELYS------Y---DGPLGA-------HY----S-K----------N----

-----------AVSLHLWAPTA-QAVLVYIYKDSFS------------KVPL--EIHQLK

EV---NGVWSI------KG-PK-D-----WE--GCYYVYEVSVYHP-----STLRIEKCY

ANDPYARGLSSD-----GKRTLLVNL---TSESLKPEGWDNLAKEKP-SL-LSFCDISLY

ELHIRDFSANDQTVHPDFRGGYLAFTFEN---------SAGVLHLKKLSNAGITHVHLLP

TFQFAG--VDDVKE---------------NWKCVDN------------------------

--KMLETLL---PDSIEQQAQITAVQDNDGYNWGYNPVLWGVPKGSYASNP---NGPSRT

IQFRKMVQALNRIGLRVVLDVVYNHLHGSGPFDENSVLDKIVPGYYLRRN-IDGIIENST

C--VNNTASEHYMVERLIIDDLLNWAVNYKVDGFRFDLMGHLMKSTMVKAKIALHSLSVE

RDGVDGSSLYIYGEGWDFG-EVAKNGRGV----NASQFNLY-GTGIGSFNDRIRDAMLGG

SPF-GHP----LHQGFVTGLMLQPNGHD---------HGGKD-VEELMLTTAKDHIQVGM

AANLRDFVLIN-S-EGK-EVKGSEITTY-GG-EPVAYALSPTETINYVSAHDNETLFDIV

SMKT--------P---------------MEISVDERCRLNHLATSIIALSQGIPFFHAGD

EMLRSKSLDRDSYNSGDWFNRLDFSY---------N-SNNWA-VGLPPQKKN----EKNW

PLIKPRLA-D-PS-FK--PQKHHIVAATENFLDVLQMRYSSPLFRLTTANAIQERVRFHN

T--G-PS--WIPGVIVMSIEDGHEGF-PGLS-QLDPIYSYIVVIFNTRPTKVSFTSPALR

--------ARTFELHPVQVK--S-A--DEV-VKNSRYEASS--GCFTVPPITTSVFVEHR

-------

>UniRef90_A0A251QIZ0_76_965 | Uncharacterized protein n=11 Tax=Prunus TaxID=3754 RepID=A0A251QIZ0_PRUPE | E_val=0

-----ESTSTSQGSLLYSRAFWVSESIIAWN-VYV----------G-N-GSCYLLASKTA

A-LSLTSD---GIL---GE-D-----VKVKLEED-KHG--LPENVKEKFPHIK--DYRAF

NVPP--------DLDAK-----PLLKCQLAVATFN-----S----------DGR-CSDAT

GLQLPGILDDLFS------Y---NGPLGA-------LY----S-K----------Q----

-----------SVSLYLWAPTAQQEVCVCIYKEPSG------------GSPL--EVVQLE

EF---NGVWST------KG-PK-S-----WE--GCYYVYEVSVYHP-----STLRIEKCY

ANDPYARGLSSD-----GRRTLLVNL---DSDNIKPEGWDKLVDEKP-DI-LSFSDISIY

ELHIRDFSASDQAVHPEFRGGYLAFTLQD---------SAGAIHLKKLSNAGITHVHLLP

AFQFAG--VDDEKE---------------NWKSVDP------------------------

--EVLEKFP---SDSDKQQALITAIQNDDGYNWGYNPVLWGVPKGSYASNA---NGTYRA

IEFRKMVQALNRFGLRVVLDVVYNHLHGSGPVDDNSVLDKIVPGYYLRRN-TDGFIEHST

C--VNNTASEHFMVERLIVDDLLHWAVDYKVDGFRFDLMGHIMRRTMVKAKDALCSLTKE

RDGVDGSSIYIYGEGWDFG-EVANNGRGI----NASQFNIH-GTGIGSFNDRIRDAILGG

SPF-GHP----LQQGFVTGLLLQPNGHD---------HGPEA-VAEHMLAESKDHIQVGM

AANLRDFVLTN-C-EGK-EVKGSEVLTY-GG-TPVAYTLCPTETINYVSAHDNETLFDIV

SLKT--------P---------------MEISVEERCRINHLATSIIALAQGIPFFHSGD

EILRSKSLDRDSYNSGDWFNRLDFTY---------S-SNNWG-VGLPPKEKN----EKSW

PLFKPRLA-D-PS-FK--PQKSHIIAAVENFSNLLRIRYSSPLFRLRTANAIQERVRFHN

T--G-PS--LVPGVIVMSIEDGHEGV-PGLS-QLDPIYSYIVVIVNACPTEVSFASPSLQ

--------ARTLQLHPEQVM--S-T--DEI-VKRSTYNASA--GCFTVPPRTTSVFVEPR

G------

>UniRef90_A0A068TWS5_101_983 | Uncharacterized protein n=9 Tax=Coffea TaxID=13442 RepID=A0A068TWS5_COFCA | E_val=0

----------PQDSLLYSRAFWVSKSLIAWN-VEA----------G-N-GPCYLYASVTA

D-LFVADN---GIQ---GY-D-----SKIELQGG-DIG--LPQCVIEKFPHIR--HYKAF

NLPP--------DLDVE-----SLLKCQLAVAIFS-----S----------DGK-CSSAT

GLQLPGVLDDLFS------Y---CGPLGA-------VF----S-S----------E----

-----------TISLHLWAPTA-QGVHAFIYKDLSL------------SDPS--EIVQLK

GQ---NGVWSA------KV-PR-D-----WE--GYYYVYEVSVYHP-----STLQIEKCI

VNDPYARGLSAD-----GQRTLLVDL---DSDLFKPEGWDNLADEKP-HL-LSFADISIY

ELHIRDFSANDFTVHPELRGGYLAFTLQD---------SAGMLHMKRLASAGLTHVHLLP

TFHFGG--VDDERH---------------KWKDVGK------------------------

--QLLESLP---PDSDQQQAHITAIQDEDGYNWGYNPVLWGVPKGSYASDA---NGSCRT

LEFRKMVQALNRVGLRVVLDVVYNHLHASGPFDKNSVLDKIVPGYYLRSD-IDGFIEHST

C--TNNTASEHFMVERLILDDLLCWAVHYKVDGFRFDLMGHIMKRSMVKAKSMLQSLSME

KNGVDGSKIYIYGEGWDFG-EVAKNGRGI----NASQFNLS-GTGIGSFNDRIRDAMLGG

SPF-GHP----LQQGFVTGLSLEPNGHD---------HGDKS-AAELMLAVSKDHIQVGM

AANLRDFVLTT-F-DGQ-EVKGSEVLTH-DG-VPVAYALWPTETINYVSAHDNETLFDIV

SLKT--------P---------------KDISLDDRCRMNHLATSMVALSQGIPFFHAGD

EMLRSKSLDRDSYNSGDWFNRLDFSY---------N-SNNWG-IGLPPREKN----ERNW

PLIQPRLA-D-PD-FK--PHKSEIVAAVESFLTFLQIRYSSPLFRLRTANAVQERVKFHN

T--G-PS--WIPGVIVMSIEDGYEGV-PGLT-QMDECYSFVVVIFNACPTEVLFSSPVLR

--------ARTLQLHPMQLR--S-S--DDI-VKKSKYEASS--GCFTVPARTSSVFVESR

-------

>UniRef90_A4RRA2_181_1062 | Uncharacterized protein n=1 Tax=Ostreococcus lucimarinus (strain CCE9901) TaxID=436017 RepID=A4RRA2_ | E_val=9e-282

-----------------YRAIWVAERVIAVP-GDFA---------NDG-DSFTLVSSSTA

D-LKVTGE---GVV---GG-D------DVVTVVR-SGE--LPSSVCAKFPHIKAAGYRAL

EVPS--------SVNVR-----DALKRQIAVAAVD-----A----------AGK-PTDAT

GVQLQGAIDDLFA------Y---DGPLGA-------EF----GVN----------D----

-----------KVTLRVWAPTA-LNVALALFDEPRG------------EETRRVVAMTRD

ET---SGVWSA------TG-D--D-----FK--DKYYNFEVTVFNP-----TTGKVSTNV

ASDPYARSLAAD-----GRRAHVCDI---SRDDLKPKGWETF--EKP-KF-THPVDCSIY

ELHVRDFSALDETVSASARGKYLAFCEES---------SVCVSHLKKLADAGLTHVHLLP

SYDFGS--VPELPE---------------NQLSVDF------------------------

--KELAKLP---PNSRKQQEEISKIAWSDCFNWGYDPVHYGVPEGSYATNP---DGPRRI

FEYRQMVHALASNGLRVICDVVYNHTLSSGPSDVNSVLDKIVPGYYHRRN-FDGFIEAST

C--CNNTASEHYMMDRLIVDDLVHWAKDYKVDGFRFDLMGHLMLSTMLRAKDALQSLTLE

KDGVDGKSLYLYGEGWDYA-EVEKGRVGK----NASQLNLA-HTGIGSFNDRVREGCIGG

SPF-GDP----RMQGFLTGLYYTPNGAV--------DQGDQD-SQRYRMMEDGEKIIAAL

AGNVRDFVFVN-R-HGV-EVPSSSA-AW-PD-SNVAYAGEPEETVNYVSAHDNETLFDCI

MLRA--------A---------------ASVSLEQRCRINHLATAIVALSQGVPFFHAGD

EILRSKSLDRDSYSSGDWFNRLDYSG---------D-THNFG-VGLPGEQKN----GDRY

DFITPMLA-D-TS-MR--PSKEFIEEATRNFCELLSIRQSTPLLRLQTTRDIQRRMKFYN

R--G-PA--QTPGLIIASINDGDAST-PGLP-SLDANYKRVVLAFNATPNEISHHEAGLK

V----DFAGVDLELHPLVGGV-T-A--DAV-AMRSVF--IE--GVPTIPPYTWTVFVQHR

-------

>UniRef90_A0A2E0ZKD0_473_1357 | Alpha-dextran endo-1,6-alpha-glucosidase n=1 Tax=Anaerolineaceae bacterium TaxID=2024896 RepID=A | E_val=3.9e-267

------------GNISQRSAHWVAADTIAWD-IEA----------APG-NQYFFHYDPLG

GVFSLGFD---GIS---GG-E------AVPLTVD-PNG--LSEEIVAKFPHLG--SFTAL

KFSEDD------LRVAR-----VALKGQVAVSAQD-----E----------SGQ-IIDAA

GLQIPGVLDDLYP------Y---DGELGV-------TL----S-E----------G----

-----------VPTLTLWAPTA-RQVRLHLFDDANPN-----------TEAEI-LVMRPT

PS---NGTWSI------TG-ES-D-----WL--GKYYLYEVQVYVP-----TEGSVQTNL

VTDPYSFSLATN-----STRSQIVDL---SDPATMPAGWQGV--VKP-GI-EAPEDIVIY

ELHVRDFSVNDPSVPQEMQGTYSAFTVMD---------SNGMQHLAALSEAGLTHLHLLP

TFDIAT--IDEDKS---------------TWTTPSF------------------------

--EELASLP---PDSEEQQAMISAIRDQDPFNWGYDPFHYTVPEGSYAVYP---EGTSRI

VEYREMVQALNEIGLHVVVDVVYNHTNASGQ-SENSVLDRIVPGYYHRLN-STGRVETST

C--CQNTATEHDMMRKLMVDSVVTWATAYKIDAFRFDLMGHHMKEDMLAVRAALDVLTLA

EHGVDGRNIFTYGEGWDFG-EVGGNARGI----NATQFNMA-GTGIGTFSDRLRDAARGG

SPF-GGQ----TEQGFLNGLVVYPNETD---------QGSEA-EQLAQLHNFMDIIRLGL

AGNLSDFSFVG-S-DGT-MVTGSDI-LY-NG-SPAGYTADPQEQIVYISKHDNETLFDII

QYKA--------P---------------LETSTSERARMQTLGNSIVMFSQGVPFFQAGD

DLLRSKSLDRNSYNSGDWFNRLDFTY---------H-SNNWG-VGLPPAGDN----ESMW

PTMQPLLA-N-PN-LA--PTPDDIAFTRDTFRELLQIRAGSPLFRLQTAAEVQNRLQFHN

V--G-EG--QIPGLIVMSLSDVAG------E-DIDPNFDMVVVLFNATPDEMSFTETATS

ASLSAGVTNLPFSLHPIQQN--S-V--DGV-VQTAAFEAAS--GTFTVPAWTTAVFVLPQ

-------

>UniRef90_A0A3A5G238_253_1120 | DUF3372 domain-containing protein n=1 Tax=Corallococcus sp. H22C18031201 TaxID=2249217 RepID=A0A | E_val=3.2e-262

--------------LRMARAHWISRDTLVWE-PEQL---------KDG-ATVRLHTDPAG

A-MHLSGT---GVV---GG-E------ALTLTRS-TSG--LSAEQQARFPQLV--GRPVF

KLAASE------VARVP-----AMLKGQVALSVTD-----P----------DGK-PADAT

GLQLAGVLDDTDL------Y---TGPLGV-------SF----E-R----------G----

-----------RPTLRVWAPTA-RAVSVRVFEDARP------------SSAFVSVPMT-E

GE---KGVWSV------TG-DP-A-----WY--GRFYLYEVEVYVP-----REGATFVNL

VTDPYSVALSTN-----STRSQLVNL---ATSEFQPRGWSQL--AKP-AL-AAPEDIVLY

ELHARDFSIHDLTVPAEERGTFKAFTR-D---------SDGMRHLSRLARAGVTHVHLLP

VFDFAT--VNEDAA---------------SRREPEG------------------------

---DLVSMP---PDSAEQQARVAAVVDADGFNWGYDPLHYTVPEGSYSTNP---NGPTRT

LEFRQMVQSLNQHGLRVVMDVVYNHTHSAGQ-DARSVLDRIVPGYYHRLN-DDGNVETST

C--CQNTASENAMFEKLMVDSVVTWAREYKVDGFRFDLMGHHMKSTMLHLRAALDALTVE

KDGVDGKSIYIYGEGWDFG-EVQNNQRGV----NATQRNLA-GTGIGTFSDRLRDAARGG

GPF-SGI----QDQGFITGLSLWSNGTP---------QGTPA-EERARLLHDTDLIRLGL

AGNLRDFSLVD-S-TGK-DVTGAQV-DY-NG-SPAGYAEDPQEVITYVSAHDNETLFDAV

QLKA--------P---------------RAATLADRVRMHNLGISLVALSQGIPFFHAGD

ELLRSKSLDRNSYNSGDWFNQLDWTY---------Q-RNNWG-VGLPPGQEN----QDHW

PLFRPLLA-D-PA-LK--PAPADIQRALEHFEEMVRIRKSSPLFRLRTGDAVKRQVRFEN

T--G-PG--QTPGLIVMSISGE----------GTDTPDARAVVLFNSGVTQQTFQAERYR

--------NLALVLHPVQKD--S-T--DPF-VRAASFDAPS--GTFTLPARTTAVFLSP-

-------

>UniRef90_A0A355F9N6_449_1324 | DUF3372 domain-containing protein n=1 Tax=Acidobacteria bacterium TaxID=1978231 RepID=A0A355F9N6 | E_val=4e-258

----------------RAQAYWVNEDTIAWK-PGNP------AAIQAD-WTFALHYDEDA

G-LLLNNA---GIQ---GG-T------AIALAWD-PDG--LPADVLAKFPHLA--GFLAF

RIPTGQ------LGQVP-----DALQGQIAVEAKA-----G----------DGT-LVDAT

ALQIQGVLDDLYT------Y---DGALGV-------TF----A-A----------D----

----------RTPTFRLWAPTA-QDVRLLVTETAAD------------DSPVTEVAMT-R

GD---HGVWSA------SG-SP-T-----WY--GRYYRYQVTVFAR-----STGRIEVNK

VTDPYAVSLSRN-----SLRSQIIDLA--TDTTLVPAGWSSL--KKP-RL-DAPEDIVLY

ELHVRDFSANDASVPADYRGTFKAFTVNN---------SNGIRHLAGLAASGLTHVHLLP

VFDIAT--IDEDKT---------------QWQQPAG------------------------

---DLASFP---ADSEEQQSRVSAVADLDGFNWGYDPWHYTVPEGSYATDP---AGAARI

REFREMVQSLNRIGLRTVMDVVYNHTNAAGQ-NDKSVLDRIVPGYYHRLN-GDGNVETST

C--CQNTASEFNMMEKLLIDSVLTWATQYKVDGFRFDLMGHHMKANLVKLRQRLDALTLA

HDGVDGTKIYLYGEGWNFG-EVANNARGV----QATQANMA-GTGIGTFSDRLRDGVRGG

GPF-SGI----QEQGFINGLYTDPNATN---------QGSAS-DQLATLLLRSDWIRTGL

AGSLKSYVLED-R-NGN-DVAASAI-DY-NG-QPSGYTLDPQEVITYIEAHDNETLFDAI

AAKA--------P---------------VGTPMDQRVRMQNLGISLVGLAQGIPFYHAGV

ELLRSKSMDRNSYNSGDWFNKLDWTL---------V-DNNWG-VGLPPARDN----QANW

PVMQPLLA-N-PA-LK--PAPVHIGQAQAHFQEILGIRKSARLLRLRTAGEINDLLHLHN

T--G-PS--QIPGLLVYSVSDTLG--------AIDHRHSLAVVLFNARPDAVTFPLNVFP

--------CRGLALHPLQQQ--S-A--DPV-VRTATFTDST--CTFNVPGRTTAVFLKVR

P------

>UniRef90_A0A017T1F1_251_1126 | Alpha-dextrin endo-1,6-alpha-glucosidase n=1 Tax=Chondromyces apiculatus DSM 436 TaxID=1192034 R | E_val=8.2e-256

--------------LAAMQAHWVDRDTFAWK-LPVD------AAALEG-ATFTLVHAPAG

G-IRGEEG---RVT---GGSE------ELTLTLD-PAG--LGDAARARFPHLA--GHTAL

RFPSEA------QARLP-----ELLKGQLVVVATL-----P----------SGK-VL-AT

GVQLPGVLDDRFA------T---DSPLGV-------DL----S-G----------G----

-----------APTLRLWAPTA-RAVRLHLFDTPSG------------DATEI-VPMT--

EA---QGVWRA------DG-DP-G-----WM--ARYYLFEVEVFAP-----STGKVETNL

VTDPYALSLSMN-----SQRSQIIDL---HDPALAPLGWSTL--AKP-QL-ASLRDTTLY

ELHVRDFSIHDLSVPAGHRGKFLGFTHPA---------SDGMTHLTGLAQAGLTHVHLLP

TFDFAT--VNEDEA---------------QQQSPVI------------------------

----PAGLS---PDSQEQQAAVWALRDQDGFNWGYDPWHYTVPEGSYATAP---DGPARI

VEFRSMVQALNQAGLRVVMDVVYNHTSAAGQ-NPRSVLDRIVPGYYHRLD-DKGAINTST

C--CQNTATEHTMMQRLMVDSLLTWATAYKVDGFRFDLMGHHMKANLIEVRDRLAALTLA

NDGVDGSRIVLYGEGWDFG-EVAGQARGE----NATQAGMA-GTGIATFNDRLRDAIRGG

DPFDSGAA-L-QRQGFATGLYTAPNAHD---------QGTEE-EQRALLLDAADHIRLGL

AGNLRDYEFED-R-NGV-LVTGKDL-TY-KG-APAGYTAAPGEAVTYVEAHDNQTLWDML

QVKV--------A---------------AGTTLPDRVRLQNLSVSVCALGLGVPFFHAGM

EMLRSKSLDRDSFNSGDWFNRLDFTY---------T-TNNWG-VGLPPAEKN----QDSW

DLLGALLA-N-PA-LQ--VDQAHIVGAVAHFQEVLQIRRSSPLFRLETAEAVSARVRFHN

T--G-PD--QVPGVIVMAISDEVP----GQP-DLDPATGGVLVVFNARPEAVTLPLAGFD

--------TAGLSLHPVQEN--S-A--DGI-VKQALFTAGS-APTVMVPARTTAVFV---

-------

>UniRef90_A0A3D4NFI7_252_1119 | DUF3372 domain-containing protein n=1 Tax=Anaerolineaceae bacterium TaxID=2024896 RepID=A0A3D4NF | E_val=1e-252

------------GSLAKQKAFWINEDTILWN-TVG----------SSS-YQYSLFYSADA

T-LELTSD---GVA---GG-E------GIPLTFS-KSG--PGDNVLIQNPYLA--GFSTF

KIAKTD------LAKIP-----VLLRGQLAIIVHD-----N----------KGK-VVDST

GVQIAGVLDAIYN------Y---SGKLGV-------EF----N-G----------Q----

-----------NPTLRLWAPTA-ISVGVRIYDSSSA------------LVGKL-HPMTRD

DK---SGVWSI------EG-DK-E-----WK--NKFYLFEVQVYSP-----KTGKIEKNL

VTYPYSFSLSTN-----SLKSQMVDL---DDLELTPAGWDNL--EKP-EL-LNPEDIVIY

ELHVRDFSIHDETVPEKLRGKFLAFTVSD---------SNGMNHLTSLAEAGLTHIHLLP

IFDIAS--VDESSS---------------NWLSVDE------------------------

--ALLKTYP---ANSDQQSLAVSLIKGEDGFNWGYDPYHYTVPEGSYATNP---DGSTRI

KEFREMVKALNDNGLRMVMDVVYNHTSQAGQ-DAKSVLDKIVPGYYYRLD-TEGNVTTST

C--CQNTATEHKMMEKLMVDSVVTWATEYKVDGFRFDLMGHHMLSNMEAVRSALDVLTLE

KDGVDGKSIYIYGEGWDFG-EVAKNARGV----NATQLNIG-GTGIGVFNDRMRDAVRGG

NPF-DDL----RLQGFSTGLYLNPNAAE---------TSKPA-NQLEKLLDYSDWIRLSL

AGNLENYVITR-A-NGD-LVDGSRV-LY-GG-VQAGYTQDPQENIVYVSAHDNQTLFDAI

QVKA--------A---------------ANTNLTNRIRMNNLALSFPMFSQGIPFFHAGD

DMLRSKSLDDNSYDSGDWFNTLDWTY---------E-SNNWG-VGLPI--EG----SSNW

AIYKPLLG-N-AQ-LK--PNTSQITFASDVFKEYLEIRKSSPLFRLTTADQVSSCVSFYN

T--G-TK--QIPALIVMRIQDA--------N-DLDLNYGDILVFFNANQKSITFSEETLA

--------DLEYQLHPVLAS--S-V--DPI-VKTSAYVSSK--GSFTIPALTTSVFV---

-------

>UniRef90_A0A136P217_359_1226 | Alpha-1,6-glucosidase n=2 Tax=unclassified Chloroflexi TaxID=189774 RepID=A0A136P217_9CHLR | E_val=9e-250

----------------ERRAHWVTADTIAWD-IEP----------SDS-EEYRLLYSADG

S-IEVSVF---GIS---GDFV------SYPVSPS-VSG--LPDAVQTKFPHLA--GYQAF

TLSAEA------IEAAP-----DILRGQFAIAAYS----------------GDT-LVNLA

GLQIPGVLDDLYT------Y---DGDLGV-------TI----V-D----------G----

-----------VPTLSVWAPTA-QSVALNLYADAGTD-----------TAPTV-IDMARD

DA---TGVWSV------TG-EA-G-----WM--GQFYTYTVRVFAP-----TELAMVDNE

VTDPYSINLSQN-----SRRSQIVDL---SSPDLMPEGWLTY--EKP-DYGDAFEDITIY

ELHIRDFSIFDQTVPEDLRGAYLAFTVDD---------SAGMQHLKALAQAGLTHLHLLP

SFDIAT--INENRA---------------RHFEPDY------------------------

--DLLASLP---PDSPEQQAAYDRIRDLDGFNWGYDPYHYMAPEGSYATEA---TGTARI

LEYREMVKAINEAGLRVVQDVVFNHTNASGQ-SARSVLDRVVPGYYHRLD-AAGQVTRST

C--CENTATEHNMMRRLMIDTVVLNAVQYKIDGFRFDLMGHHMVSDMIAVREALDALTLE

AHGVDGKSIYIYGEGWDFG-EVQNGARGV----NATQFNLA-GTGIGTFSDRLRDAVRGG

SPF-GGR----DEQGLGNGIYTDPNGLN-----------PVN-EDLARVLRLTDLVRVGL

AGNLRDFQFVG-A-SGE-TITGFDT-DY-NG-SPGGYTLDPQEQIVYVSKHDNETLFDNM

LFRL--------P---------------PGSTADDIVRMQNLSLSYVLYAQGVPFLQAGS

DLLRSKSLDRNSYNSGDWFNRIDWTG---------Q-TNNFG-VGLPPAADN----QERW

ETMAAILT-N-ED-YY--PTPDHIALNAAVVREMLQIRYSSPLFRLRTADAIQARLSFLN

T--G-PD--QQPGVIAMNLSDLTG------E-DIDPAFEQIVVIFNGSDEPVTIGDARFA

--------GGSFRLHPIQQA--S-A--DAI-AQTATFDDAA--GMFTVPALTTSVFV---

-------

>UniRef90_UPI0004B2518B_236_1094 | pullulanase-type alpha-1,6-glucosidase n=1 Tax=Deinococcus pimensis TaxID=309888 RepID=UPI000 | E_val=3.6e-244

--------------LAKAQAHWLTRDTLAVK-PELV---------ANG-AILNLVYAPQA

G-LKLAAG---VVS---GSTT------SLPLIPA-EGG--LSAALKAKYPQLA--TYAVV

KVRPED------ASKVA-----EVLRSQLVVTSTG-----L----------DDK-PVDAT

GVQIYGVLDDLYA------Y---DGALGV-------TW----D-K----------T----

-----------VPTLRLWAPTA-QSVRLRLFDGPSG------------GEARV-VDMKRD

-----GGVWSV------TG-DA-S-----WK--NRFYQYEVKVYAP-----STQKVETNV

VTDPYSVDLALN-----STRSRIVDI---TDAAFKPQGWDNL--KKP-EL-RSVNDLTLY

ELHLRDFSVNDASVPAARRGTYLAFTEKA---------SRGMTHLKSLASAGLKAVHLLP

TFDIAT--IDEDRR---------------NWKATP-------------------------

---DLKALA---PDSAQQQAAVNAVKDQDAFNWGYDPYHYNVPEGSYAVNP---D--ARV

VEYRSMVAALNGAGLRVVQDVVYNHTNASGQ-AERSVLDRIVPGYYQRLN-LDGGVENST

C--CSNTATEHRMMQKLMVDSVVFWARNYKIDGFRFDLMGHHMREDMLRVRAALDALTPQ

KDGVDGKKVYVYGEGWNFG-EVENNKRGV----NATQVNMY-GAGIGTFNDRIRDAIRGG

NPF-GGL----QDQGFATGLVGLPNGQK--------QN-----TDTARLLKLSDQLRVGL

TGNLRDYRLVD-A-SGK-TVTGAQI-DY-NG-APTGYAASPRESINYASAHDNQTLWDAV

LLKA--------P---------------LTATTAQRVRMQNLANSLVALGQGVPFFQAGD

ELLRSKSFDTDSYNSGDWFNAITWDG---------A-DNGFG-RGLPLAEKN----EGNW

PLYRTLLG-N-AN-LK--VTAADRQRASDHLAEMLRVRASSTLFRMETAQQVQGGLTFLN

T--G-PK--QVPGLIVMRLQGQVG---------QGNPYRDVVVVFNATGSAVNFSDASLR

--------ALKLDLHPALAG--G-T--DAV-VKSSR--AAN--GTLTVPGLTTAVFV---

-------

>UniRef90_UPI000A070938_249_1102 | pullulanase-type alpha-1,6-glucosidase n=1 Tax=Deinococcus misasensis TaxID=392413 RepID=UPI0 | E_val=4.6e-240

--------------LSKSQAHWLTQDLLAVK-PGMV---------KEG-AKYQLHFDISA

G-LKLSAD---GVE---GG-R------AIEVDYV-GTG--LSEVLAQKYPHLK--GYAVL

KIRERD------LSKIP-----NILASQIAVSALG-----S----------DKK-PIDAT

GVQTAGVLDSMYA------Y---FGNLGA-------TF----G-K----------T----

-----------NITLKLWAPTA-QEIKLFLYNNATD------------KQPA--DQLEPT

FN---NGVWTA------TI-PS-K-----WK--NKFYQYRISVYHP-----LTGKIETSV

VTDPYSVGLSLN-----SKMSWLIDL---NDPSTQPAGWAGL--KKP-AL-DKFTDLSLY

ELHIRDFSAMDNTVPANQRGTYLAFTQGN---------ADGMKHLKSLAGAGLKAIHLLP

SFDIAT--INEYKP---------------DQKTTP-------------------------

---DLKAFG---PADTRQQEEVTKIKDQDAFNWGYDPYHFMVPEGSYAVSA-----SNRT

KEYRQMVMALNQSGLRVIQDVVFNHTNASGI-AEKSVLDKIVPGYYQRLN-LDGLVETST

C--CANTATENKMMEKLMIDSLVLWAKQYKIDGFRFDLMGHHMVQNMKNVRKALDALTLA

KDGVDGKKIYVYGEGWDFG-EVQGGKRGL----NATQLNLY-GNGIGTFNDRIRDALRGG

SPF-TDP----REQGFATGLLTSPNGFG-----------VTA-GQESRLQNLQDLLKVGI

AGNLRDFTFKN-A-AGE-TVKGSQV-MY-NGNSPAGYSANPQETINYVSAHDNETLFDAI

QWKA--------P---------------ASADINTRVRMNNLSLSVIMLSQGIPFFHAGD

DLLRSKSLDRDSYNSGDWFNHLDFTY---------Q-NNNWG-VGLPPAEKN----LDKW

DLMRPLLE-N-PA-LK--AGNAQIVRARDHFQDLLKVRYSSDLFRLSSARDVQNSLAFLD

T----PN-----SVLAYTLSGAVS--------KTNP-FSKIVVVFNASPEMQTVQTG---

--------ISGLSMHPVLQK--S-T--DAA-LKLVSV--SG--SQVNVPAFTTAVLV---

-------

>UniRef90_UPI00167490BB_247_1107 | pullulanase-type alpha-1,6-glucosidase n=1 Tax=Arenicella chitinivorans TaxID=1329800 RepID=U | E_val=1.6e-234

--------------LRQAKAFWLDANTLAWN-TVI----------DST-DQLTLHASATA

E-LGLDLD---SVT---GG-T------DYPLVL--LGG--LPDPLKAQFRHLS--DYTAI

SLPNLD------PQTKR-----QLLKTQLAISIRD-----Q----------DGK-LKDAT

AIQTPGAIDDLFV------F---DGELGP-------VY----Q-D----------D----

-----------TLSTHVWAPTA-QQVDLLLYEDSSQ------------TEPSAVFPMQED

PA---TGVWSA------SN-LA-D-----WD--RMYYRYRVTVYTK-----VTRQVEVNE

VTDPYSVSLSMN-----SRLSQFVDL---ADRDLKPRGWDYL--RKP-RL-RAAEDISIY

ELHVRDFSILDTTVPEHERGTFNAFTRNH---------SRGMRHLRRLSAAGLSHIHLLP

AFDIAS--VNEDRS---------------QQVSIDA------------------------

---DLSVLA---PDSVEQQAAVAAVQNQDGFNWGYDPLHFNVPEGSYSTDP---DGSKRV

REFRAMVKNLNRNGLRVVMDVVYNHTSSFGQ-YDQSVLDKIVPQYYHRYN-TNGGLETST

C--CANTASEHRMMEKLMIDSLLLWAKAYKVDGFRFDLMGHHSKQNILRVREALDALTLR

EDGIDGKNVYLYGEGWNFG-EVVNDARFD----QATQLNMA-GTGVGSFDDRGRDAIRGG

NPF-GGY----RDQGFGTGLHTNPNGYG-------------D-DRLSELLVLSDRTRSSL

AAGLKEFAFET-H-TGE-IRKAVNI-DY-FG-LPTGYTDDPQEQIAYIAAHDNETLLDGI

QAKA--------P---------------DTMSIADRARMQNFGNALVMLGQGIPFIHAGQ

DFLRSKSMDRDSYNSGDWFNAIDWTL---------R-ESGWG-KGLPIAEKN----EEKW

SIIAPLLA-N-PD-LK--PDRWLQFKSSLYFRELLRIRYSSPLFRLRDQQSVIDQVHFLN

T--G-PA--QTPGLIVMQLNGEGR------------HAKDLVVLFNGSNQTVSFEHDALD

--------APHYSLHPVQRW--S-I--DRS-TRRAFY--SN--QQFHIPAMTTSVFIAHR

-------

>UniRef90_UPI00194479F4_136_992 | pullulanase-type alpha-1,6-glucosidase n=1 Tax=Asanoa siamensis TaxID=926357 RepID=UPI0019447 | E_val=9e-231

-----------------SAAHWLRRGVIAWQ-PP-----------PSA-VRFALYSAPSG

G-MSVVDG---AVV---GG-S------AHPLGTR-AAG--LPSDVQRDYPHLA--GFRAL

TVPS--------SVPVS-----DVLTGQLAVAAFE-----A----------AGA-LVDAT

GVQIPGVLDDVYGG----AA---RRTLGV-------TW----SRH----------G----

-----------VPSFALWAPTA-LSVSLLV-------------------DGVAPVSLR-R

DR---DGVWTA------AG-SR-S-----WQ--GRSYVYSVRVFAP-----TTGRVETNT

VTDPYSVALTAD-----STRSVLASL---SDPALAPAGWRQL--RKP-AL-AAP---HVY

ELHVRDFSISDSSVPAAHRGTYAAFTDRS---------SAGMRHLRSLSSAGVTHLHLLP

VFDFAT--VPERRA---------------DQQVPGC------------------------

---DLAALP---PASEEQQACVEPVRPTDGFNWGYDPWHYTTPEGSYAVDP---VGAART

REFRSMVAGVNGAGLRVVMDVVYNHTTASGQ-AAKSVLDRIVPGYYHRLS-LTGQVETST

C--CANTATEHRMMEKLMVDSVVTWARDYKVDGFRFDLMGHHSKANMLAVRHALDRLTPA

RDGVDGRSILLYGEGWNFG-EVADDARFV----QATQANMA-GTGIATFSDRLRDAVRGG

GPFDANP----RLQGFGSGLLTDPNGDP--------VNGTAA-EQQARLLLAQDQIKVGL

AGNLRSYRFES-R-TGA-LVTGAEV-DY-NG-QPAGYAASPADTVTYVDAHDNETLFDNL

QYKL--------P---------------ASLPMPDRVRMNTLSLATTTLSQGVGFWHAGT

DLLRSKSLDRNSYDSGDWFNRIDWTG---------T-TSTWG-SGLPPRADN----EAKW

PYMRPLLA-D-PA-LK--PAPSDIRAAAAGSAEFLRIARSSPLFALPSAPEIQRRVGFPL

G--G-PT--QTPGVIVMTLDDTRG------R-DLDDRWERLVVIFNATPTTTTQSVPGAA

--------GRSYALHPVQAT--G-S--DPV-VKTAR-HTGG--GTFTVPPRTVAVFV---

-------

>UniRef90_A0A4V6PAY6_1051_1928 | Pullulanase-type alpha-1,6-glucosidase n=3 Tax=Jiangella TaxID=281472 RepID=A0A4V6PAY6_9ACTN | E_val=5.3e-228

---------QSQADLTQQKAHWLRRGLLAWD-LPAA---------AAD-WSFRLHAAPEG

G-LGVDAE---AVT---GG-T------SYPLTLD-QDG--LPDDVREEYPHLA--SYDAL

RLAERD------RKDIE-----EVLTGQVAVAAYD-----D----------LGR-LVDAT

GVQLPGVLDDVYAG----AA---DRELGV-------VW----N-G----------R----

-----------RPDIAVWAPTA-KHVDLLLTPPGA-------------TTEQR-IAMR-R

DR---DGVWTA------RG-NP-R-----WA--GAAYAFEVDVYVP-----ATDRVETNV

VTDPYSLALTTN-----SERSLVVDL---SDEALKPAGWDRL--RKP-RL-AQPEDSTIY

ELHVRDFSVGDTSVPEERRGTYLAFTDGD---------GAGMRHLSALAGAGLNTVHLLP

VFDIAT--VNENRA---------------EQASPDC------------------------

---DLEALTAADPAGTAQQECVTAVAGQDGFNWGYDPLHYTVPEGSYATNP---DGPGRT

REFRQMVAALNGAGLRVVMDVVYNHTHAAGQ-DDKSVLDRIVPGYYQRLS-ATGTVETST

C--CANTASEHAMMEKLIVDSVVTWARDYKVDGFRFDLMGHHSRSTMERVRAALDELTVP

GDGVDGASVYVYGEGWNFG-EVANNARFR----QATQLELF-GAGIGTFNDRLRDGVRGG

GPFDEDP----RLQGFGSGLFTDPNGAA--------VNGSEA-EQRARLLQHQDLIKVGL

TGNLRDYTFVD-A-TGA-TVTGADV-DY-NG-SPAGYTADPDEVITYVDAHDNETLYDAL

AYKL--------P---------------ADTPMADRVRMNVLSLATTALGQGPSFWHAGA

DLLRSKSLDRNSYDSGDWFNRVDWTG---------Q-DNTFG-SGLPPATDN----EAKW

DFMRPLLA-D-PA-VK--PAAGDMATSTALAQDLLRLRFSSPLFRLGSASRVQERVSFGA

G--G-PS--QPAGVVVMQLDDRAG------T-DLDPDRERLVVVFNATPSAQTVAVDD--

--------GASLRLHPVQAA--G-A--DPV-VRGSVV--GA--GGVTVPARTVAVFEQP-

-------

>UniRef90_UPI00174C4D16_1040_1894 | pullulanase-type alpha-1,6-glucosidase n=1 Tax=Nocardioides flavus Wang et al. 2016 TaxID=205 | E_val=2.1e-225

--------------LTKARAHWLRRDLVAWD-VSE----------PQS-RRFRLHWSQAG

D-LAVDAE---AVT---GG-S------SAPLTHD-PAG--LPEDVLADFPHLE--GYEAF

RLDRAT------ARRVP-----EILTGQLAVASYD-----A----------GGT-LHDAT

GVQVPGVLDDVYAD----AA---DADLGV-------TW----R-G----------G----

-----------RPTLALWAPTA-QDVSATV-------------------AGRT-VPMT-R

RR---DGTWTA------SG-PA-S-----WR--NARYTYDVTVFSP-----AAGEVVTNT

VTDPYAVALTTN-----SQAAVIADL---GDPSLAPEGWAGT--RAP-AV-DQPEDRAVY

ELHVRDFSIGDETVPAAERGTYLAFTREG---------SAGMRHLRRLADAGLNTVHLLP

AFDIAT--IEERRS---------------AQQAPAC------------------------

---DLESLP---PDSSRQQECVDAVRSKDGFNWGYDPLHYTAPEGSYATDP---DGTRRT

VEFRRMVQGLHGAGLQVVMDVVYNHTAASGQ-DPKSVLDRIVPGYYHRLN-ASGAVENST

C--CANTATEHRMMEKLMIDSVLTWARDYRVDGFRFDLMGHHSLENMTRLRSALDGLTLR

EDGVDGTAIYLYGEGWNFG-EVADDARFT----QATQLNLA-GSGIGSFSDRLRDAVRGG

GPFDEDP----RVQGFGSGLFTDPNGAP--------VNGTAE-EQREQLLHLQDLVKLGL

AGNLADFRFRS-S-TGE-EVRGAEV-DY-NG-QPAGYAADPGETVTYVDAHDNETLFDSL

ALKL--------P---------------AGTSMDDRVRMNTVSLSTVALSQGVVFWHAGT

DLLRSKSLDRNSYDSGDWFNRVDWQR---------R-ENTFG-SGLPPRADN----ESKW

DYMRPLLA-D-AA-LE--PTPAAMDEAHARALDLLRIRSSSPLFRLGSLEAIQAKVSFLD

A--G-------PGVVAMLVDDTAG------T-DADPDRDGVLVVINAGSTDATVT---GT

--------GDGWTLHDVQAS--G-S--DAV-VKGSTV--VS--DAVTVPPRTTAVF----

-------

>UniRef90_UPI00143A9353_138_1019 | pullulanase-type alpha-1,6-glucosidase n=1 Tax=Arthrobacter pigmenti TaxID=271432 RepID=UPI00 | E_val=1e-223

------TSDVPESLVAGAAAHWLAPGVLAWD-LQEA---------PEG-SFYRLYSSMDG

S-LEVIDG---VVT---NG-E------FIDLERS-SDV--LNPELAAASPHLK--SFDTL

TLPKKA------ARQAR-----HLLKGELLAVQLA-----A----------DGT-VLQAT

GVQAPRVLDALYPN----AL---KRTLGL-------SW----K-G----------Q----

-----------RPRFDLWAPTA-RQVTLQVYREGSG------------GEALATVPLK-E

GH---DGVWSL------LG-EK-D-----WK--DAYYLYEVEVYVP-----ETGKVERNV

VTDPYSVGLSTN-----SERSMIVNL---ADPSLAPSGWNST--KKP-AL-AQPEDLSLY

ELHVRDFSISDTTVPAGRRGTYAAFAETG---------SDGMQRLADLADAGLNAVHLLP

VNDIGT--IEERRD---------------VQAEPQC------------------------

---DLASFA---PDSDGQQACVSAVAGKDGFNWGYDPLHYTTPEGSYTTNP---EDATRI

REFRDMVAGLNGIGLRVIQDVVYNHTAGAGQ-ESSNNLDRIVPGYYHRLNPTSGAVETST

C--CPNTATENAMMGKLMVDSVVTLARTYKLDGFRFDLMGHHSKQNMLDVRAALDELTLK

KDGVDGKSIYVYGEGWNFG-EVANNARFE----QATQLNMA-GTGIGTFNDRLRDAVRGG

GPFDEDP----RVQGFGSGLWTQPNGAE--------VNGSPE-EQRARLLLSQDQIKVGL

TGNLRDYSFID-R-TGT-EVTGADV-DY-NG-SPAGYTADPQEAITYVEAHDNETLFDSL

AFKL--------A---------------PGTSMDDRIRFQTLSLSTTAFGQGVSFWHAGG

EALRSKSLDRNSYNSGDWFNLLDHTG---------T-ENGFA-RGLPPRADN----ADKY

EFMKPLLA-D-PA-LK--PAPAEIAAARDQALSLLEIRTSTPLFHLGTAELVQQKVSFPT

G--G-PE--QTPGVIVMHIDDISG------P-DLDSERSGVVVVFNASDTATTQTVAAAA

--------GQSFELHPVQRN--G-S--DEV-VKDAAFDSAT--GSFTVPPLTVAVF----

-------

>UniRef90_A0A0Q7K466_1055_1935 | Alpha-1,6-glucosidase n=2 Tax=Angustibacter sp. Root456 TaxID=1736539 RepID=A0A0Q7K466_9ACTN | E_val=2.1e-222

--------------LTKAKAHWVARDLIAWP-ANAVP----AGTSPER-LRWRLHWSVTG

G-LAVDDE---AVT---GG-S------SAALTYD-PAG--LPADVVAKDPQLK--GFLAL

RLARSS------ARQVP-----TILKGQVAVAQYD-----D----------LGR-LTDAT

GVQIPRVLDDVYAG----AR---SERLGL-------SW----H-G----------R----

-----------GPTFAVWAPTA-TDVDLLVWRAGRES-----------GAPQR-VQLR-A

DH---DGVWST------RG-RP-D-----WR--GAQYLYDVTVYAP-----STRKVEHNQ

VTDPYSLTLTPD-----SKRSVAVDL---DDPALQPKQWRTT--PSP-KL-AQSVDSTIY

ELHVRDFSVSDPSVPADHRGTYLAFAD-D---------GAGTKHLRALAKAGLNTVHLLP

TFDITS--IPEKRS---------------EQKTPDC------------------------

---DLASMP---PDSEQQQACVTAVAKDDAFNWGYDPYHWMAPEGSYATDP---DGAPRV

AQFRTMVGALHADGLRVVLDQVFNHTSASGQ-ADTSVLDKIVPGYYHRLNVSTGEVETST

C--CQNVATEHAMAEKMMVDAVVVWARDYRVDGFRFDLMGHHSRANMLAVRHALDALTLR

RDGVDGRSIYLYGEGWNFG-EVANNARFY----QATQGQLG-GTGIGTFSDRLRDAVRGG

GPFDEDP----RIQGLGSGLAGDPNGAA--------VNGDAA-QQAATLAHATDLVELGM

AGNLRAYRFRA-S-SGD-VVRGDQV-DY-NG-QPAGYADQPDEVISYVDAHDNETLFDTL

TYKL--------P---------------QSTSMADRVRMNTVSLATTALGQSPSFWHAGA

DLLRSKSLDRNSFDSGDWFNRLDWTG---------A-DNGFG-HGLPPKADN----AAKW

AYQKPLLA-D-PA-LK--PTANDVATASAAAADLLRLRSSSPLFTLGSADLINAKVGFPV

S--GTSE--AHPGVIVMRIDDSVG------S-DVDPALKGLVVVLNASASAVDQKVPGLD

--------GHALALSPVQES--G-S--DRV-VKTAAWDATS--GSASVPARTVAVFVE--

-------

>UniRef90_A0K1H2_143_1022 | Alpha-1,6-glucosidase, pullulanase-type n=6 Tax=Arthrobacter TaxID=1663 RepID=A0K1H2_ARTS2 | E_val=4.3e-221

-------DDVPDALGADAGAHWLTADTIAWK-APA----------AEG-TTYRLYSAPDG

G-LAVADG---QVT---GG-S------ALPLELD-AQG--LDAGLAAKYPHLA--GLASL

RLTEAG------SRQAK-----ELLKGQLLVAAIG-----P----------DGK-VTATT

GIQVPGVLDSLYPG----AA---ERELGL-------AW----K-G----------R----

-----------RPELSLWAPTA-RSVAVRTYASGSG------------GEPVATTAMK-P

GK---DGVWSI------TG-DK-D-----WN--GGYYLYEVEVFVP-----ETGKVERNL

VTDPYSVGLSAN-----SERSLFVDL---DDKSLAPSGWAKL--QKP-AL-SKPEDLSVY

ELHVRDFSITDDSVPAGHRGTYKAFTDTG---------SNGMKRMQELVDAGMNAVHLLP

VNDIGT--IEEHRS---------------EQREPLC------------------------

---DLAALP---ADSEEQQACVAGTAAKDGFNWGYDPLHYTTPEGSYSTNP---DGATRI

TEFREMVAALNTTGARVIQDVVYNHTSSAGQ-SGSNNLDRIVPGYYHRLNAVTGSLETST

C--CANTATENAMMGKLMVDSLVTLARTYKLDGFRFDLMGHHSKQNMLDVRAALDKLTLH

RDGVDGKNIALYGEGWNFG-EVANNARFV----QATQANMA-GTGIGTFNDRLRDAVRGG

GPFDPDP----RVQGFASGLFTDPNDSP--------ANGTPE-QQKAALLLAQDLVKVGL

TGNLKDYSFID-R-TGA-AVKGSDV-PY-NG-APAGYTSDPQEAITYVEAHDNETLFDAL

ALKL--------P---------------QDTPMADRTRMQTLALSTTAFGQGVSFWHAGG

ESLRSKSLDRNSYDSGDWFNVLDHTD---------A-TNGFG-RGLPPKADN----EDKY

GYMRPLLA-D-PA-QK--PASADITQARQRAEELLRIRKSTPLFHLGDAGLVQQKVSFPA

G--G-PE--QTPGVVVMRIDDSVG------T-DVDPNLRGLVVVFNASDEATSQTLSGTA

--------RSAYALHPVQAG--G-V--DSV-VKAAGYDAQS--GTFSVPARTVAVF----

-------

>UniRef90_A0A2Z3JFU7_263_1121 | Alpha-dextrin endo-1,6-alpha-glucosidase n=1 Tax=Deinococcus irradiatisoli TaxID=2202254 RepID=A | E_val=1.2e-219

--------------LTQQQAFWLTRDTLAVK-PELL---------AGG-ALLNLHYSASG

D-LKLTPA---GVT---GG-E------SLPLVRA-DEGDALSATLKARYPYLA--NYAVV

KLRPED------LGKVA-----GALRGQLALSSVG-----L----------DDK-LVGAT

GVQVWGVLDDLDT------Y---AGPLGV-------SW----Q-G----------G----

-----------VPSLTVWAPTA-QDVKVHVMIG---------------SQEKV-LPMR-A

GP---QGSWSI------TG-DP-S-----WK--NAPYRYEVKVYAP-----STGKIETNL

VTDPYSVGLTKD-----SRMSVMLDL---SDAAQKPAGWDSL--KKP-PL-GSVGDLNFY

ELHLRDFSVMDASVPQPQRGTYLAFTQTQ---------SDGMKHLRALAASGLKAVHLLP

TFDIAS--LPKDKA---------------TWKTPG-------------------------

---DLGKLP---PASEEQQAAVNAVKEQDGFNWGYDPYHYMTPEATYAVNP---D--ERT

KEYRQMVMALNAAGLRVVQDVVFNHTAASGE-ADKSVLDQVVPGYYHRLD-VNGAVTNST

C--CSNTATEHTMMRRLMVDTLVLQARQYKVDGFRFDLMGHHMVADIQAVRAALDALTLA

KDGVDGKQIYLYGEGWDFG-EVAGNARGV----NATQVNMY-GQGVGTFNDRIRDAVRGG

NPF-GGL----QEQGFATGLFTLPNGLP--------ANAATP-ENKAKLLKLTDQIRVAL

SGNLRDFKFVD-S-SGK-TVTGAQV-PY-NG-APTGYAASPREAINYVSAHDNQTLWDAV

LLKA--------P---------------LSATTAQRVRMQNLAYSLVLLGQGMPFIHAGD

ELLRSKSFDTDSYNSGDWFNSISWTG---------A-DNGFG-RGLPLAEKN----KDQW

DLYRPLLA-S-AA-LK--VTAADRQRASDNLETLLTIRNSTPLFRLPSAQAVQNQLSFLN

T--G-PN--QTPGVIVMKLSGG------------QAPYKNVLVVFNATPAAYTLKDAALA

--------PLKLDLHPALKA--G-S--DPL-VKTSSVNG----NTVTVPGLTTAVFV---

-------

>UniRef90_A0A0Q9TEA7_1039_1893 | Alpha-1,6-glucosidase n=1 Tax=Nocardioides sp. Soil805 TaxID=1736416 RepID=A0A0Q9TEA7_9ACTN | E_val=1.2e-218

--------------LAVAKAQWVRDDLVAWD-VPD----------AAS-RRYRLHWSTAG

D-LAVDAE---AVT---GG-S------SVPLRLD-PAG--LPADVLADFPQLE--GYEAF

RLDRTA------VRQVR-----EILTGQLAVASYD-----T----------GGA-LHDAT

GVQVPGVLDDVYAA----AA---DRALGV-------TW----R-G----------P----

-----------RPTLSLWAPTA-QSVAAVV-------------------GGQL-APMA-R

QA---DGTWTV------TG-PA-A-----WK--GAAYTYQVTVWAP-----AAQQVVTNR

VTDPYSVALTTN-----SAQSLVVDL---TDPALAPAGWSRT--AAP-RI-AQPEDRNVY

ELHVRDFSIGDETVPAGERGTYLAFTHDD---------TAGMRHLRGLADAGLNTVHLLP

AFDIAT--IEERRS---------------AQAVPAC------------------------

---DLEAMA---PDSTEQQACIDAVRGKDGFNWGYDPLHYTTPEGSYATDP---EGTRRT

VEFRRMVQGLNGAGLQVVMDVVYNHTAASGQ-AATSVLDRVVPGYYQRLD-AKGAVETST

C--CSNTATEHRMMEKLMIDSVLTWAREYKVNGFRFDLMGHHSLENMQHLRAALDRLTVR

RDGVDGRSVYLYGEGWNFG-EVADDARFV----QATQRNLA-GTGIGSFSDRLRDAVRGG

GPFDEDP----RVQGFGSGLLTDSNGAA--------ANGTPA-EQRTRLLHLEDLVKLGL

AGNLRDFSFRS-S-TGD-TVRGEDV-DY-NG-QPAGYAADPSETVTYVDAHDNETLFDSL

AFKL--------P---------------QGTSMADRVRMNTVSLSTVALSQGVVFWHAGT

DLLRSKSLDRNSYDSGDWFNRVDWSR---------R-ENTFG-SGLPLEEDN----AAKW

DFMRPLLA-D-PA-LE--PGPEAMDAAHAAALDLLRLRTSSPLFRLGSAAAIQDKVSF--

-----LD--AEPGVVAMLIDDTVG------A-DADADRDGVLVVFNATPSATTVS---GA

--------GTGWALHDVQAS--G-A--DEV-VKGATV--AA--DAVTVPARTTAVF----

-------

>UniRef90_A0A7W8IBJ0_1056_1941 | Pullulanase-type alpha-1,6-glucosidase n=1 Tax=Microbacterium sp. B8U3A TaxID=2735903 RepID=A0A7 | E_val=2e-217

-----------------SRAHWIDADTIAWP-ARL-------GDVSGD-TTWQLYGSPDA

A-LTIAGD---EVS---GG-D------PIDLTRI-AGG--LTDQQKERFPALA--DYLAL

RVEDAD------RAAVA-----DLLRGQLRVAQRD-----G----------DGS-LTAFT

GVQIPGVLDDLYAAE---VT---DDRLGV-------TF----S-G----------SGR--

---------KAAVTFRVWAPTA-QSASLLTWEAGAS------------GDPVR-REAAFD

AA---SGTWTV------AG-DR-G-----LA--GDEYVWEVEVYAP-----STDAVETNT

VTDPYSVALTEN-----STRSVAVDL---DDKTWMPKQWQKA--KAP-IV-KRPVDRAIY

ELHIRDFSITDTTVPEAERGTYLAFTR-D---------SAGTKQLKQLAKAGINTVHLLP

SFDIAT--IEEDRS---------------AQQVPDC------------------------

---DLASFG---PASAEQQACVEAVADTDGFNWGYDPYHFTTPEGSYAVDP---DGGARV

GEFRSMVGALHGMGLQVVLDQVFNHTAQSGQ-GEKSVLDRVVPGYYHRLN-AAGAVETST

C--CQNVATEHAVAQKLMVDSVVTWARDYKVDGFRFDLMGHHSTQNMLAVRSALDDLTVK

KDGVDGSKVYLYGEGWNFG-EVADNALFE----QATQGQLG-GTGIGTFNDRLRDAVHGG

SPVDSGST---FRQGFGTGLGTDPNGDP--------INGTTE-QALADLAKQTDLVKLGL

AGNLRDFALVT-A-DGT-VKTGAEI-DY-NG-QPAGYADEPDEIINYVDAHDNETLYDLS

VLKL--------P---------------VGTSMADRVRMNTLSLATVTLSQTPSFWHAGT

ELLRSKSLDRNSYNSGDWFNRIDWTG---------T-ESTFG-SGLPRAADN----EEKW

PIMEPLLA-D-PA-LK--PGAADMAAAESAALDLLRVRSEVGLLRLGSADLISEKVSFPG

S--G-PD--ADPGVIVMQIDDRAG------STDADEALDGALVVFNASPEPTTQTIDALA

--------GRGFALASAQAK--G-S--DAV-VKTTTWDAAT--GTVSVPARTVAVLVDAA

PVAT---

>UniRef90_A0A1A8ZUE4_948_1827 | Alpha-dextrin endo-1,6-alpha-glucosidase n=2 Tax=Micromonospora TaxID=1873 RepID=A0A1A8ZUE4_9ACT | E_val=7.9e-217

------TGVAKDLDITKQKAQWIDRSTVAWR-TGP----------TDG-RRYALVAAPTG

G-LSVTDG---ELT---GS------YTTLPLRAE-RNG--LTEAQRAAWPHLW--EYQAF

TLDRAD------LAKVP-----AALRGQLVVTERD-----A----------RGH-LLGAT

GVQIPGVLDDVYRA----AT---TAKLGP-------TF----A-G----------K----

-----------VPTLAVWAPTA-RTVSLQLFDSPT-------------AQPRS-VSMSRD

DR---TGVWSV------RG-SK-A-----WT--GQYYRYQVEAWQP-----AAQKVVTAS

VTDPYSVALAPD-----STHSQIVDL---DDPALAPAGWAKL--RKP-AA-VSSSRAQIQ

ELSVRDFSVADATVPTDRRGTYLAFTDPG---------TAGMKHLKALGDAGVNYLHLLP

AFDFAT--IPEKRA---------------DQRQPAC------------------------

---DLAALP---PDSDEQQKCVAAVADTDGYNWGYDPLHYTVPEGGYAVDP---TGARRT

TEFRQMVAGINQAGLRVVMDVVYNHTSAAGT-DPKSVLDQVVPGYYQRLL-DDGTVANST

C--CANTAPEHAMMGKLVVDSMVTWARQYKVDGFRFDLMGHHPKANILAVRAALDRLTVA

KDGVDGKRILLYGEGWDFG-EVAGDARFV----QATQANMA-GTGIGTFNDRLRDAVRGG

GPFDDNP----RIQGFASGLWSDPNGDP--------VNGSAA-EQKARLLHQQDLIKVGL

AGNLRDYRFTD-S-SGR-QVTGAQV-DY-NG-SPAGYTAAPGEAVTYVDAHDNEILYDAL

AYKL--------P---------------PGTSATDRARMQVLALAATALGQGTGFVTAGS

ERLRSKSLDRNSYNSGDWFNQIRWDC--------AQ-GNGFG-AGLPPAADN----QSKW

SYARPLLA-D-PK-LV--PDCAAINLADARYAELLKVRQSSPVFGLTTADQVQQRVAFPL

S--G-AQ--ETPGVVTMTLD---A------R-GLGGSWKSVTVVFNSTPEAAKQAVTGLR

--------GADVALHPVLRS--S-V--DPV-LRTASFDRAN--GTFTVPARSVAVFVQ--

-------

>UniRef90_A0A4R8XNT7_1036_1920 | Pullulanase-type alpha-1,6-glucosidase n=18 Tax=Cryobacterium TaxID=69578 RepID=A0A4R8XNT7_9MICO | E_val=4.8e-216

-----------------QRAHWVSEETIAWP-RDLLP----VNADPAA-LGYALHSSPAA

G-LAVADG---VVT---GG-D------TVALAYD-PAG--LSDAEKARFPALA--GSVAL

RPVGLD------RTDVE-----RLLTTQLAVSQSGTGAGDT----------TGT-LTAFT

GVQLPGVLDDLYADA---AT---DRTLGV-------TW----S-K----------K----

-----------RPTIAVWAPTA-QNVDALVWTAGDT------------ADPER-IAMT-R

QD---DGAWTA------TG-AK-N-----WT--GASFVFEVTVFAP-----TTGKIEVNQ

VTDPYSVALTPN-----STRSVLIDL---ADKNFRPPVWEKA--KAP-VI-EDAVDRSIY

ELHVRDFSISDDTVPEDLRGTYGAFGV-D---------SAGTRHLAELADAGLNTVHLLP

TFDIAT--IEEDRA---------------LQAVPAC------------------------

---DLASFG---PASPEQQACIQAIRDQDGFNWGYDPFHFSVPEGSYAVNA---DGGARV

AEFRGMVGALHEDGLQVVLDEVFNHTAQSGQ-GEKSVLDQVVPGYYHRLN-PQGAVETST

C--CQNVATEHELAEKLMVDSVVLWAKEYRVDGFRFDLMGHHSKDNLLAVRDALDALTLK

KDGVNGQAIYLYGEGWNFG-EVANNALFE----QATQGQLG-GTGIGTFNDRLRDAVHGG

SPVDGSTV---QQQGFGTGLGTDPNGVA--------INGTTE-QALARLAQASDLVRLGL

AANLRDFSFVT-S-DGT-VKRGDEL-DY-NG-SPAGYADQPDEVINYVDAHDNETLYDLL

ALKL--------P---------------SDTGMSDRVRMNTLSLATATLSQSPSLWHAGT

DLLRSKSLDRDSYNSGDWFNRIDWTG---------Q-TSNFG-VGLPSAAAN----EDKW

AIMRPLLE-D-PA-LK--PTPDDIAFASAQAQDLLRLKQSTELFRLGDAKLINEKLTFPA

S--G-PD--ATPGVIAMSVDDTVG------V-DVDQTLDGLLVLFNASPEPRTIRLGTLA

--------GRGFALSGIQAA--G-A--DAL-VKTTTWDAAT--GTVTVPARTVAVLVDAQ

E------

>UniRef90_UPI0014476323_1237_2121 | pullulanase-type alpha-1,6-glucosidase n=1 Tax=Sanguibacter hominis TaxID=1312739 RepID=UPI00 | E_val=2e-215

-----------------SRAVWLDSRTVALP-VGAVP------GESAS-RTWTLYSAPEG

G-LSATKE---KVTVPADA-L------SVDLTQD-ASG--LAPELRAKYPHLA--AYVAL

ELPESL-----TREELE-----EYLTGQLFLVQRS----------------GAV-VEYAT

GVQIAPVLDDVFAA----VV---DRDLGA-------TW----A-G----------D----

-----------VPTLAVWAPTA-RSVTTLVWTDGDIA-----------SDPTE-VPAV-R

QA---DGSWVT------AG-DA-S-----WK--DKPYKYDIEVYVP-----STGKVEHNV

VTDPYSVGLTLN-----STHSVLVDL---ADPAHRPTQWATT--KAP-TI-ARDVDRSIY

ELHIRDFSISDPSVPEAERGTYLAFAG-E---------GDGVKHLRKLAAAGLNTVHLLP

SFDIAT--IEEDRS---------------KQATTG-------------------------

---DLSGFA---PDGEEQQAAVAAIKDADGFNWGYDPMHYTAPEGSYASAG-NQDGGARV

AEYRTMVGALHKNGLQVVQDVVYNHTAASGQ-DPKSVLDRVVPGYYQRLS-ATGGVETST

C--CSNVATENLVAQKLMVDSVVTWAKQYRIDGFRFDLMGHHSKANMLAVREALDELTLS

RDGVDGKSIYLYGEGWNFG-EVADNARFE----QATQGQLN-GTGIGTFSDRLRDAVHGG

SPVDGGST---FQQGFGTGLGTDPNGNK---RGDGGVNDGSA-AELADLAHQSDLVRLGL

AGNLEDFTFVT-S-SGA-AQRGDEL-DY-RG-APAGYASEPGEVVTYVDAHDNETLFDLL

TLKL--------P---------------TATSMADRVRMNTLSLATTTLSQTPTFWHAGT

DLLRSKSLDRDSYNSGDHFNKVDWSG---------Q-SNNFG-VGLPSKEKN----EEKW

GLMRPLLA-D-PA-LK--PSATDIAAANAQALDLLRLRYSTPLLRLGSADLIKQKVSFPG

S--G-AD--ATPGLLVMRVDDTVG------T-DVDPALDGVLVVFNASPAPITEKVPGLA

--------GRALALSQVQKD--G-A--DAV-VKTTTWDAAS--GTVTVPGRTVAVLTQA-

-------

>UniRef90_UPI0018EFD7E9_1034_1912 | pullulanase-type alpha-1,6-glucosidase n=1 Tax=Salinibacterium sp. CAN_S4 TaxID=2787727 RepID | E_val=8.5e-215

-----------------QRAHWVSERTLAWP-RDLLP----TNGDPKD-QRFALYSSATA

G-LTSADG---TVT---GG-D------VVELEYD-PAG--LSDAEKALFPALA--DSVAL

RPVGVD------RAAVE-----RLLTTQLLLAQFA-----P----------DDA-LTALS

GVQLPGVLDDLFAEA---AS---GRTLGT-------SW----S-K----------S----

-----------TPTMAIWAPTA-QKVDALVWTGGDD------------ADPTR-VALS-R

QD---DGAWTA------TG-KK-A-----WA--GSSYAFEVTVFAP-----TTGAIEVNL

VTDPYSVALTVN-----SERSVLVDL---ANKRYRPKEWEKT--PAP-AI-ENPVDRSIY

ELHVRDFSLSDETVPENLRGTYGAFDV-D---------SAGTRHLAQLADAGLNTVHLLP

TFDIAT--IEEDRA---------------AQATPDC------------------------

---DLASFG---PASIEQQACIQAVADTDGFNWGYDPYHFSVPEGSYAVDP---RGGARI

AEFRSMVGALHETGLQVVLDEVFNHTAQSGQ-GEKSVLDKVVPGYYQRLN-ASGGVETST

C--CQNVATEHDMAEKLMVDSVVLWARQYKVDGFRFDLMGHHSKANMIAVREALDALTVK

KDGVDGSAVYLYGEGWNFG-EVADNALFE----QATQGQLD-GTGIGTFNDRLRDGVHGG

SPVDGGTI---QQQGFGTGLGTDPNAAP--------INGTPD-EALERLAQQTDLVRLGL

TGNLSDFSFVA-S-DGT-LKRGDQF-DY-NG-QPAGYASQPAENLNYVDAHDNETLFDLL

ALKL--------P---------------ADTPMSDRVRMNTLSLATATLSQSPSLWHAGT

DLLRSKSLDRNSYNSGDWFNRIDWTG---------Q-SNNFG-VGLPTAADN----EDKW

PIMIPLLE-D-PA-LS--PAPADIAASTAAAQDLLRLKQSTTLFRLGDAATIGEKLAFPG

S--G-PD--AAPGVITMTVDDTLG------A-DVDEELDGLIVVFNASPDATTQVIGGLA

--------GRDFGLSDIQSH--G-L--DET-VKQTTWDAAT--GTVTVPARTVAVLTEAE

-------

>UniRef90_UPI0018784C5E_1031_1911 | pullulanase-type alpha-1,6-glucosidase n=1 Tax=Microbacterium sp. YJN-G TaxID=2763257 RepID=U | E_val=2.1e-214

------------------RAHWIDETTLAWP-AGL--------GAADR-AAYQLYSASDA

S-LAVTDG---DVT---GA-E------PIALEPV-DGG--LTAEQKERFPALA--GYVAL

RVADAD---------AA-----ALLRTQLAVAQRD-----T----------AGA-LTAYT

GVQIAGVLDDLYADA---VG---DADLGV-------TF----R-G----------V----

-----------NPTFRLWAPTA-QSATLLTWDPSTGSGSPA------EADPIR-HEASYD

AA---SGVWQV------KG-KK-A-----LK--GDEYLWEVVVYAP-----ATGEIETNL

VTDPYSVALTVN-----SERSVAVSL---DDKQWKPKAWSKA--KAP-IV-EKPVDRAIY

ELHIRDFSISDETVPVAQRGTYLAFTR-D---------SAGTAQLRQLADAGMNTVHLLP

SFDIAT--IEENRA---------------AQAMPDC------------------------

---DLASYG---PADDAQQACVAAVADQDGFNWGYDPYHYSAPEGSYAVDP---DGGARV

AEFRSMVGALHGMGLQVVLDQVFNHTAASGQ-AERSVLDRVVPGYYHRLN-AAGAVETST

C--CQNVATEHRVAQKLMVDSVVTWARDYRVDGFRFDLMGHHSVANMLAVREALDALTLE

DDGVDGKAVYLYGEGWNFG-EVANNALFE----QAVQGQLG-GTGIGTFNDRLRDAVHGG

SPVDGSST---FRQGFGTGLGTDPNGDP--------INGSTG-QALADLGHETDLVKLGL

AGNLRDFAFTT-S-DGA-TTAGKDI-DY-RG-SRAGYADQPDEVINYVDAHDNETLYDLS

VLKL--------P---------------VDTSMADRVRMNTLSLATVTLSQSPSFWHAGT

ELLRSKSLDRNSYNSGDWFNRIDWTG---------Q-ESTFG-SGLPMAADN----EGKW

GIMAPLLS-N-PA-LK--PGAADIAAAEAAALDLLRVRGEVGLLRLGSAELIAQKVTFPN

G--G-PD--AAPGVIVMRIDDLVG------A-DVDPALDGALVVFNASPEAVAQTLPGLA

--------GRDFALTAVQAN--G-S--DAV-VKTTTWDAAT--GTVTVPARTVAVLVDDR

AAA----

>UniRef90_UPI00160B5DE0_983_1850 | pullulanase-type alpha-1,6-glucosidase n=1 Tax=Thermocatellispora tengchongensis TaxID=107325 | E_val=1e-213

--------------LAKSRAHWIDAGTVAWD-VEP----------SAS-LHYSLVLSASG

R-IAYQDG---DLT---GE-D----LRLIRL--V-PGA--LTEEQKARWPHLA--GYAAL

KVDPRD------AALVK-----QALRGRIVAAERD-----A----------SGA-LRAAT

GVQIPGVLDDVYAA----AT---TAELGP-------S-----A-G----------P----

-----------LPRLAVWAPTA-QKVRLALYREPSG------------GTPAL-HDMRRD

DH---TGVWSV------RG-LP-T-----WK--NRYYTFWVTVYSP-----AAGRVVTNE

VTDPYGLALAAG-----SGRSRLVDL---SDRAHRPEGWAGL--RKP-EP-VRQDRASIY

ELHVRDFSASDTTVPEALRGTYGAFAA-G---------GAGMRELRALARDGLTHVHLLP

VFDFAT--VPEKRA---------------ERTEPGC------------------------

---DLAAMP---PDSAGQQECVAATAATDSFNWGYDPLHYTVPEGSYASDP---E--NRT

REFRGMVKALNEAGLRVVMDVVYNHTHAAGQ-DAGSVLDRIVPGYYHRLL-DDGTVANST

C--CANTAPEHAMMGRLVVDSVVTWAREYKVDGFRFDLMGHHPKANILAVRRALDRLTLD

EDGVDGKSIILYGEGWDFG-EVAGGARFE----QATQANMA-GTGVGTFNDRLRDAVRGG

GPFDADP----RVQGFASGLAGAPNGSP--------ANGTEE-ERRARLLRYHDLIKVGL

AGNLRDYAFTS-SVSGE-RVTGAQV-DY-NG-SPAGYTAAPGEAVTYVDAHDNETLFDAL

AYKL--------P---------------QDTLMADRVRMQTLALAAATLGQGTAFVHAGS

ERLRSKSLDRNSYDSGDWFNRLLWDC--------SA-GNGFG-AGLPPKADN----EAKW

PYARPLLA-D-PA-LR--PGCADIGAARAAYGELLRIRASSPAFALGSLDEVQRRLAFPV

A-----E--ETPGVITMRVD---V------S-GLDPRWKSVTVVFNATPGEQRQTVSALA

--------GADVALHPVQAA--S-A--DPV-VRRSAFDAGT--GTFTVPPRTVAVFVQS-

-------

>UniRef90_UPI00146F8F92_1091_1964 | pullulanase-type alpha-1,6-glucosidase n=1 Tax=Microbacterium TaxID=33882 RepID=UPI00146F8F92 | E_val=5.6e-213

------------------RSHWIDAETIAWP-SDL-------GSSAAD-ASWQLHSSADA

S-LALADG---QIS---GG-D------AIDLSRI-DGG--LTADQKERFPALA--GFTAL

RVEGVD------RDAAA-----DLLRGQLMVSQRD-----A----------AGT-LKAFT

GVQIPGVLDDLYADA---VA---DVELGV-------TF----K-K----------D----

-----------KPTFRLWAPTA-QSATLLTWEAGAD------------GDPVR-HEAAWD

EA---SGVWTV------KG-AK-S-----LK--GDEYLWEVVVYAP-----ATGAIETNR

VTDPYSVALTVN-----SERSVAIDL---DDKSWKPKQWEKT--KAP-VV-DRPVDRAIY

ELHIRDFSITDESVPAEERGTYRAFTR-D---------SAGTKQLRQLEAAGINTVHLLP

SFDIAS--IEEDRA---------------KQQTPDC------------------------

---DLASYG---PASTEQQACIQKVVDADGFNWGYDPYHYSAPEGSYAVDP---EGGARV

HEFREMVGALHGMGLQVVLDEVYNHTAQSGQ-GDKSVLDKVVPGYYHRLN-LAGKVETST

C--CQNVATEHEVAEKLMVDSIVVWARDYKVDGFRFDLMGHHSKENMLAVRAALDELTLK

HDGVDGTSIYLYGEGWNFG-EVANNALFV----QATQGQLG-NTGIGTFTDRLRDAVHGG

SPVDGASI---FYQGFGTGLYTDPNGRP----------GVPG-DPAGDLARKTDLVKLGL

AGNLRAFTMTA-A-DGS-VKRGDQF-IY-NT-SPAGYADQPDEIITYVDAHDNETLYDHQ

VFKL--------P---------------VDTSMADRVRMNTVSLATTTFSQTPSFWHAGT

ELLRSKSLDRNSYNSGDWFNRIDWTG---------K-ESTFG-SGLPRESDN----KDKW

PLMQPLLE-N-PA-LK--PGASDIAKAEASALDLLRVREEVGLLRLGSADLIQQKVTFPG

S--G-PD--ATPGVIVMLIDDLVG------E-DVDPALEGALVVFNASPEATTEVLPELA

--------GRGFTLTPALAK--G-S--DAV-VKTTTWSAAS--GTITVPARTVAVLVDAQ

-------

>UniRef90_A0A4Q2L4Y8_1035_1920 | Pullulanase-type alpha-1,6-glucosidase n=1 Tax=Agromyces albus TaxID=205332 RepID=A0A4Q2L4Y8_9MI | E_val=2e-212

-----------------SRAHWVSEDTLAWP-AELL-----GGASAAS-ATWTLEHSADA

T-LVAADG---AVS--GGG-T------PVELALD-PAG--LTDAQLAKFPALE--GFIAL

HPVGVD------RAAVQ-----QLLTEQLAVAQS------T----------DGA-LSAFT

GVQMSGVLDDLYADA---VA---SVPLGV-------SW----E-G----------D----

-----------APTLSIWAPTA-QSVSVDVFPTGES------------GMPAT-TPAVFD

EA---SGVWSVTDATDVNG-DP-V-----SS--GDEYRWSVEVYAP-----TTGAIETNS

VTDPYSVALTTN-----SVRSVVVDL---DDESLRPAEWEAT--PAP-TV-ERAVDRAIY

ELHVRDFSISDETVPEEERGTYRAFTR-D---------SAGTAQLEELAEAGINTVHLLP

TFDIAT--IEERRD---------------QQATPAC------------------------

---DLASFG---PASAEQQACLESIRDLDGFNWGYDPFHFQAPEGSYAVDP---NGGARV

AEFREMVGALHATGLQVVLDEVYNHTAASGQ-GEKSVLDKVVPGYYQRLN-AVGGVETST

C--CQNIATEHEVAEKLMVDSVVLWAREYKVDGFRFDLMGHHSKANMLAVRAALDELTLE

DDGVDGSAVHLYGEGWNFG-EVANNALFE----QATQGQLG-GTGIGTFNDRLRDAVHGG

SPVDGSST---FVQGYGTGLATDPNGDP--------INGTPE-QALADLRHQTDLVKLGL

AGNLRAYELTD-A-SGQ-VVRGDAL-DY-RG-SPAGYADQPDEVINYVDAHDNETLYDLS

VFKL--------P---------------TDTPMADRVRMNTLSLATVTLSQSPSFWHAGT

ELLRSKSLDRNSYNSGDWFNRIDWTG---------Q-ESTFG-SGLPPAADN----EGKW

PIMAPLLA-D-PA-LK--PGVADIAAAEASALDLLRVRDEVDLLQLGSAALIDEKVSFPI

S--G-PD--ATPGLIVMQIDDLVG------D-DVDPELDGALVVFNATPEAITESVDGLA

--------GREFALANALAN--G-A--DPV-VKATEWDAAT--GALTVPARTAAVLVDGR

E------

>UniRef90_A0A7X7NM05_339_1228 | Pullulanase-type alpha-1,6-glucosidase n=1 Tax=Actinomycetales bacterium TaxID=1911520 RepID=A0A | E_val=5.3e-212

--------------LTAAKAHWLTPGLLAWP-ADAVP----AGAELAL-LDWRLHWSPDG

G-LALDAE---AVT---GG-E------SAPLTYD-PAG--LPAEVVADHPELA--DYLAL

RLDKKV------AKDAD-----DILRGQVAVALYD-----D----------LGR-LKDAT

GVQIPGVLDELYAE----AQ---DARLGA-------TW----Q-G----------G----

-----------KPTLRLWAPTA-HDVHLLLWPAGASGDAPV-------EDARR-VDLK-R

AK---DGSWSV------KG-DR-S-----WA--GRNYLYEVEVFAP-----STGAVETNL

VTDPYSVALTLN-----STRSVLVDL---ADRALQPALWRTA--ATP-AL-GDEVDQTIY

ELHVRDFSVSDPDVPAEHRGSYLAFAD-Q---------GHGIRHLETLAEAGLNTVHLLP

TFDIAS--IPENRWD------------PSEVTEPAC------------------------

---DLESFA---PDSPEQQACVKAQAGTDAFNWGYDPWHFLAPEGSYASTAQAAHGGARV

AEFRTMVGGLHDAGLRVVLDQVYNHTAQSGQ-GEKSVLDRVVPGYYHRLN-AMGAVETST

C--CQNVATEHVMAEKLMVDSVVLWARDYKVDGFRFDLMGHHSRENMEAVRAALDELTVR

RDGVDGSAVTLYGEGWDFG-EVAGNRLFH----QAIQGQLA-GTNIGTFNDRLRDAVRGG

GPFDEDP----RKQGFGSGQFTDPNGAP--------INGDQT-AQQRSLAHDTDLVQIGL

AGNLRDFELRSAE-TGD-LVTGEQL-DY-NG-APAAYAEDPDEVINYVDAHDNETLFDSL

TLKL--------P---------------QATTMQDRVRMNTLSLATVTLGQSPSFWHAGA

DLLRSKSLDRNSYDSGDWFNLLDFTM---------A-DNGFG-RGLPPEGDN----GSKW

DFQRPLLA-D-PA-LA--PAPEDIAAASAMAQDLLELRFSTPLFRLGTAEAIAEKLTFPV

S--G-TD-QAHDGVIVMRIDDTAG------A-DVDPALDGLVVVFNASDESVAQELPGLT

--------GANLSLSPVQVD--G-A--DEV-VKTSTWDAGS--GTLSVPARTVAVFV---

-------

>UniRef90_A0A3P0X6C1_269_1149 | Pullulanase-type alpha-1,6-glucosidase n=2 Tax=Acidipila sp. EB88 TaxID=2305226 RepID=A0A3P0X6C1 | E_val=3.6e-211

------------------QSYWIDATTIAVQ-PQYA---------QSG-YTWTLNASPTA

G-LQITAT---GLT---GG-T------RIALTPF-SGT--LTQQELTRFPQLA--GYALF

KLPVQID-----PATVR-----QALKSQLEVAATA-----A----------DGT-LKYTT

GVQTFGALDALLA------Y---AGPLGV-------VI----P-H----------GITPD

SILQGAESPTYPIQIKVWAPTA-QSLSLLLFREPTD------------TVPAKTIAM--Q

EQ---NGVWSA------GV-PI-E-----WR--SRYYQLQARVYVA-----AQQAILTNT

TTDPYSVDLSLN-----GTMSRITDL---ASETTKPALWDFS--PSP-RL-DNILDTSIY

ELHVRDFSVADSTVPASHRGTYDAFADQG---------SDGMRHLHALALSGLKAVHILP

SFHFAS--VNEDKS---------------TWTTTG-------------------------

---NLAQYP---PDSEQQQQAVTAVQSTDAYNWGYDPVHYMTPEGAYAINP---D--NRV

LEYRAMVQGLHLAGLRVIQDVVFNHTSASGQ-DPSANLDELVPNYYHRLD-ANGVLETGS

C--CADIASEHQMTEKLMIDTLLLNAKQYKIDGFRFDEMGMHFVYNMVDIRNALDALTVE

RDGIDGSKIYVYGEGFQIA-EAANNAIGP----NASQINLY-GTGIGSFNDRMRDSVRGG

NAFGTAE----QVQGFATGLLTDSSSYTN-------QNTAAS-DQRTTLLQGADLIRLSL

AGALRDYTFQN-Y-LGQ-TTTGAQL-NY-GG-QPAGYTATPLEDVNYCSVHDNQDLFDAV

QLKS--------A---------------EGDTIGQRARRQVLAMSLIALGEGIPFFHGGD

DLLRSKDMDNNSYDSGDWFNKIDWSG---------K-GNNWG-TGLPIASQN----QSEW

PVMQPLLA-N-PA-LE--ATPTSLAATTQAFQEFLRIRYSSGLFRMATFNEVQQNLSFLN

T--G-QS--QTPGLIVMKLDDHGH------A-YGDA--HHIVVFFNAANTSLTYTDSSLQ

--------GMALHLHPVQQA--S-S--DAT-VQQSTFNSKE--GSATIPALTTVVFVSE-

-------

>UniRef90_A0A386WFP9_960_1831 | Alpha-dextrin endo-1,6-alpha-glucosidase n=39 Tax=Micromonosporaceae TaxID=28056 RepID=A0A386WFP | E_val=1.1e-210

--------------LTKQKAQWIDRSTVAWA-TGP----------TDG-RRYALAVAPAG

G-IGLADG---ELT---GT------YTTIGLRAQ-RNG--LTEAQRQAYPHLW--ASHAF

TLDRAD------LARVP-----AALRGQIVVTERD-----A----------DGA-LLAAT

GVQIPGVLDDVYAA----AT---GAKLGP-------TF----A-G----------R----

-----------VPTLAVWAPTA-RTVSLELYDSPT-------------AQPTS-VAMRRD

DR---TGVWSV------RG-TK-A-----WN--GRYYRYRVQAWQP-----ATQKMVTAS

VTDPYSVALAPN-----STHSQIVDL---TDPALAPAGWANL--RKP-AA-PPASKVQIS

ELSVRDFSIADTTVPAERRGTYLAFTDPN---------TAGMKHLRALGDAGVNHLHLLP

AFDFAT--IPEKRA---------------DQARPNC------------------------

---DLAALP---PDSDEQQKCVAAVAGTDGYNWGYDPLHYTVPEGGYAVDP---NGAKRT

TEFRRMVAGVNQAGLRVVMDVVYNHTSAAGA-DPKSVLDQVVPGYYHRLL-EDGTVANST

C--CANTAPEHAMMGKLVVDSLVTWAKAYKVDGFRFDLMGHHPKANILAVRAALDKLTLA

RDGVDGRKILLYGEGWNFG-EVADDARFT----QATQANMA-GTGVGTFNDRLRDAVRGG

GPFDANP----RVQGFASGLYTDPNGDE--------VNGSAA-EQRARLLHQHDLIKVGL

TGNLRGYRFTD-S-TGK-RVTGAQV-DY-NG-APAGYTAAPGEAVTYVDAHDNEILYDAL

AYKL--------P---------------QGTSAQDRARMQVLALATTALGQGAGFVTTGS

ERLRSKSLDRNSYNSGDWFNQIRWDC--------AQ-GNGFG-AGLPPEADN----KDKW

SYAKPLLA-D-PS-LV--PDCAAIELADARYAELLRVRRSSPVFGLDTAEQVQKRVAFPL

S--G-TK--ETPGVLTMTLD---A------R-GLDGKWKSVTVVFNATPETAEQTVTGLR

--------GANVALHPVLRN--S-A--DPA-LRTASFDRAA--GTFTVPARSVAVFVQ--

-------

>UniRef90_A0A0D9AIM1_146_1037 | Alpha-1,6-glucosidase n=1 Tax=Pseudomonas stutzeri TaxID=316 RepID=A0A0D9AIM1_PSEST | E_val=3.2e-210

------------VAIKGARAHWLDPFTLALV-DGA----------PDA-SRIELRYSTSA

D-IRIDSET-RTVR---GG-T------ALALQ---PAT--LREGLKRQHPHLA--EAPAF

HLSAH-------ARDLR-----RAVMSQLVVVTYD-----D----------DDK-VLDAT

EVQTAGILDLLFA------Y---NGELGA-------RV----D-A----------R----

-----------GVSFTLWAPTA-QRVRLHVFDEAKR------------LLPG--YPKIMR

ER---LGVWSL------DG-PR-S-----LD--RQYYQYEVTAYRP-----STGKIETTL

VSDPYALSLSRN-----SQYAQVVDL---DADDLKPSGWDAL--RPP-HP-ARPEASVIY

ETHLRDFSASDASLPAELRGTYGAFTHPD---------SNGMRHLRDLKNAGLTHVQLLP

VFDIAT--IDEDPT---------------RRVDLDDPFAKLCDLSTQARENWSQYCSATS

IRLVLQGFD---PASGQAQALYNDLRALDNFNWGYDPFHFSAPEGSYASDA---EGAQRI

IEFRQMVQALANDGLATVMDVVYNHTNASGL-TDKSVLDKIVPGYYHRRNPSTGAVETST

C--CENTASEHRMMAKLMTDSLEVWARDYKIAGFRFDLMGHHLRKNLVDAYRAVRKI---

-----DRDTYFYGEGWDFG-EVAGNARGI----NATQLNMA-GTGIGTFNDRQRDAVRGG

SPFDSGES-IRRNQGFANGLYVLPNELT----------GAGE-QEKAQLLHAADLIRVGI

AGGLRDYQFVT-A-DGS-TRSGSQI-DY-NG-QPAGYTLDPQETINYVSKHDNQTLWDNN

QYKL--------P---------------VGLPVADRVRLQLLALSVPLFSQGVPFIHLGS

DILRSKSMQRDSYDSGDWFNAVDFSY---------Q-DNNWN-KGLPRADKD----ADNW

PLVRQVIA-N-PH-TK--PSAVNIVAAKRRFLELLKIRSDSPLFQLDSAREVQRRLRFHN

T--G-PT--QQPGVIAFSLADGPG----DGR-DLDRRYQALMVVFNASAERVRLP-----

-------GADGYQPHPVQRD--S-S--DPI-SRQARV--VA--GEFEVPPFTSAVFLQPQ

R------

>UniRef90_UPI0018DF750C_197_1069 | pullulanase-type alpha-1,6-glucosidase n=1 Tax=Cellulomonas sp. PSBB021 TaxID=2003551 RepID=U | E_val=1.1e-209

-----------------ARAHWVTADTLAFP-QDLL-----GDADAAD-LTWSLHASPTG

G-LELADG---AVE---GG-E------PVALTYD-PAG--LPGDVTAAFPALA--GYAAL

HLSASD------AARAP-----QLLEGELRVLAA-----------------GDS-PVAFT

GVQVPGVLDDLYADA---AA---TADLGL-------TW----D-D----------G----

-----------TPSFALWAPTA-QDVDLLVWPGEVS------------GEPTR-VPAE-R

GD---DGVWRV------AG-EA-G-----WT--GAAYRYAVTVYAP-----SLRRVVVND

VTDPYSVALTQN-----STHSVVVDL---ADPATRPAIWTDT--PSP-TV-EQAVDRAIY

ELHVRDFSIADETVPAAERGTYLAFAR-D---------SAGTRHLAELAGAGLTTVHLLP

TFDIAT--IEEDRG---------------EQESPAC------------------------

---DLASYA---PDSTEQQACVTAVADADGFNWGYDPFHYQVPEGSYAVDP---D--ARV

GEFRTMVGALHATGLEVVLDEVYNHTSAAGQ-ADTSVLDKVVPGYYHRLD-ATGAVQTST

C--CSNIATEHALAGKLMVDSVVLWAREYKVDGFRFDLMGHHSVANMQAVRDALDELTLA

DDGVDGSRIYLYGEGWNFG-EVADDALFE----QARQGNLG-GTGIGTFSDRLRDAVRGG

GPFDEDP----RVQGFGSGQLTDPNGAA--------VNGDAA-AQAATLAHQTDLVRLGM

AGNLADFSFLA-S-DGT-VRTGAQV-DY-NG-QPAGYATEPDEVVTYVDAHDNETLWDSL

TMKL--------P---------------QATSMDDRIRMNTLSLATTALAQTPSFWHAGA

DLLRSKSLDRNSYNSGDWFNAIDWTG---------H-DNGFG-KGLPPRPDN----EAKW

PYMAPLLA-D-PA-LK--PSADDVATASDAALDLLRLRASTRLLRLGSADAIETKLTFPT

S--G-PD--ATPGVIVMSVDDTLG------A-DADPALDGVLVVLNAGTEATTQVVPELA

--------GREYALSPVQVA--G-S--DDV-VKVTAWDTAT--GTVTVPARTVAVLVEAQ

-------

>UniRef90_A0A6V8KUV1_919_1779 | Alpha-dextrin endo-1,6-alpha-glucosidase n=1 Tax=Phytohabitans houttuyneae TaxID=1076126 RepID=A | E_val=1.9e-209

--------------LSKSRAHWIDRGSVAWQ-IPS----------TDG-KRYELALAPAG

G-LSIVDG---ELA---GS------HETIPLAAT-RNG--LTEKQRGRFPHLW--QYTVL

SLSKKD------QSRVA-----EALRGQVVVTERD-----H----------EGK-LLAAT

GVQVPGVLDDLYAGR---AT---KATLGP-------TV----K-G----------D----

-----------RATLSVWAPTA-RSVALELYDAPS-------------GSPQV-TPMRRD

GR---TGVWSA------TG----P-----WK--GKYYKYRVEAWQP-----AVQRVVTAS

VTDPYSLALAAD-----STHSLVADL---ADPALSPPGWDRL--RKP-AA-VPATRTQIT

ELSVRDFSIADATVPAPQRGTYAAFTG-D---------GAGAAHLRKLGDAGVTHVHLLP

AFDFAT--IPERRA---------------DQATPPC------------------------

---DLAALP---PDSDKQQACIAEVAERDGYNWGYDPLHYTVPEGGYAVDP---D--SRT

REFRQMVAALNGSGLRVVMDVVYNHTSASGV-DDRSVLDQIVPGYYHRLL-ADGAVANST

C--CSNTAPEHAMMGKLVVDSVVTWARQYKVDGFRFDLMGHHPKANLLAVRKALDGLTPR

RDGVDGKAILLYGEGWDFG-EVAGGARFV----QATQANMA-GTGIATFNDRLRDAVRGG

GPFDANP----RQQGFASGLYTDPNGDP--------VNGSTD-EQRARLLHQHDQIKVGL

AGNLAGYRFTD-T-SGK-LVTGAEV-DY-NG-SPAGYTAAPGEAVTYVDAHDNEILYDAL

AYKL--------P---------------QATTPTDRARMQVLALSTVVLGQGIGFVTAGT

ERLRSKSLDRNSYNSGDWFNQIRWDC--------DS-GNGFG-AGLPPAADN----QDKW

QYARPLLA-D-PA-LV--PDCAAIGLAEARYAELLRIRASSPLFSLGTAAEVQKKLTFPL

S--G-PL--EVPGVITMQLG---G--------------GAVTVLFNATPQAVRQPVPAAR

--------GARLALHPVLRQ--S-A--DPV-LRTASFDSAT--GTFTVPARSVAVFVK--

-------

>UniRef90_UPI0017821BA3_1272_2147 | pullulanase-type alpha-1,6-glucosidase n=1 Tax=Cellulomonas sp. JH27-2 TaxID=2774139 RepID=UP | E_val=4.4e-209

-----------------QRAHWVSRDTLAWP-RDLLP----AGADPAD-LSWELETASSG

G-LALADG---EVT---GG-Q------AHELTYD-SVG--LAQAVTDRYPALK--GYVAL

HLSAAD------AADAA-----DLLTGQLAVLQRT----------------GDD-ASAWT

GVQIPGVVDDLYAS----AK---DADLGL-------TW----D-D----------A----

-----------TPTFALWAPTA-KHVDLLVQAGSTT------------AEPQR-VAAT-R

SD---DGVWRV------RG-EA-G-----WK--NAAYRYDVQVYVP-----STDEVEHNV

VTDPYSVALTTN-----STHSVVVDL---DDPSTQPSLWADT--PAP-TI-EKAVDRSIY

ELHVRDFSIKDLTVPADERGTYLAFAR-D---------SAGTKHLRALAAAGLNTVHLLP

TFDIAS--IQEDRT---------------KQATPDC------------------------

---DLASYG---PASSEQQACVAKTADTDGFNWGYDPYHYLAPEGSYATDP---DGAARV

GEFRTMVGALHATGLQVVLDEVFNHTAASGQ-ADTSVLDKVVPGYYHRLN-AVGAVETST

C--CQNVATEHALAGKLMVDSVVLWAKEYKVDGFRFDLMGHASKQNLLDVRAALDKLTLA

KDGVDGKKTYLYGEGWNFG-EVADDALFT----QARQGNLG-GTGIGTFSDRLRDAVRGG

GPFDEDP----RVQGFGSGVYTDPNGAE--------ANGTAD-DQLATAQHDADLVRLGL

AGNLRDYAFTT-S-DGR-TTKGSEI-DY-NG-QPAGYADEPDEVITYVDAHDNETLWDSL

TMKL--------P---------------VATTMSDRIRMNTLSLATTALAQTPSFWNAGA

DMLRSKSLDRNSYNSGDWFNAIDWTG---------A-DNGFG-HGLPQAADN----QAKW

DYQRPLLE-N-PA-LK--PTAAQVATASDQAQDLLRLRTSSRLFRLGTAAAIEQKVTFPG

S--G-SD--AVPGVITMSIDDTVG------A-DADPALDGALVVFNAGTKAVTQKISALA

--------GRDYALSSVQAH--G-A--DEV-VKATTWDTAT--GTVTVPARTVAVLVDAQ

-------

>UniRef90_A0A4Y8TZC7_167_1040 | Pullulanase-type alpha-1,6-glucosidase n=1 Tax=Glutamicibacter arilaitensis TaxID=256701 RepID=A | E_val=1.3e-208

----------------QSKAQWISVDTIAIP-RDL----------GKG-EKYALFASEDA

G-MSLEGE---QIA---GA-K------PVELKPV-NGG--LTEKQRQDFPHLA--NYRAM

HVDMS-------RAEAA-----AALRSQLAFARFD-----DQGE-------APK-LTAYT

GVQIPGAIDELYARA---LE---DVELGV-------SF----T-G----------K----

-----------KPTFRLWAPTA-RHAELLITGGN--------------GKSSR-HAARYD

SN---SGVWTV------QG-KP-Q-----FA--GAEYRWEVQVFVP-----ETGKIETNT

VTDPYSVALSTN-----STQSVAVNL---EDPKHQPKQWRKA--KAQ-IV-EKDVDRSIY

ELHIRDFSISDSSVPEDLRGRYGAFGV-D---------GAGNKQLKELADAGLTTVHLLP

SFDIAS--IQEDEM---------------QRVETDC------------------------

---DLESMP---AASSKQQECVSAHVDEDGFNWGYDPFHFMAPEGSYATNP---EGAQRV

EEFRGMVGSLHNMGLEVVLDQVYNHTAASGQ-ADQSVLDQIVPGYYHRLD-ATGKVETST

C--CQNIATENAAAQKLMVDAVVTWARDYKVDGFRFDLMGHHSRETMEAVRSALDELEPG

RDGVDGKSVYLYGEGWNFG-EVADNARFY----QATQGQLN-GTGIGTFNDRLRDAVHGG

SPVDSSST---FRQGFGTGLGTDPNGNE--------VNGTTD-EALADLRQETDLVRLGL

AGNLADFEFMA-S-DGQ-VHRGDEL-DY-RG-AAAGYASQPGEVINYVDAHDNETLYDLT

VLKL--------P---------------RDTSMEQRVRMNTLSQATVMYSQAVPFWHAGT

ELLRSKSLDRNSYNSGDWFNRIDFTG---------Q-ENTFG-AGLPPKAGN----GEKW

DLMAPLLQ-D-PE-LK--PNAQAMAQAEGAALDLLRTRKEVGLLRLGTAELIEQKVSFPL

S--G-PQ--AQPGIIVMHIDDTVG------A-DADSKHDGALVLFNASPREHTQVLEELV

--------GRDYQLATALEE--G-S--DEV-VKSTLFDSAT--GELRIPARTAAVLVEA-

-------

>UniRef90_A0A5D0PIU8_1077_1930 | Alpha-dextrin endo-1,6-alpha-glucosidase n=9 Tax=Microbispora TaxID=2005 RepID=A0A5D0PIU8_9ACTN | E_val=3.8e-208

-------------DLSKATAQWIDRDTVAWK-GEV----------ATA-NHYALAYSPKG

D-IAYDKG---DLT---GD------VHLIRL--A-PGQ--LSDAQKAAWPHLA--AYGAL

KVDPRD------AGLVR-----DALRGQVVAVERG-----P----------SGA-LLTAT

GVQIPGVLDDLYAA----AA---RAPLGP-------T------------------G----

-----------GGALSVWAPTA-RTVELALYDGPSA------------GHRTV-HPMRRD

DA---TGVWSV------SG-PP-S-----WR--GRYYTYLVTVYAP-----AAGKIVTNE

VTDPYSLSVSAG-----SGRSQIVDL---DDRALRPDGWTAL--RKP-PA-VRQDRASIY

ELHVRDFSASDATVPAAERGTYKAFTETG---------SAGMTELRSLAKDGLTHVHLLP

VFDFAT--VPDLAA---------------DRAEPGC------------------------

---DLAALP---PDSDRQQACVAETAAKDSFNWGYDPLHYTVPEGSYATHP---D--RRI

REFREMVAGLNGAGLRVVMDVVYNHTHAAGQ-DPGSVLDRVVPGYYQRLM-DDGTVATST

C--CANTAPEHAMMGRLVVDSIVTWARQYKVDGFRFDLMGHHPKANILAVRKALDALTPD

RDGVDGRSIILYGEGWDFG-EVAGGARFE----QATQAAMA-GTGIGTFNDRLRDAVRGG

SPFDADP----GVQGFGSGLADRPGD---------------------RLAHYEDLIRLGL

AGNLRDFTFTG-S-SGT-PVKGSQI-DY-NG-GPAGYAATPGDAVTYVDAHDNETLFDAL

AYKL--------P---------------RDTPMADRVRMQTLSLATALLGQGTAFVHAGS

ERLRSKSLDRNSYDSGDWFNRLLWDC--------ER-GNGFG-AGLPPAKDN----EARW

PYARPLLA-D-PA-LR--PDCAAIGAARAGFGELLRIRYSSPAFSLGSAAEIARRVTFPP

S--G------TPGVIVMRID---A------A-GADPAVKALYVVFNATGQAAAPAVPGVR

--------GARVALHPVQAA--S-A--DPV-VRQAAADPAS--GVLRVPARTVAVFTEAS

-------

>UniRef90_A0A2V4PZJ3_1032_1905 | Pullulanase-type alpha-1,6-glucosidase n=1 Tax=Microbacterium esteraromaticum TaxID=57043 RepID= | E_val=1.5e-207

-----------------QRAQWIDERTIAWP-AKL--------GSPDR-AAYQLYASADA

S-VKVADG---DVT---GA-E------PIALNVI-DGG--LTDAQKKRFPALA--GYLAL

QVPDAD---------AA-----TLLKGQLAVAQRD-----T----------SGA-LTAFT

GVQIAGVLDDLYADE---VD---DQRLGV-------TF----K-G----------V----

-----------NPTFRLWAPTA-QSATLLTWDAGAGA----------DVQPTR-HTAVYD

RA---SGVWAV------KG-KK-A-----LK--NDEYLWEVVVYAP-----TTGGIETNR

VTDPYSVALTEN-----SERSVAVDL---TDKAFQPKDWRKT--KAP-VV-DRPVDRSIY

ELHIRDFSIGDKSVPEAERGTYLAFTR-D---------GAGAKQLRQLADAGINTVHLLP

SFDIAT--IEENRK---------------KQAQPDC------------------------

---DLRSYG---PADTAQQECVAAIAAEDGFNWGYDPYHYSTPEGSYAVNP---DGGARV

AEFRSMVGALHGMGLQVVLDQVFNHTAESGQ-ADRSVLDRVVPGYFHRLN-AAGAVETST

C--CENVATEHSVAQKLMVDSVVTWARDYKVDGFRFDLMGHHSTANMRAVRKALDALTVK

EDGVDGKSILLYGEGWNFG-EVADNALFE----QATQGQLG-GTGIGTFNDRLRDAVHGG

SPVDGSST---FRQGFGTGLGTDPNGNA--------INGSTE-QALADLGHETDLVKLGL

VGNLRDYSFVT-S-DGK-TTAGDDI-DY-RG-ARAGYADQPDEVINYVDAHDNETLYDLS

VLKL--------P---------------VDTSMADRVRMNTLSLATITLSQAPSFWHAGT

ELLRSKSLDRNSYDSGDWFNRIDWTG---------K-ESTFG-SGLPMAADN----EEKW

GIMRPLLE-N-AA-LK--PKPADIAAAEASALDLLRVRSEVDLLRLGSAKLIEQKVTFPN

G--G-AD--AAPGVIVMQIDDLVG------A-DVDPALDGALVVFNASPESVTQTVDGLA

--------GRSFALTRAQAK--G-A--DAV-VKKTTWDART--GTVTLPARTVAVLVDA-

-------

>UniRef90_D2PZ46_1042_1905 | Alpha-1,6-glucosidase, pullulanase-type n=4 Tax=Kribbella TaxID=182639 RepID=D2PZ46_KRIFD | E_val=3.3e-207

-------------DLEQAKAHWLGRDLLAVP----------PVPHPER-SRWRLHWSPTG

S-LAIDAD---DI----GG-S------SAGLRVD-PNG--LPAAVLAKFPALK--GYTAL

RLGRVD---------AE-----SMLTGQLGLAQYD-----D----------AGR-LLDAT

GVQIPGVLDDLYGAA---AT---GRSYGV-------TW----H-G----------G----

-----------VPRFTLWAPTA-QKVALLV-------------------GAQR-IPMR-R

TS---DGSWIA------QG-RR-S-----WQ--NAPYRYEVTVFAP-----STGKVETNL

VTDPYSVALTTD-----STHSVAVDL---DDPAGKPALWART--PAP-KL-ARPVDSTIY

ELHVRDFSIDDPTVPAAHRGSYLAFAD-E---------GAGTKHLRKLAQAGLNTVHLLP

TFDIAS--IPE--G---------------TQEKPAC------------------------

---DLKALP---PDSEQQQACVTAVAAKDGFNWGYDPYHWLAPEGSYAVRK---DGLSRV

AEFRTMVGGLHRSGLRVVLDQVFNHTPAAGQ-APTSVLDKVVPGYYQRLN-ATGGVETST

C--CSNIATEHAMAEKIMVDGTVSWARNYRVDGFRFDLMGHHSKANMLKVRAALDRLTLA

KDGVDGRQVFLYGEGWNFG-EVANDALFE----QARQGNLG-GTGIATFSDRLRDAVRGG

GPFDEDP----RVQGFGSGAASDPNGAP--------VNGSPA-DRAKRLAHDTDLTQLGL

AGNLRAFTFRSAE-TGA-VVRGDAV-DY-NG-APAGYADQPGEVITYVDAHDNETLWDAL

TYKL--------P---------------TGLPMADRVRMNTLSLATTALAQTPSFWHAGA

DLLRSKSLDRNSYDSGDWFNTLDWTG---------A-DNGFG-HGLPPKPDN----EAKW

PFMKPLLG-N-PA-LK--PSAADVGTASAAAADLLKLRFSTPLFRLGSADLVNQKVGFPL

S--GTPA--AVPGVVTMRIDDTVG------P-DVDPALKGLVVVFNSTGSTVSQQIPGLT

--------GATLSLSPVQAG--G-A--DPV-VKQSTWTAAS--GTVSVPARTVAVLIQ--

-------

>UniRef90_A0A4Q8A8I2_967_1834 | Alpha-dextrin endo-1,6-alpha-glucosidase n=1 Tax=Micromonospora sp. CNZ295 TaxID=2512146 RepID=A | E_val=1.1e-206

----------------KRKAHWIDRSTVAWA-TGP----------TDG-KTYALVAAPAG

G-VTVADG---ELT---GT------YTTIPLRAQ-RNG--LTEAQRDRFPHLW--AYQAF

TLDRAQ------LAKVP-----AALRGQLVVTERD-----A----------QGT-LLAAT

GVQIPGVLDDVYSR----AT---QARLGP-------TF----T-G----------G----

-----------VPALAVWAPTA-RSVSLQLFDSPA-------------AQPET-VSMRRD

DR---TGAWSV------RG-SR-S-----WI--GKYYRYRVEAWQP-----AAQKMVTAS

VTDPYSLALAPD-----STHSRIVDL---ADPRLAPKGWGDL--RKP-AA-VPAAKVQIS

ELSVRDFSIADTTVPAERRGTFLAFTDPG---------TAGMKHLRGIADAGVTHLHLLP

AFDFAT--IPERRA---------------DQRQPAC------------------------

---DLAGLP---PDSDQQQRCVAEVADTDGYNWGYDPLHYTVPEGGYAVDP-----ANRT

TEFRQLVAGVNGAGLRVVLDVVYNHTSAAGT-DPKSVLDQIVPGYYHRLL-EDGTVANST

C--CANTAPEHAMMGKLVVDSVVTWAKAYKVDGFRFDLMGHHPKANMLAVRAALDRLTLG

RDGVDGRKILLYGEGWNFG-EVANDARFV----QATQANMA-GTGIGTFNDRLRDAVRGG

GPFDANP----RQQGFASGLFTDPNGDP--------VNGSAA-AQRARLLHQHDLIKVGL

TGNLRGYRFTN-S-AGE-QVTGAEV-DY-NG-APAGYTAAPGEAVTYVDAHDNEILYDAL

AYKL--------P---------------QDTAAVDRARMQVLALGTVLLGQGTGFVTTGS

ERLRSKSLDRNSYNSGDWFNQIRWDC--------AQ-GNGFG-VGLPPEQDN----RDKW

PYAKPLLA-D-PR-LV--PDCAAIDLTDARYAELLRIRASSPVFGLATAEQVQKRVAFPL

S--G-EA--ETPGVLTMTLD---G------R-GLDGRWTSLTVIFNATTGTATQRLAGLR

--------GADVALHPVLRD--S-A--DPA-LRTASFATSS--GTFTVPARSVAVFVQ--

-------

>UniRef90_A0A0W8IHH0_1040_1907 | Alpha-1,6-glucosidase n=5 Tax=Serinicoccus TaxID=265976 RepID=A0A0W8IHH0_9MICO | E_val=3.8e-206

--------------LGSQRAYWVRDDLVAWP-AEGV-------GDAVL-RDWRLHWSADG

G-LGIDAE---AVT---GG-D------SSPLTLD-PDG--LPEEVLEDFPHLE--GYLAL

RLDRRS------ARAAG-----DILTGQVAVAMYD-----D----------LGR-LADAT

GVQVPGVLDDLYAER---AA---DTALGV-------SW----R-G----------P----

-----------NPTLRLWAPTA-QDVRLLTWPEGGQ------------GDPQE-SSLR-R

LK---DGSWEI------KG-KR-S-----WT--GREYLFEVDVFVP-----ETGAVETNR

VTDPYSVALTLD-----SERSVLVDL---DDRQWQPRVWQRA--EAP-AL-EDEVDQTIY

ELHVRDFSMADPDVPAELRGSYLAFAE-D---------GYGRRHLEALAEAGMNTVHLLP

TFDIAS--IPETTAD------------AEAVPQPDC------------------------

---DLDSLP---PDSAEQQECVMAQADADAFNWGYDPWHFMAPEGSYASTTQAAQGGARV

AEFRTMVGGLHESGLRVVLDKVFNHTAQSGQ-GSKSVLDKVVPGYYHRLN-AVGDVETST

C--CENIATEHAMAEKLMVDAVVVWARDYKVDGFRFDLMGHHSRDNMQAVRAALDELTVR

RDGVDGSQVTLYGEGWNFG-EVADNARFY----QATQGQLD-GTSIGTFNDRLRDGVRGG

GPFDEDPG---AEKGFATGGTS---------------------------GNDTDLVQVGL

AGNLRGFELRSQE-TGE-VVTGEQV-DY-NG-SAAGYAEDPDEVVNYVDAHDNETIFDAL

TFKL--------P---------------QDLPMADRVRMNTLALSTTTLSQSISFWHGGA

DLLRSKSLDRNSYNSGDWFNLLDFTM---------T-ENGFG-RGLPPAADN----EDKW

DLMRPLLA-D-PS-LA--PEPADIEQASEMAADLLRLRFSSDLFRLADPELVQEKVSFPV

S--GTAD--GDAQVVVMRVDDTVG------A-DVDPGLDGLVVVFNASGEQVDQVVPGLE

--------GEQLELSPVQAE--G-A--DEV-VREATWEAAT--GTATVPARTVAVFVQP-

-------

>UniRef90_K6UKS6_45_936 | Putative glycosidase n=1 Tax=Austwickia chelonae NBRC 105200 TaxID=1184607 RepID=K6UKS6_9MICO | E_val=1.2e-205

-------------DLSTAKAVWVAPDLLAWP-AKALP----AGHTPAK-LTWRLHSSPRG

G-ITRTGN---TLN---AP-A------SYDLTWQ-KTG--LPASVLTTHPHLK--DYLAL

KAPAAL------ATQAG-----NILKGQIAVTLHD-----D----------RSR-LHTAT

GAQVAPVLDTLYARR---AV---QNSYGV-------TW----S-A----------G----

-----------RPTLRLWAPTA-HKVTLLTWPAGSAADAPT-------ATATR-TAMT-P

DS---DGSWAA------RG-PV-S-----WK--NARYLYEIDVLDP-----ATGKIRTNQ

VTDPYSTALTPD-----STRSVVVDL---TDPALAPAQWKQA--RPP-KL-TRSVDQSIY

ELHIRDFSASDAKVPPNHRGTYLAFAD-Q---------GHGTRHLRALASAGLNTVHLLP

SFDITS--VPENKS---------------AQQRPPC------------------------

---DLRSYP---ADSEEQQRCLQKTAAKDAFNWGYDPYHFLAPEGSYASSTQNADGAQRI

REFRTMVGGLHASGLRVVLDQVYNHTAAHGQ-AAPSVLDRVVPGYYHRLD-ATGKITTST

C--CSNTATEHAMMNKLMVDATVSWAKHYKVDGFRFDLMGHHSVSTMKAVRTALNRLTPA

ADGIDGRAITLYGEGWNFG-EVADNARFV----QASQGQLG-GTDIATFNDRLRDAVRGG

GPFDADP----RKQGFATGLADAPNGSP--------ANGDPA-AQTARLRHETDLIQLGL

AGNLRGYTFRSQA-SGR-PTAGAKI-DY-NG-KPAGYADQPGETVTYVDAHDNETLFDAL

TLKL--------H---------------PATTMSDRIRMNTLALSTTAFSQGISFWHAGA

DLLRSKSLDRNSYNSGDWFNLLDFSM---------Q-DNGFG-RGLPPAGDN----KAKW

PYQKPLLA-D-PR-LK--PAPQDIRAAAAAAQDLLRLRHSTPLFRLGSADAVKAKLSFPA

S--GTAS--AVPGVIAMRLDDTRG------A-PVDPRLRGIVVVFNSTPAPVRQAVPGMA

--------GQAASLSPIQAA--G-T--DPV-VRKATWDRAA--GAAHVPARTVAVFVQTR

-------

>UniRef90_A0A1H1V0M6_1045_1948 | Alpha-1,6-glucosidases, pullulanase-type n=2 Tax=Paraoerskovia marina TaxID=545619 RepID=A0A1H1V | E_val=5e-205

----------------EERAHWLDADLLAWP-RDLL-----GDSDTED-LTWTLHHAPDG

G-IRVEDG---VVVEPDDA-G------SLNLAVD-PDG--IPAEVAETFPALA--DAVAL

RLRDENGDAP-ARTAVE-----SVLQGQVQVAQHH----------------GDE-LTAFT

GVQLPGVLDALYADA---AG---DQDLGV-------EFAP--N-G----------K----

-----------WASFRLWAPTA-RSVTLLVWPGGSDGA----------GDARR-VEAV-R

SG---DGTWAV------DG-KA-VDKKFRWS--GAQYLYEVEVYVP-----GTGAVETNV

VTDPYSVGLTLN-----SERSVAVDL---TASSLAPKAWRTT--AAP-VV-ERPVDRSIY

ELHVRDFSIDDETVPADRRGTYLAFAESG---------SDGMTHLRELADAGMNTVHLLP

TFDIAT--IEEDRS---------------AQADPG-------------------------

---DLSGFA---PDSAEQQAAVAAVQGEDGFNWGYDPYHFSTPEGSYATEP---DGGARV

AEFRTMVGALHGAGYQVVLDQVFNHTAASGQ-DAKSVLDKVVPGYYHRLD-GEGDVETST

C--CQNLATEHRMTEKLMVDSVVTWARDYRVDGFRFDLMGHHSVENMQAVRDALDALTLR

KDGVDGSAVYLYGEGWNFG-EVADDALFT----QARQGNLG-GTGIGTFSDRLRDAVHGG

SPVDGAST---FTQGFGTGLGTDPNGRPARAGDSSTVNDGSA-DELADLAHQTDLVRLGL

AGNLADFTFTT-S-AGT-EQRGDEI-DY-RG-SPAGYAAQPGEVVTYVDAHDNETLFDLL

ALKL--------P---------------QETSMDDRVRMNTLSLATTALAQTPSFWHAGT

DLLRSKSLDRNSYDSGDWFNAIDWTG---------T-DNGFG-RGLPLAADN----EEKW

PVMAPLLA-D-EA-LA--PTADEIGSATAQAQDLLRLRYSSDLFRLGSAEKIEQKVTFPG

S--G-PD--APAGTLVMSVDDTVG------E-DVDPALDGLLVAFNASPDEVTIPLADLA

--------GREYSLSEIQAD--G-S--DPV-VRGTTWDEDA--GTVTVPARTVAVLVDEQ

S------

>UniRef90_A0A1S9AEN9_172_1036 | Alpha-dextrin endo-1,6-alpha-glucosidase n=1 Tax=Massilia sp. KIM TaxID=1955422 RepID=A0A1S9AEN9 | E_val=3.7e-204

--------------LALAGAHWLSARHLAWP-GA-----------PSS-GSYRLYYAKEG

G-LGAAAE---GVT---GA-D-----GSFALQ---PAS--LPDGVRQKFPHLA--GATGL

ALSTQD------AAAAA-----QLASAQFAIAQFG-----P----------DGR-LVQVT

SLQTAGMLDEVFASK---AA---SAQLGL-------SF----D-RA---------G----

-----------RPTFRVWAPTA-RSVALDLYPNADA------------PATSQ-VPMVRD

D----SGVWSY------TAPDG-S-----ITN-RAWYTYTVKVLSR----WANNAVVTNT

VTDPYSVSLNAN-----GRRSFVADL---DSQALKPAGWDHQ--RLP-KL-EHPADISLY

ELHVRDFSASDATVQAKHRGKYLAFTERD---------SNGMRHLKSLQKAGLTHVHLLP

VFDIAS--VDETGC-----------------VTP--------------------------

---TIPNAG---PDSEAQQAAVDAVRDSDCFNWGYDPVHYNAPDGSYATNA--ADGASRV

REFRAMVQSLHETGLRVTMDVVFNHTSGSQQ-GPTSVLDKIVPAYYYRLN-DKGAILNDS

C--CADTAAEHTMMGKLMIDSVALWARHYQVDSFRFDIMGMAPLELMKRLQQEVNRAA--

-----GREIYLYGEAWNFG-AVGNDARSV----QARQANMY-GSGIGSFNDRIRDAVRGG

GCCDGGIN-LVSQQGFINGAWLDPNAQA--------------NQSKDDLLRLADLVRVGL

SGTLRDYRFTD-R-SGA-LRSNAEI-DY-FG-QRAGFAANPAEVINYIEAHDNQTLFDIN

ALRL--------P---------------QNTPLAERVRVQNLGAAIVLLSQGLPFIHAGQ

EILRSKSLDRDSYNAGDWFNRLDYSY---------A-SNNFG-VGLPMAGVN----RDNW

SIMQPVLA-N-PL-IK--PDSRAIMSAREVFEEYLAIRNDSALFRLRSAQDVQQRLRFFN

T--G-PA--QVPGVIVMRIDGEQP-----SR-YRDAHYRSVVVVFNVDKAAKTVAVPELR

--------GQKLSLHKLQRK--S-A--DAA-LRGASYDPAS--GSFSVPARSAVVFVD--

-------

>UniRef90_A0A542ZMF1_1041_1926 | Pullulanase-type alpha-1,6-glucosidase n=1 Tax=Oryzihumus leptocrescens TaxID=297536 RepID=A0A54 | E_val=1.4e-203

-------------DLKKAKAYWVSRDLLAWP-ASGVP----AGSDPAL-LHWRLHWSPTG

D-LGVDAE---AVT---GG-S------TADLTYD-ASG--LPASVTAAHPELK--GYLAL

RLDPKV------ARQAE-----DILRGQVAVGLYD-----E----------LGR-LLDAT

GVQTPGVLDDLYAPA---AS---RRSMGV-------TW----D-G----------R----

-----------TPSFALWAPTA-RSVDLLTWPAGSAADAPV-------SAATR-TAMR-R

QG---DGSWTA------AG-RP-Q-----WR--GARYLYDVEVFAP-----TTGRVEHNL

VTDPYSVALTLD-----STRSVAVDL---ADPAYRPALWSGT--PSP-TL-KQDVDSTIY

ELHVRDFSIGDTSVPADHRGSYLAFAD-D---------GNGTKHLKALAAAGLNTVHLLP

TFDIAS--IEEDPA---------------KQAKPAC------------------------

---DLASYG---PASEEQQKCVTAVAGKDAFNWGYDPWHWMAPEGSYASSAAAADGGQRV

AQFRTMVGGLHQDGLRVVLDQVFNHTPASGE-APTSVLDQVVPGYYQRLN-ATGAVETST

C--CQNVATEHAMAQKVMVDAVVSWARSYHVDGFRFDLMGHHSRDTMLAVRHALDALTLQ

HDGVDGRSIYLYGEGWNFG-EVANNALFT----QATQGQLG-GTHIGTFSDRLRDAVRGG

GPFDEDP----RKQGFGSGEATDPNGAA--------VNDGA----AGSLQHDTDLVELGL

AGNLRAFTFTD-T-EGK-VVRGDQV-DY-NG-SPAGYADQPDEVVSYVDAHDNETLWDSL

TYKL--------P---------------VATSMADRVRMNTLSLATTALSQTPSFWHAGA

DLLRSKSFDRDSYDSSDWFNTLDWTG---------A-DNGFG-HGLPREGSN----SAKW

PYMKPLLA-N-PA-LK--PSATDVATASAQAQDLLRLRFSSPLLRLGSADAIRAKVGFPV

S--GTGD--AHPGVVVMRIDDTVG------A-DVDPALKGLVVVFNASPSAVAQKLPGMT

--------GADLALSPVEAG--G-A--DPV-VKTSRWDASS--STLTVPARSVAVFVQ--

-------

>UniRef90_A0A5B8IP16_165_1040 | Pullulanase-type alpha-1,6-glucosidase n=5 Tax=Glutamicibacter TaxID=1742989 RepID=A0A5B8IP16_9M | E_val=3.7e-203

--------------VGQSKAQWISTDTIAIP-RDL----------GSG-QRFALYASKDA

S-LRLDGE---VIS---GV-K------PIELATV-IGG--LTDAQRKKFPHLA--NYRAV

RVDMT-------REETA-----TALRSQLALARFE-----TQGE-------DSK-LTAFT

GVQTAGAIDELYAQG---LE---NVELGV-------SF----T-G----------K----

-----------KPTFRLWAPSA-HRATLLITGKN--------------EKTSR-HDATFD

KN---SGAWTV------MG-RP-Q-----FT--EAKYLWEVQVFVP-----GTGKIETNI

VTDPYSVALTTN-----STQSVAVNL---EDPKHQPKQWRKA--KAA-GV-EKDVDRSIY

ELQIRDFSVSDFSVPEEQRGRYGAFGT-N---------GAGSKQLKQLAEAGLTTVHLLP

SFDIAS--IEEDEN---------------QRIETGC------------------------

---DLESLP---AASPKQQECVSAHAADDGFNWGYDPFHFMAPEGSYASNP---DGAERV

EEFRGMVGSLHEMGLEVVLDQVFNHTAASGQ-ADQSVLDKIVPGYFHRLD-ATGQVETST

C--CQNLATEHAAAQKLMIDSLVVWAKDYKVDGFRFDLMGHHSRETMEAARSALDELEPG

RDGVDGESIYLYGEGWNFG-EVADNARFY----QATQGQLN-GTGIGTFNDRLRDAIHGG

SPVDSSST---FQQGFGTGLGTDPNGHA--------VNGTAE-EALKDLGHETDLVRLGL

VGNLAGYEFLT-S-EGQ-MQRGDEL-DY-RG-AAAGYASQPGEVINYVDAHDNETLYDLT

VLKL--------P---------------RGTSMEDRVRMNTLSQAAVMYSQAVPFWHAGT

ELLRSKSLDRNSYNSGDWFNRIDFSG---------Q-ENTFG-SGLPPEADN----KEKW

DLMAPLLE-D-PE-LK--PDAQAMSRAEGAALDLLRTRRDVGLLRLGSAELIEQKVSFPL

S--G-PK--AEPGIIVMRIDDRAG------E-DADSEHDGALVVFNASPQDHTQVVEELA

--------GHEYDLAEALEQ--G-S--DKR-VKSTSFDSSS--GKLMIPARTAAVLVEA-

-------

>UniRef90_UPI0012FAC5DD_1069_1920 | pullulanase-type alpha-1,6-glucosidase n=1 Tax=Agromyces salentinus TaxID=269421 RepID=UPI001 | E_val=8.5e-203

-----------------------------------------------D-LAYTLEHSPEA

T-LGVEDG---EVT--GGG-D------AVELAFD-PSG--LSEAQAAKFPHLA--EYTVL

RPEGLD------RAEVA-----DLLTQQLQVAQR------L----------DGS-LTALT

GVQVPGVLDDLYADA---VA---SVPLGV-------GW----S-G----------D----

-----------EATATLWAPTA-QAVSLERWDAGAS------------ADPEV-VEASFD

DA---NGTWSV------DG-LA-A---------GDEYRWSVRVYAP-----TTGAIETNS

VTDPYSVALTTN-----STRTVVVDL---DDASLAPEQWAET--PAP-VV-ERPVDRAIY

ELHVRDFSITDETVPEAERGTYRAFTR-D---------SAGTAQLEQLADAGINTVHLLP

TFDIAT--IEEDRA---------------AQATPAC------------------------

---DLAAYG---PASEEQQACIDDVRDLDGFNWGYDPFHFQAPEGSYAVDP---NGGARV

GEFREMVGALHGMGLQVVLDEVYNHTAASGQ-GAKSVLDRIVPGYYQRLN-AAGNVETST

C--CQNVATEHEVAEKLMVDSVVLWAKEYKVDGFRFDLMGHHSKANMLAVRASLDELTLA

EDGIDGKAVYLYGEGWNFG-EVADNALFE----QATQGQLG-GTGIGSFNDRLRDGVHGG

SPVVGESL---FEQGFGTGLAGDPNGRP--------LT-RDE-SEIRTLGQQTDLVKVGL

AGNLRDFEFTG-Y-DGV-VKRGAEV-DY-NG-APAGYADQPDEVINYVDAHDNETLYDLG

VLKL--------P---------------VDLPMADRVRMNTLSLATVTLSQTPSFWHAGT

ELLRSKSLDRNSYNSGDWFNRIDWTG---------Q-GSTFG-SGLPGEADN----GDNW

AVMRPLLE-N-PA-LK--PGAADIAAAEASALDLLRVRNEVDLLQLGSAELIDQKVSFPN

S--G-SD--ATPGLIVMEIDDLVG------E-DADPELDGALVVFNASPEAITEQVDGLA

--------GRDFALTDAQAN--G-S--DAV-VKATAWDAAS--GTVTVPARSVAVLVDEQ

-------

>UniRef90_A0A1Q5D2G4_952_1801 | Alpha-dextrin endo-1,6-alpha-glucosidase n=35 Tax=Streptomyces TaxID=1883 RepID=A0A1Q5D2G4_9ACTN | E_val=1.6e-202

-----------------AKAVWIDRNTVAWN-G-S----------AGA-ASTQLLASRTG

G-IKAEDG---TLT---AD-D---GTSWLRL--T-KTA--LTDAQKAEFPHLR--EYTAW

SVDPRD------RDRVR-----TALRGQVVATQRA-----A----------NGA-VLAAT

GVQIAGVLDDLYAAE---AT---KAGLGP-------VF----R-H----------G----

-----------RPTLSLWAPTA-QDVRLEI-------------------GDRT-VRMRRD

DA---TGVWSV------RG-PR-S-----WK--GESYRYAVKVWAP-----EAGKVVTNK

VTDPYSLALTTD-----SERSLVVDL---DDRRLAPRGWHSY--DKP-EA-VPLRDAQIQ

ELHIRDFSVADRTADPDHRGTYLAFTDDG---------SDGSKHLRELAEAGTSYVHLLP

AFDIAT--IPEKKS---------------DQATVDC------------------------

---DLAALP---ADSDKQQECVAKAAAKDAYNWGYDPYHYTVPEGSYATDP---DGTART

VEFREMVKALNEDGLRVVMDVVYNHTAASGQ-ARTSVLDRIVPGYYQRLL-PDGSVANST

C--CANTATENAMMGKLVVDSVVTWAKEYKVDGFRFDLMGHHPKENILAVREALDALTLK

KDGVDGKKIILYGEGWNFG-EVADDARFT----QATQKNMA-GTGIATFSDRARDAVRGG

GPFDEDP----GVQGFASGLYTEPNGSE--------ANGTEA-EQKARLLHYQDLIKVGL

TGNLADYTFTD-T-DGK-EVKGSEV-DY-NG-APAGYAEAPGDALAYADAHDNESLFDAL

AFKL--------P---------------TSVNADDRARMQVLAMATAALSQGPALSQAGT

DLLRSKSLDRNSYDSGDWFNAIHWNC--------AD-GNGFG-RGLPPAADN----QDKW

PYAKPLLG----S-VQ--VGCPQITGASAAYRDLLKIRTGERDFSLATAEQVQSRLTFPL

S--G-KH--ETPGVITMRL-------------------GDLVVVFNATPEAQEQRIEAAA

--------GTGYRLHPVQAA--G-A--DPV-VKKSAYAAKT--GTFTVPARTVSVF----

-------

>UniRef90_A0A0A0B6H1_1046_1934 | Alpha-1,6-glucosidase n=3 Tax=Cellulomonas cellasea TaxID=43670 RepID=A0A0A0B6H1_9CELL | E_val=5.2e-202

------------------RAHWVDETTLAWP-AALV-----GDADPAD-LVFALHGSPDG

A-VEVADG---EVT---GG-E------TFPLTVR-DGG--LTEEQLTRFPALG--GYVAL

GLEDVD------RAQVE-----ALLRGELLVSQRAAG---D----------EGA-LQAAT

GVQIPGVLDDLYADA---AD---DGAFGA-------TW----R-R----------G----

-----------VPTLRLWAPTA-QDVELLLWPATKGSQGPGSGKPDLGAEPTR-VAME-R

GD---DGSWTV------RG-AK-S-----WR--GAAYRYAVTVYVP-----STDAVEVNE

VTDPSSVALTLN-----STHSVLVDL---ADGAHRPTQWEKA--KQP-VV--RPVDQTIY

ELHVRDFSISDETVPAEKRGTYSAFAV-E---------STGREHLRDLADAGLTTVHLLP

TFDIAT--IEEDRA---------------EQLVPQC------------------------

---DLAASA---PDSPEQQECIEAVRAGDAFNWGYDPLHWSAPEGSYAVDA---HGGARV

AEFRTMVGALHADGLQVVLDQVFNHTAASGQ-DEKSILDRVVPGYYHRLN-AVGAVETST

C--CANVATEHAMAEKLMVDSVVTWARDYKVDGFRFDLMGHHSVENMQAVRDALDDLTVR

RDGVDGKRVYLYGEGWNFG-EVADNRLFT----QATQGQLG-GTGIGTFSDRLRDAVRGG

GPFDEDP----RVQGFGSGVATDPNGAA--------VNGTEA-EQLARAAHQGDLVRLGM

AGNLRGYAFLA-S-DGT-VRTGAEI-DY-NG-QPAGYADSPEEVVTYVDAHDNETLFDAL

TYKL--------P---------------VGTAMPDRVRMNTVSLATTALAQTPSFWHAGA

ELLRSKSLDRNSYDSGDWFNLLDLSK---------Q-DNGFG-RGLPLAADN----EAKW

PLQQELLA-N-PA-LK--PAPADIQAASTAAEELLRLRFSTDLFRLGSAAQIEKKVTFPG

S--G-PD--ADPGVVVMRIDDTVG------R-KTDRKLDGVLVVVNASDEATTQTVPELA

--------GHRLALSPVQAS--G-A--DPV-VKTTQWVAAD--GEVTVPARTVAVLVEKE

-------

>UniRef90_A0A411WX83_172_1039 | Alpha-dextrin endo-1,6-alpha-glucosidase n=1 Tax=Massilia albidiflava TaxID=321983 RepID=A0A411W | E_val=1.7e-201

-----------------AKAHWLSATTLAWP-GA-----------PAN-ATYKLFYATHG

G-LAPTPDATTGLA---GA-E-----GSLPLQ---PAA--LPEAIRQKFPHLA--GATGL

RLAAADA----TVTKLA-----SLASAQFAIAQFG-----A----------DGA-LVQVT

SLQQAGMLDDVFAAR---AS---DAQLGV-------TF----D-RQ---------G----

-----------VPTFRVWAPTA-KAVQLDVYASATA------------ASANS-VPMTRD

AA---SGVWSY------TAPNA-S-----WTN-TAYYTYTVQVLSR----WANNTLVTNT

VTDPYSLSVSAN-----SKRSFVANL---DSPRLKPAGWDHH--QIP-KL-AAATDIALY

ELHIRDFSASDDTVPAAHRGKYLAFTDTE---------AAPMRHLKSLQKAGMTHVHLLP

SYDFSS--VDETAC-----------------VTP--------------------------

---AIPGAA---ADSTAQQAAVAATRDTDCFNWGYDPVHYNTPEGSYATNA--ADGAVRV

REFRAMVQALHEAGLRVTLDVVYNHTSQSQQ-GPLSVLDRIVPAYYYRLG-AEGNILNDS

C--CADTAQENAMMAKLMIDSVALWTKQYKIDSFRFDIMGFTPLELMKRLQVAADSAA--

-----GRPIYLYGEAWNFG-AVGNDARFV----QARQANMF-GTGIGSFNDRIRDTVRGG

GCCDTGAA-LVDQQGFINGAGLDPNGQS--------------TQTRDDTLRLADLVRVAL

SGTLRDYRFVD-R-FGN-LRSNAEI-DY-FG-QRAGFAGSPAETINYVEAHDNQTLFDVN

ALKL--------P---------------QATRPADRVRVQTLGAAINILAQGVPFIHAGQ

EILRSKSLDRDSYNAGDWFNRLDYSY---------A-SNNFG-VGLPLAEKN----EESW

PLMAPVLA-N-TL-IK--PDTQAILAAKGGFEELLAIRQSSTLFRLRTAQDVIDRLKFHN

T--G-PA--QVPGVVAMSISGAEP-----RP-YPGAKYKRVVVLFNVDKVANTVTIPELQ

--------GRKLKLHDVQRF--G-S--DAV-VRASAYDPGA--GSFTIPARTTVVFVQ--

-------

>UniRef90_A0A7W7D166_917_1773 | Pullulanase-type alpha-1,6-glucosidase n=1 Tax=Actinoplanes abujensis TaxID=882441 RepID=A0A7W7D | E_val=5.5e-201

-----------------SSAVWLDRGTVAFKPAGS----------TDG-KVYDLVYAPGG

I-----TN---EVT---GD------FRSVRL--T-ARA--LTDAQRARFPHLW--QYDAF

DLPR--------GVNVK-----DVLRGQVVFTERD-----A----------TGK-LLAAT

GVQTAGVLDNVYAAA---AA---DAALGP-------V-----------------------

-----------GTTLSVWAPTA-QKVDLQLYDTAT-------------AQPAT-VAMRRD

DR---TGIWSV------RG-PA-S-----WR--GKYYTYRVTAWQP-----AANKVVTAA

VTDPYALGLSTD-----STLSRIVDL---ADPALAPTGWSAL--RKP-PA-GPSAKIQIS

ELSVRDFSMADKSVPAAQRGTYAAFTNPS---------STGMKHLATLAGAGVTHLHLLP

AFDFAT--IPEKRA---------------DQKTPAC------------------------

---DLPLLP---PDSEEQQKCVTAVAADDGYNWGYDPLHYTVPEGGYAVDP---D--ART

KEFRQMVAGVNRAGLRVVMDVVYNHTSAAGT-DPKSVLDQIVPGYYQRLL-ADGTVASST

C--CANTAPENAMMGKLVVDSIVTWAREYKVDGFRFDLMGHHPKANILAVRAALDRLSVR

KDGVDGKSINLYGEGWDFG-EVAGDARFE----QATQANMA-GTGIATFNDRLRDAVRGG

GPFDESP----RVQGFATGLFSAPNGDT--------VNGDPA-AQKARLLHYQDLIKVGL

SGNLASYRFVN-S-AGT-TVTGAQV-DY-NG-SPAGYTAAPGEAITYVDAHDNEILYDAM

AFKL--------P---------------ASTPALDRARAQSVALATTALGQGAGFVTTGS

ERLRSKSLDRNSYNSGDWFNQIIWDC--------AD-GNGFG-RGLPPAADN----ADKW

DFGRPLLA-D-PA-LV--PGCDAVALSDARYQELLKIRGSSPVFGLPTAAEVQKRLSFPL

S--G-AA--ETPGVVTMTLD---G------R-GIDRTWKSVTVVFNGTSAATTQTVKPLA

--------GKTIALHPIQRT--S-A--DPL-VRTAAFAKAT--GTFTVPARTVAVFVE--

-------

>UniRef90_A0A3R9V9U2_320_1165 | Alpha-amylase (Fragment) n=1 Tax=Streptomyces sp. WAC06614 TaxID=2487416 RepID=A0A3R9V9U2_9ACTN | E_val=1.4e-200

----------------KAQAVWLDRDTLAWN-G-P----------EAA-ASVQLLASRAG

A-ISVRDG---RLV---EG-G----AQWLRL--T-PAA--LTDAQKRKFPHLA--GHRAF

TVDPRD------RDRVR-----EALRGQLVASART-----A----------TGA-VLAAT

GVQLAGVLDDLY------AT---GAALGP-------VF----S-R----------D----

-----------RVTLSVWAPTA-QQVALEL-------------------DGRT-LPMRRD

DT---TGVWSA------TG-GR-A-----WA--GKPYRFAVTVWAP-----AVQQVVTNL

VTDPYSTALTTD-----SRRSLAVDL---AAPALQPPGWREL--SKP-AA-VPFTTAQIQ

ELHIRDFSIADPT--SRHPGQYLAFTDTD---------SAGMRHLRELAASGTSYVHLLP

AFDFGT--VPEDPE---------------DRTAPAC------------------------

---DLKVYA---PDSEQQQACVAAAAAKDGFNWGYDPLHYTVPEGSYAGDP---DGAART

VEFRRMVQSLNGAGLRTVMDVVYNHTVAAGQ-SEQSVLDRIVPGYYQRLL-PDGTVATST

C--CANTAPENAMMGRLVVDSVVTRAKQYKVDGFRFDLMGHHPKANILAVRQALDALTPQ

KDGVDGKKIILYGEGWNFG-EVADDARFV----QATQQNMA-GTGIATFSDRARDAVRGG

GPFDEDP----RVQGFATGLYTAPNGSP--------ANGGPE-QQKARLLHAQDLIKVGL

SGNLAGYRFTD-T-TGR-TVTGAQV-DY-NG-RPAGYAAAPGDALAYVDAHDNESLYDAL

TYKL--------P---------------PGTTESDKARMQVLAMATAALSQGPALSQAGT

DLLRSKSLDRNSFDSGDWFNAIHWDC--------RT-GNGFG-RGLPPAADN----RAKW

PYAKPLLT-G-PA-----PGCAEIGAASAAYRDLLRIRTTEPGFALPTAAEVQARLAFPL

S--G-TT--ETPGVITMTL-------------------DDLVIVFNATPDDRAPRVPALA

--------GQGYRLHPVQAT--G-A--DPV-VRRATYDRTS--GTFTVPARTVGVFT---

-------

>UniRef90_UPI001940C9C1_934_1788 | pullulanase-type alpha-1,6-glucosidase n=1 Tax=Actinoplanes couchii TaxID=403638 RepID=UPI001 | E_val=4.4e-200

-----------------SAAVWIDRGTIAWK-TGAS---------TDG-KVYQLAYSPTG

G-IGVADG---ALT---GT------FRTIDL--K-AAS--LSEAQRKKFPHLW--QYDAF

KLD---------RSAVT-----DILRGEVVVAELA-----A----------DGK-LIAAT

GVQLAGVLDDVYRS----AA---SATLGP-------VV----D-G----------R----

-----------KASVAVWAPTA-RDVQLEV-------------------GTAL-TPLTRD

AR---TGVWSG------RG----D-----WA--GKDYRFRVTAWQP-----ATQKIETAS

VTDPYSVALTTN-----STHSRFVSL---SDPSSVPAGWASL--KKP-AA-VPTAKIQIS

ELSVRDFSIADESVAAPARGTYRAFTDPK---------TAGMKHLTGIAAAGVTHLHLLP

AFDFAT--IPEKRS---------------DQQQPAC------------------------

---DLASLP---PDSAEQQACVAAVAATDGYNWGYDPLHYTVPEGGYAVDP-----AQRT

REFREMVAGVNKAGLRVVMDVVYNHTAAAGV-DPKSVLDQIVPGYYHRLL-EDGTVANST

C--CANTAPENTMMGKLVVDSLVTWAKAYKVDGFRFDLMGHHPKANILAVRAALDKLTVT

KDGIDGKTIFLYGEGWNFG-EVADDARFV----QATQLNMG-GTGIATFNDRLRDAVRGG

GPFDGNP----RIQGFVSGLAGAPNGDP--------VNGTAE-EQRARLLHYQDLIKVGL

TGNLAGYTFTG-S-SGT-VVKGSDV-DY-NG-SPAGYTVNPGDVITYVDAHDNEILYDSL

AYKL--------P---------------QATTAADRARAQSVALATTILGQGPGFVTAGS

ERLRSKSLDRNSYDSGDWFNQIIWNC--------AD-GNGFG-RGLPPKPDN----EAKY

PYAGPLLA-D-PA-LV--PDCAAIDQTDTSYREMLRIRKSTPAFGLATGAQVQKRVTFPL

S--G-TS--ETPGVITMTID---T------R-GVDPNWKSVTVVFNAGTTAATQKVPALA

--------GKKVALHPIQQS--S-A--DPV-TRTASFTPGT--GTLTVPPRTVAVFVQ--

-------

>UniRef90_A0A1M6A2X2_1051_1930 | Alpha-1,6-glucosidases, pullulanase-type n=1 Tax=Tessaracoccus bendigoensis DSM 12906 TaxID=1123 | E_val=7.5e-200

-------------------AHWVDASTFAWP-TNLIT--------GGG-TTWELWTDPDG

G-LALADG---VVV---GG-SGAGAVKLGDLTRD-TAG--LTAEQLADRGHLA--GFAAL

KLEAS-------RADVE-----AALKGDLAVVQRG-----A----------DGI-AQVFT

GVQIPGVLDDLFGED---AR---GEALGV-------TF----A-D----------D----

-----------VPSVRLWAPTA-TSVDLLLFDDLLGS-----------GEPTR-VAMT-R

AS---DGAWSV------DG-LP-A-----WK--NRAYQFDVNVFVP-----SLDALTHNV

VTDPYSEGLTLN-----STHSVLVDL---DDPEWAPEIWTET--PAP-TI-DQFVDRAIY

ELHIRDFSITDETVPEALRGTYEAFALKG---------TEGVKRLTELAEAGMNTVHLLP

SFDIAT--IEEDRA---------------EQKEPVV------------------------

-----PDAG---PASEEQQAAVMAVADQDGFNWGYDPFHFSTPEGSYASAA-NQNGGART

AAFREMVGGLHDIGYQVVLDQVFNHTSASGQ-GDTSVLDKVVPGYYHRLN-LRGAVETST

C--CQNIATEHAMAEQLMVDSVVTWAADYHVDGFRFDLMGHHSVDNMTAVRSALDELTMA

DDGVDGKGIYLYGEGWNFG-EVADNARFT----QATQGQLN-GTNIGAFNDRLRDAVHGG

GPFDENKS---ENQGFGTGLYTDPNGQS---------KASPA-DQLADLRHRQDLIRLGM

AGNLADYSFET-A-AGT-VQKGSEL-DY-NG-QPAGYASEPDESVNYVDAHDNETLFDLG

VWKL--------P---------------SDTSMADRIRMNTMSLATVTLGQSPTFWHAGT

DLLRSKSMDRNSYNSGDHFNALDWSM---------G-SNNFG-VGLPPAADN----SSQW

SAMRPLLT-N-TA-NL--PSASDIATSRDQALELLQLRSTHPLLTLGSAELIDQKVTFPN

A--G-AD--QTAGLIVMRIDDTVG------T-DVDGDLDGLLVVLNSSPEPITEAVDALA

--------GVDLSLSPVLTD--G-VHDDPV-LAEATWDKAS--GTVKVPARSAVVFVEE-

-------

>UniRef90_UPI000F08ECF1_153_1025 | pullulanase-type alpha-1,6-glucosidase n=2 Tax=unclassified Brachybacterium TaxID=2623841 Rep | E_val=1.6e-199

------------------------TDTIAWP-SDLV-------SDAGA-TRWELLASSGG

G-ISADGD---EAT---GG-R-----SLGELTLR-EGG--LAEDELTGRAHLS--GHLAL

DLPELE------RAELE-----KALRGQLAVAQHG-----P----------DG--LEVLT

GLQIPGVLDALYAEA---AA---EQELGV-------TW----D-D----------G----

-----------APSLALWAPTA-RSVTLQLHGPADESTAP--------SDPVRKFPMR-R

GA---DGTWSV------DG-RK-H-----WA--GQAYTFAVEVYVP-----ALDQVVTNV

VTDPYSVGLTVN-----STHSVLVDL---DDPRWAPEVWADT--PAP-EV-AQFADQTIY

EMHLRDFSAGDEGLPEGIRGSYAAVGHPD---------SAGSARLAELAEAGMTTVHLLP

TFDIAT--IEEDRS---------------AQQHPDI------------------------

---P-ADAG---PASPAQQEAVTAVADQDAYNWGYDPLHYMAPEGSYATEG-HQVGGERT

RQFRGMVGQLHSMGLQVVLDQVYNHTAAHGQ-SERSVLDRIVPGYYQRLD-LSGGVENST

C--CSNVATEHAMAQKLMVDSTVTWARDYGVDGFRFDLMGHHSRENMLAVREALDELTLE

SDGVDGKGIYLYGEGWDFG-EVADNARFT----QATQGQLG-GTSIGTFNDRLRDAVHGG

SPFDTDKR---TSQGFGNGLATMPNGFA--------EVGEA--EQLTDLRHRTDLIRIGM

AGNLADYELLG-S-TGE-VVRGDQL-DY-NG-APAGYATAPEETVNYVDAHDNEALYDMN

VWKL--------P---------------QDAPMDVRVRMNTLSLATVALGQSPSFWAGGT

DLMRSKSLDRDSYDSGDHFNAIDWSM---------Q-DNGYG-NGLPPAEKN----GEAW

ELMGPMLE-D-PA-KD--PATEDIATSSEVTLDLLRLRSSTPLFTLGDADLIRQKVTFPN

A--G-PE--ATPGLLVMRIDDTVG------P-DVDPALDGAVTVFNASDEPLTEQIEDMA

--------GLGYRLHEVQAN--G-A--DDV-VKDASWDAAT--GTVSVPAHTVAVFVA--

-------

>UniRef90_A0A367YWI1_1048_1916 | Pullulanase-type alpha-1,6-glucosidase n=2 Tax=Desertihabitans TaxID=2676837 RepID=A0A367YWI1_9A | E_val=2.8e-199

----------------QQRASWVRDDLLAWP-SDAL-----GDLPVEL-LDWRLHSAPEG

G-AGIDAE---TLS---GT-R------SVPLTRL-PDG--LPEDVLEDFPHLE--GYVAL

RLDTTD-------KAAK-----EWLTGQLAVAVYD-----D----------IGR-LQDAT

GVQVPGVLDDLYAD----AE---DADLGA-------TW----Q-GR------PGAA----

-----------RPSLRLWAPTA-KDVDLLVWPGAGAETRPV-------EEATR-VQLR-R

DP---DGTWLG------RG-TP-R-----WN--GAHYLYEVRVYVP-----QTDTVETTL

VTDPYSVALTLD-----STRSVLVDL---RDPDLAPEQWRSA--EPP-TL-GDEVDQSIY

ELHVRDFSIGDEDVPAEQRGSYLAFAA-D---------GNGMRHLRELAGAGLNTVHLLP

TFDIAS--IPEDPA---------------DQRTPDC------------------------

---DLAALP---PDSPEQQACIAEVAAADAFNWGYDPWHFMAPEGSYASTAETAHGGQRV

AEFRAMVGGLHDAGLRVVLDQVYNHTAASGQ-AERSVLDKVVPGYYHRLD-AVGEVETST

C--CQNVATEHAMAQKLMVDSTVLWARDYKVDGFRFDLMGHHSRENMLAVRAALDELTVP

RDGVDGSAVTLYGEGWNFG-EVADNARFT----QASQGQLD-GTHIATFNDRLRDGVRGG

GPFDEDP----RTPGFGSG-GTDPN--------------------------DTDLVQIGL

TGNLRDFELRSAE-TGE-VVSGEEI-DY-NG-APAGYATDPDEVVTYVDAHDNETLFDAL

TFKL--------P---------------PDLPMEDRVRMNTVSLATATLGQGVTFWHAGT

DLLRSKSLDRNSYDSGDWFNSLDFTR---------T-DNGFG-RGLPPAPDN----EDKW

DYMRPLLA-D-PD-LE--PAPADIDAAAEAAQDLLRLRYSSPLFRLGSAEAVEQKVSFPV

S--GTAD--GRADVIVMRIDDTVG------A-DADPERDGLLVVFNASGEPLTQTVPGLE

--------GAELELSAVQAD--G-A--DEV-VKGATWDAEA--GAASVPARTVAVF----

---E---

>UniRef90_A0A0C5G0R0_951_1798 | Alpha-dextrin endo-1,6-alpha-glucosidase n=4 Tax=Streptomyces TaxID=1883 RepID=A0A0C5G0R0_9ACTN | E_val=4.7e-199

--------------LTTSEAVWIDRDTVVWN-G-P----------EGA-ASTQLRYSRTG

S-IKAENS---VLT---ST-D----ERWLRL--D-KTS--LTDAQKAKFPHLK--EYAAF

SVDPRD------RDRVR-----EALRGQLVASRTA-----A----------DGA-VLAAT

GVQIAGVLDDLY--D---AT---EAELGP-------TF----R-H----------G----

-----------RPTLAVWAPTA-RSVSLEL-------------------DGSL-EPMRRD

DT---TGVWSV------TG-PK-S-----WK--GKPYRYVVEVWAP-----STRRIVTNK

VTDPYAVALTAD-----SRQSLVVDL---ADKSLAPRGWTTY--PKP-KA-VPLRDAQIQ

ELHIRDFSVEDRT--AKHRGTYLAFTDKE---------SDGSKHLRKLAEAGTSYVHLLP

AFDIAT--IPEKKS---------------DQATVDC------------------------

---DLAALP---ADSDQQQECVAGAAGKDAYNWGYDPYHYTVPEGSYATDP---DGTART

VEFRKMVKALNEDGLRVVMDVVYNHTAAGGQ-ADTSVLDRIVPGYYQRLL-ADGSVANST

C--CANTATENAMMGKLVVDSVVTWAKQYKVDGFRFDLMGHHPKANILAVREALDALTVE

KDGVDGKKIILYGEGWNFG-EVADDARFV----QATQKNMA-GTGIATFSDRARDAVRGG

GPFDADP----GVQGFASGLYTDPNPAP--------ANGTPA-EQKARLLHYQDLIKVGL

SGNLAGYRFTD-T-GGR-EVTGSEV-DY-NG-APAGYAEAPGDALAYADAHDNETLFDAL

AFKL--------P---------------KDTPAQDRARMQVLAMATAALSQGPALSQAGT

DLLRSKSLDRNSYDSGDWFNAIHWDC--------RD-GNGFG-RGLPMAADN----ADKW

PYAKPLLT----S-VR--VGCPQIEGASAAYRDLLRIRTGEKDFSLATAAQVQSRLSFPL

S--G-PD--ETPGVITMRL-------------------GDLVVVFNATPQRQEQRVGELA

--------GTGYRLHPVQAA--G-A--DPV-VKESAYAART--GTFTVPARTVAVF----

-------

>UniRef90_A0A2S1YVT8_935_1783 | Alpha-dextrin endo-1,6-alpha-glucosidase n=3 Tax=Streptomyces TaxID=1883 RepID=A0A2S1YVT8_9ACTN | E_val=9.7e-199

------------------EAQFIDRDTVVWK-AAH----------SDA-ASRQLVYAPGG

G-IEVVDG---ALS----D-E----GHWLRL--D-PSE--LTADQKARFPHLK--DLPAF

TVDPRD------RDRVE-----KALRGQLIATQRA-----A----------NGA-LLAAT

GVQIPGVLDDLYAGR---AE---KAALGP-------VF----R-K----------G----

-----------RPTLSVWAPTA-RRVALEI-------------------GGRT-VPMRRH

DA---SGVWSV------TG-PA-S-----WR--GKPYRYAVTVWAP-----SVQKLVTNK

VTDPYSTALTTD-----SARSLVVDL---ADPKLAPEGWSTL--RKP-AA-VPLKDAQIQ

ELHVRDFSAADRT--AKHPGGYLAFTDRD---------SAGMKHLTSLAKSGTSHVHLLP

AFDIGT--IPERKS---------------GQSVPDC------------------------

---DLKAYP---PDSEEQQACVARTAAKDAYNWGYDPLHYTVPEGSYASDP---EGTRRT

VEFRRMVQGLNGAGLRTVMDVVYNHTVASGQ-SEKSVLDRIVPGYYQRLL-DDGSVATST

C--CANTAPENAMMGKLVVDSVVTWAKQYKVDGFRFDLMGHHPKANILAVREALDALTPR

KDGVDGKRIVLYGEGWNFG-EIADDARFV----QATQENMA-GTGIATFSDRARDAVRGG

GPFDEDP----GVQGFASGLFTEPNASD--------ANGTPA-EQRARLLHYQDLIKVGL

SGNLASYTFTD-T-RGR-TVKGSEV-DY-NG-SPAGYADEPGDALAYADAHDNETLYDAL

AFKL--------P---------------PSVSAADRARMQVLAMATAALSQGPALSQAGT

DLLRSKSLDRNSYDSGDWFNPVHWDC--------RD-GNGFG-RGLPPAADN----KAKW

PFARPLLA-E-PA-LK--PGCAEITGTSAAYRDLLKIRTTEKAFSLGTAGKVRSALSFPL

S--G-TE--ETPGVITMRL-------------------GDLVVVFNATPDRQTQRIGALA

--------GTPYGLHPVQAG--G-A--DAV-AKSSSYDGAT--GTFAVPGRTAAVFV---

-------

>UniRef90_UPI001670E6EE_954_1805 | pullulanase-type alpha-1,6-glucosidase n=2 Tax=Streptomyces TaxID=1883 RepID=UPI001670E6EE | E_val=1.7e-198

--------------LTTAKAVWIDRNTLAWN-G-S----------EGA-ASTQLLASRSG

S-VKAANG---TLT---AD-D----AHWLRL--S-RTT--LTEAQKAKFPHLK--AYTAW

SVDPRD------RDRVR-----EALRGQVVASQRA-----A----------GGA-VLAAT

GVQIAGALDDLYA-G---AT---RVGLGP-------VF----R-H----------G----

-----------RPTLSVWAPTA-QSVKLEI-------------------GDRT-VGMRRD

DA---TGVWSV------TG-PA-S-----WK--GKPYRYVVQVWAP-----TAGKVVTNK

VTDPYSVALTTD-----SERSLVVDL---DDRALAPRGWSHY--TKP-KA-VPLRDAQIQ

ELHIRDFSVADRTADPDHRGTYLAFTDKD---------SDGSKHLRELARAGTSYVHLLP

AFDIAT--IPEKES---------------DRTSVDC------------------------

---DLASYP---ADSDKQQECVARAAAKDAYNWGYDPYHYTVPEGSYATDP---DGTART

VEFRRMVKALNDDGLRVVMDVVYNHTAASGQ-ADTSVLDRIVPGYYQRLL-ADGSVANST

C--CANTATENAMMGKLVVDSIVTWAKEYKVDGFRFDLMGHHPKANILAVRKALDALTVE

KDGVDGKKIILYGEGWNFG-EVADDARFV----QATQKNMA-GTGIATFSDRARDAVRGG

GPFDADP----GVQGFASGLYTDPNSSK--------ENGTEA-EQKARLLHYQDLIKVGL

SGNLAGYRFTD-T-DGK-EVKGSEV-DY-NG-APAGYAEAPGDALAYADAHDNESLFDAL

AFKL--------P---------------KGTSADDRARMQVLAMATAGLSQGPALSQAGT

DLLRSKSLDRNSYDSGDWFNAIHWNC--------AD-GNGFG-RGLPPAADN----KDKW

PYAAPLLT----S-VK--VGCPQIRGASAAYRDLLKIRTTEKAFSLATAAQVQSALAFPL

S--G-KE--ETPGVITMTL-------------------GDLVVVFNATPQEQRQRVGEAA

--------GTGYRLHPVQAS--G-A--DPV-VKKAAYARDS--GTFTVPARTVAVFT---

-------

>UniRef90_A0A1F3XYF8_34_913 | Uncharacterized protein n=1 Tax=Bdellovibrionales bacterium RIFOXYD1_FULL_55_31 TaxID=1797404 Re | E_val=3.3e-198

---------------HHARAYWLDSSTLAWN-PPAD------APPSAE-CEFQLFV----

-----------------GT-E------PYPLVPSGNVG--VKRTLAKRFPYLL--NYQTL

QGTIT-------PSAAR-----AMIKQPLSVVVRH-----P----------SRG-TVFHS

GVQTFGVIDAIATYK--------GDDLGV-------RF----S-EA---------D----

-----------IPTVKLWAPTA-QSVALWIFADPLD------------AAPAEVVDMT-F

SD---SGVWSA------TG-RP-E-----WK--GAYYLYEVEVYVP-----YTGKVEKNL

VTDPYSTSLSTN-----AKRSFMIDL---RDPLTFPTGWKSV--RVQ-GP-PNAEDIAIY

ELHIRDFSANDTTVDPELRGKFLGFSDLK---------SYGMKHLRQLAAAGLTHVHLLP

AFDFGS--VNEDVS---------------QQITPQ-------------------------

---IPNGLA---PDSPEAARIIDSIKDKDAYNWGYDPVHYFSPEGSYATDP---DGLARI

VEFREMIIGLHKTGLNAVLDTVLNHTYRGGQ-DEKSILDRVVPGYYHRLD-ANGKIQGTA

CGDCADTATERFMMEKLMIDSVVSWAKNYKVAGFRFDLMGFHTLSNLKHLRAALNQLTLE

KDGIDGSRIYLYGEGWKFG-SLEAILPNE----TANQMNLY-GAGVGSFNDRFRDRARGG

GFMTETK----ADQGFITGLYYDNNGIGSK-----ALPQKPR-DLRGRLLQYTDAIRVGM

AGNLRNFRFRN-S-EGK-SVTGAQI-DY-HG-SPVGYAARPGETINYVSAHDNYDLWDQI

AAKAAFQKPDRYP---------------ETASIEDRVRMQKLGLALVALGQGVPFFHAGS

EFLRSKSGDNNSYNSGDWFNRMDFSL---------R-DNNWG-VGLPPGE-N----SGDA

AFWAPRLR-D-PE-LR--ASAKDMESTTRYFQALLEIRRDSALLRLTTTEDVQARLRFLE

TDFG-KE--QPPGLIVMALQDSIR----NRP-KLDSSRKLLLVAINVTRHPISFRHSFLK

--------DRKLRLHPNWTE--E-I--EPR-VLDSSFTPER--GTLAIPAQTVLVYEEPR

-------

>UniRef90_UPI001009E3C7_907_1758 | pullulanase-type alpha-1,6-glucosidase n=1 Tax=Streptomyces roseicoloratus TaxID=2508722 RepI | E_val=6.4e-198

-----------------AEAQWIDRNTVVWK-VKT----------TDA-TSQQLVYAENG

G-ITVTDG---ALS----D-E----GRWLRL--T-AAR--LTDAQKARFPHLR--DYPAF

TVDPRD------ADRVR-----GALRGQLIATQRA-----A----------NGA-LLAAT

GVQTAGVLDDLYAAK---AT---GANLGP-------VF----R-G----------G----

-----------RPTLSVWAPTA-QSVALEL-------------------DGRT-VPMRRD

EI---SGVWSV------SG-PK-S-----WQ--GKPYRYVVKVWAP-----SVRKLVENR

VTDPYSTALTTD-----SARSLVVDL---ADPALAPAGWKTL--RKP-AA-VPLRDAQIQ

ELHIRDFSVADPTAKAAHRGTYLAFTDKD---------SKGSRHLKALAASGTGYVHLLP

AFDIGT--IPEKKS---------------AQTTPSC------------------------

---DLEVHA---PDSEEQQACVAAAAARDAFNWGYDPLHYTVPEGSYATDP---EGTRRT

VEFRRMVQALNGDGLRTVMDVVYNHTVAAGQ-SDKSVLDRIVPGYYQRLQ-ADGSVATST

C--CANTAPENAMMGKLVVDSVVTWAKQYKVDGFRFDLMGHHPKENILAVREALDALTVE

KDGVDGKAIVLYGEGWNFG-EVADDARFV----QATQKNMA-GTGIATFSDRARDAVRGG

GPFDEDP----GVQGFASGLFTDPNTSK--------ANGTPE-QQKARLLHYQDLIKVGL

TGNLASYTFTD-T-AGR-TVKGSEV-DY-NG-SPAGYAAAPGDALAYADAHDNESLYDAL

AFKL--------P---------------AGTPAADRARMQVLAMATATLSQGPALSQAGS

DLLRSKSLDRNSYDSGDWFNAIHWDC--------RD-GNGFG-RGLPPAADN----QAKW

GYARPLLS-N-PA-LT--PGCAEIDGASAAYRDLQKIRTTEPVFSLGTTARVQDALSFPL

S--G-RD--ETPGVITMRL-------------------GDLVVVFNATPQQQKQTVGTLA

--------GKGYRLHPVQAH--G-S--DAT-VRTAAYEPGT--GAFTVPARTVAVFT---

-------

>UniRef90_UPI0009E94994_1277_2119 | pullulanase-type alpha-1,6-glucosidase n=2 Tax=Sanguibacter TaxID=60919 RepID=UPI0009E94994 | E_val=1.5e-197

------------------------------------------------------------

-----------------DG-T------DLPLTLL-PDG--VPEDLRTRHPLAS--GYLAL

ALDAGV-----TDEQVA-----GLLTGELRLTQAG-----P----------AG--LEYST

GVQVSGVVDDLFTV----AA---DTPLGA-------TW----D-G----------D----

-----------VPTLTLWAPTA-KSVTTRVWLDGDGLAA---------GTPTD-VPAV-R

QA---DGTWVT------QG-AA-S-----WK--DAAYVYDVEVYVH-----STGAVEHNV

VTDPSSVALTLN-----SAQSVLVDL---ADPTYQPALWADT--PQP-VV-ERDVDRSIY

ELHVRDFSISDESVPEAERGTYKAFTR-D---------SDGMKHLRSLAAAGLNTVHLLP

TFDIAT--IEEDRA---------------LQETTG-------------------------

---DLSGFA---PDSTEQQAAVGAVASTDAFNWGYDPLHFTAPEGSYATEG-NQDGGARV

AEFRSMVGGLHASDLQVVLDQVFNHTAASGQ-SEKSVLDRVVPGYYQRLS-ATGAVETST

C--CENVATENAMAERLMVDSVVTWARDYKVDGFRFDLMGHHSRDTMLAVRAGLDALTLE

RDGVDGSSVYLYGEGWNFG-EVADNALFE----QATQGQLG-GTGIGTFSDRLRDAVHGG

SPVSGDSI---FQQGFGTGLGTDPNGRPTSTSDPATLNDGSP-EELAALAHSTDLLRLGL

AGNLRAFSFET-S-AGT-VQRGDEI-DY-NG-QPAGYADSPEEVVTYVDAHDNETLFDIL

TLKL--------P---------------VETSMADRVRMNTLSLATTTLSQTPSFWHAGT

DLLRSKSLDRDSYNSGDHFNEIDWTG---------Q-ENTFG-SGLPPEEKN----GDKW

DYMRPLLA-D-PA-LK--PAPADMATAQAAASDLMRLRFSTPLFRLGEAGLITEKVTFPG

S--G-AD--AAPGVIVMHVDDTVG------A-DVDPALDGLLVVFNASPESVTQTVDGLV

--------GRELALSSVQAD--G-S--DAV-VRETTWDAAT--GQVTVPARTVAVLTETQ

-------

>UniRef90_A0A7W7HWN5_938_1805 | Pullulanase-type alpha-1,6-glucosidase n=2 Tax=Actinoplanes digitatis TaxID=1868 RepID=A0A7W7HWN | E_val=3.5e-197

----------------KSNAVWIDRETIAWK-TGTGTTLEPVGAGTDG-RVYDLVWAPEG

G-LSILDG---ELA---GP------HESVRLTAR-RNG--LTEAQRAKNPHLW--QYPAF

RTG--D------LSGLK-----DVLRGQVAVTERD-----A----------EGK-LISAT

GVQTAGVLDDIYAD----AA---DEDLGP-------TF----D-G----------K----

-----------RPTLAVWAPTA-RTVALELFDDPA-------------ATAKQ-VPMTRD

AD---TGVWSA------TG-TK-D-----WK--GKYYRYRVTAWQP-----AAGKVVTAS

VTDPYSVDLSAD-----STHSRITDL---TDPALAPAGWSGL--RKP-EY-TP-GRIQIS

ELSVRDFSIGDETVPPAHRGTYGAFTDPR---------TAGMKQLKSLAAAGTTHVHLLP

VFDFAT--IPEKRA---------------DQKQPAC------------------------

---DLAKLP---PDSDEQQKCVTAVAAQDGFNWGYDPLHYTTPEGGYAVDP-----AART

KEFRQMVAGLNGTGLRAVMDVVYNHTSAAGT-DPHSVLDQIVPGYYQRLL-EDGTVANST

C--CANTAPENTMMGKLVVDSIVTWAKAYKVDGFRFDLMGHHPKANILAVRAALDKLTPA

RDGVDGKKIYLYGEGWNFG-EIANDARFE----QATQANMA-GTGIGTFNDRLRDAVRGG

GPFDGNP----RIQGFASGLFTAPNGDP--------INGTPE-EQKARLLRYQDQIKVGL

SGNLASYRFVN-S-AGT-PVTGAQV-DY-NG-SPAGYNAAPGEAITYVDAHDNEILYDAF

AYKL--------P---------------QATPALDRAKLQSVALATTALGQGVGFVTAGS

ERLRSKSLDRNSFNSGDWFNQIAWDC--------TK-GNGFG-RGLPPAADN----EPKW

QYAKPLLA-D-KA-LV--PGCDAVNLADARYRELLEIRKSSPVFGLPTAAEVQKRLSFPL

S--G-TA--ETPGVITMHLD---G------R-GLDRRWKSVTVVFNATPAAVTQTVPALS

--------GASIGLHPAQHE--P-----------ATFDRAT--GAFRVPARTTAVFVQ--

-------

>UniRef90_A0A7T8DZR6_263_1009 | DUF3372 domain-containing protein n=1 Tax=Actinomyces sp. HMT 897 TaxID=2789424 RepID=A0A7T8DZR6 | E_val=8.1e-197

------------------------------------------------------------

------------------------------------------------------------

------------------------------------------------------------

------------------------------------------------------------

-------------SFALWAPTA-RAVTLLTWETGDPLGSV----PEVPGPPVR-TPAV-R

GE---DGRWCV------PN-AE-GAI---GV--GCQYLWEVEVYVP-----STRTVETNL

VTDPYSVALTLD-----STRSVAVDL---ADGRLAPPQWATT--PAP-RL-RNDSARSIY

ELHVRDFSAADPTVPPDLRGTYLAFTVED---------SAGVRHLADLAEAGMNTVHLLP

TFDIAT--IPEDRS---------------AQRAPSI------------------------

---P-AGAG---PASTEQQAAIAAVADNDAYNWGYDPYHWGVPEGSYATEG-HQDGGARV

VEFRQMVGALHALGYQVVLDQVYNHMAACGQ-DPFSVLDKVVPGYYHRLD-AVGTVTASA

C--CANTATENAMCERLMIDSVVRWARWYRVDGFRFDLMGHHPRATMERLRAALDRLTPQ

ADGVDGAGLYLYGEGWNFG-EVANNALFT----QATQGQLD-GTGIGTFNDRLRDAVHGG

SPFDPDHR---TYQGFGTGLLTQSNGLD--------PRGWN--DQAADLGHRTDLVRLGL

AGNLRDYVMTL-S-DGS-VRRGADL-LH-NG-SPAAYASAPQENVNYVDAHDNETLYDLL

AYKL--------P---------------RGMTPAERVRMNTVCLATVALGQSPAFWCAGT

ELLRSKSLDRDSFNSGDWFNAIDFSG---------Q-SNGFG-RGLPPASRN----EGAW

PIQGPLLA-D-AW-LR--PTSEQIAAARAQALDLLRLRSSTPLFALGDAALIRDKLRFPG

A--G-FG--APPGAIAMLIDDTVG------R-DVDPGLDAVLVVFNASGHTLTWPLGELV

--------GRGLHLSPVQAE--G-A--DDV-VRRTGFDRST--GTVSVPARTVSVLVQ--

-------

>UniRef90_A0A562Q1D6_169_1039 | Alpha-dextrin endo-1,6-alpha-glucosidase n=1 Tax=Massilia flava TaxID=871742 RepID=A0A562Q1D6_9B | E_val=1.4e-196

-----------------ANAHWLSATTLAWP-GA-----------PAT-GTYKLYYAANG

G-MTASPDATTGLA---GA-D-----GSLNLQ---VAA--LPDAMRQKYPHLA--GATGL

RLAETE------LANVGRFNTLRVASAQFAIAQYG-----A----------DGK-LVQVT

SLQQAGMLDDVFATH---AA---NTPLGL-------TF----D-GR---------G----

-----------VPTFRVWAPTA-KSVRLDVYPNATA------------AATAT-VPMTRD

AA---SGVWSY------TAPDA-S-----WTN-SAYYTYTVQVLSR----WANNTVVTNT

VTDPYSVSVSAN-----STRSLVANL---DSPRLKPAGWDEQ--RIP-RL-DAPTDIVLY

ELHVRDFSALDTTVPAAHRGKYLAFTDGD---------AAPMRHLKSLQKAGLTHVHLLP

SFDFAS--VNEAGC-----------------VTP--------------------------

---AIPNGA---ANATTQQAAVAATADTDCFNWGYDPVHYNTPEGSYATDT--NDGAARV

REFRAMVQALHEAGLRVTMDVVYNHTSQSQQ-GPLSVLDRIVPTYYYRLG-AGGNILNDS

C--CADTAQENAMMAKLMTDSVALWAKQYKIDSFRFDIMGFTPLDLMKRLQAAADQAA--

-----GRPVYLYGEAWNFG-AVGNDARFV----QARQANMF-GTGIGSFNDRIRDTVRGG

GCCDTGTA-LVTQQGFANGVWFDPNGQS--------------TQTRDDALRLADLVRVAL

SGTLRDYRFTD-R-SGA-VRSNADI-DY-FG-QRAGFAGSPAETINYIEAHDNQTLFDVN

ALKL--------P---------------QATPLAERVRVQTLGAAINMLAQGVPFFHAGQ

EILRSKSLDRDSYNAGDWFNRLDYGY---------T-SNNFG-VGLPMAGVN----EGSW

SVMSPVLA-N-PL-IA--PDTRAILAAKAGFEELLAIRKDSTLFRLRTAQDVIDRLKFHN

T--G-PD--QVPGIVAMSISGSAP-----SP-YPGAQYKQVVVVFNVDKVAKSVALPALK

--------GSKLQLHRIQRN--G-S--DSV-VKASTYDAAT--GSFTIPGRTTAVFVQ--

-------

>UniRef90_UPI000567CB76_1013_1857 | pullulanase-type alpha-1,6-glucosidase n=1 Tax=Kitasatospora azatica TaxID=58347 RepID=UPI000 | E_val=2.1e-196

------------------------------------------------------------

--------------------------YWLRLNPV-AGG--LTDAQKKKYPNLA--GYRAF

TVDSRD------TARVP-----QALRAQLVFTEHL-----P----------SGA-ALAAT

GVQIPGVLDARYAGQ---AA---QAQLGP-------VY----R-Q----------SGP--

----WWWPFNREVTLSLWAPTA-QQVTVQLYDSATG------------GTPRA-VPLKRD

EA---SGVWSL------TD-SRAA-----LA--GKYYVYQVKVWAP-----SVQQLVTNT

VTDPYSVALSPD-----SKRSLVADL---TDRTTKPAGWDDS--RSP-QA-VAASRQQIQ

ELQVRDFSAADSTVPAAERGSYLAFTESQ---------SAGMQHLRELAKAGITAIHLLP

TFDIAS--IPPT-A---------------DQKLPAC------------------------

---DLAALP---KDSDKQQECVAAVAAQDAYNWGYDPLHYTVPQGSYATDP---TGTART

VQFRQMVQAIHAAGLRVVLDVVYNHTAAAGQ-DEHSVLDQVVPGYYQRLS-DSGAVTTDS

C--CADTAPEHAMMNKLIVDSVRTWAEQYKVDGFRFDLMGLDPKSTMLDVQSELKKLTIR

QNGVDGSSIFLYGEGWNFG-VVANNARFE----QATQLNMA-GTGIATFNDRVRDAGRGG

NFMLSSA----PQQGFASGLYTDPNGSS--------ANGSSD-QQKAALLHQMDQIKVGL

TGNLADYSFTD-S-SGK-SVTGAGV-DY-NG-SPTGYAANPGEAVEYLDAHDNTDLFDAL

AYKL--------P---------------TSTSAADRARMQALGLSLTALTQGPGFAVAGS

DLLRSKSLDSNSFNSGDWFNAIHWPVPDSRTCADGN-GNGWG-HGLPPAADN----QSMW

PVAKALLA-D-PR-LT--VGCDRISATSQQYQEFLRIKQGSPLFGLSSAAEVQRRLSFPL

S--GTAG--ETPGVITMHLD---G------T-GLPGAERGITVVFNATPSPQSQALPALA

--------GTSQQLHPVQAN--G-A--DPV-VKQATFQSDS--GRFTVPARTVAVFVQ--

-------

>UniRef90_A0A7X6KMF7_941_1791 | Pullulanase-type alpha-1,6-glucosidase n=1 Tax=Streptomyces somaliensis DSM 40738 TaxID=1134445 | E_val=4.2e-196

-----------------STAVWIDGTTVAWN-G-A----------PTA-ASSQLVYSPTG

S-LAVKDG---VLT---GE-D---QAHWLRL--R-PTA--LTDAQKAAFPHLA--KYTAY

SVDPRD------RDRIR-----TALRGQLVATQRA-----A----------DGA-LLTAT

GVQTAGALDALYGEE---AR---TRTLGP-------VF----R-G----------G----

-----------TPTLSVWAPTA-RSVALEL-------------------DGRT-VPMRRD

DA---TGVWSV------RG-TR-S-----WK--GKPYRYVVTVWAP-----SVRRIVTNR

VTDPYSTALTTD-----SARSLVVDL---SAPELAPEGWATL--RKP-PA-VPLRDARIQ

ELHVRDFSASDPT--SEHPGGYLAFTDRD---------SAGMRHLAELARSGTSHVHLLP

VFDIGT--VPERRS---------------DQTAPDC------------------------

---DLPAHP---ADSERQQECVAGAAAKDAFNWGYDPLHHTVPEGSYASDP---EGPRRT

VEFRRMVRSLGDAGLRTVMDVVYNHTVAAGQ-DDGSVLDRIVPGYYQRLL-ADGSVATST

C--CANTAPENTMMGKLVVDSVVTWAREYKVDGFRFDLMGHHPKENVLAVRRALDALTVG

KDGVDGKEIILYGEGWDFG-EVAEGARFV----QATQENMA-GTGVATFSDRARDAVRGG

GPFDADP----GVQGFASGLYTEPNGSA--------ANGTRA-EQRARLLHYQDLIKVGL

SGNLADYAFTD-S-SGR-RVKGSEV-DY-GG-APAGYAAAPGDALAYADAHDNETLYDAL

AFKL--------P---------------AGTSPGDRARMQVLAMATAALSQGPALSQAGT

DLLRSKSLDRNSYDSGDWFNALHWDC--------RL-GNGFG-RGLPPAADN----RDKW

PYARPLLS-S-PS-LK--AGCAQIDGASAAYRDLLRIRTTERVFGLATTEQVRSALTFPL

S--G-PD--ETPGVITMRL-------------------GDLVVVFNATPERREQTVPALA

--------GGGYALHPVQAG--G-S--DAT-VRTAAYDGTS--GTFTVPARTVAVFT---

-------

>UniRef90_A0A1Q5KFV1_951_1796 | Alpha-dextrin endo-1,6-alpha-glucosidase n=1 Tax=Streptomyces sp. TSRI0107 TaxID=1703942 RepID=A | E_val=5.6e-196

-----------------AKAVWIDRDTLVWN-G-S----------DAA-ASTQLLYSRTG

S-IKAEKG---VLT---ST-D----ERRLRL--N-KTS--LTDAQKAKFPHLK--SHTAW

SVDPRD------RDRVR-----EALRGQLVAAQYA-----A----------NGA-VLSAT

GVQIAGVLDDLY--D---AT---KADLGP-------TF----R-D----------G----

-----------RPTLAVWAPTA-QSVSLEL-------------------DGSL-KPMRRD

AA---TGVWSV------TG-SK-S-----WK--GKPYRYVVKVWAP-----SVREVVTNK

VTDPYSVALTTD-----SKRSLVVDL---ADRSLAPSGWSGL--RKP-KA-VPLKDAQIQ

ELHIRDFSVADRT--AKDRGTYLAFTDKD---------SDGSRHLRELAEAGTSYVHLLP

AFDIAT--IPEKKS---------------EQATVDC------------------------

---DLASYP---ADSDKQQECVAKAAAKDAYNWGYDPYHYTVPEGSYATDP---EGTART

VEFRRMVKALNEDGLRVVMDVVYNHTAASGQ-ADTSVLDRIVPGYYQRLL-ADGSVANST

C--CANTATENAMMGKLVVDSVVTWAREYKVDGFRFDLMGHHPKENILAVRKALDALTVA

KDGVDGKKIILYGEGWNFG-EIADDARFV----QATQKNMA-GTGIATFSDRARDAVRGG

GPFDADP----GVQGFASGLYTDPNPNA--------ENGTEA-EQKARLLHYQDLIKVGL

SGNLAGYAFTD-T-RGE-RVKGSEV-DY-NG-APAGYAAAPGDALAYADAHDNETLFDAL

AFKL--------P---------------SSVSAADRARMQVLAMATATLSQGPALSQAGT

DLLRSKSLDRNSYDSGDWFNAIHWNC--------AD-GNGFG-RGLPPAADN----QAKW

PYAKALLT----K-IQ--VGCPQIEGASAAYRDLLRIRTTEKAFSLDTAAQVQSALSFPL

S--G-TE--ETPGVITMKL-------------------GDLVVVFNATPERQEQRIAELA

--------GDGYRLHPVQAS--G-S--DAV-VKKSAYTEES--GTFTVPGRTVAVFT---

-------

>UniRef90_A0A6I3SVI6_168_1035 | Alpha-dextrin endo-1,6-alpha-glucosidase n=1 Tax=Massilia buxea TaxID=1949069 RepID=A0A6I3SVI6_9 | E_val=1.1e-195

--------------LTSAKAHWLSGTTLAWP-GA-----------PAG-ATYKLYYAANG

G-MTAGPDANTGLA---GA-E-----GSFALQ---AAS--LPDALRQKFPHLA--GATAL

RLSDAD------AARAA-----RLASGQFAIAQFA-----A----------NGS-LVQVT

SLQQAGMLDDVFATR---AA---SAQLGL-------SF----D-R----------G----

-----------VPTFRVWAPTA-KSVRLTVYSAPTG------------GKSDT-LPMTLD

AA---SGVWRY------TAPNA-S-----WTN-RAYYTYTVQVLSR----WANNTVVTNT

VTDPYSVSVSAN-----SARSFVADL---DSAQLKPAGWDEH--RIP-KL-DSPLDIALY

ELHIRDFSASDSTVPAAHRGKYLAFTDTE---------SAPMRHLKSLQKAGMTHVHLLP

SYDFSS--VNEAGC-----------------ATP--------------------------

---AIPGGA---ADATIQQAAVAATRDSDCFNWGYDPVHYNTPEGSYATDT--SNGAVRV

REFRAMVQALHEAGLRVTLDVVYNHTSSAKQ-DPLSVLDRIVPTYYYRQG-AEGNLLNDS

C--CADTAQENVMMAKLMLDSASLWARQYKIDSFRFDIMGFTPLALMQRMQGAVDAAA--

-----GRPIYLYGEAWNFG-AVANDARFV----QARQANMF-GTGIGSFNDRIRDTVRGG

GCCDTGAS-LVNQQGFVNGAWFDPNGQS--------------TQTRDDALRLADLARVAL

SGTLRDYRFTD-R-FGT-VRSNAEI-DY-FG-QQAGFAGSPAETINYVEAHDNQTLFDVN

ALKL--------P---------------QNTPLADRVRVQTLGAAVNILSQGVPFFHAGQ

EILRSKSLDRDSYNAGDWFNRLDYGY---------T-SNNFG-VGLPMAGSN----ESSW

SIMAPVLA-N-PL-IS--PDTRAILAARAGFEELLAIRKDSTLFRLRTAKDVSERLAFHN

T--G-PN--QVPGIVAMSISGDQP-----SH-YPGAQYKKVVVVFNVDKTAKSVAIPELK

--------GLKLQLHRIQKS--G-S--DAV-VKTSAYDTAI--GSFTIPARTAAVFVQ--

-------

>UniRef90_A0A6G3BWK8_946_1794 | Alpha-dextrin endo-1,6-alpha-glucosidase n=10 Tax=Streptomyces TaxID=1883 RepID=A0A6G3BWK8_9ACTN | E_val=1.6e-195

--------------LTTSKAVWIDRNTLAWN-G-S----------DAA-ASTQLLYARDG

S-IAVKDG---ALT---G--D----ATWLRL--S-RSE--LTDAQQAKFPHLK--EYDAW

TVDPRD------RDRVR-----EALRGQVVATQHA-----V----------NGA-VLAAT

GVQIAGVLDDLYS-G---AT---GADLGP-------TF----D-K----------G----

-----------RPTLSVWAPTA-QQVSLEI-------------------GDST-TAMRRD

AG---TGVWSV------TG-PK-S-----WK--GKPYRYVVKVWAP-----SVRKVVTNK

VTDPYAVALTAD-----SKRSLVVDL---DDKALKPTGWSAY--TKP-KA-VPLRDAQIQ

ELHIRDFSVEDRT--AKHPGTYLAFTDKA---------SDGSKHLRELAKAGTSYVHLLP

AFDIAT--IPERKT---------------DQAKTDC------------------------

---DLASFP---ADSDKQQECVGKIAAKDAYNWGYDPFHYTVPEGSYATDP---DGTART

VEFRKMVRALNDDGLRVVMDVVYNHTAASGQ-ADTSVLDRIVPGYYQRLL-ADGSVANST

C--CANTATENAMMGKLVVDSVVTWAKEYKVDGFRFDLMGHHPKANILAVREALDALTPA

KDGVDGKKIILYGEGWNFG-EIADDARFP----QATQKNMA-GTGIATFSDRARDAVRGG

GPFDEDP----GVQGFASGLYTDPNSSK--------NNGTEA-EQKARLLHYQDLIKVGL

SGNLAGYRFTD-T-AGK-EVKGSEV-DY-NG-APAGYADAPGDALAYADAHDNETLFDAL

AFKL--------S---------------ASTSAADRARMQVLAMATATLSQGPALSQAGT

DLLRSKSLDRNSYDSGDWFNAVHWRC--------ED-GNGFG-RGLPMAADN----TDKW

PYAKPLLT----K-VT--VGCEQIEGTSAAYRDLLRIRSTERDFSLGTAAQVQSRLSFPL

S--G-TD--ETPGVITMRL-------------------GDLVVVFNATPEKQEQRVPALA

--------GAGYRLHPVQAA--G-A--DST-VKSASYAAES--GTFAVPGRTVAVFT---

-------

>UniRef90_A0A0J1GDZ5_167_1036 | Alpha-dextrin endo-1,6-alpha-glucosidase n=1 Tax=Massilia sp. WF1 TaxID=1406431 RepID=A0A0J1GDZ5 | E_val=2.1e-195

-------------SLAFASAHWLSATTLAWP-GA-----------PAG-GSYKLFYAANG

G-LAATPDAATGLA---GA-D-----GSFTLS---AAP--LSDAIRQKYPHLA--GATGL

QLAAAD------AAKVA-----QLASGQFAIAQYD-----G----------AGN-LVQVT

SLQMAGMLDDVFARA---AS---NAQLGV-------SF----D-RA---------G----

-----------VPTFRVWAPTA-KSVSLNVYPSASA------------PATAS-VAMSAD

AA---SGVWRY------TAPDA-S-----WTN-RAYYTYTVNVLSR----WANNALVTNT

VTDPYSLSLNAN-----STRSFVANL---DSATLKPAGWDDQ--RIP-KL-DAPTDIALY

ELHVRDFSALDTTVPAAHRGKYLAFTDLD---------ANPMRHLRELQKSGMTHIHLLP

SFDFAS--VNEAGC-----------------VLP--------------------------

---SVPNAA---ADSTAQQAAVAASQDSDCFNWGYDPVHYNAPEGSYATDA--NDGAARV

REFRAMVQSLHETGLRVTMDVVYNHTSQSQQ-GPLSVLDRIVPTYYYRLG-ANGSILNDS

C--CADTAQENTMMGKLMIDSVSLWARQYKVDSFRFDIMGFTPLELLKRLQAGVNQAA--

-----GRDIYLYGEAWNFG-SVANDARFV----QARQANMA-GTGIGSFNDRMRDAVRGG

GCCDGGSA-LVTQQGFINGAWLDPNAQA--------------NQSRDDALRLADMVRVSL

SGTLRDYRFTD-R-FGS-LRTNAQI-DY-FG-QQAGFAANPSEVINYIEAHDNQTLFDIN

AFRL--------P---------------QSTLLSDRVRVQTLGASIVLLSQGVPFIHAGQ

EILRSKSLDRDSYNAGDWFNRLDYSY---------G-ANNFG-VGLPMAGPN----QDNW

GIMAPILT-N-PL-IR--PDTRAILSAKGAFEDLLAIRKDTSLFRLRTAQDVIDRLKFHN

V--G-PS--QVPGAIVMEIDGNDP-----GK-YAGAQYKGVVVVFNVDKVAKTFAVPALK

--------GRKLQLHRIQRN--G-S--DEV-VKASAFDSAS--GGFSIPARTTAVFVQ--

-------

>UniRef90_A0A7Y0DE16_1051_1945 | Pullulanase-type alpha-1,6-glucosidase n=2 Tax=Phycicoccus sp. TaxID=1902410 RepID=A0A7Y0DE16_9M | E_val=5.4e-195

--------------LAKSKAYWVSNDLVAWP-ATAVP----SGANLAL-LNWRLHWSPTG

G-LAVDAE---SVT---GG-A------VADLTYD-SAG--LPAAVVAAHPELK--GFLAL

RLDDKT------AKLAG-----TILQGQVAVALYD-----D----------LGR-LLDAT

GVQTPGVLDDLYGS----AA---SRTYGV-------TW----K-SESPSFMLPSFT----

-----------SPSFTLWAPTA-QKVSVLLWPASASADAPV-------TEATR-VPMI-R

RS---DGSWGA------DT-GS-Q-----WR--GARYLFEVVVYAP-----TTGKVETNL

VTDPYSVALTLN-----STRSVAADL---ADPTLKPAIWQGS--SAP-VL-KQRVDQTIY

ELHVRDFSVNDSTVPVAHRGSYLAFAD-N---------GDGTKHLKALAAAGLNTIHLLP

TFDIAS--IEEDPA---------------KQLKPAC------------------------

---NLASFA---SDSEEQQKCVMAAAGKDAFNWGYDPFHWMAPEGSYASSPTAADGGSRV

AEFRTMVGGLHADGLRVVLDQVFNHTPTSGQ-ADTSVLDKVVPGYYQRLN-ATGAVETST

C--CQNVATEHAMAQKVMVDSVVSWVRNYHVDGFRFDLMGHHSKANMLAVRAALDALTSE

HDGVDGKSITLYGEGWNFG-EVANNKLFT----QATQGNLG-GTGIASFSDRLRDGVRGG

GPFDEDP----RKQGFGSGEATDPNGAP--------INAGA----TASLAHDADLVQLGL

AGNLRSFSFKDNA-SGV-VVRGDKV-DY-NG-SPAGYADQPDEVVNYVDAHDNETLWDSL

TFKL--------P---------------VATSMADRVRMNSLSLATTALSQTPSFWHAGA

DLLRSKSLNRNSYDSGDWFNTLDWTG---------A-DNGFG-HGLPPSADN----SAKW

PFMKPLLA-N-PA-LK--PSATDVQSATAQAQALLELRFSSPLFRLGSADAINTKVTFPV

S--GTAD--AHQGVIVMRVDDTLG------A-DVDPALAGLVVVFNGSTATVNQKLPGMA

--------GANLTLSSVQST--G-S--DPV-VKTSLWDGAA--STLSVPARTVAVFVQ--

-------

>UniRef90_A0A7K2Y0A8_226_1073 | Alpha-amylase n=4 Tax=unclassified Streptomyces TaxID=2593676 RepID=A0A7K2Y0A8_9ACTN | E_val=9.4e-195

--------------LTKSQAVWIDRDTLAWN-A-P----------ATA-ASVQLLASREG

A-VKAENG---TLT---G--R----AQWLRLG-A-RTE--LTAAQKQKFPHLA--AYGAH

TVDPRD------RDRVR-----EALRGQLVASARA-----A----------NGA-VLAAT

GVQLAGVLDDLY------AT---TTSLGP-------VF----K-D----------G----

-----------RPTLSVWAPTA-QQVSLEL-------------------DGRT-VAMRRD

DA---TGVWSV------RG-ER-S-----WT--GKPYRFDVTVWAP-----STRQVVRNL

VTDPYSTALTAD-----SVHSLLVDL---ADPKLAPPGWKTL--RKP-AP-VPFTSAQIQ

ELHIRDFSVADRT--TTHPGQYLAFTDTA---------SAGMRHLRDLATAGTSYVHLLP

AFDIGT--IPEKAA---------------DRTEPAC------------------------

---DLWVYA---PDSQEQQACVAAAAAKDAYNWGYDPLHYTVPEGSYASDP---NGTART

VEFRRMVQSLNGSGLRTVMDVVYNHTVAAGQ-SDKSVLDRIVPGYYQRLL-ADGSVATST

C--CANTAPENAMMGRLVVDSIVTWAKEYKVDGFRFDLMGHHPKANILAVRSALDALTVA

KDGVDGKKIVLYGEGWNFG-EIADDSRFV----QATQKNMA-GTGIATFSDRARDAVRGG

GPFDEDP----RVQGFASGLFTAPNASP--------ANGTAE-QQKARLLHAQDLIKVGL

SGNLASYAFTD-T-TGR-RVKGAEV-DY-NG-APAGYAAAPGDALAYADAHDNESLADAL

TYKL--------P---------------TGTAPRDQARMQVLAMATAALSQGPALSQAGT

DLLRSKSLDRNSYDSGDWFNAIHWDC--------RD-GNGFG-RGLPPAADN----ASKW

LYAKPLLT-G-PA-----PSCTDIGATSAAYRDLLRIRTTEPDFALTTTEAVQSRLAFPL

S--G-KD--ETPGVITMTL-------------------GDLVVVFNAAPTGAAQRVPALA

--------GATYRLHPVQAA--G-S--DAA-VKGSTYDAKT--GEFTVPARTVAVFT---

-------

>UniRef90_A0A2G5IV34_945_1792 | Alpha-dextrin endo-1,6-alpha-glucosidase n=2 Tax=Streptomyces TaxID=1883 RepID=A0A2G5IV34_9ACTN | E_val=1.8e-194

--------------LTTSKAVWIDRNTVAWN-G-S----------EGA-ASTQLLYSRDG

S-IAVKDG---ALI---G--D----ARWLRL--S-KTS--LTGAQKAKFPHLK--DYTAW

SVDPRD------RDRVR-----AALSGQVVASQRA-----A----------NGA-ALAAT

GVQIAGVLDDLY--D---AT---KAELGP-------VF----H-R----------G----

-----------RPTLAVWAPTA-QSVSLDL-------------------DGST-VAMKRD

AT---TGVWSV------TG-KK-S-----WT--NKPYRYVVKVWAP-----SVRKVVTNK

VTDPYSVALTAD-----SERSLIVDL---DDTSLAPSGWSSL--KKP-QA-VALKDAQIQ

ELHIRDFSVEDKT--ARHPGTYLAFTDKD---------SEGSRHLRRLAESGTSYVHLLP

AFDIAT--IPEKKA---------------DQARTDC------------------------

---DLASYA---PDSEKQQECVAKTAAKDAYNWGYDPYHYTVPEGSYATDP---DGTART

VEFRKMVKSLNEEGLRVVMDVVYNHTTSSGQ-ADTSVLDKVVPGYYQRLL-ADGSVATST

C--CANTAPENAMMGKLVVDSIVTWAKEYKVDGFRFDLMGHHPKANILAVRKALDALTPA

KDGVDGKKIILYGEGWNFG-EIADDARFT----QATQKNMA-GTGIATFSDRARDAVRGG

GPFDEDP----GIQGFASGLSTDPNSSA--------ANGTPA-EQKARLLHYQDLIKVGL

SGNLAQYRFTG-T-DGK-EVKGSEV-DY-NG-QPAGYADAPGDALAYADAHDNESLFDAL

AFKL--------P---------------GSTSASDRARMQVLAMATATLSQGPALSQAGS

DLLRSKSLDRNSYDSGDWFNAIHWNC--------QD-GNGFG-RGLPMAADN----ASKW

PYATPLLT----R-VK--VGCDQIEGTSAAYRDLLRIRTAESAFGLSTADQVQSKLSFPL

S--G-KD--ETPGVITMQL-------------------GDLVVVFNATPEKQEQTIGALA

--------GKGYALHPVQAS--G-A--DPI-VKSASYEAES--GTFAVPGRTVAVFA---

-------

>UniRef90_UPI00194EDC74_345_1160 | pullulanase-type alpha-1,6-glucosidase n=1 Tax=Spirilliplanes yamanashiensis TaxID=42233 RepI | E_val=4.5e-194

------------------------------------------------------------

----------------------------LPLTAR-PGG--LFQVQRRQFPHLG--SYRAL

AVPEND------PARLG-----ELLRGQLLVVGLD-----A----------EGR-VVTVT

GVQLPGILDDLYAD----AR---DAPIGP-------GL----E-D----------G----

-----------RPTLAVWAPTA-RTVTLQLWKAPG-------------EEPRE-HPMTRD

DA---TGIWSV------TG-RH-K-----WL--GRAYRFAVEVWHP-----AAQAVVTES

VTDPYAVALTAG-----SAHAVIADL---TDPALAPPGWDR---PPP-RP-VPPARAVIA

ETSVREFSYLDASVPAEHRGTYRAFTHPD---------ATSVRHLRRLADAGLTHLHLLP

AFDFAT--VPDRRA---------------DQAEPEG------------------------

---DLASFP---PDSPEPQARVAAVADRDGWNWGYDPLHYNVPEGSFATDP---DGPART

REFREMVAALHAAGLRVVMDVVYNHTMADGL-APYSVLDRIVPGYYHRLL-ADGTVAAST

C--CSNTASEHAMTARLVVDSVVFWARHYRVDGFRFDLMGHHPRRLMLDVRAALDALP--

----GGRDIYLYGEGWEFG-EVAGDARFV----QATQRHLA-GTGIGSFNDRLRDAARGG

VAFEDNP----RAQGFATGLWTAPNGDG--------VNGDLH-RQRETLLHLQDRIKVGL

TGNLAAYMFTD-H-RGR-HVTGAQV-DY-NG-SPTGYAGAPGECVTYVDAHDNEILYDAL

AFKL--------P---------------VETAPLDRARAQLVALALVLLGQGVGFFALGS

ERLRSKSLDRNSYNSGDWFNAIRWDP--------AQ-GNGFG-LGLPPFADN----EHAW

PYAAPLLA-D-AS-LV--PDPATIAFAEARFAELLRVRASSPLFGLPTLDEVQRRLTFPL

S--G-PG--ETPGVLTMHLD---G------A-GLDPRWRSVTVVINAGPDPAWQTVPALI

--------GAHVALHPVLRE--S-A--DPV-LRTAAVDPAT--GTCTVPGRSVAVYVE--

-------

>UniRef90_A0A7X1LTF6_947_1797 | Pullulanase-type alpha-1,6-glucosidase n=3 Tax=Streptomyces mexicanus TaxID=178566 RepID=A0A7X1L | E_val=6.7e-194

--------------LTASKAVWIDRDTVAWN-G-V----------DGA-ASTQLLYSDDG

S-IAVKDG---TLT---SD-D----ERWIRL--E-RTT--LSDAQKARFPHLK--DYTAW

SVDPRD------RDRVR-----TALRGQLVASQRA-----A----------DGA-VLAAT

GVQIAGVLDDLYP-G---AT---KAHLGP-------VF----H-D----------G----

-----------KPTLSVWAPTA-RRVALEL-------------------GGTT-VPMRRD

DA---TGVWSV------TG-PA-S-----WK--GKEYRYVVTVWAP-----SVRKLVVNK

VTDPYSVALTAN-----SERSLVVDL---DDKALAPSGWSSL--RKP-RA-VPLRDAQIQ

ELHIRDFSVADPTVPAEDRGTYLAFTDKD---------GDGSRHLRELAKAGTSYVHLLP

AFDFAT--VPEKKA---------------DQATPGC------------------------

---DLASYP---ADSDRQQACVAKTAAQDAYNWGYDPYHFTVPEGSYATDP---DGTART

VEFRRMVQALNQDGLRVVMDVVYNHTAADGQ-ADTSVLDRIVPGYYQRLL-ADGSVADST

C--CANTAPENAMMGKLVVDSIVTWAKEYKVDGFRFDLMGHHPKANILAVRKALDALTPD

KDGVDGKKIILYGEGWNFG-EVADDARFV----QATQKNMA-GTGIATFSDRARDAVRGG

SPFDADP----GVQGFASGLYTDPNSSP--------ANGDPA-EQKARLLHYQDLIKVGL

SGNLAGYRFTD-T-GGN-EVTGAQV-DY-NG-APAGYAAAPGDALAYADAHDNETLFDAL

AYKL--------P---------------ASTGAADRARMQVLAMATATLSQGPSLSQAGT

DLLRSKSLDRNSFDSGDWFNAIHWNC--------AD-GNGFG-RGLPPAADN----QDKW

PYAGPLLT----S-VK--VGCPEIEGAFAAYRDLLRIRTTERAFSLATAAQVQSALSFPL

S--G-TH--ETPGVITMRL-------------------GDLVVVFNATPERQQQRVASLA

--------GAHYRLHPVQAR--G-A--DPV-VKTSSYEAGS--GTFTVPARTVAVF----

-------

>UniRef90_A0A430HN06_189_1051 | Alpha-dextrin endo-1,6-alpha-glucosidase n=1 Tax=Massilia atriviolacea TaxID=2495579 RepID=A0A43 | E_val=1.2e-193

----------------NAAAHWLAPDTLVWP-GT-----------PAA-ASYKLFYAADG

G-LGSAPG---GVT---GA-D-----GSIDLRV--SGA--LAPALQEKYPHLA--GSTAL

TLSSAD------VAGLP-----AKVSGQFAIAQFD-----A----------AGK-LVQVT

SLQTSGLLDALFAPA---AA---NSALGA-------TF----S-RA---------G----

-----------VPTFRVWAPTA-RSVSLNVYPDATS------------ANARS-VPMTRD

AA---SGVWHY------TASDA-A-----WTN-RAYYTYTVNVLSR----WADNRVVANV

VTDPYSLSLNAN-----GGRSFVANL---DSPTLKPPGWDLH--PIP-RL-DHPTDIALY

ELQVRDFSASDMTVPPAHRGKFLAFTDVQ---------SNGMRHLRALQRAGMSHIHLLP

SFDIAS--VNETGC-----------------TTP--------------------------

---SIPNAA---ANAEAQQAAVAASGDSDCFNWGYDPVHYSAPDGSFATDA--NDGAVRV

REFRAMVKALHEQGLRVTMDVVYNHTSGSQQ-GPLSVLDKIVPAYYYRLN-ASGAIINDS

C--CADTAAENAMMAKLMTDSVVTWATDYKVDSFRFDIMGMAPLSVITQLKEKVDRAA--

-----QRDIYLYGEAWNFG-TVGNDARFV----QARQANMF-GTGVGSFNDRLRDAVRGG

GCCDGGPE-LIGQQGFINGAFVDPNATS--------------TQTKDDLLRLGDLVKVGL

SGTLRDYSFTD-R-TGA-VRKNAQI-DY-FG-QQAGFAASPAETINYVEAHDNQTLFDLN

AFKL--------P---------------QSTSLADRVRVQNLGAAINMLSQGVPFFHAGQ

EILRSKSLDRDSYNAGDWFNRLDFTY---------Q-SNNFG-VGLPMAGVN----KANW

EFMAPILA-N-AR-IM--PDAAAIVSARDYFLDLLEIRKDSTLFRLRSARDVSERLRFHN

T--G-PQ--QVAGLIAMSIDGR--------R-YPGAKYGSVATFFNVDKVAKTITIDELK

--------GRKLGLHKVQRK--SDS--DTL-AKTATYERAS--GTFSIPPRTTAVFVE--

-------

>UniRef90_A0A7M2TC52_951_1799 | Pullulanase-type alpha-1,6-glucosidase n=1 Tax=Streptomyces chromofuscus TaxID=42881 RepID=A0A7M | E_val=2e-193

--------------LTTSKAIWIDRDTVAWN-G-S----------DAA-ASTQLLYSRTG

S-IAVEDG---TLT---GD-D----QRWLRL--S-KTS--LTDAQKAKFPHLE--AYTAW

TVDPRD------RDRVR-----EALRGQVVASQRA-----A----------NGA-VLAAT

GVQIAGVLDDLY--D---GT---KADLGP-------TF----R-H----------G----

-----------RPTLSVWAPTA-QNVSLEL-------------------DGST-VRMQRD

AA---TGVWSV------TG-PK-S-----WK--GKPYRYVVTVWAP-----SVRQVVTNK

VTDPYSLALTAD-----SARSLVVDL---GDKALAPAGWSTY--TKP-RA-VPLKDAQIQ

ELHVRDFSVEDRT--AAHKGTYLAFTDKL---------SDGSKHLRRLAEAGTSYVHLLP

AFDIAT--IPEKKS---------------DQATVDC------------------------

---DLASFP---ADSERQQECVTAIAAKDAYNWGYDPYHYTVPEGSYATDP---EGTARN

VEFRKMVKALNEDGLRVVMDVVYNHTAASGQ-AATSVLDRIVPGYYQRLL-ADGSVANST

C--CANTATENAMMGKLVVDSVVTWAKEYKVDGFRFDLMGHHPKANILAVRKALDALTPE

KDGVDGKKIILYGEGWNFG-EVADDARFT----QATQENMA-GTGVATFSDRARDAVRGG

GPFDEDP----GVQGFASGLYNEPNSSP--------GNGSTA-EQKARLLHYQDLIKVGL

SGNLAAYRFTD-T-GGR-EVTGAQV-DY-NG-RPAGYADAPGDALAYADAHDNESLFDAL

AYKL--------P---------------ATASADDRARMQVLAMATATLSQGPALSQAGT

DLLRSKSLDRNSYDSGDWFNAIHWNC--------AD-GNGFG-RGLPMAADN----RSKW

PYAKPLLG----S-VG--VGCAQIEGASAAYRDLLRIRTTEGAFSLGTAQQVQEQLSFPL

S--G-TD--ETPGVITMRL-------------------GELVVVFNATPQRQEQRVADLA

--------GSGHRLHPVQAR--G-A--DAV-VKEASYEAGS--GTFAVPGRTVAVFT---

-------

>UniRef90_A0A1M5K7K5_167_1029 | Alpha-dextrin endo-1,6-alpha-glucosidase n=1 Tax=Massilia sp. CF038 TaxID=1881045 RepID=A0A1M5K7 | E_val=4.2e-193

-----------------AKAHWLSASTVVWP-GA-----------PAAGSSYKLYYAANG

G-LGSAPQ---GVT---GA-D-----GSIDLTV--SGA--LPADLKARFPHLA--SHVAL

TMSAQS------AAALP-----AKASNQFAIAQFD-----G----------AGN-LVQVT

SLQAAGMLDSLFAAA---AA---SARLGL-------SF----D-RA---------G----

-----------VPTFRVWAPTA-KSVGLNVYASADS------------AIAST-VPMTRD

NA---SGVWSY------TAPNA-A-----WTN-SAYYTYSVNVLSR----WAGNAIVSNV

VTDPYSLSLNAN-----GKRSFVANL---DSAALKPSGWDGH--AIP-KL-DHPTDISLY

ELQVRDFSASDMTVPAGHRGKFMAFTDLE---------SNGMRHLRMLQKAGLSHIHLLP

SFDIAS--VNETGC-----------------TTP--------------------------

---VIPNVA---GNWADQQAAVAAAADTDCFNWGYDPVHYTAPDGSFATDA--NDGAVRV

REFRSMVKSLHEQGLRVVMDVVYNHTSGSQQ-GPLSVLDKIVPTYYYRLN-GSGGITNDS

C--CADTAAENAMMAKLMIDSVATWARDYQVDSFRFDIMGMAPLSVITRLKADVSQAA--

-----GRDIYLYGEAWNFG-VVGNDARFV----QARQANMF-GTGIGSFNDRLRDAVRGG

GCCDSGPD-LIRQQGFINGAYVDPNAQS--------------TQNKDDLLRLGDLIKVGL

SGTLRDYRFTD-R-FGN-LRKNSEI-DY-FG-QQAGFTASPQETINYVEAHDNQTLFDLN

AYKL--------P---------------QGTSRADRLRVQNLGAAINMLSQGIPFFHAGQ

EIMRSKSMDRDSYNAGDWFNKLDYSY---------Q-SNNFG-VGLPMQGVN----GSNW

ELMSPILV-N-PL-IK--PDSASIVAARDYFVDLLQIRSDTSLFRLRSAADVNQRVKFHN

T--G-PQ--QVAGVIAMTIDGV--------G-YQHAKYKQLAVFFNVDKEARDITIPELR

--------NQIFAVHKVQRN--SSS--DAL-AKTATYNRAS--GTFRIPPRTTVVFVS--

-------

>UniRef90_A0A1M4RWU0_1147_1951 | Glycosyl hydrolase all-beta n=12 Tax=Actinomyces TaxID=1654 RepID=A0A1M4RWU0_9ACTO | E_val=8.8e-193

------------------------------------------------------------

------------------------------------------------------------

--------------EVA-----EALKGQVAVVQHG-----A----------DGTGATAFT

GVQTAVVIDSLYADA---VA---DAPLGV-------TF----V-G----------G----

-----------VPDFALWAPTA-QAVTLLTWDTGTATGSA----ALVDGDPVR-TAAV-R

GD---DGRWTV------DG-AD-AGI---TA--GSQYLWEVKVYAP-----STGKVETNL

VTDPYSVGLTLG-----SARSVAVDL---SDPDLAPEQWTST--ASP-LV-SSDAARTIY

ELHVRDFSAADATVPEEYRGTYKAFTVTD---------SDGMQHLAELADAGIDTVHLLP

TFDIAS--IEEDRS---------------AQQVPDI------------------------

---P-ADAG---PDSTEQQAAVGAVKDADAYNWGYDPYHYTTPEGSYATEG-NQDGGART

YEFREMVGALHATGLQVVLDQVFNHTAGACQ-DTTSVLDRVVPGYYQRLD-AKGDVLTST

C--CPNTATENALAERLMIDSVVTWARDYHVDGFRFDLMGYHSVDTMNRLRAALDELTVA

KDGVDGSAIYLYGEGWNMG-EIADNALFT----TATQGQLD-GTGIGTFNDRLRDAVHGG

GPFDDDPR---TYQGFGTGLYTAPNGLS---------NKTEE-EQLADLGHATDLVKLGL

AGNLKDYSFTQ-S-DGT-VKRGAEV-DY-NG-QAAGYASSPEETINYVDAHDNETLYDLG

VYKL--------A---------------ADTSMADRVRMNSLSLATVTLGQSPSFWAAGT

EMLRSKSLDRDSYNSGDYFNAIDWTG---------Q-DNGFG-AGLPMESSN----GSKW

DIQRPLLA-D-PA-LK--PTAEDIAASNAQMLDFLRIRTSTPLFHLGSADAIQAKLSFPN

S--G-TD--ATAGVIDMLIDDTVG------D-DVDASLAGVLVVFNASDTEVSETITDLA

--------GREFVLHQVQAD--G-S--DAV-VKDSTFDAAT--GTATVPARTVAVYVEKQ

-------

>UniRef90_A0A2M9K046_920_1773 | Alpha-dextrin endo-1,6-alpha-glucosidase n=3 Tax=unclassified Streptomyces TaxID=2593676 RepID=A | E_val=1.5e-192

--------------LAKSEAQWIDRDTVALP-ADG----------TAA-LSAQLVYDRDG

G-ITVKDG---ALS---SE------GQWLRL--N-KAAGGLTDAQLARFPQLK--KYDAY

TVDPRD------RDRVG-----DALKSQLILTRRI-----A----------NGA-LVTAT

GVQTAGVLDDLYAQK---AG---KASLGP-------VF----T-K----------S----

-----------GVTLSVWAPTA-QSVALDL-------------------DGRA-VAMRRD

GV---TGVWSV------KG-PR-S-----WQ--GKQYRYAVKVWAP-----SVQKVVTNK

VTDPYSTALTAD-----SERSLVVDL---DARNLAPSGWSAL--KKP-KA-VPLRDAQIQ

ELHIRDFSVEDRT--AKHPGTYLAFTDKD---------SKGSEHLRVLAKAGTSYVHLLP

AFDIAT--IPERKS---------------EQATPDC------------------------

---DLNSFG---AASDRQQECVGKGAAKDAYNWGYDPYHYTVPEGSYASDP---DGTART

VEFRQMVESLNVDGLRVVMDVVYNHTAASGQ-AKTSVLDRIVPGYYQRLL-ADGSVATST

C--CANTAPENAMMGKLVVDSIVTWAKEYKVDGFRFDLMGHHPKANILAVRKALDALTVE

KDGVDGKKIILYGEGWNFG-EVADDARFV----QATQKNMA-GTGIATFSDRARDAVRGG

SPFDADP----GVQGFASGLYTDPNSSK--------ANGTEA-EQKARLLHYQDLIKVGL

TGNLAAYTFTG-S-DGR-TVKGADV-DY-NG-APAGYADAPGDALAYADAHDNESLYDAL

TYKL--------P---------------ASTSPADRSRMQVLALATAALSQGPALSQAGT

DLLRSKSLDRNSFDSGDWFNAVHWDC--------RD-GNGFG-RGLPPAADN----KDKW

PYATPLLT-SVPA-----AKCADIEGTSAAYRDLLKIRTTEPAFGLATAGQVQEKLSFPL

S--G-KD--ETPGVITMRL-------------------GDLVVVFNATPRTQEQRVGAVA

--------GTSYRLHPVQAA--G-A--DSA-VKSASYEAGS--GTFTVPARTVAVFT---

-------

>UniRef90_A0A542Q1V2_913_1761 | Alpha-dextrin endo-1,6-alpha-glucosidase n=1 Tax=Streptomyces sp. SLBN-118 TaxID=2768454 RepID=A | E_val=2.5e-192

---------------AKAEAQWIDRDTVVWK-VKA----------TDA-TSQQLVYAANG

G-ISVVEG---ALS----D-E----GRWLRL--N-PAA--LTDAQKAAHPHLA--AYPAF

SVDPRD------RERVR-----DALRGQLIATQRA-----A----------NGA-LLAAT

GVQIPGVLDDLY--D---AT---GAKLGP-------VF----D-R----------G----

-----------RPTLSLWAPTA-RSVSLEL-------------------DGTT-VAMRRS

GT---SGVWSV------TG-PK-S-----WA--GKPYRYVVKVWAP-----SVQKVVTNK

VTDPYSTALTTD-----SARSLVVDL---ADPRLAPKGWATL--KKP-AA-VPLRDAQIQ

ELHIRDFSVADRT--SRHPGQYLAFTDRA---------SAGMKHLRALAASGTSYVHLLP

AFDIGT--IPERKS---------------DQTVPAC------------------------

---DLKVYA---PDSDEQQACIAKAAAKDAYNWGYDPLHYTVPEGSYASDP---EGTRRT

VEFRQMVASLNEGGLRTVMDVVYNHTVASGQ-ADKSVLDKIVPGYYQRLL-ADGTVATST

C--CANTAPENAMMGKLVVDSVVTWAKEYKVDGFRFDLMGHHPKANILAVRKALDALTVE

RDGVDGKKIILYGEGWNFG-EIADDARFV----QATQKNMA-GTGIATFSDRARDAVRGG

GPFDEDP----GIQGFASGLFTDPNSSA--------ANGTPA-EQKARLLHYQDLIKVGL

TGNLAGYTFTD-S-SGR-TVKGSEV-DY-NG-APAGYAAAPGDALAYADAHDNESLYDAL

AFKL--------P---------------RATSPADRARMQVLAMATAALSQGPALSQAGT

DLLRSKSLDRNAYDSGDWFNAIHWDC--------RD-GNGFG-RGLPPQADN----KSKW

PFAKPLLT-A-PS-LA--EGCEGIDGASAAYRDLLSIRATEKAFSLASADDVQSTVSFPL

S--G-KE--ESPGVITMAL-------------------GDLVVVFNATPDTQTQRAEAFA

--------GRSYALHPVQAT--G-A--DLT-VKKASFEGSS--GTFAVPGRSVAVF----

-------

>UniRef90_A0A124C5K4_148_999 | Alpha-amylase n=4 Tax=Streptomyces scabiei TaxID=1930 RepID=A0A124C5K4_STRSC | E_val=4.3e-192

--------------LTTSKAVWIDRNTVAWN-G-N----------DTA-ASTQLLYSRSG

S-ITAKDG---ALT---ST-D----ERWLRL--T-KAA--LTDAQKARFPHLK--AYTAW

TVDPRD------RDRVR-----EALRGQLVASQRA-----A----------NGA-VLTAT

GVQVAGVLDDLYDGS---A-----ADLGP-------TF----R-K----------G----

-----------RPTLAVWAPTA-QSVKLEL-------------------DGKT-LPMKRD

DA---TGVWSV------TG-EK-S-----WK--NKPYRYVVKVWAP-----SVRKIVTNK

VTDPYSVALTAD-----SERSLVVDL---TDRSLAPSGWSSY--TKP-KA-VPLKDAQIQ

ELHVRDFSIGDATVPAKDRGTYLAFTGKG---------SDGSRHLRHLAKAGTSYVHLLP

VADIAT--IPERKA---------------DQATVDC------------------------

---DLASYP---ADSEKQQECVAKAAAKDGFNWGYDPYHYTVPEGSYATDP---DGTRRT

VEFRKMVRALNADGLGVVLDVVYNHTPASGQ-ADRSVLDKVVPGYYHRLL-ADGSVATST

C--CANTAPENTMMGKLVVDSIVTWAKEYKVDGFRFDLMGHHPKANILAVRKALDALTPE

KDGVDGKKIILYGEGWNFG-EVADDARFV----QATQKNMA-GTGVATFSDRARDAVRGG

GPFDEDP----GVQGFASGLWTDPNPAA--------DNGTPA-EQKARLLHYQDLIKVGL

SGNLANYRFTD-T-DGK-EVQGSEV-DY-NG-TAAGYADAPGDALAYVDAHDNESLFDAL

AFKL--------P---------------KSTSPADRARMQVLALATAALSQGPALSQAGT

DLLRSKSLDRNSYDSGDWFNAIHWDC--------RD-GNGFG-RGLPLAADN----RSKW

PYAKPLLT----S-VK--VGCEQIEGASAAYRDLLRIRTTEDVFSLDTAGQVQSKLSFPL

S--G-KD--ETPGVITMEL-------------------GDLVVVFNATPERRKQTVDDLA

--------GTGYALHRVQAA--G-A--DST-VRTSSYEAES--GTFVVPGRTVAVFAR--

-------

>UniRef90_A0A120GAK0_87_943 | Aamy domain-containing protein n=2 Tax=Erythrobacter sp. YT30 TaxID=1735012 RepID=A0A120GAK0_9SP | E_val=7.3e-192

--------DAQQAQPLYAQAHWLTRDLIAWDA-------------PDA-TKFAIRVSLNA

D----------------GQ--------YVERELE-TAG-TVDGTMADAHPHLR--GMPLW

RITDLS------EQEAK-----AYLKGATEFLAYD-----A----------ENV-LAART

RPQIGWVLDRLYAN---------DAELGA-------RF----I-G----------D----

-----------DLTIAVWAPTA-RSLRLRLFDTSNG------------DTATV-LPMEED

PQ---TGVWSI------KG-AS-E-----WN--RKYYIYEVDVYVP-----EEGRIVTNL

VTDPYSLNLAGN-----STRTQIIDL---SDAELKPSGWDSF--ARD-LP-EAPEDRVFY

ELHVRDFSIGDNTVEADARGRYAAFTDRD---------STGNRHLRALAEAGLSDVHLLP

SYDCAT--IPERHV---------------EQASPP-------------------------

---DLNAFP---ANSEEQQAAIAAIADRDGFNWCYDPWHYMAPEGSYSSDP---DTTARI

KEFRAMVMGLSESGLGTVLDVVFNHTMASGQ-DRQSVLDRIVPGYYHRLD-ENGAVAEST

C--CANTATERRMMERLMLDSLEVWAREYKVSGFRFDLMGHHTRDNILNVRALLDGLSLA

KDGVDGPDVYVYGEGWNFG-EVVNDTRFV----QATQGNMGQGTGIGTFNDRIRDAIRGG

GVGDKGET-TIAAQGFASGLFTAPNALT----------EDDE-NARAKALKVADQIRASL

AGSIANYSIQT-A-DGI-TKTAAEI-SY-GG-GPFAYASDPQEVINYTEAHDNQTLFDSN

AFKL--------P---------------RNISKAERVRWQNIATSIIMLSQGIPFIHAGQ

ELLRSKSLDHNSYNSGDWFNRLDFEM---------E-HNGWG-RGLPPAPDN----KAEW

DFGRELLA-D-DR-FR--MGTKEIALANAHLRELLQIRQSSLLFRLKTAEDVVANVHFDN

T--G-PD--QTLGLIVMRLGLEGE--------------EQVMVVINATRDPQTVAAPS--

--------GQTYELHKVQRA--S-V--DAT-LRDTVFRA----GEISVPALTTAVFVR--

-------

>UniRef90_UPI0016771D90_950_1800 | pullulanase-type alpha-1,6-glucosidase n=2 Tax=Streptomyces TaxID=1883 RepID=UPI0016771D90 | E_val=1.4e-191

--------------LTTSKAVWIDRDTLVWD-G-S----------EAA-ASTQLLASRTG

S-VTVRDG---TLR---SG-D----ARWLRL--T-RTA--LTDAQKAKFPHLA--RGTAW

SVDPRD------RDRVR-----EALRGQVVATQHA-----A----------NGA-VLAAT

GVQTAGVLDDLYAAG---AT---KARLGP-------VF----R-G----------G----

-----------RPTLSVWAPTA-RNVSLEI-------------------GDRT-VRMRRD

DT---TGVWSV------TG-PP-S-----WK--GKEYRYKVEVWAP-----STREITTNT

VTDPYSVALTTD-----SERSLVVDL---ADRRLAPKGWSSL--AKP-KA-VPLKDAQIQ

ELHVRDFSVEDRT--AKNPGTYLAFTDKD---------SDGSRHLRELAESGTSYVHLLP

AFDIAT--IPEKKS---------------DQTRVDC------------------------

---DLASYP---ADSAEQQKCVAKAAAKDAYNWGYDPYHYTVPEGSYATDP---DGTART

VEFREMVKALNEEGLGVVMDVVYNHTASHGQ-AKTSVLDRIVPGYYQRLL-ADGSVANST

C--CSNTAPENAMMGKLVVDSVVTWAREYKVDGFRFDLMGHHPKANMLAVREALDALTPE

KDGVDGKKIILYGEGWNFG-EIADDVRFE----QATQKNMA-GTGIATFSDRARDAVRGG

GPFDEDP----GVQGFASGLYTDPNSSK--------GNGTEA-EQKARLLHYQDLIKVGL

SGNLAGYTFTD-T-SGK-EVKGSQV-DY-NG-APAGYAAVPGDALAYADAHDNESLFDAL

AFKL--------P---------------EDTPAGDRARMQVLAMATATLSQGPALSQAGT

DLLRSKSLDRNSYDSGDWFNAIHWNC--------AA-GNGFG-RGLPPAADN----EDKW

SYAKPLLG----S-VR--VGCPQILGTTAAYQDLLRIRTTEKVFSLDTAAQVQSRLSFPL

S--G-KD--ETPGVITMRL-------------------GDLVVVFNATPQRQEQRVGTLA

--------GTGYRLHPVQAA--G-G--DAV-VKTSSYAKGT--GTFTVPARTVAVFT---

-------

>UniRef90_A0A221NW98_945_1794 | Alpha-dextrin endo-1,6-alpha-glucosidase n=2 Tax=Streptomyces TaxID=1883 RepID=A0A221NW98_9ACTN | E_val=2e-191

--------------LTTSKAIWIDRDTLAWN-G-I----------DGA-ASSQLLYSRDG

S-ITVKDG---TLT---SD-D----ERWLRL--T-KTT--LSEAQKAKFPHLK--DYTAW

SVDPRD------RDRVR-----RALRGQLVASQRA-----A----------NGA-VLAAT

GVQIAGVLDDLYP-K---AT---KAALGP-------VF----H-H----------G----

-----------RPTLSVWAPTA-RSVRLDL-------------------DGRT-VRMHRN

RT---TGVWSV------TG-PR-S-----WK--GKPYRYVVKVWAP-----SVRKMVTNK

VTDPYSVALTAD-----SERSIVADL---DDRSLAPRGWSKL--RKP-KA-VPLKDAQIQ

ELHIRDFSIGDPT--SKHPGTYLAFTDKN---------SDGSRHLRALARAGTSYVHLLP

AFDIAS--IPEKKA---------------DQARPDC------------------------

---DLASYP---ADSAKQQECVAKTAAKDAYNWGYDPYHYTVPEGSYATDP---DGTART

VQFRKMVQALNQDGLRVVMDVVYNHTAASGQ-ADTSVLDKIVPGYYQRLL-ADGSVANST

C--CANTAPENAMMGKLVVDSIVTWAKEYKVDGFRFDLMGHHPKANILAVRKALDALTLK

KDGVDGKKIILYGEGWNFG-EVANDARFV----QATQANMA-GTGIATFSDRARDAVRGG

GPFDSDP----GIQGFGSGLYTDPNSST--------ANGTRA-EQKARLLHYQDLIKIGL

TGNLAKYRFTD-D-DGR-EVTGAEV-DY-NG-SPAGYADAPGDALAYVDAHDNESLFDAL

TYKL--------P---------------KDTSASDRARMQVLAMATAALSQGPSLSQAGT

DLLRSKSLDRNSYDSGDWFNAIHWNC--------AD-GNGFG-RGLPMAADN----ASKW

TYAKPLLG----T-VK--VGCAQINGASAAYQDLLKIRTTERAFSLGTTARVQDQLSFPL

S--G-KD--ETPGVITMRL-------------------GDLVVVFNATPQKQEQRLDAVA

--------GTHYRLHPVQAR--G-A--DPV-VKASSYAAGS--GTFTVPARTVAVFT---

-------

>UniRef90_UPI0006E1E0A0_957_1809 | pullulanase-type alpha-1,6-glucosidase n=3 Tax=Streptomyces hirsutus TaxID=35620 RepID=UPI000 | E_val=4e-191

-------------DLATSKAVWIDRNTVVWN-G-S----------ASA-ASTQLLASRTG

S-VKAQDG---TLV--VGD-D----TRVIRL--L-PAA--LTDAQKERFPHLK--DGTAH

SVDPRD------RDRVR-----EALRGQVVAAQRA-----A----------NGA-VLAAT

GVQIAGALDDLYAAG---AT---KAELGP-------VF----R-D----------G----

-----------RPTLSVWAPTA-QSVSLEI-------------------GGST-ARMRRD

DA---TGVWSV------TG-PK-S-----WK--GKPYRYAVQVWAP-----STRELVTNK

VTDPYSVALTTD-----SARSLVVDL---GDRDLAPKGWSGL--DKP-KA-VPLKDAQIQ

ELHIRDFSVEDRT--AKQPGTYLAFTDKD---------SNGSRHLRKLAEAGTSYVHLLP

AFDIAT--IPEKKS---------------DRATVDC------------------------

---DLAAHP---ADSDKQQECVAKAAAKDAYNWGYDPYHYTVPEGSYATDP---DGTART

VEFREMVKALNEDGLRVVMDVVYNHTAASGQ-SGTSVLDRIVPGYYQRLL-ADGSVANST

C--CANTAPENAMMGKLVVDSIVTWAKEYKVDGFRFDLMGHHPKANILAVRKALDALTLK

KDGIDGKKIILYGEGWNFG-EIADDVRFE----QATQQNMA-GTGIATFSDRARDAVRGG

GPFDEDP----GVQGFASGLYTDPNPSE--------NNGTEA-EQKARLLHYQDLIKVGL

SGNLAEYRFTD-T-DGK-EVKGSQV-DY-NG-APAGYAAAPGDALAYADAHDNESLFDAL

AFKL--------P---------------TSVSADDRARMQVLAMATAALSQGPALSQAGT

DLLRSKSLDRNSYDSGDWFNAIHWNC--------AD-GNGFG-RGLPPAADN----ADKW

PYAKPLLG----A-VK--VGCPQIEGASAAYRDLLRIRTTEGAFSLDTAGQVQSALSFPL

S--G-KD--ETPGVITMRL-------------------GDLVVVFNATPDRQEQRVGALA

--------GTDHRLHPVQAA--G-S--DAV-VKTSSYAADT--GTFTVPARSVAVFT---

-------

>UniRef90_A0A2G9DW20_967_1815 | Alpha-dextrin endo-1,6-alpha-glucosidase n=4 Tax=Streptomyces TaxID=1883 RepID=A0A2G9DW20_9ACTN | E_val=1e-190

-----------------SKAIWIDRNTLAWN-G-S----------EAA-ASTQLLSSRTG

S-IKVKDG---TLS---ST-D----ERWIRL--T-KSS--LTDAQKARFPHLK--AYPAW

TVDPRD------RDRVR-----EALRGQLVASQRA-----A----------NGA-VLAAT

GVQIAGVLDDLY--D---GS---DADLGP-------TF----R-D----------G----

-----------RPTLAVWAPTA-QQVSLDL-------------------DGTK-VAMKRN

GA---TGVWSV------TG-PK-S-----WK--GKPYQYTVKVWAP-----SVGKVVTNK

VTDPYSVALTTD-----SERSLVVDL---ADRSLAPRGWTSY--DKP-KA-VPLKDAQIQ

ELHIRDFSIADRTVPAKDQGTYLAFTDKD---------SAGSKHLRQLAKAGTSYVHLLP

AFDIAT--IPERKA---------------DQATPDC------------------------

---DLGSYT---ADSEQQQECVTKAAAKDAFNWGYDPYHYTVPEGSYATDP---NGTRRT

VEFREMVKSLNDEGLRVVMDVVYNHTTASGQ-ARTSVLDKVVPGYYQRLL-ADGSVATST

C--CANTAPENTMMGKLVVDSIVTWAKEYKVDGFRFDLMGHHPKANILAVRKALDALTPA

KDGVDGKKIVLYGEGWNFG-EVADDARFT----QATQKNMA-GTGIATFSDRARDAVRGG

GPFDEDP----GVQGFASGLYTEPNSSG--------SNGTPA-EQKSRLLHYQDLVKVGL

SGNLANYRFTD-T-SGK-EVKGSEV-DY-NG-SAAGYADAPGDALAYVDAHDNESLFDAL

AFKL--------P---------------KSTSAADRSRMQVLAMATAALSQGPALSQAGT

DLLRSKSLDRNSYDSGDWFNAIHWDC--------RD-GNGFG-RGLPPATDN----KAKW

SYAKPLLT----S-VS--VGCDQIEGASAAYRDLLRIRSTEGDFSLDTTGQVQSKLSFPL

S--G-KD--ETPGVITMEL-------------------GDLVVVFNATPEKAKQTVTDLA

--------GTAYALHPVQAE--G-A--DST-VKSASYAAES--GTFAVPGRTVAVFAR--

-------

>UniRef90_A0A1H8QFL7_176_1038 | Alpha-dextrin endo-1,6-alpha-glucosidase n=2 Tax=Duganella sp. CF517 TaxID=1881038 RepID=A0A1H8Q | E_val=1.9e-190

-----------------ASAHWLSATTFAWP-GV-----------PAA-GTYKLYYAANG

G-LGSNPA---GVT---GA-E-----GSIALNA--VGA--LSAALREKYPHLA--GATAL

TMSSTD------AAGIA-----ARAGNQFAIAQFD-----A----------SGK-LIQVT

SLQTSGMLDDVFAGA---AA---NATLGL-------SY----N-RN---------G----

-----------APTFRVWAPTA-KAVSLNLYPDANS------------ASTAT-RAMTRD

GA---SGVWSY------TAPDA-S-----WVN-RYYYTYNVQVLSR----WAGNTVVTNT

VTDPYSLSVSAN-----SQRSFVADL---DSRALKPGGWDHH--RIP-RL-DQPTDIALY

ELHIRDFSASDASVPAAHRGKYLAFTDDG---------SNGMRHLRELRKAGLTHIHLLP

SFDYAS--VNETGC-----------------TTP--------------------------

---SIPNAA---PNAPDQQAAVEAARGADCFNWGYDPVHYTAPEGSYATNA--NDGATRV

REFRAMVQGLHEQGLRVVMDVVYNHTSGSQQ-GPLSVLDKIVPTYYYRLN-AGGGITNDS

C--CADTASENAMMAKLMIDSVSTWARDYKVDSFRFDIMGFTPLSVMKRLQAAVNTAA--

-----QRDIYLYGEAWNFG-TVGNDARFV----QARQANMY-GSGIGSFNDRLRDAVRGG

GCCDSGAS-LIDQQGFINGAFYDPNARS--------------TQTKDDLLRLGDLVKVGL

SGTLRDYAFTD-R-TGA-WRKNWQI-DY-SG-QQAGFAANPAETINYVEAHDNQTLFDLN

AYKL--------P---------------QATSMADRVRVQNLGAAINILSQGVPFLHAGQ

EILRSKSMDRDSYDAGDWFNKLDYSY---------Q-SNGFG-IGLPMAGVN----QDNW

PLMSPILA-N-PL-IK--PDTGAIVAARDYVADMLEIRQDSALFRLRSGDEVAKRLRFYN

T--G-PQ--QIPGLIVMSLDGRVP-----LR-GGDAKYKSVVVLFNVDKVAKTIAVPELA

--------GRSLALHHVLRK--A-G--GGR--GIADHDSVG--GRFTIAARATAVFVE--

-------

>UniRef90_A0A2A9CX41_1235_1966 | Pullulanase-type alpha-1,6-glucosidase n=2 Tax=Serinibacter salmoneus TaxID=556530 RepID=A0A2A9C | E_val=5.2e-190

------------------------------------------------------------

------------------------------------------------------------

------------------------------------------------------------

------------------------------------------------------------

-------------TATVWAPTA-QSVELVRWDGAT-------------GSETV-LTADRD

DA---AGSWSA------GP-LA-A---------GDAYRWRITVYAP-----ATGAIETNE

VTDPYSVALTTN-----SVASVAVDL---SDPALAPSEWAET--ASP-VV-ARDVDQTIW

ELHVRDFSIGDETVPEADRGTYRAFTHTD---------SAGMTHLRELAEAGITTVHLLP

TFDIAS--IEEDPE---------------AQAAPEC------------------------

---DLAGMA---PDSADQQECVGAIRGEDGYNWGYDPYHFMAPEGSYATSP---EGTERV

SEFREMVGALHAADLQVVLDQVYNHTAASGQ-APTSVLDRVVPGYYHRLN-AAGGVETST

C--CQNIATEHEVAERLMVDSVVLWARDYKVDGFRFDLMGHHSLANMQAVREALDALTLE

EDGVDGSAVYLYGEGWDFG-EVAGDALFE----QARQANLA-GTGIGSFNDRLRDGVHGG

SPVDGSST---FDQGFGTGLGTDPNGRS--------RTGA------EDLGHLTDLVKLGM

AGNLAEYSFTA-S-TGE-TVTGAEV-DY-NG-SPAGYAGQPSETINYVDAHDNETLFDLA

VFKL--------P---------------TDTPMDERVRANTVQLATVALGQSPSFWHAGT

ELLRSKSLDRDSYDSGDWFNRIDWTG---------Q-ENTFG-SGLPGAWSN----EEKW

DLMAPLLA-D-AS-LK--PDAAAMASSYAQALDLLRVRSSVDLLRLGSTQAVQEKVTFPG

S--G-PD--EAPGTIAMLIDDTVG------S-PASESLDGALVLINASPDAVTRSFPELA

--------GREFVLNQVQAE--G-S--DDV-VTSVSL-TAE--GSVEVPARSYVVLVEEA

E------

>UniRef90_A0A5Q0LJJ3_948_1798 | Alpha-dextrin endo-1,6-alpha-glucosidase n=2 Tax=Streptomyces TaxID=1883 RepID=A0A5Q0LJJ3_9ACTN | E_val=1.3e-189

--------------LTTSKAVWIDRNTVAWN-G-S----------DAA-VSTQLLSSHDG

S-IAVKDG---GLT---SD-D----ERWLRL--G-KTT--LTDAQKAKFPYLK--AYTAW

SVDPRD------RARAR-----SALGGQLVASQRA-----A----------NGA-VLAAT

GVQIAGALDDLY--D---AT---KAHLGP-------VF----H-D----------G----

-----------VPTLSVWAPTA-QAVSLDL-------------------DGST-VAMKRD

GA---TGVWSV------TG-KK-S-----WT--NKPYRYVVKVWAP-----SVRKIVTNK

VTDPYSVALTAN-----SERSLVVDL---DAKSLAPSGWSSL--KKP-RA-VPLRDAQIQ

ELHVRDFSVADKTVPAKDQGTYLAFTDKN---------SDGSKHLKQLAKSGTSYVHLLP

AFDIAT--VPEKKA---------------DQQSTDC------------------------

---DLASYA---ADSDKQQECVAKTAAKDAYNWGYDPYHYTVPEGSYATDP---DGTGRT

VEFRRMVKSLNEDGLRVVMDVVYNHTAASGQ-ADTSVLDQVVPGYYQRLL-ADGSVANST

C--CANTATENAMMGKLVVDSIVTWAKEYKVDGFRFDLMGHQPKANILAVRKALDGLTPA

KDGVDGKKIILYGEGWNFG-EVADDARFV----QATQKNMA-GTGIATFSDRARDAVRGG

SPFDSDP----GVQGFASGLYTDPNSST--------ANGTTA-EQKARLLHYQDLIKVGL

TGDLAAYRFTG-T-DGK-EVEGSEI-DY-NG-QPAGYAAAPGDALAYADAHDNESLYDAL

AYKL--------P---------------ATTSADDRARMQVLAMATATLSQGPSLSQAGS

DLLRSKSLDRNSFDSGDWFNAVHWDC--------RD-GNGFG-RGLPMAADN----QSKW

PYATPLLS----T-VK--VGCGQIQGSSAAYQDLLRIRTTESVFSLATAGQVQSKLSFPL

S--G-KD--ETPGVITMKL-------------------GDLVVVLNATPQRQEQRIGSLS

--------GTRYALHPVQRA--G-A--DPV-VKSAAYTKDT--GTFTVPARTVAVFS---

-------

>UniRef90_UPI000A6A66DA_229_1082 | pullulanase-type alpha-1,6-glucosidase n=4 Tax=Streptomyces TaxID=1883 RepID=UPI000A6A66DA | E_val=2.8e-189

--------------LTTSKAVWIDRNTVAWN-G-N----------DTA-ASTQLLSSRTG

S-ITAKDG---ALT---ST-D----ERRLRL--T-KSA--LTDAQKARFPHLK--SYTAW

TVDPRD------RDRVR-----EALRGQLVASQRA-----A----------TGA-VLAAT

GVQIAGVLDDLYGRS---AA---DADLAP-------TF----R-K----------G----

-----------RPTLAVWAPTA-QGVSLDL-------------------DGRT-LPMKRD

DA---TGVWSV------TG-PK-S-----WK--NKPYRYVVKVWAP-----SVREVVTNK

VTDPYSVALTAD-----SERSLVVDL---ADRSLAPSGWSSY--TKP-KA-VPLKDARIQ

ELHVRDFSIADKAVPAKDKGTYLAFTDKG---------GDGSKHLRKLAKAGTSYVHLLP

VADIAT--IPERKA---------------DQASVDC------------------------

---DLGSYA---ADSDQQQECVAKAAAKDGFNWGYDPYHYTVPEGSYATDP---DGTRRT

VEFRKMVKALNGDGLGVVLDVVYNHTPASGQ-ADKSVLDKVVPGYYHRLL-ADGSVATST

C--CANTAPENTMMGKLVVDSIVTWAKEYKVDGFRFDLMGHHPKANILAVRKALDALTPK

KDGVEGKKIILYGEGWNFG-EVADDARFT----QATQKNMA-GTGVATFSDRARDAVRGG

GPFDEDP----GVQGFASGLYTEPNSSK--------DNGTPA-EQKARLLHYQDLIKVGL

SGNLANYRFTD-T-DGK-EVKGSEV-DY-NG-AAAGYADAPGDALAYVDAHDNESLFDAL

AFKL--------P---------------KSTSAADRSRMQVLAMATAALSQGPALSQAGT

DLLRSKSLDRNSYDSGDWFNAIHWDC--------RD-GNGFG-RGLPLAADN----ESKW

PYAKPLLT----S-VG--VGCEQIEGASAAYRDLLRIRTTEDVFSLDTAGQVQSELSFPL

S--G-KD--ETPGVITMEL-------------------GDLVVVFNATPERRKQTVDDLA

--------GTGYVLHQVQAA--G-A--DST-VRTSSYEAES--GTFVVPGRTVAVFAR--

-------

>UniRef90_UPI0009A10F2F_76_914 | pullulanase-type alpha-1,6-glucosidase n=1 Tax=Streptomyces oceani TaxID=1075402 RepID=UPI000 | E_val=4.7e-189

------------------RAQWIDRETIAWD-VKT----------PED-RHAQLDYAR--

-------------------------QERLRLNPV-EGG--LTRQQREEYPHLA--DYTAF

TVHPSD------RDQVR-----QALRGRLVVTQRD-----T----------SGE-VLEST

GVQIPGVLDDLYGTE---AA---EAELGP-------VFD---R-H----------G----

-----------RPTLSVWAPTA-QRVTLEL-------------------DGEP-VRMRRD

DS---TGVWSV------RG-DR-A-----WR--GKEYRYQVRVWAP-----SVRKNVTNK

VTDPYSTALTAN-----SKRSVVTDL---DAARLKPPGWESG--AKP-EA-TALSDARIQ

ELHVRDFSVADES--ARHRGEYRAFTERD---------SDGMRHLRKLAKAGTSYVHLLP

TYDIAS--IPEREK---------------DQRQPDC------------------------

---DLAALP---AASVEQQKCVGEVRDQDAYNWGYDPLHYTVPEGSYASDP---DGPRRT

LEYREMVQGLNRAGLRVVLDVVYNHTFAAGQ-DERSVLDRIVPGYYQRLL-KDGSVADST

C--CANTAPEHTMMGKLVVDSVVTWAREYKVDGFRFDLMGHHPKANILAVREALDELTLA

EDGVNGEDIIMYGEGWNFG-EVADDARFE----QATQANMA-GTGVATFSDRARDAVRGG

APFDEDP----RVQGFGSGLYTDSNGSG--------SNGTRE-EQKRRLLHYQDLIKVGL

TGNLADYTFTD-S-TGE-RVRGSEV-DY-NG-APAGYAKSPRDAVAYADAHDNESLYDAL

AYKL--------P---------------ADTSAADRARMQVLAMGTAALSQGPALSQAGS

DRLRSKSLDRNSYESGDWFNAIHWDC--------RD-GNGFG-RGLPPSWDN----KDKW

PYAKPLLA-R-DN-LT--PSCSETTAASAAYRDLLRIRTSEEALSLDTAAEVQREVGFPI

SG-G-EQ--QTPGVMVMTA-------------------GDLVVVINATPEARRQPVEELV

--------GGDYALHPVQRS--G-S--DEV-VKAARYDSAD--GVFEVPARTVAVFR---

-------

>UniRef90_A0A1S2PW21_962_1810 | Alpha-dextrin endo-1,6-alpha-glucosidase n=1 Tax=Streptomyces colonosanans TaxID=1428652 RepID=A | E_val=1e-188

----------------TSKAIWIDRNTLAWS-G-A----------DGA-ASTQLVYSHEG

S-IAVKGG---TLT---SD-D----ERWLRL--T-RTT--LTDAQKAKFPHLK--DYTAW

SVDPRD------RDRVR-----QALGGQIVASQRA-----A----------NGA-VLAAT

GVQIAGVLDDLY--D---AT---KADLGP-------TF----Q-G----------G----

-----------RPTLALWAPTA-QNVSLEL-------------------DGST-VAMHRD

AA---TGVWSV------TG-PA-S-----WK--NKPYRYSVTVWAP-----SVRKIVTNK

VTDPYSLALTAG-----SERSLVVDL---KDKSLAPGGWSTL--KKP-NA-VPLRDAQIQ

ELHIRDFSAADKTVPAKDQGTYLAFTDKN---------SDGSRHLRDLAAAGTSYVHLLP

AFDIAT--IPEKKS---------------EQATPDC------------------------

---DLASYP---ADSDRQQECVSATAAKDAYNWGYDPYHYTVPEGSYATDP---NGTDRT

VEFRKMVKALNEDGLRVVMDVVYNHTAAAGQ-ADTSVLDKVVPGYYQRLL-ADGSVANST

C--CANTAPENAMMGKLVVDSIVTWAREYKVDGFRFDLMGHHPKANILAVRKALDALTPA

KDGVDGKKIILYGEGWNFG-EVANDARFE----QATQQHMA-GTGIATFSDRARDAVRGG

GPFDEDP----GIQGFASGLYTDPNPAA--------DNGTTA-EQKARLLHYQDLIKVGL

TGNLAAYRFTD-T-DGK-EVTGSEV-DY-NG-APAGYADAPGDALAYADAHDNESLFDAL

AFKL--------P---------------KNTSAAGRARMQVLAMATAGLSQGPSLSQAGS

DLLRSKSLDRNSYDSGDWFNAIHWDC--------RD-GNGFG-RGLPMAADN----RSKW

DYAGPLLT----S-VK--VGCDQIDGASAAYRDLLRIRTTEPAFSLGTTGQVQSQLSFPL

S--G-KN--ETPGVITMEL-------------------GDLVVVFNATPGKQQQTVGALA

--------GKHYALHPVQSA--G-T--DPV-VKTSSYAAES--GTFTVPGRTVAVFA---

-------

>UniRef90_A0A4S2TUS4_916_1771 | Alpha-dextrin endo-1,6-alpha-glucosidase n=5 Tax=unclassified Streptomyces TaxID=2593676 RepID=A | E_val=2e-188

----------PTPDLTKAEAQWIDADTVVWK-VKA----------TEA-TSQQLVYAEKG

G-ISVVDG---ALS----D-E----GRWLRL--T-PSA--LSDAQKAKYPHLK--GYPAF

TVDARD------RDRVR-----EALRGQLIATQRA-----A----------NGA-LLAAT

GVQSAGVLDALYGSK---AK---DASLGP-------VF----R-N----------G----

-----------TPTLSVWAPTA-RTVSLDL-------------------DGKS-VAMRRD

DR---TGVWSV------TG-NK-S-----WN--GKPYRYVVDVWAP-----TVHKLVTNK

VTDPYSTALTTD-----SARSLVVDL---EDRKLAPKGWDTL--RKP-AA-VPLRDAQIQ

ELQIRDFSIADPT--SKHPGQYLAFTDTR---------SDGMKHLEQLADAGTSYVHLLP

AFDIGT--IPERKK---------------DRAEPAC------------------------

---ELSVYA---PDSAEQQACVAKAAAKDAYNWGYDPLHYTVPEGSYASDP---NGTKRT

VEFRQMVQGINGAGLRTVMDVVYNHTVASGQ-DDKSVLDRIVPGYYQRLL-DDGTVATST

C--CADTAPENTMMGKLVVDSVVTWAKEYKVDGFRFDLMGHHPKANILAVRKALDALTPA

KDGVDGKKIILYGEGWNFG-EIADDARFV----QATQKNMA-GTGIATFSDRARDAVRGG

SPFDEDP----GVQGFATGLYTDPNPSA--------ANGTKA-EQKARLLHYQDLIKVGL

TGNLADYTFTD-T-SGA-AVKGSGV-DY-NG-APAGYAAVPGDALAYADAHDNETLYDAL

AFKL--------P---------------SGTSAADRARMQVLAMATATLSQGPALSQAGT

DLLRSKSLDRNSFDSGDWFNALHWDC--------RA-GNGFG-RGLPPAADN----EAKW

PYAEPLLT-N-AK-VS--PGCAQINGASAAYRDLLTIRTTENDFDLTSTAQVQSRLSFPL

S--G-RN--ETPGVITMRL-------------------GKLVVVLNAAPGTTTQKVTALA

--------GKNYALHPVQAK--G-A--DST-VKRSTYERSS--GSFTVPGRTVAVF----

-------

>UniRef90_A0A6G3UK21_916_1771 | Alpha-dextrin endo-1,6-alpha-glucosidase n=25 Tax=Streptomyces TaxID=1883 RepID=A0A6G3UK21_9ACTN | E_val=6e-188

----------PTPDLTKAEAQWIDAGTVVWK-VKA----------TEA-TSQQLVYAEKG

G-ITVKDG---ALS----D-E----GQWLRL--T-PSA--LTDAQKAKYPHLK--DYPAF

TVDARD------RDRVR-----EALRGQLIATQRA-----A----------NGA-LLAAT

GVQSAGVLDDLYGAK---AS---GAALGP-------VF----R-H----------G----

-----------TPTLSVWAPTA-RTVSLEL-------------------DGRT-VPMRRD

DR---TGVWSV------TG-KK-S-----WN--GKPYRYVVNVWAP-----TVQKLVTNK

VTDPYSTALTTD-----SARSLVVDL---EDRKLAPKGWDTL--RKP-AA-VPLRDAQIQ

ELQIRDFSIADPT--SKHPGEYLAFTDTR---------SDGMKHLKQLADSGTSYVHLLP

AFDIGT--IPEKKK---------------DQQKPAC------------------------

---DLSVYA---PDSAEQQACVAKAAAKDGFNWGYDPLHYTVPEGSYASDP---DGTKRT

VEFRQMVQGLNGAGLRTVMDVVYNHTVASGQ-DDKSVLDRIVPGYYQRLL-EDGTVATST

C--CANTAPENTMMGKLVVDSVVTWAKEYKVDGFRFDLMGHHPKANILAVRKALDALTVA

KDGVDGKKIILYGEGWNFG-EVADDARFV----QATQKNMA-GTGIATFSDRARDAVRGG

SPFDEDP----GVQGFATGLYTDPNTST--------ANGTKA-EQKARLLHYQDLIKVGL

TGNLADYTFTD-T-TGN-TVKGSGV-DY-NG-APAGYAAAPGDALAYADAHDNESLYDAL

AFKL--------P---------------AGTSAADRARMQVLAMATATLSQGPALSQAGT

DLLRSKSLDRNSFDSGDWFNALHWDC--------RA-GNGFG-RGLPPAADN----EAKW

PYAEPLLT-N-AK-AT--PGCAQINGASAAYQDLLTLRTTEPDFDLATAAQVRSRLTFPL

S--G-KD--ETPGVITMRL-------------------GKLVVVFNAAPGTTTQKVAALA

--------GENYALHPVQAK--G-A--DST-VKESSYERSS--GSFTVPGRTVAVF----

-------

>UniRef90_UPI0003767C68_947_1798 | pullulanase-type alpha-1,6-glucosidase n=1 Tax=Streptomyces hokutonensis TaxID=1306990 RepID= | E_val=1.1e-187

--------------LTTSKAVWIDRNTVAWN-G-S----------DAA-ASTQLLYSHDG

S-IAVQDG---ALT---SD-D----ERWLRL--S-KTT--LTDAQKAKFPYLK--DYTAW

SVDPRD------RSRVR-----EALTGQLVASQRA-----V----------NGA-VLAAT

GVQVAGVLDDVYA-A---AA---KADLGP-------TF----R-K----------G----

-----------RPTLAVWAPTA-QNVSLDL-------------------DGSL-KRMSRD

AT---TGVWSV------TG-PA-S-----WT--NKPYRYVVKVWAP-----TVRKVVTNQ

VTDPYSVALTAD-----SERSLVVDL---DAKSLAPSGWSSL--KKP-KA-VALKDAEIQ

ELHIRDFSVADKTVPAKDQGTYLAFTDKN---------SDGSKHLRELAESGTSYVHLLP

AFDIAT--IPEKKS---------------EQSTTDC------------------------

---DLASYA---ADSEKQQECVAAVAAKDAYNWGYDPYHFTVPEGSYATDP---DGTGRT

VEFRKMVKSLNEDGLRVVMDVVYNHTAASGQ-ADYSVLDKIVPGYYQRLL-ADGSVATST

C--CANTATENAMMGKLVVDSVVTWAKEYKVDGFRFDLMGHQPKANILAVRKALDALTLA

KDGVDGKKIIMYGEGWNFG-EVADDARFV----QATQKNMA-GTGIATFSDRARDAVRGG

SPFDADP----GVQGFASGLYTDPNSST--------ANGTSA-EQKARLLHYQDLIKVGL

SGNLAKYRFTD-T-DGK-DVTGAEV-DY-NG-TAAGYADAPGDALAYVDAHDNESLFDAL

TYKL--------P---------------AGTSAADRARAQVLAMATATLSQGPSLSQAGT

DLLRSKSLDRNSFDSGDWFNAIHWNC--------AD-GNGFG-RGLPMAADN----ASKW

PYAKGLLT----S-VK--VGCGQIEGASAAYQDLLRIRTTEGVFSLDTAGQVQSKLSFPL

S--G-KD--ETPGVITMEL-------------------GDLVVVFNATPGAQEQKVGALA

--------GTGYRLHPVQAA--G-A--DAV-VKSASYDKAT--GTFVVPERTVAVFT---

-------

>UniRef90_UPI001A9CD060_942_1779 | pullulanase-type alpha-1,6-glucosidase n=1 Tax=unknown TaxID=N/A RepID=UPI001A9CD060 | E_val=2.9e-187

--------------------------------VNA----------KAA-ASTQLVYSRDG

G-ITVKDG---ALS----D-E----GRWLRL--L-PAQ--LTDTQKTKYPHLK--EYTAF

TVDPRD------RDRVR-----DALAGQTVLTQRL-----A----------NGA-LVAAT

GVQTQGVLDDLYGSK---AE---QAALGP-------VF----K-G----------N----

-----------KVTLSVWAPTA-QHVALEL-------------------GGKN-LAMRRD

DT---TGVWSV------TG-SA-SA----WR--GKEYRYAVKVWAP-----SVQKVVTNE

VTDPYSVALTAN-----SERSVVADL---DDKSLAPSGWSSL--KKP-EA-VAMKDAQIQ

ELHIRDFSVEDKTTPKDERGTYLAFTDKN---------SDGSKHLKELAKSGTSYVHLLP

AFDIAT--IPEKKS---------------EQATPDC------------------------

---DLTSYP---ADSDKQQECVAAAAAKDAYNWGYDPYHYTVPEGSYATDP---DGTART

VQFRKMVKSLNDDGLRVVMDVVYNHTTSSGQ-SKTSVLDKIVPGYYQRLL-ADGSVANST

C--CSNTAPENTMMGKLVVDSIVTWAKEYKVDGFRFDLMGHHPKANILAVRKALDALTPA

KDGVDGKKIILYGEGWNFG-EVADDARFV----QATQKNMA-GTGIATFNDRARDAVRGG

SPFDSDP----GVQGFASGLYTDPNSSD--------ANGSPA-EQKARLLHYQDLIKVGL

SGNLAGYTFTD-T-EGK-QVKGSDV-DY-NG-AAAGYADAPGDALAYADAHDNETLYDAL

TYKL--------P---------------ASTSTADRSRMQVLALATAALSQGPALSQAGS

DLLRSKSLDRNSFDSGDWFNAIHWDC--------RA-GNGFG-RGLPPAADN----KDKW

PFGKPLLT-S-VA-V---PDCPAITGTSAAYQDLLKIRTDESAFHLSTTGQVQKALSFPL

S--G-KD--ETPGVITMRL-------------------GDLVVVFNATPKTQEQTVKDTE

--------GAAYHLHPKQAN--G-S--DDV-VKSASFKASS--GTFTVPARTVAVFT---

-------

>UniRef90_A0A0W1KMU7_1056_1935 | Alpha-amylase n=5 Tax=Trueperella TaxID=1069494 RepID=A0A0W1KMU7_9ACTO | E_val=5.6e-187

------------------TAYWVDESTFAWP-GALT-------GGRTD-LTFEL-WGGTG

A-LSVANG---AVA---GD-G---ATKLAKMTHS-SSG--LSSEQLKNRGHLR--GYETL

TVAME-------RSAVE-----EALRGNLAILAKD-----A----------SGT-PVAFT

GLQIPGVLDALYASA---ARE--AGGLGV-------HF----N-D----------G----

-----------VPTLKLWAPTA-KSVALQLFDSEDAT-----------GDPAS-LPME-H

QA---DGTWTI------TG-ES-A-----WK--NRAYRYDVEVFVP-----STNQVEHNV

VTDPYSVGLTVD-----STHSVLVDL---SDPDYMPTLWQTS--EAP-VI-TNDSARSIY

ELHVRDFSIGDTTVPEKLRGTYEAFALED---------SAGVKQLRELAAAGMNMVHILP

TFDIAT--IPEKRA---------------EQKVASV------------------------

-----PDAG---AASSEQQAAVMAVADEDGFNWGYDPYHYMTPEGSYAAEE-NQSGANRT

MAYREMVGALHSMGYQVILDQVFNHTAQSGQ-GEKSVLDKVVPGYYHRLN-ADGTVANST

C--CENLATENAMAEQLMVDSVVTLARDYKIDAFRFDLMGHHSRANMEAIQAGLAQLTPE

KDGVDGKNIYMYGEGWNFGDDVQNNKRFE----QATQGQLD-GTGIGSFNDRLRDAVHGG

SGFDSNKS---QGQGFGNGQYTDPNA----------LNSASD-DQLASLRHNQDLIRLGM

AGNLKDFTFVT-S-SGE-EKRGDQL-DY-NG-QKAGYAISPEENVNYVDAHDNETLFDTN

MWKL--------P---------------ADSTMDTRVRMNTVSLATVTFGQSPSFWHAGT

DLLRSKSMDRNSYNSGDYFNAIDWTG---------Q-KHNFG-VGLPPARDN----DVQW

NAMKPFLE-N-TA-NV--ASPQDLAKAHAQALELLRLRHAHPLLTLGSAELVKEKVTFPG

A--G-KD--QQAGLILMRVDDTVG------A-DVDPDSDGLLVAINASPVELSQGIDEMK

--------GLTLSLSPVLTD--G-QDDDPV-LQGTTWDSET--GTVTVPARSAVVLVQEK

-------

>UniRef90_A0A561EMB8_1128_1866 | Alpha-dextrin endo-1,6-alpha-glucosidase n=1 Tax=Kitasatospora atroaurantiaca TaxID=285545 RepID | E_val=1.2e-186

------------------------------------------------------------

------------------------------------------------------------

------------------------------------------------------------

------------------------------------------------------------

-----------KVTLSLWAPTA-TEVSVQLFDRSTG------------GTPRT-VPLKRD

EA---SGVWSL------TR-DADE-----LD--GKYYLYQVKVWAP-----SVQQLVTNV

VTDPYSVALSTD-----SKRSLVADL---SDRSTKPRGWDRH--RSP-QP-VSANRQQIQ

ELHVRDFSSADTTVPAAERGTYLAFTESR---------SAGVQHLRDLAKAGVTTIHLLP

TFDIAT--IRENRA---------------EQKLPAC------------------------

---DLTSLA---ADSEKQQECVAAVQADDAYNWGYDPLHYTVPEGSYATDS---DGTART

VQFRQMVQAMHEAGLRVVLDVVYNHTAAAGQ-AEHSVLDKVVPGYYQRLS-DAGKVTTDS

C--CADTAPEHAMMNKLVVDSVVTWARQYRVDGFRFDLMGLDPKSTMLDVQSALRGLTKE

KDGIEGKDVFLYGEGWNFG-VVANNSRFV----QASQLNMA-GTGIATFNDRVRDAARGG

NFMLSSA----PQQGFASGLYTDPNGSA--------DNGTPA-EQKARLLHQMDQIKVGL

TGNLAGYSFTD-S-SGK-AVSGAGV-DY-NG-SPTGYAASPGEAVEYVDAHDNADLFDAL

AYKL--------P---------------AGTTAADRARMQALGLSLTALGQGPGFAQAGS

DLLRSKSLDANSFDSGDWFNSIHWDC--------TQ-GNGWG-RGLPMAADN----QAMW

PRAKSLLA-D-PK-LT--VGCNEIDATTAQYQQFLRIRQSSPLFSLSSAAAVQQRLSFPL

S--GTAG--ETPGVITLHLD---G------T-GLPGAEKGITVVFNATPTAQQQTVTALQ

--------GTRQALHQVQAE--G-A--DQV-VRQSGFDAAT--GTFTVPGRTVAVFVQ--

-------

>UniRef90_UPI0011EFF529_43_907 | pullulanase-type alpha-1,6-glucosidase n=1 Tax=Luteimonas sp. LNNU 24178 TaxID=2605430 RepID= | E_val=3.2e-186

----------------DARAHWLDRRLIRWP-GVVT---------GDG-QRMQLYHSAGG

T-IVARHG--ARVA---GA-D-----GALALEAY-DGE--IPAALAERFRFLE--SGTTL

AVRDED------AARLP-----SLHTGQLLLVHED-----A----------DGR-VREAT

ALQVAGALDDLYAAA---EG---IDDFGA-------TV----G-F----------D----

-----------GTRFRLWAPTA-RAVSVCLHRDDRA------------PADAV-HPLRRD

AA---TGVWSR------SL-PR-D-----LS--GRYYTYVIDVFVR-----DMG-VVRNR

VTDPYSTGLGAD-----SRRSVAVDL---DAPALKPDGWDAA--AAP-AI-AAQTDMTIY

ELHVRDFSRDDASVSEANRGKYLAFTESG---------SDGMRHLRTLAGAGLTDVHLLP

VFDLAS--VPETGC-----------------VSP--------------------------

---AIPDAA---PDSPAQQAAVMAVAARDCFNWGYDPFHFNAPEGSFASDA--ADGAARI

LEFRRMVMALHAAGLRVGMDVVYNHTSASGQ-HPQSVLDRIVPGYYHRYD-AAGRVERST

C--CDNTATEHRMMAKLMIDSAVLWARHYRIDSFRFDLMGHQPRAAMERLQAAVDAAA--

-----GRRIHLIGEGWNFG-EVADGARFV----QASQQSLA-GSGIGTFSDRTRDAVRGG

GPADRGRA-LLTEQGWINGLVYDRNPHA------------PE-RPLEELKRSADLVRVGL

AGTLRDYTMSA-H-DGT-RKTLAEI-DY-KG-QPAGYAVQPDEVVNYVENHDNQTLFDIN

VFKL--------P---------------PDTSREDRARVQMLGAAVTAFSQGIAYFHAGL

ETLRSKSLDRNSYDSGDWFNRLDWTL---------R-DNHFG-SGLPPAQDN----REDW

PLMQPLLS-H-PD-IE--PGPEQIRLARDMFLDLLRIRASTPLFRLRSADEITRRLTFPN

G--G-PA--QNPRVIAGRLDGA--------G-LADAGFAEVLYFINVDPAAQTLELSGLR

--------GRDWTLHPVHRAPDA-A--DPRPAAQAHYDRAS--GRFTLPGRTALAYVI--

-------

>UniRef90_UPI0005F2A217_266_1057 | pullulanase-type alpha-1,6-glucosidase n=1 Tax=Actinoplanes rectilineatus TaxID=113571 RepID= | E_val=6.3e-186

------------------------------------------------------------

------------------------------LVPR-PGG--LFQAQSRKFPHLR--SYRAF

AIPEMS------DLALA-----ELLRDQLVVTGRD-----R----------DGR-EVART

GVQLAGVLDELYAD----AL---DVPLGP-------QL----D-G----------D----

-----------DPHLAVWAPTA-REVALELFRTVD-------------DEPKI-LPMERD

DT---TGVWSL------PV-KR-K-----WF--GRYYRYRVEVWHP-----AAQRIVTTS

VTDPYSISLAAG-----STHSQLVDL---SDPELCPPGWDTG--GEL-PP-VAPARMQIS

ELSVRDFSIADPTVPAAERGTYLAFTHAG---------SHGMRHLRSLAEAGLTHVHLLP

VNDFAS--VPDRRS---------------EQAVPAC------------------------

---DLAALP---PDSEAQQAAVMAVADRDGYNWGYDPWHWTTPDGSYAHDP---DGSARV

VEMRSAVAALHTAGLRVVLDVVYNHTMHDGL-DRFSVLDRVVPGYYHRLL-ADGSTAEST

C--CPNTATEHAMMGRLVVDSLVTWRRAYRVDGFRFDLMGHHPKANILAAREAL------

-----GAGVALYGEGWNFG-EVAYDARFV----QATQVNMA-GTGIGTFNDRLRDAVRGG

GSS-GDP----SVRGFASGLGSETP------------------------LWLHDQIKVGL

SGALAAYRFVT-H-TGA-EHSGSQI-DY-NG-SPSGYAHDPGEAVNYVDAHDNEILYDAM

AFKL--------P---------------VALDPLERARMQVLALSLVVLSQGAGFVALGS

ERLRSKSLDRNSYNSGDWFNQIRWDP--------AD-GNGFG-VGLPPWPDN----GDKW

DQARPLLG-E-AS-LV--PPVEAVELTAERYRELLRIRRSSPVFGLPTAEEVQRRLTFPL

G--G-PG--ETPGVIVMCLD---G------T-GLDPRWRAVVVVFNVTDQATRQVVPDVV

----------SLRPHPAQVS--S-A--DPL-LRGAA-AFPD--GAVEVPARSVAVFVSP-

-------

>UniRef90_A0A2X0J8T1_986_1831 | Alpha-dextrin endo-1,6-alpha-glucosidase n=2 Tax=Streptacidiphilus pinicola TaxID=2219663 RepID= | E_val=1.5e-185

----------------------------------------------NG-SSAELVYSPTG

A-IAVKNG---DTT---DP------GYWLRLLPV-SGG--LTPAQLAKHPELK--GYAAF

TVDPRD------VKRIP-----LALRSQLVMTERE-----A----------NGA-LLAAT

GVQLPGALDAIYGAA---AA---KAPLGP-------VY----GHD----------G----

-----------TPSLSVWAPTA-RQVSLELYDSATA------------TTGRV-VPMRRQ

DA---SGVWTV------RG-DK-S-----WT--GRFYLYRVTVWAP-----SVQQVVTNH

VTDPYSVSLSTD-----AKRSQLVDL---SDPSLAPKGWRQD--RSP-KA-ISAGQQEIQ

ELHVRDFSVADATVPAAERGTYLAFTDLN---------SAAMKHLAQLARQGLTTVHILP

AFDFAS--VPDAKS---------------EQTTPAC------------------------

---DLASFA---ADSDQQQACVAKQAASDAYNWGYDPRHYDVPEGAYSTDP---NGTARI

EQFRAMVAALHRIGLRVVMDVVYNHTAASGE-NADSVLDQIVPGYYQRLS-ADGAVTTDS

C--CADTAPEHALMNRIVVDSTRLWATQYHVDGFRFDLMGLDPKATMLDVKAGLAA----

----TGRHEFLYGEGWNFG-VVANDARFV----QATQANMA-GTGIATFNDRLRDAARGG

GPFDTNP----HLQGFASGLFTDPNGDA--------VNGSAA-DQKARLLNQMDQIKVGL

TGDLAAYSFTD-S-AGK-TVTGSEI-DY-NG-SPTGYTAAPGEAITYVDAHDNLDLYDAL

AYKL--------P---------------AGTSMADRVRMQALALATTELSQGPGFSVAGS

DLLRSKSLDANSYDSGDWFNAIQWQC--------AN-GNGFG-RGLPAAGSN----SAFW

PFAKPLLA-Q-PS-LV--ADCSAQQQASALYQQFLKIKASTPLFSLGTAAAVQQRLSFPL

S--GTAG--ETPGVITLHLD---G------S-GLS-TYRSVTVVFNATPSAQQQTLTGLV

--------GTAQRLAPAQAD--G-A--DPV-VKTSTFDSAT--GTFTIPARTVAVYVQ--

-------

>UniRef90_UPI0012FC7831_989_1768 | pullulanase-type alpha-1,6-glucosidase n=1 Tax=Nonomuraea typhae TaxID=2603600 RepID=UPI0012F | E_val=3e-185

------------------------------------------------------------

------------------------------------GT--LTDAQKAAWPHLK--ALPAY

KVDPRD------TGLVP-----EALRGQVAAVERS-----A----------AGR-LVSAT

GVQLPGVLDDLY------AT---DEPLGY-------------A-G----------G----

-------------RLALWAPTA-QQVRLALYDG---------------GDRTE-HDMRRE

AK---TGVWSV------RA-PA-S-----WL--GRDYTYLVTVYSP-----AEGRVVTNE

VTDPYSVAVAPD-----GVRSRLADL---ARQ--RPTGWETL--AKP-PA-KPA--KTVY

ELHVRDFSAADRSVPAAVRGTYGAFTG-D---------GAGMRALRDLARDGLTHVHLLP

VFDVAT--IPERAA---------------DRAEPGC------------------------

---DLAAMP---PDSPLQQECVARTAARDAFNWGYDPLHYSVPEGSYAADP---D--QRT

REFRAMVAGLNRAGLRVVMDVVYNHTHSA------RTLDAIVPGYYHRLL-ADGSVATST

C--CPNTAPEHAMMGRLVVDSVVTWAREYKIDGFRFDLMGHHPKSNILAVRKALDRLTPA

GDGVNGKEILLYGEGWDFG-EVAGGARFP----QATQANLA-GTGIGTFNDRLRDGVRGG

GPFDADP----RLQGFGSGLAGDVNDAP--------GNR----DAAARLRHYQDLVMLGL

TGNLRDYVLPG-G------RTGAEV-SY-NG-SPAGYAAQTSDVVTYVDAHDNETLFDAL

AYKL--------P---------------QERDMSARVRMQGLALATVLLAQGTAFVHAGS

ERLRSKSLDRNSYDSGDWFNRLHWDC--------AQ-GNGFG-AGLPPEADN----AGRW

PYARPLLA-D-PA-LR--PDCAAITTARARFGEFLRIRGSSPLFALPAA-EARARISFPV

T---------RPGVIVMRLD---G------T-GLDPRWSSITVVFNATPSAAGATVKELQ

--------GTATTLHPEQ------A--DPV-AAGSAFDPAT--GTLTVPGRTAAVFV---

-------

>UniRef90_A0A4R7MDD7_1014_1848 | Alpha-dextrin endo-1,6-alpha-glucosidase n=4 Tax=Streptomycetaceae TaxID=2062 RepID=A0A4R7MDD7_9 | E_val=8.6e-185

---------------------------------------------------------PTG

A-LAVKDG---DLT---DP------GYWLRLLPV-STG--LTAAQLAAHPELK--GYAAF

TVDPRD------TKRIT-----TALRGQLIMTERE-----A----------NGA-LLAAT

GVQIPGVLDAVYATK---AV---STTLGP-------VV----S-N----------A----

-----------SARLSVWAPTA-QSVSLELYDSPTA------------TTTRT-VAMHRD

DS---TGVWSV------TG-PA-S-----WK--GKYYLYNVTVWAP-----SVQKVVTNH

VTDPYSLALSAD-----SKRSEIVDL---NDPSTAPSGWTGD--RSP-KA-LTATQQEIQ

ELHVSDFSSADTTVPAADRGTYLAFKDLN---------SDGMKHLAALARSGVTTVHLLP

TFDFSS--VPELKS---------------EQTTPAC------------------------

---DLASYA---PDSQAQQACVSAQAATDDYNWGYDPYHFTVPEGSYATNA---DGTVRT

EQFRQMVAALHRIGLRVVMDVVYNHTAASGE-AATSVLDQIVPGYYQRLS-ATGAVTTDS

C--CADTAPEHTMMNKLVVDSTRTWADAYHVDGFRFDLMGLDPKQTVLDVQASLKK----

----TGRSEFLYGEGWNFG-VVANNARFV----QATQANMA-GTGIATFNDRQRDAVRGG

GPFDTDP----RIQGFASGLYTDPNGAA--------VNGTSD-QQKANLLHAMDQLKVGL

TGNLAAYSFTD-S-SGK-TVKGSEV-DY-SG-SPTGYTAAPGEAITYVDAHDNLDLYDAL

IYKL--------P---------------IGTSMADRARMQSLALATTGLSQGPGFAVAGS

DLLRSKSLDANSYDSGDWFNAIHWNC--------TQ-GNGFG-HGLPLAASN----QSSW

TFAQPLLA-R-TD-LV--PGCAAELSASAQYQQFLQIKKSTPLFSLTTAADVQQRLSYPL

S--GTSG--ETPGVITLHLD---G------T-GLN-TYKSVTVVFNATPTSQQQTLTTLA

--------GTTQQLHPAQAD--G-S--DPV-VRTSAFDPAT--GTFTIPARTVAVFVQ--

-------

>UniRef90_A0A3N6FGT5_913_1769 | Alpha-dextrin endo-1,6-alpha-glucosidase n=3 Tax=unclassified Streptomyces TaxID=2593676 RepID=A | E_val=2.8e-184

----------PTPDLAKAEAQWIDADTVVWK-VKA----------TGA-TSQQLVHSKDG

S-ISVVDG---ALS----D-E----GQWLRL--T-PSA--LTDVQKAKYPHLK--DYPAF

TVDARD------RDRVR-----EALRGQLIATQRA-----A----------NGA-LLAAT

GVQSAGVLDDLYGKH---AA---GASLGP-------VFD---R-H----------G----

-----------RPTLSVWAPTA-RTVALEL-------------------DGRG-VPMRRD

DR---TGVWSV------TG-PR-S-----WK--GKPYRYTVDVWAP-----SVGKMVTNK

VTDPYSTALTTD-----SARSLVVDL---DDPALAPKGWSGL--RKP-AA-VPLRDAQIQ

ELHIRDFSIADRT--SKHPGEYLAFTDTG---------SDGMKHLKRLADSGTSYVHLLP

AFDIGT--IPEKKS---------------AQQKPAC------------------------

---DLSVYA---PDSEEQQACVTKAAAKDAFNWGYDPLHYTVPEGSYASDP---DGTRRT

VEFRRMVQGLNGAGLRTVMDVVYNHTVASGQ-DDKSVLDRIVPGYYQRLL-DDGTVATST

C--CANTAPENTMMGKLVVDSVVTWAKEYKVDGFRFDLMGHHPRANILAVRKALDALTVA

KDGVDGKKIILYGEGWNFG-EIADDARFV----QATQKNMA-GTGIATFSDRSRDAVRGG

SPFDEDP----GVQGFATGLYTDPNASA--------ANGTKA-EQKARLLHYQDLIKVGL

TGNLADYTFTD-S-SGR-TVKGSGV-DY-NG-APAGYAAVPGDALAYADAHDNESLYDAL

AFKL--------P---------------AGTPAADRARMQVLAMATATLSQGPSLSQAGT

DLLRSKSLDRNSYDSGDWFNALHWDC--------RD-GNGFG-RGLPPAADN----EPKW

SYAKPLLA-D-AA-IG--PGCAQIDGASAAYRDLLTIRSTERDFSLATAGQVQSALSFPL

S--G-KD--ETAGVITMRL-------------------GKLVVVLNAAPGTETQTVAELA

--------GKKYALHPVQAA--G-A--DPT-VKKSTYDRSS--GRFTVPGRTVAVF----

-------

>UniRef90_A0A540P4D2_954_1818 | Alpha-dextrin endo-1,6-alpha-glucosidase n=3 Tax=Streptomyces ipomoeae TaxID=103232 RepID=A0A540 | E_val=7.8e-184

--------------LTTSKAVWIDRNTLAWN-G-D----------ESA-ASTQLLYSRTG

S-IALRNG---TLT---ST-D----ERWLRL--T-RSA--LTDAQKARFPHLK--SYTAW

TVDPRD------RDRVR-----EALRGQLVASQRA-----AGGTSQAFSSGGGV-VLAAT

GVQIAGVLDDVHGTA---AS---DADLGP-------TF----H-D----------G----

-----------RPTLAVWAPTA-QKVSLDL-------------------DGTT-VAMRRN

DT---TGVWFV------TG-SR-S-----WK--NKPYRYVVKVWAP-----SVGKVVTNK

VTDPYSVALTAD-----SERSLVVDL---GDRSLAPSGWTSY--TKP-KA-VPLKDARIQ

ELHVRDFSIGDRTVPAKDQGTYLAFTDKD---------SDGSRHLRELAQAGTSYVHLLP

VADIAT--VPEKKA---------------DQATVDC------------------------

---DLGSYA---ADSDKQQECVAKAAAKDAYNWGYDPYHYTVPEGSYATDP---DGTRRT

VEFRKMVKALNDDGLGVVLDVVYNHTPASGQ-ADKSVLDKVVPGYYHRLL-ADGSVATST

C--CANTAPENTMMGKLVVDSIVTWAKEYKVDGFRFDLMGHHPKANILAVRKALDGLTPA

KDGVDGKKIILYGEGWNFG-EVADDARFT----QATQKNMA-GTGIATFSDRARDAVRGG

GPFDADP----GVQGFASGLYTEPNSSD--------GNGTSA-EQKARLLHYQDLIKVGL

SGNLADYRFTD-T-SGK-EVKGADV-DY-NG-TAAGYADAPGDALAYVDAHDNETLFDAL

TFKL--------P---------------KSTSPADRARMQVLAMATAALSQGPALSQAGT

DLLRSKSLDRNSYDSGDWFNAIHWDC--------RDGGNGFG-RGLPPAADN----QSKW

PYAKPLLT----S-VE--VGCAQIEGASAAYRDLLRIRTTESAFSLDTTAQVQSTLSFPL

S--G-KD--ETPGVITMEL-------------------GDLVVVFNATPEKRDQTVADLA

--------GTEYTLHPVQAE--G-A--DST-VKNASYEAES--GTFAVPGRTVAVFAR--

-------

>UniRef90_A0A5J4LJC7_88_875 | Uncharacterized protein n=3 Tax=Streptomyces angustmyceticus TaxID=285578 RepID=A0A5J4LJC7_9ACTN | E_val=2.2e-183

------------------------------------------------------------

-----------------------------------------------AHPHLT--GYPAF

TVDPRD------RGLAP-----TALRGRLLAAQRT-----A----------DGG--RATT

GVQIPGVLDDLYGAR---AR---HATLGP-------DF----H-H----------G----

-----------RPTLTVWAPTA-RTVALEL-------------------DGRR-VPMRRD

DT---TGVWRV------TG-AR-D-----WA--GKPYRYLVTVWAP-----TVGKTVTNK

VTDPYSTALTAD-----SARSLVTDL---DAPELAPPGWSRL--AKP-AA-VPLRRARIQ

ELQIRDFSAEDRT--SRHPGRYLAFTDRR---------SDGMRHLASLARSGTSYVHLLP

AFDFAT--TPERPA---------------DQARPGC------------------------

---DLASYA---PDSDRQQDCVAKTAAHDAYNWGYDPLHYTVPEGSYASDP---DGTART

VEFRRMVQGLNGAGLRAVMDVVYNHTAASGQ-DPHSVLDRIVPGYYQRLL-DDGGVATST

C--CSGTAPEHTMMGKLVVDSVVTWAKEYKVDGFRFDLMGHHPKANILAVRKALDALTPA

KDGVDGKKIILYGEGWNFG-EAANDARFV----QATQRNMA-GTGIATFNDRARDAVRGG

SPFDADP----RVQGFASGLYTDPNSAQ--------ENGSPA-EQRTRLLHYQDLLKVGL

TGNLADYTFTD-S-TGH-RVKGSGI-DY-NG-TPAGYAAAPGDALAYADAHDNETLYDAL

AYKL--------P---------------PRTGAADRARMQVVALATAALSQGPALSQAGS

DLLRSKSLDRNSFDSGDWFNALHWNC--------AD-GNGFG-RGLPPAADN----KDKW

PYARPLLA-D-PA-LR--PGCATIRHTAAAYRDLLRIHATEPAFGLPTAAAVQKALSFPL

S--G-KD--ETPGVLTMRL-------------------ANLVIVFNVTPQRAHQTVPSLA

--------GTPYTLHPTQAH--G-T--DPT-TATATYTRPS--GTFTVPARTVAVF----

-------

>UniRef90_I0HKS6_313_1173 | Pullulanase n=4 Tax=Rubrivivax gelatinosus TaxID=28068 RepID=I0HKS6_RUBGI | E_val=6.9e-183

----------------DARAYWLTRALIQAP-RW-----------DST-GVFKLYYASRG

Q-IQAPRG--AKAS---GA-D-----GALTLDVS-TAE--LDAAVAERFKFVA--PGVRL

AVRSAD------QARLG-----DLLKRQLVLVQET-----A----------DGL-VRNAT

TAQLPGALDDLYAAA---AT---VGDLGV-------TP----G-A----------E----

-----------RTVFKLWAPTA-QKVSVAIYDSATG------------PTVAL-EDASFD

AA---TGVWRA------ER-SG-D-----LS--GRYYRWVVEVFVR-----GVG-LVRQL

VTDPYSVSLSAD-----SKRSYVGSL---SAAALKPAGWDGH--TRPAAL-AASPDMSIY

ELHVRDFSANDATVPAAHRGKYLAFTDTA---------SNGMRHLAALAGAGLTDVHLLP

VFDIAT--VPETGC-----------------VTP--------------------------

---TI-SGA---PDGSTQQAAIGAVKSEDCFNWGYDPFHFNAPEGSYATDA--QDGAVRV

REFRSMVMALHAAGLRVGMDVVYNHTTAAGQ-DDKAVLDRVVPGYYQRLN-AVGDLENST

C--CANTATENLMMGKLMVDSVVLWATQYGIDSFRFDLMGHQPRAVMERLQAAVDAAT--

-----GRHVDLIGEGWNFG-EVADGARFV----QASQLSLN-GSGIATFSDRARDAVRGG

SPFDGGDA-LIANQGYVNGLFYDPNALG------------GG-KTATDLLRAADLVRVGL

AGSIRDYTLRT-Y-TGD-QRQLQAI-DY-NG-QPAGYVSQPGEVVNYVENHDNQTLFDID

VYKL--------P---------------LATSAEDRARVQMLAAAVNVFSQGVAYFHAGI

DTLRSKSLDRNSYDSGDWFNKLDWTY---------T-SNNFG-VGLPPEGDN----GSNW

DLMRPLLA-D-TE-LL--PPPAQIAWTRDAFRDLLRIRASSTLFRLRSADDVKARLAFRN

T--G-AS--QVPTVLVGHLDGS--------G-YAGAGFGEILYFVNVDDEARTLTIPEDA

--------GKAWVLHPVHRAAGA-A--DTR-AATATVDSAA--GRFTLPARTAVVFV---

-------

>UniRef90_A0A3S9PFZ7_106_845 | Pullulanase-type alpha-1,6-glucosidase n=2 Tax=Streptomyces TaxID=1883 RepID=A0A3S9PFZ7_STRLT | E_val=2.7e-182

------------------------------------------------------------

-----------------------------------R------------------------

-----------------------------------------------------------T

GVQIPGVLDDLYAPA---AT---KAHLGP-------VF----R-D----------G----

-----------RPTLSVWAPTA-KTVALEL-------------------DGRT-LPMRRD

AA---SGVWSA------TG-PR-S-----WA--GKPYRYAVTVHAP-----TIGKTVVNH

VTDPYSTALTAD-----STRSLVTDL---SAPAFAPPGWANL--RKP-KP-VPFRDARIQ

ELHVRDFS---------PRGDYLAFTEPA---------SPGMRHLKSLADAGTSYVHLLP

VFDFAT--IPERRA---------------DQATPPC------------------------

---DLAAFP---AESEEQQKCVARTAARDAYNWGYDPLHYTVPEGSYASDP---DGPRRT

VEFRRMVQGLAGAGLRTVMDVVYNHTAASGQ-DERSVLDRIVPGYYHRLL-DDGSVATST

C--CANTAPEHAMMGKLVVDSVVTWAKEYKVDGFRFDLMGHHPKANILAVRAALDTLTPA

RDGVDGKSVILYGEGWNFG-EVANDARFV----QATQKNMA-GTGIATFSDRSRDAIRGG

GPFDDDP----RVQGFASGLFTDPNASP--------ANGSPA-EQRARLLHAQDLIKVGL

TGNLAAYRFTD-T-TGR-SVRGADV-DY-NG-QPAGYAAAPGDALSYADAHDNETLYDAL

AYKL--------P---------------QATTPDDRARTQVLALAAATLSQGPALSQAGT

DLLRSKSLDRNSYDSGDWFNAIHWNC--------AE-GNGFG-RGLPPAADN----AAKW

PYARPLLA-D-PA-LR--PGCGPIAAASAAYRDLLRVRTTERAFSQPTAEAVQAALSFPL

S--G-RD--ETPGVITMRL-------------------GDLVVVLNATPQERAQTVPALA

--------GTGYRLHPVQAH--G-S--DPV-VKSASYATGT--GTFTVPGRTVAVF----

-------

>UniRef90_A0A5D4XSG5_324_1185 | Pullulanase-type alpha-1,6-glucosidase n=2 Tax=unclassified Luteimonas TaxID=2629088 RepID=A0A5D | E_val=7.7e-182

-----------------ARAYWLSERLLRWP-RT-----------DTS-GRFRLYHSATG

Q-VVARKG--VAIA---GA-D-----GALDLQVA-QHE--VPADVAERFRWVG--AGAVL

EVPADQ------LDSLP-----GLMRRQLVLVQED-----E----------EGR-VLGAT

TTQLPGYLDARYAAA---DG---LDDLGV-------TL----D-R----------G----

-----------QARFRLWAPTA-QRVWLC-GERARG------------GGPLL-EPMQFD

PV---TGSWSL------AT-RA-L-----RA--GDHYRYAVEVFVR-----GVG-LVRNL

VTDPYSISLDAD-----SQRSYVGDL---SSPTLMPPGWARD--RSPDAV-RAQEDMSIY

ELHVRDFSINDASVRAPLRGKYLAFAQYN---------GDGLRHLRALAQAGLTDVHLLP

AYDFAS--VPETGC-----------------TTP--------------------------

---DIPDAA---PDSDAQQAAVAATRASDCFNWGYDPWHFNAPEGSYASDA--RDGAARV

REFRAMVMALHAAGLRVGMDVVYNHTSASGQ-DARSVLDRIVPGYYHRLD-AQGGIERST

C--CENTATEHLMMGKLMIDSVKLWATQYHVDSFRFDLMGHQPREVMVRLQDEVNAAA--

-----GRPVQLIGEGWNFG-EVANGARFV----QASQLSLN-GTGIGTFSDRARDHARGG

SGFDNGID-LLRNQGFLNGLHYDDNGSG------------GN-HPRSALMWSGDIIKVGL

AGSIRSYRLTT-H-WDA-TLPLEQI-DY-NG-QPAGYVVDPQEVVNYVDNHDNPTLFDAN

VYKL--------P---------------RTTSREDRARVQVLGAAITAFSQGVAYFHAGI

DTLRSKSLDRNSYDSGDWFNALDWTY---------Q-SNNFG-VGLPMQSDN----GDNW

PVMAPLLA-D-PS-IV--PMPADIRWTRDAFRDLLRIRASSTLLRMRTADDIEQRLRFHN

T--G-SA--QEPTVLVAHLDGR--------G-YPGANFRELVYLVNVDKRAHALPIAAEA

--------EKGYRLHPVHRARDA-A--DRR-AAAARYDRGS--GTFHVPARTAVVFVVP-

-------

>UniRef90_A0A6L8KED9_46_903 | DUF3372 domain-containing protein n=1 Tax=Duganella sp. FT135W TaxID=2692175 RepID=A0A6L8KED9_9B | E_val=4.1e-181

----------------EAHAYWLNRQLIKWP-GAN----------AAG-GVFKLYYSATA

Q-LQAAQG--SRVS---GA-D-----GAIALSRF-DGS--VPADVAQRFKFVG--NGEVL

AVAKAD------AARVP-----AALQQQVLLVLEA-----E----------DGT-IRDAT

ALQLAGALDDIYASA---AK---SDDLGI-------TV----G-V----------R----

-----------DVAFKLWSPTA-QNVALCTYASGTS------------KAAAI-TPMTRD

DS---TGIWSA------------R-----AG--GAYYQYLVDVVTP-----SAG-LVRNL

VTDPYSISLTTD-----SKRSYIADL---SAAKLKPAGWDKT--PAPKKV-AAQTDMTVY

ELHVRDFSINDSTVSTAHRGKYAAFAESK---------SNGMKHLAALSKAGMTDIHLLP

VYDIGS--VPEQGC-----------------VTP--------------------------

---YIPAAA---PDSEEQQAAVAKVKAVDCYNWGYDPFHYNAPEGSYSSDP--ADGAKRI

VEFRQMVQALHKTGLRVGMDVVYNHTFIAGQ-NEKSVLDRVVPGYYHRLN-AKGGIERST

C--CDNTATENLMMGKLLVDSVALWTKQYKIDSFRFDLMGHQPRAVMEQLQARI------

-----GKQINLIGEGWNFG-EVADGARFV----QASQLSLN-GTGIGTFSDRGRDAVRGG

GAGDSGTQ-LISQQGYINGLVYDANEQA------------GQ-RPVADLLHASDMVKVGL

AGTIRNYPLTI-A-DGR-TVALQDI-VY-GGNQPAGYASEPGETVNYVENHDNQTLYDIN

AFKL--------P---------------QATTARERAQVQMLGAAINAFSQGVAYFHAGF

DILRSKSLDRNSFESGDWFNRLDWSY---------Q-DNYYG-TGLPPAEDN----GKDY

ALIKPLLR-N-AS-IK--PAPADIAYARDAFRDLLSIRSSSTLFRLRSAEDIKQRLRFFN

T--G-PS--QVPTVIAAHIDGN--------G-YQGARFKSITYLINVDKVAQRITIGEEK

--------GRNYQLHPVHTSMAA-A--DKRVASEAKYDKNT--GTYSIPARSAVVFVE--

-------

>UniRef90_A0A4Q6FKV4_46_913 | DUF3372 domain-containing protein n=1 Tax=Xanthomonadaceae bacterium TaxID=1926873 RepID=A0A4Q6F | E_val=1e-180

-----------------SRAYWLDAATIRWP-KM-----------PAD-ARYRLYASTDA

R-LRVERG--HRVA---EA-D-----VVVALEP--AAA--LPEAVAERFRYTG--AGVEL

RLPPKG------RLRLH-----ELLRGQLLLVQED-----A----------QGR-VRDAT

YLQSAAALDALHASA---DG----MSLGA-------NP----G-A----------G----

-----------KTDFAVWAPTA-RTAAVCLYAGGNI------------PAHAA-LPLQRD

AA---SGTWSL------SL-PR-D-----LR--GAYYGWLLEVFVP-----GVG-IVRNR

VTDPYSVSLTTD-----SLRSYIADL---DDPALKPQGWDEA--QRPPAP-RARVDMAIY

ELHVRDFSVGDATVPPAHRGKYLAFTDVG---------SAGMRHLRALGQAGISDIHLLP

VFDIAT--IPESGC-----------------ATP--------------------------

---VIPHAA---ADSEAQQAAAMGAAAKDCFNWGYDPYHFNAPEGSYASDA--ADGAVRI

REFRAMVQALHAAGLRVGMDVVYNHTTASGQ-SPRSVLDRIVPGYYQRLD-ANGAVEHST

C--CDNTATEHRMMAKLMRDSVALWARQYRIDSFRFDLMGHQPRDAMLDVQRAANAAA--

-----GRNIPLLGEGWNFG-EIAGNARFV----QAAQGELD-GTGIATFSDRARDALRGG

GCCDSGAD-LIANQGLLNGLHEAPNELA------------QGRATRDDLLHAADLARAGL

AGTLRGYRMQF-A-DGR-IAPLQEL-AY-KG-QPAGYASQPGEVVNYVENHDNPTLWDIN

ALKL--------P---------------AATSAAERARVQLLGAAFVAFSQGVAYFHAGM

DLLRSKSLDRNSFDSGDWFNRLDWTY---------A-DNGFG-AGLPPGQDN----GKDW

PLLKPVLA-N-PA-NR--PSPGDIAWMRDAFRDLLRIRSSTPLFRLAEAGEVQRRLRFYN

T--G-PT--QDPRVIVGHLDGA--------G-MASA-YREVLYLLNVSPGTRVLTLPQES

--------GKAYVLHPAQAAPDA-A--DSR-AKLARYAASH--GRFTVPGRTAVVFVIRN

DMQALL-

>UniRef90_A0A0X3V8R8_262_1059 | Alpha-amylase n=2 Tax=Actinoplanes TaxID=1865 RepID=A0A0X3V8R8_9ACTN | E_val=2.4e-180

------------------------------------------------------------

-------------------------------TPR-PGG--LFQAQSRRFPHLR--AYRAF

AVRELG------DRVLG-----GLLRDRLLIEGRD-----T----------AGD-LVART

AVQIAGVLDDLYLE----AA---DADLGL-------TL----D-V----------D----

-----------RPQLAVWAPTA-RTVELELSREPG-------------SEPRI-LPMDRD

DL---TGVWSI------PV-KR-K-----WL--GRFYRYRVETWHP-----AAQRVVTTS

VTDPYSVSLAAD-----STHSQLVDL---DAAELKPPGWDDA--AKP-AA-VAPARMQIA

EVSVRDFSIGDGSLPAAERGTYLAFTHAG---------SDGMRHLRSLAEAGLTHVHLLP

VNDFGT--VPDHRA---------------DQAQPAC------------------------

---DLVTLP---PDSPEQQRAVMAVADEDGYNWGYDPWHWTTPEGSYATDP---AGTARI

LQMRSAVAALNATGLRVVLDVVYNHTMRDGL-DRFSVLDRIVPGYYHRLL-ADGSTAEST

C--CPNTAPEHMMMGRLVIDSLVTWARAYRIDGFRFDLMGHHPRANILEARIALDHRVE-

----HGRDICLYGEGWNFG-EVAYDARFA----QATQVNMA-GTGIGSFNDRMRDAARGG

GAF-GDP----GVPGFATGLGQRTPG------------------------FVHDRIKVGL

AGGLATYRFVT-H-DGV-ERSGAQV-DY-NG-SPCGYASAPGETVNYVDAHDNEILYDAM

AFKL--------P---------------PGTRPADRARMQVLALSLVVLGQGAGFMALGS

ERLRSKSLDRNSFNSGDWFNQIRWDP--------GH-GNGFG-TGLPPFDDN----REVW

GHARPLLA-D-PA-LI--PSAEVINLAAERYRELLRIRRSSPVFGLPTADEVQRRLTFPL

G--G-PG--ESPGVIVMGLD---G------T-GLDERWRRLVVVFNATDEPTCQVVPGEG

----------PLRLHPELTA--S-V--DPV-LRGASAVTKLDGAELTVPARQVAVFV---

-------

>UniRef90_A0A2S5SR28_168_1018 | Alpha-dextrin endo-1,6-alpha-glucosidase n=1 Tax=Zhizhongheella caldifontis TaxID=1452508 RepID= | E_val=6.9e-180

-----------------ARAMWLSTSRLVWP-GL-----------PAG-GTLRLYHAAQG

G-ITANAN---GIQ---GA-D-----GFHELS---STT--LETALRERFRHLA--TAPAF

ALPSAA------QADVK-----QLLKGQLVVARVS----------------QGR-VTHAT

QLQIQGVLDDVYAAE---AV---SETLGV-------SF----D-GE---------G----

-----------RPVFRLWAPTA-RSVELAIGSEPL-------------------RAMTED

TR---TGVWHY------TG-DA-A-----WVN-NAYYTYRVQVYSR-----QDGGVVTNT

VTDPYALTLNAN-----SQAAMVARL---SDAAFKPAGWDAH--AIP-AL-DAPADSVIY

ELHVRDFSVNDPTVPAAHRGKYTAFAQAD---------TDGVRHLQALASAGLTHVHLLP

TYDLAT--IPETGC-----------------VTP--------------------------

---NVVSAG---PTSEVPQATIAATKDEDCFNWGYDPRHYGAPEGSYATDA--ADGAVRV

REFREMVQALHGNGLRVVLDVVYNHTSG-------SFLDRIVPGYYYRQN-GDGFIERST

C--CDNTAPEYAMMEKLMTDTLKTWAVEYKVDGFRFDIMGHIPLSAMQAARAAVDAAA--

-----GRELLYYGEAWNFG-EVENDRQFI----QARQANLR-GTGIGSFSDRIRDAIRGG

GPFDSGDD-VIRRQGFVSGRCYDNNALN--------AGACTT-AQRNDLRYMQNLIRLGM

AGSVHDFMLNG--------QLASAY-DY-GG-QPAGYTADPQEVINYAGVHDGETLFDIN

QFKL--------P---------------TTTTAAERARAQVVALGTVLMGQGIPFLHAGD

ELLRTKSLDRDSYNAGDWFNRIDWSA---------T-TNYFGTMGLPSAEKN----EAEW

DRMRPILS-N-AN-IP--PTSAHIRATRDAVLDLLRVRSDTTMLRLRSGADVRDCVSFPD

A----AD--QRDGLIVMRVTGRKAN---NSL-CGDGRYANLVVLINAAPNAQTYAVGALT

--------GRSLVLHPVLAS--G-S--DTR-VQGATFTAGT--GTFTVPGRSVAVFVEA-

-------

>UniRef90_UPI00164C6EDD_39_906 | DUF3372 domain-containing protein n=1 Tax=Undibacterium sp. NL8W TaxID=2762300 RepID=UPI00164 | E_val=3.4e-179

-----------------ARAYWLSSKLIRWP-GM-----------ATG-SKVKLYTSRTG

Q-LKLSKG--SKPL---GF-D-----EAIMLTES-NIP--ADSSIAKRFAYTG--SDKAY

ELANSG------LKQLR-----SLQQAQVMIATED-----E----------DGQ-IIDYT

SLQVAGALDDLYKAA---SS---LNDYGA-------NL----G-KAAAT----KTA----

-----------QTQFRVWAPTA-QRISVCLYPDGKA------------KSNAI-LAMQFD

ES---TGTWHT------SD-KS-D-----LS--NKYYTYLVDVYIP-----GQG-LVRNR

VTDPYSISLTTD-----SMRSYIADL---SSSKLKPSGWDAQ--KLNPKV-KNQTDMQVY

ELHVRDFSINDTTVKPAWRGKYLAFTEAG---------TKGMKHLQALSAAGMTDIHLLP

VFDFAT--VSEAKC-----------------ETP--------------------------

---AI-KGK---ADSAEQQKLATQYADKDCYNWGYEPYHYNAPEGSYATDA--SDGAKRI

IEFRKMVMALQNAGLRVGMDVVYNHTAGAGQ-TQYSVLDRIVPGYYQRLN-QLGQIERST

C--CDNTATENSMMAKLMSDSVLLWTKQYKIDSFRFDLMGHQPRQIMEEMQARLLQET--

-----GRQIQFLGEGWNFG-EIADGKRFV----QASQLSLN-GSGIGSFSDRARDAIRGG

GHGDAGAG-IVKNQGYLNGLIHMPNALA------------DKARPKQDLLRAADMLRVGL

AGSLRQFRMQG-F-DDQ-SKQLQEI-DY-AG-QAAGYVSQPGEVVNYVENHDNQTLFDIN

AYRL--------P---------------AETSSADRARIQALALATTAFSQGVAYYHAGV

DILRSKSMDGNSFNSGDWFNRLDWSY---------S-DNYFG-TGLPPEKDN----AQFY

PYIKTALN-N-PN-IK--PTKQDIKFSRDQFRDLLKIRSSSSLFRMSTLDEIAQRLHFYN

T--G-ST--QNPLLIVAHLDGK--------Q-MPGANFSSIMYFINASQQTQILDRIKPG

--------QTPYVLHPVHRASGA-A--DQRVAKEAKYDSNS--GRFTIPALSAVVFV---

-------

>UniRef90_UPI00036B9994_42_903 | DUF3372 domain-containing protein n=1 Tax=Massilia niastensis TaxID=544911 RepID=UPI00036B999 | E_val=1.2e-178

---------------LEARAVWLDRRLIRWP-GA-----------TPD-GAWRLYHSPTG

T-IHARGG--RKVT---GA-A-----GALTLELS-TGP--APPAAAARFKYVP--SGPLL

AVRDAD------LERLG-----ALHRQQLVLVQEA-----P----------DGT-VRAAT

RVQAAGALDDLYAAA---DA---IHDLGA-------TP----A-G----------G----

-----------RTSFKVWAPTA-QQAALCTYDSATG------------RAGAV-HEMAFD

KA---SGAWSA------TL-PR-D-----LS--GSYYKYAVDVVVD-----GAG-LVRNL

VTDPYSVSLTTD-----SKRSYVADL---DSPKLKPKGWDRT--RPPNTV-RAQTDMVIY

ELHVRDFSINDPSVPEAKRGKYAAFGETR---------SNGMKHLAALARAGLTDIHLLP

VYDLGS--VPEQGC-----------------AVP--------------------------

----LPAGA---PDGDSQQALVRKTADTDCFNWGYDPYHYNAPEGSYASDP--ADGARRI

LEFRQMVMDLHAVGLRVGTDVVYNHTFIAGQ-NEKSVLDRIVPGYYHRLN-ASGGIERST

C--CDNTATENLMMAKLMIDSAELWTRHYKIDSFRFDLMGHQPRAAMERLQQRVDKAA--

-----GRRVQLIGEGWNFG-EVADGARFV----QASQLSLN-GSGIGTFSDRARDAVRGG

SAGDAGEA-MVRQQGYINGLVYDPNALG------------KE-RQLSELLRAADLVRIGL

AGSVRSYPLRT-F-DGR-VRLLEDI-GY-GN-QPAGYASQPGEVVNYVENHDNQTLYDAN

VLKL--------P---------------VDTPAAERARVQVLGMAINAFSQGVAYYHAGI

DTLRSKSLDRNSFNSGDWFNRIDWSY---------Q-DNYFG-TGLPPADDN----GKDW

ALLKPYLA-N-AA-LK--PLPSDIGFARDAFRDLLKIRASSTLFRLRGADDITQRLRFFN

T--G-PG--QQPTVIAAWLDGE--------G-YPGARFAGISYLVNVDKVERKVADESLR

--------GKRLRLHPVHAARGA-A--DAR-AKQARFDSAS--GSFTVPPRTAVVFV---

-------

>UniRef90_UPI00036A88D7_168_1019 | pullulanase-type alpha-1,6-glucosidase n=2 Tax=Caldimonas TaxID=196013 RepID=UPI00036A88D7 | E_val=6.6e-178

----------------HARAMWLSASRLVWP-NL-----------PAG-GELRLYHAAQG

G-ITVGAA---GVQ---GA-D-----GFHTLS---PTT--LEAALRERFRHLA--SAPAF

AVPVAA------QAEVR-----SLLKGQLVVARLF----------------QGQ-VTHAT

QLQIQGVLDDVYAAQ---AR---IQTLGV-------SF----D-GQ---------G----

-----------RPVFRLWAPTA-RSVELAVDGQPL-------------------RPMTED

PN---SGVWSY------TG-ET-S-----WVN-DAYYTYRVQVYSR-----QDGGVVTNT

VTDPYALTLNAN-----SEKAMVARL---SDAMFKPTGWDGH--AIP-PL-QAPSDSVIY

ELHVRDFSANDASVPAAHRGKYTAFAQAN---------TDGVRHLQALAAAGLTHVHLLP

TYDLAT--IPETGC-----------------VTP--------------------------

---TITAAG---PTSEVPQATVAATKDEDCFNWGYDPRHYGAPEGSYASDA--ADGAVRV

REFRQMVQALHGMGLRVVLDVVYNHTSG-------NFLDRIVPGYYYRLN-GDGFIERST

C--CENTAPEYAMMEKLMIDTLKTWAVEYKVDGFRFDIMGHIPLAAMQAARTEVDAAA--

-----GRPLLYYGEAWNFG-EVADDRQFV----QARQANLR-GTGIGSFSDRIRDAIRGG

GPFDAGDD-VLRRQGFANGRCYDNNALN--------GSTCTS-DQRSELHYQQNLIRLGL

AGSVHDFVLNG--------RLASSY-DY-GG-QSAGYTADPYEVINYAGVHDGETLFDIN

QFKL--------P---------------STTTASERARAQVVALGTVLMGQGIPFLHAGD

ELLRSKSLDRDSYNAGDWFNRIDWSA---------T-TNFFGTMGLPSAEKN----QAEW

DRMRPILS-N-AN-IP--PSAADIRATRDAVLDLLRVRRDTTMLRLRSGVDVRECVSFPD

A----AD--QRDGLIVMRIVGRKSD---GSP-CGDGRYANLVVLINAAPADQTYSVAALA

--------GRSLSLHPVLAN--G-S--DAR-VRGASFASGS--GLFTVPGRSVAVFVEP-

-------

>UniRef90_A0A0X8JDJ5_318_1194 | Alpha-1,6-glucosidase n=3 Tax=Actinomyces TaxID=1654 RepID=A0A0X8JDJ5_ACTRD | E_val=1.9e-177

----------------------------------------------------ALVTSPDA

T-ASLTDG---VVT---GG-T------ETALRV--AGN--LPDDVLAAHPNLK--GYVAL

SLENADGDAALGHDEVV-----TALEGQTAVVQRGTGAAGA----------EDT-VSAFT

GVQTAIAVDALYGEA---AQ---KGDLGA-------TF----E-T----------GL---

-----------LRSLALWAPTA-QKVTLLTWDTGDATGSV----PEVSGDGVR-HDAV-R

GD---DGRWVV------DN-AD-GAI---KA--GSQYLWEVTVYAP-----STGKIVTNT

VTDPYSLALTVD-----STRSVAVDL---SDAALAPAQWAST--ASP-KV-ANDASRTIY

ELHVRDFSASDETVPADERGTYLAFTESG---------FDGMKHLAQLAKSGVDTVHLLP

TFDIAS--IEEDRS---------------KQVTATV------------------------

-----PDAG---AASEEQQAAVASTADTDPYNWGYDPWHYTTPEGSYATDG-HQDGGART

YQFRQMVGALHATGLQVVLDEVFNHTAAAGQ-ASTSVLDKIVPGYYQRLD-AKGAVQTST

C--CSNTATENAMAARLMIDSVVTWARDYHVDGFRFDLMGYHSVATMKALRAALDELTVS

KDGVNGKAIYLYGEGWNMG-EIANNALFT----TATQGQLD-GTGIGTFNDRLRDAVHGG

GPFDSDHR---TYQGFGTGQYTDPNGLS---------DRSDA-DELADLQHNTDLVRLGL

AGNLKSYFFTT-S-DGT-VKKGSEI-DY-NG-QAAGYASSPEETINYVDAHDNETLYDLG

IYKL--------P---------------TSTSMADRVRMNTVSLATVALGQSPSFWAAGT

EMLRSKSLDRDSYNSGDYFNSIDWSG---------Q-TNGYA-KGLPMASSN----SSKW

AIMRPLLE-N-GA-LK--PAAADIATSDTQAETLLRLRKSTPLFSLGTAALIQSKVSFPG

S--G-TD--ATPGVIDMLIDDTAANADGSVT-DVDKALDGVLVVLNASDEATTQTIGSIK

--------GRDFALSSIQAD--G-S--DAT-VKGVAYDAAT--GSVTVPARTVAVLTDPQ

-------

>UniRef90_A0A3E0WHM1_6_707 | DUF3372 domain-containing protein n=2 Tax=Alkalilimnicola ehrlichii TaxID=351052 RepID=A0A3E0WHM | E_val=7.5e-177

------------------------------------------------------------

------------------------------------------------------------

------------------------------------------------------------

------------------------------------------------------------

------------------------------------------------------------

-T---SGIWQV------AG-DP-S-----WHDGNHYYLYEIELYVPAYIGGSSGGIKTTQ

VTDPYALGLSAN-----SERSFIVDL---NDPATKPDGWDSV--SLP-AI-EHPVDIAIY

ELHVRDFSVFDASTPATHRGKYLAFTHTD---------TAPMQHLLRLRDAGLTHVHLLP

TYDIGS--IPDIDP-----------------AAPDY------------------------

--SELQAAVAEDPASLVPQDEISAIAHDDAYNWGYDPWHYTVPQGSYATDP---NGVARI

REYRQMVKALAEQGLRVTKDIVYNHTFASGW-HEQSVLDRVVPGYYYRLT-DDGAVHTST

C--CPNTATEHRMMEKLMLDSMETWAEQYRIGAFRFDLMGHHMKSHVLAAQERVQAI---

-----DPDLYIYGEGWNFG-EVENNARGI----NATQFNMA-GTGIGTFNDRLRDAARGG

TPFDEDEA-LIDNKGVINDLYHD-----------------------NSLNGEVDRVRVGI

AANLAAYSFLR-Y-SGE-VLPGSH--------DGVGYAARPDDVITYVSKHDNQTLFDNN

VYKL--------P---------------IDTPMEERVRVQNLGISVTALSQGIPFFHGGV

DMLRSKSLDRDSYNSGDWFNRLDFTYGQSGSEAFTN-ANNWA-VGLPPAEKN----AENW

EVMEPLL--R-AV-TA--PETRHIERSHAHLQEMLQVRQSSELFRLRTAEDVKSRLSFHN

T--G-PE--QQPGLIAFTLSDNVP----GLP-QVDANWGSAIVLFNFSPEHREITLAAYE

-------GDETVALHPVQVA--S-D--DPE-LGNAQFNAAN--ATFAVPPRTTAVFVSHS

E------

>UniRef90_A0A7Z2VWZ8_49_908 | DUF3372 domain-containing protein n=1 Tax=Duganella sp. GN2-R2 TaxID=2728020 RepID=A0A7Z2VWZ8_9B | E_val=2e-176

-----------------ARAVWLDSRLATWP-GA-----------GAD-GVFKLYHSPIG

A-IAAPAG--GKVE---GA-A-----GAVVLQPF-KGT--LPAAAATRFKYLA--AGPLL

QVGDAD------VPALG-----ALHREQLVLVQED-----A----------RGI-VVAAT

RLQAAGALDDLYGAA---AG---LDRLGV-------SV----D-G----------K----

-----------RTGFTLWAPTA-QQAAVCVYDSGRS------------VVRAV-YQMGFD

AK---TGAWSA------AL-PG-D-----LS--GKYYRYAVDVPVD-----GAG-IVRNL

VTDPYSISLTTD-----SKRSYIGRL---DAPRLKPQGWDAS--AAPRTV-KNQTDMVVY

ELHVRDFSINDASVPYEHRGRYAAFTDLR---------SNGMKHLAALAKNGLTDIHLLP

VYDLGS--VPETGC-----------------AVP--------------------------

---S-PGGA---PDGTAQQALVKKTAQTDCFNWGYDPYHYNAPEGSYASDP--ANGATRV

LEFRQMVDSLHGVGLRVGMDVVFNHTFIAGQ-NEKSVLDRIVPGYYHRLD-AKGAIEQST

C--CDNTATENLMMGKLMIDSVTLWATQYKIDSFRFDLMGHQPRAVMERLQRQVNAAA--

-----GRNVQLIGEGWNFG-EVADGARFV----QASQLSLN-GSGIGTFNDRTRDAVRGG

AAGESGAG-LF-RQGWINGLVYDPNAQA------------AK-ASPADLLKAADLVRAGL

AGSIRSYPLQT-A-DDR-IRTLEAI-DY-NG-QPAGYAAEPGETVNYVENHDNQTLYDVN

VLKL--------P---------------VATSSQERARVQVLGMAVDAFSQGVAYYHAGI

DVLRSKSMDRNSFNSGDWFNRLDWTY---------R-DNYFG-TGLPPEQDN----GKDY

ALLKPLLA-N-PA-LK--PQPADIAFARDAFRDLLAVRASSTLFRLPTSDEVKRRLRFFN

T--G-SA--QVPTVAAAHLDGR--------G-YDGAGFAGISYFINVDKVGHRVTDPQAV

--------GKRLRLHPVFMAPGA-A--DKR-ATQATFDSAS--GAFEIPPRTAVVFVE--

-------

>UniRef90_A0A2S9H5C0_62_919 | Aamy domain-containing protein n=2 Tax=Solimicrobium silvestre TaxID=2099400 RepID=A0A2S9H5C0_9B | E_val=1e-175

-----------------ARAIWLNQRLIKWP-GL-----------NAS-ANFRLYYSASS

N-LHVVEG--THMQ---GA-D-----GVLALEPT-SSP--LPTALATRFNFVA--DGIIL

KLHATD------SKKVA-----QLLQQQLILVQEN-----S----------LGE-VIDST

RIQIAGALDDLYTSA---QY---IDDFGA-------TVIDRGD-S----------H----

-----------ATQFKLWAPTA-QRVSLCLYENGQG------------GAEQI-AAMQRV

SA---TGHWQY------TI-PK-N-----LT--GHYYKYVVDVVVP-----GIG-LVRNR

VTDPYSISLTTD-----SKRSYITQV---DAPQTVPNGWRQS--RAPQSI-RKQTDMTIY

ELHVRDFSINDQSVSAAHRGKYLAFTELG---------SNGMQHLATLVKASLTDVHLLP

VFDFAS--VQETGC-----------------DHL--------------------------

---AI-------NSQDDVVDPVNKTVSTACFNWGYDPFHYNAPEGSYATDP--ADGATRM

LEFRQMVMALHRIGLRVGMDVVYNHTYAAGQ-QPKSVLDRIVPGYYHRLN-ALGAIEQST

C--CDNTATENRMMSKLMIDSVVSWARNYQIDSFRFDLMAHQPRSVMEALQKKLKSSI--

-----NHPIQLIGEGWNFG-EVADGARFV----QASQLSLN-GSGIATFNDRLRDAVRGG

SASDSGAD-LVNRKGYINGLADQGNS-------------------SAGLLQVADLVRVGL

AGSLRDYQLLT-Y-DGQ-KKSLKEI-NY-NG-QPAGYVSQPTEVVNYVENHDNQTLFDIN

AYKL--------P---------------TDTTRIDRVRVQMLGAAITAFSQGVAYFHAGI

DVLRSKSMDSNSFNSGDWFNRLDWTY---------Q-DNYFA-TGLPPKPDN----EKNY

PLIKERLE-N-PL-IK--PTGNEIGMARAMFSDLLKIRASSTLFRLGTAEDIEQRLRFEN

V--G-PL--QNGSLIVAHLDGK--------N-YPGANYQSILYFINVASTEQTIRLASEK

--------GQAYVLHPVQSNVAA-A--DKRVAKEAAYDALT--GQFTIPARSSVVFVVP-

-------

>UniRef90_A0A254NBB6_45_898 | Alpha-1,6-glucosidase n=1 Tax=Pelomonas puraquae TaxID=431059 RepID=A0A254NBB6_9BURK | E_val=1.1e-174

------------------AAYWLNGHALQWP-GLQL---------QAG-ERVRLFHAASA

G-LQVGGR---AIT---GA-D-----GVLDLAPA-DVP--LP----QRLRFIG--AGPRF

VLPP--------GADVK-----GLLKSQLLLVHET-----A----------AGE-VKATT

GVQMPAALDDLYAAA---EQ---VTDLGA-------HP----S-P----------S----

-----------GTRFAVWAPTA-QRVMLCRHARTGG------------PATAV-EPMAFD

AA---TGVWRA------RV-AG-D-----LT--GQAYRFLVDVVTP-----TQG-RVRNL

VTDPYSVALTAD-----SRLSVVADL---ASPRLKPAGWDRL--PAPARV-KTATDQVIY

ELHVRDFSISDETVRPAWRGKYLAFTERR---------SNGMRHLQALARAGVTDVHLLP

VFDLAS--VPERGC-----------------KTP--------------------------

---VVPAAA---PDSEAQQAAVMAVAAEDCFNWGYDPWHFNTPEGSYATDA--DDALRRI

REFRQMVMALHRAGLRVGMDMVYNHMAAAGQ-HPKSVLDRLVPGYYHRLD-AQGRIERST

C--CDNTATEHRMMAKLMIDSAALWVREYRIDSMRFDLMGHQPREAMERLQRRVNAEA--

-----GRAVQLLGEGWNFG-EVANGARFV----QASQLSLN-GSGIGAFSDRARDAARGG

GAGEGGDA-SVRNQGWLNGLVYAPNALG------------SG-RPDEDLLQAADLIRVGL

AGSLRSVELTT-W-RGE-TRRLDAI-AY-GD-QPAGFASEPGEVVNYVENHDNQTLFDIN

TLKL--------P---------------RETSREDRARVQMLGAALVAFSQGVAYFHAGQ

DILRSKSLDRNSYDSGDWFNRLDWTY---------R-SNHFG-TGLPPRQDNFGVEGRDW

ALARERLA-D-AS-IA--PGPAEIAWSRDVFRDLLRIRASTPLLRLPSAAQIRERLSFPG

S--G-PG--QNPTLVALRLDGR--------G-LPGP--KAVMALINVAPSAQALELPTEA

--------GARWVLHPVQRQ--G-V--DARVRREARF--VA--GRFEVPARTAAVFV---

-------

>UniRef90_UPI000CD4FEDA_1107_1823 | pullulanase-type alpha-1,6-glucosidase n=2 Tax=Streptomyces TaxID=1883 RepID=UPI000CD4FEDA | E_val=3.5e-174

------------------------------------------------------------

------------------------------------------------------------

------------------------------------------------------------

------------------------------------------------------------

-------------VLSLWAPTA-QEVTLEL-------------------DGEQ-HAMTRD

DA---SGVWSV------EG-ET-G-----WH--GKPYRYHVTVWAP-----EAQAIVTNK

VTDPYSTALATD-----SARSLVTDL---GDPKLAPRGWDKA--RKP-AP-VPLAEAAIQ

ELHVRDFSIGDDT--SRHPGTYLAFTEKK---------SDGARQLKALSKAGTTHAHLLP

TNDTGS--VPEEPA---------------DRREPDC------------------------

---DLPSFA---PDSPEQQACVAEVRDQDGYNWGYDPVHFNVPEGSYATDP---DGTART

LEFREMVRSLNDNDLRVVVDVVYNHTFAAGQ-DEKSILDRVVPGYYHRLL-EDGTIADST

C--CPNTAPEHAMMGKLTVDSVVLWAQHYRVDGFRFDLMGHHPKQNLLDVRAALDELTVE

EHGVDGSAVVVYGEGWNFG-EVADDARFE----QATQENMA-GTGIATFSDRARDAVRGG

GPFDEDP----GVQGFATGLYTDPNDSP--------ANGDQD-EQRARLMAYQDIIKVGL

TGNLADFEFTA-S-DGR-RVTGADV-DY-NG-SPAGYTAAPGEAVAYVDKHDNEALYDAL

AYKL--------P---------------ARTSATDRARMQVLAMSTALFSQGPAFGQAGS

ELLRSKSLDRNSYNSGDWFNAIHWNC--------SA-GNGFG-RGLPPAWDN----EDKW

PYAEPLLT-N-PN-LT--VGCDEIRATDRAYRDLLTIRSGEDAFGLATLGEVQEQVSFPL

S--G-TD-RETPGVIVMRA-------------------GDLAVVFNATPHRQYQHHPSLV

--------GEEFTLHRVQAR--G-A--DRT-VRSATFDAAQ--GTFDVPARTVAVFT---

-------

>UniRef90_A0A1M5JLJ5_46_897 | Alpha-1,6-glucosidases, pullulanase-type n=1 Tax=Massilia sp. CF038 TaxID=1881045 RepID=A0A1M5JL | E_val=2.1e-173

-----------------ARAVWLDRYALKWP-GA-----------SAS-GSFKLYHSPLG

S-ITVANG---RID---GA-A-----GSLPLTSG-AAS--TP----SRFHFVG--AGPVL

RVPSID------TERLR-----ALHREQLVLVQEA-----A----------DGS-VIGAT

RIQVAGALDDLYGAA---SR---TPDLGA-------TP----G-A----------R----

-----------GTSFKLWAPSA-QQVAVCIYEGPDT------------PAVAV-TALQSE

RA---TGIWQA------RD-AR-N-----LS--GRYYRYLVDVIAD-----GTG-LVRNH

VTDPYSVSLSTD-----SKRSYIADL---AEPRLKPKGWDQH--QTPNRV-SAQTDMVVY

ELHVRDFSINDNTVAPSRRGKYAAFGEAA---------SNGMRHLSALSSAGMTDIHLLP

VYDIAT--VPEHDC-----------------AVP--------------------------

---S-PAGA---PDSDAQQALVMANAGNDCFNWGYDPYHYSAPEGSYASDA--ADGARRI

IEFREMVMNLHRAGLRVGMDVVYNHTFAAGQ-KERSVLDRIVPGYYHRLD-ADGKVAQST

C--CDNTATENRMMEKLMIDSVALWTRHYRIDSFRFDLMGHQPRAAMERLQARVNRAA--

-----GKQVNLIGEGWNFG-EVENGARFV----QASQLSLN-GSGIGTFSDRARDSVRGG

GASDSGLD-MFKRQGYINGLVYDANAYA------------GE-RPAADLLAAADMVKVGL

AGSVRSYRMAT-Q-DGT-IKRLDAI-DY-SG-QPAGYASEPGEAVNYVENHDNQTLYDMN

VFKL--------P---------------LETSSAERARVQILAAAINAFSQGVAYFHAGI

DTLRSKSLDRNSFNSGDWFNRIDWSY---------Q-DNYFG-TGLPPAADN----GKDY

ALLKPLLA-N-AA-LK--PAPADIAFARDAFRDLLRIRASSTLFRLRTAADINARLRFYN

T--G-PQ--QVPTIVAARIDGK--------G-YRGANFSAVNYFINVDKEARSIAIDASA

--------AARMRLHPAHQ-----A--DPR-AAQAHYDHAT--GMMHIPARTAVVFVE--

-------

>UniRef90_UPI000C9FFDE6_103_945 | pullulanase-type alpha-1,6-glucosidase n=1 Tax=Actinomyces qiguomingii TaxID=2057800 RepID=UP | E_val=1.8e-172

------------------------------------------------------------

----------------------------------------LGEEAMAAVPALR--GYLAL

SLEDEFGGRSVEREDIT-----LLLAGQLAVVQRT-----AAST-------GGW-ITAFT

GVQTWPLLDHLYADA---AARDGSAPLGA-------SI----GPD----------G----

-----------VPTFSLWAPTA-VDVALLAWPTGDPTGCA----PLIDADPIR-VPADRV

DAGSWDGRWKV------GG-PA-ATAAGIDA--GSQYLWEVRVYVP-----ATGRVETNL

VTDPYSRALTVD-----SRRSVVVDL---GQRALKPSSWREN--LSP-VV-ANDAARAIY

ELHVRDFSAADASVPEQLRGTYGAFAV----------VSDGTRHLRSLAAAGIDTLHLLP

TFDFCS--VPEDRA---------------AQRAPHI------------------------

---PRDA-S---PASRRPQAAITAVADQDAYNWGYDPWHWLVPEGSYAREG-RQDGGARV

YEFREMVGAIHGMGLQVVLDQVFNHTGASGQ-EAHSVLDRIVPGYYHRLD-AAGNVEMST

C--CNNIATEHRMAERLMIDACVSWVVDYRVDGFRFDLMGYHSLETMARLRDALAQV---

ADDAVGHSIYLYGEGWNMG-EVANNALFT----QATQGQAG-LLGIGTFNDRVRDAVHGR

SD-DTDPR---VPQGLGNGELTEPNGFD---------DRSEE-TKRADLAWRTDLVRLAL

AGNLRDFELPG-S-HGQ-WARGEEI-RY-GD-EPAAYGLVPTDSINYVDAHDDETLFDRL

AYKL--------P---------------LHTPMAERIRMNTLCLATVTLGQSPCFWAAGT

ELLRSKSLDTDSYNSGDHFNAIDWTG---------Q-DNGWG-RGLPPASRN----FDAW

VVQAGILM-R-ED-LR--PTAADIATARQQALDLLRLRRSSQLFSLGSAALIRERVSFPT

---G-DF--DAPGLVVMLIDDGAG-----DS-DIDPDLDGLLVVINPAPGTVEQRVDALV

--------DRYFELSDIQLE--G-A--DPV-VKGTRFYANT--GAITIPGRTVAVLYE--

-------

>UniRef90_A0A7Y1XRL6_297_934 | Pullulanase-type alpha-1,6-glucosidase (Fragment) n=1 Tax=Acidimicrobiia bacterium TaxID=2080302 | E_val=1.3e-171

-----------------AQAHWPVSEALAWA-VP-----------DGD-AEYRLYHSPNG

S-LELTAE---GLV---GG-D------FIELTID-DSG--LPVGTIEKFPHLV--DLTAL

AVPD--------GTDID-----TLLTGQLAVAAIA-----D----------DGR-VLDAT

SLQIPGVIDDRYG------S---TAPLGV-------TL----S-G----------S----

-----------TPTLTVWAPTA-RNVRLHVFNTGSG------------TRADLIVPMD-R

SQ---QGVWSA------RG-EA-S-----WV--GKYYLYEVEVFAP-----TVGSVVRNM

VTDPYSVSLATN-----SQKSQIIDL---TDPSLAPLGWDDL--EKP-AL-DAPEDIVLY

ELHVRDFSAIDESVPAEYQGTFKAFTLPR---------SAGMDHLSSLANAGLTHVHLLP

VFDIAT--IDENEA---------------NRQEPEV------------------------

---DLASFP---VTSSEQQAAIGEVRDLDAFNWGYDPYHYTVPEGSYSTSP---DGTARI

LEFREMVQSLNESGLRVVVDVVYNHTNASGQ-GQKSVLDRIVPGYYHRLD-ANGLVATST

C--CANTATEHDMMRRLMVDSVVTWAEQYRVDGFRFDLMGHHMKSDMQAVRAALDEV---

-----DPTIYVYGEGWDFG-EVGQNARGV----NATQLNVG-ELGIGTFNDRIRDAVRGG

SPF-GGL----QEQGFATGLYYDPNVTD---------QGPAG-DQLADLLLKADQIRVGM

TGNLADYAFES-F-TGE-SITGAEV-DY-NG-APAGFTTDPQEVIKFVSAHDNDTFFDIV

QTMA--------P---------------LDMS----------------------------

------------------------------------------------------------

------------------------------------------------------------

------------------------------------------------------------

------------------------------------------------------------

-------

>UniRef90_A0A345ZNQ0_59_921 | DUF3372 domain-containing protein n=1 Tax=Lysobacter sp. TY2-98 TaxID=2290922 RepID=A0A345ZNQ0_9 | E_val=7.9e-171

S----TTLSAAADPRLDARAYWLDARTLRWP-GM-----------PDD-GRYRLVGSHDA

A-LRIDAS--HHVQ---GA-D-----LALQPRSP-TRP--LAPEAADAFRFLP--AGAQR

QLSDVD------ARTLR-----ANVATQWWLVRED-----D----------EGR-AVDAT

YLQSPGLLDAAFDGD---AA-----ELGP-------QV----E-R----------S----

-----------GTRFGVWAPTA-RRVALCLYGRGSA------------NA-MP-VALKRD

EA---SGAWTA------RV-PG-D-----VR--GRSYLFLTDVFVP-----GLG-VVRNR

VTDPYSVTLTPD-----SKRSVVEDLGDTSNPHLAPAHWPDH--ARPAPA-ENPVDMTVY

ELHVRDFSVADASVQTPHRGKYLAFTDVT---------SAGMKHLAALRAAGLTDVHLLP

VFDIAT--IPERGC-----------------ASP--------------------------

---VILKGA---PDSPAPQAAIAKVAHRDCYNWGYDPQHFGAPEGSYATDA--ADGAVRI

REFRAMVQALHALALRVGMDVVYNHTTTSGQ-ADTSVLDRIVPGYYYRLD-ANGKVEHST

C--CENTATEHRMMARLMADTLVRWARDYGIDSFRFDLMGHQPRAAMERAQRALRDAV--

-----GHDVPMLGEGWNFG-EVANGARFV----QAAQGRLD-GTDIATFSDRARDALRGG

GCCDSGEA-LRANKGWLNGLAVVP-------------------ASRTDALHAADLIRAGL

AGTLRDYTTTF-A-DGR-TAPLAAL-DY-AG-QAAGYATAPDEVVNYVENHDNLTLFDVN

ALRL--------P---------------AGTPRDERARAQVLGIAVVALSQGVAYYHAGI

DVLRSKSLDRNSFESGDAFNRLDWTY---------T-DNGFG-VGLPRSADN----GKDW

PLLAPFLR-D-AS-IK--PAPREIAWTRDVFRDWLKLRATTPLLRLRNASDVERRLSFLN

T--G-PT--QNPAVIVGHLDGT--------G-LPGA--RELLYLVNTSPEAQTLALPGEA

--------HKAYVLHPVQRA--G-T--DAR-VREARYD-PT--GRVTVPGRTAAVFV---

-------

>UniRef90_A0A1H2BU67_257_1016 | Alpha-dextrin endo-1,6-alpha-glucosidase n=2 Tax=Actinoplanes derwentensis TaxID=113562 RepID=A0 | E_val=4.1e-170

------------------------------------------------------------

-----------------------------------PGG--LFQAQSRLFPHLR--AYRAY

AVPELP------DDELR-----ELLRDRLLVEGPD-----------------GS---AVT

AVQIAGVLDDLYPD----AA---TAELGP-------VL----D-DE---------G----

-----------RPRLSVWAPTA-QVVELELFRQTG-------------DEPRI-HPMTRD

DT---TGVWSV------TG-KR-K-----WL--HRWYRFRVRVWQP-----AVQRVATTS

VTDPYSVSLAVD-----STHSQIIDF---DDPALAPPGWADL--VKP-PA-VAPARMQIT

ELSVRDFSVFDTTVPSPERGGFLAFTRDE---------SNGMRHLRSLADAGLTHVHLLP

VNDFAT--VPDRWA---------------DQAAPMC------------------------

---DLTSFP---PDSAEQQRAVMEVADQDGFNWGYDPWHWTTPEGSFATDP---DGPDRI

RQMRAAVTALNQTGLRVVLDVVYNHTMGDGL-DRFSVLDRIVPGYYHRLI-ADGSTAEST

C--CPNTATEHTMMARLVVDSLVTWAREYRIDGFRFDLMGHHPKSNILAVREALHRI---

-----DPDIALYGEGWNFG-EVAYDARFV----QATQVNMA-GTGVGTFNDRLRDAIRGG

SAH-GDP----GVRGFATGLGSDVP------------------------LWLHDQIKVGL

SGALATYRFTG-H-DGH-EHIGAEI-G-------AGYAHDPGEAVNYVDAHDNEILYDAM

AFKL--------P---------------CGISQVDRARMQVLALAMVVLSQGAGFVAFGT

ERLRSKSLDRNSYNSGDWFNGVRWDP--------AQ-GNGFG-LGLPPEADN----RDQW

DWARPLLG-D-PD-LV--PAPDVIGLAAERYRELLRIRRSSPVFGLPTAEEVQRRLSFPR

S--G-PG--EQPGVIVMDLD---G------T-GLDARWGRTVVVFNATGTPARPDIPDAG

----------GLRLHPELTG--S-V--DPV-LR---------------------------

-------

>UniRef90_A0A7J0CLQ0_923_1648 | Alpha-dextrin endo-1,6-alpha-glucosidase n=1 Tax=Streptomyces microflavus TaxID=1919 RepID=A0A7J | E_val=9.7e-170

-----------------AEAQWIDANTVVWK-VKT----------TDA-TSQQLVYAKKG

G-ISVVGG---ALS----E-E----GQWLRL--T-QAA--LSEAQKAEYPHLK--DYPAF

TVDTRD------RDRVR-----ESLRGQLIATQRA-----A----------NGA-LLAAT

GVQTAGILDDLYGKK---AA---SASLGP-------VF----R-G----------G----

-----------TPTLSVWAPTA-TSVALEL-------------------DGRT-HPMKRD

DR---TGVWSV------TG-KK-D-----WR--GKPYRYAVTVWAP-----TVQKLVTNK

VTDPYSTALTAD-----SARSLVVDL---TDPKLAPRGWSGL--KKP-AA-TPLRDAQIQ

ELHVRDFSITDRT--SAHPGEYLAFTDTR---------SKGMRHLKKLADSGTSYVHLLP

VFDIGT--IPEKKK---------------DQQKPAC------------------------

---DLSVYA---PDSEEQQACVSEAAAKDGFNWGYDPLHYTVPEGSYASDP---DGTKRT

VEFRQMVQGLNQAGLRTVMDVVYNHTVASGQ-DDKSVLDKIVPGYYQRLL-EDGTVATST

C--CANTAPENTMMGKLVVDSIVTWAKEYKVDGFRFDLMGHHPKENVLAVRKALDGLTLA

KDGVDGKKIILYGEGWNFG-EIADDARFV----QATQKNMA-GTGIATFSDRARDAVRGG

GPFDEDP----GVQGFATGLYTDPNSSP--------ANGTRA-EQKARLLHYQDLIKVGL

TGNLAGYTFTD-T-SGR-TVKGSDV-DY-NG-APAGYAAAPGDALAYSDAHDNETLFDAL

AFKL--------P---------------AGTSAAERARAQVVAMGASVLSQGPSLSQAGT

DLLRSKSLDRNSYDSGDWFNALHWDC--------RD-GNGFG-RGLPPAADN----QDKW

SYAKPLLT----------------------------------------------------

------------------------------------------------------------

------------------------------------------------------------

-------

>UniRef90_UPI000401010E_37_885 | DUF3372 domain-containing protein n=1 Tax=Lysobacter sp. URHA0019 TaxID=1298861 RepID=UPI0004 | E_val=1.1e-168

----------------DARAYWLDSATLRWP-GK-----------SPG-AQYRLAASRNA

A-LRIDRH--QRLQ---GA-D-----LSLPLTQS-DAR--LTGDVAKAFGFVP--AGAQF

ALEHDK------ARTVR-----KNIAAQWWMVEQD-----A----------NGR-VTDAT

YIQSPGLLDAAFDGD---RA-----TLGP-------QV----E-R----------T----

-----------ATSFGVWAPTA-RNVSLCIYPTATD------------PATAP-VAMQRD

DA---TGAWTT------RI-SG-D-----LH--GHAYLFLVDVFVP-----GIG-VVRNR

VTDPHSVTLTTN-----SQRSVVEDLAESRNARLTPSGWSSH--ARPPPA-ASNVDMTVY

ELHVRDFSVADTSVPAEHRGRYLGFTDTA---------SNGMKHLAALRDAGLSDVHLLP

VFDIAT--IPEQGC-----------------VTP--------------------------

---DVVRGA---PDSPAPQAAIAAISKTDCYNWGYDPQHFGAPEGSYATDA--SDGAVRI

REFRAMVQALHALGLRVGMDVVYNHTTTSGQ-ADTSVLDRIVPGYYYRLD-AKGAVEHST

C--CENTATEHRMMARLMADTLVRWARDYGIDSFRFDLMGHQPRAAMERAQRALRDAV--

-----GHDVPLIGEGWNFG-EVANGARFV----QAAQGRLN-GAGIGTFSDRARDALRGG

GCCDSGED-LLRRRGWLNGAA----------------------HTHADALRDADLIRVGL

AATLRDYTTTF-A-DGR-TAPLSSL-DY-SG-QPAGYASAPTEVVNYVENHDNLTLFDVD

ALRL--------P---------------ADTTPAERARVQALGIAIVALSQGVAYYHAGI

DVLRSKSLDRNSFESGDAFNRIDWTY---------T-DNGFG-VGLPREVDN----GKDW

PLLAPVLR-N-TS-IK--PSPDDIAWTRDVFRDWLRLRGSTPLLRLRTGDDVQRRLTFEN

V--G-GS--QNPAVIVGHLDGA--------G-MRGA--RELLYLVNTSPTAQTLDLPREA

--------NKHYVLHPVLQH--G-R--DAR-VRDARYD-PR--GRVTVPGRTAAVFV---

-------

>UniRef90_A0A5C7RGJ5_48_929 | DUF3372 domain-containing protein n=1 Tax=Xanthomonadaceae bacterium TaxID=1926873 RepID=A0A5C7R | E_val=1.5e-167

------------DTSAVARAYWLDRTLIQWP-TAPRAT--GHTADPAS-GRFRLYHSSKA

L-LRAQAG--KTVE---GA-E-----GVIELSQV-KAE--LPTALAARFRFIG--AGPRL

ALPAYA------QARLP-----DLLRAQTLLVRED-----A----------HGR-VIEAT

HLQSAAALDDVYAGA---ED----AMLGAFRYGRPERS----E-D----------F----

-----------ATRFALWAPTA-RAVAVCLFPNDHA------------GRVTA-HAARRD

DA---TGVWTL------DL-PG-S-----RH--GQVYAYLVDVYAP-----GVG-VVRNR

VTDPYSAGLLAD-----SRRSVALDL---AHPTTMPEGWAHA--ARPAAL-AAAVDMTIY

ELHVRDFSANDASVRPRWRGKYLAFTEPD---------SAGMRHLHALREAGVSDVHLLP

VFDFAT--VPERDC-----------------VIP--------------------------

---EIPRAA---AHSDAQQAAIAPVAARDCFNWGYDPYHFNAPEGSYATDA--DDATARI

REFRAMIMALHAAGLRVGMDVVYNHTSTSGQ-APKSVLDRIVPGYYHRLN-ANGEVERST

C--CANTATEHRMMAKLMIDSAALWTKHYRIDSFRFDLMGHQPRAAMERLQQAVDAAA--

-----GRRVELIGEGWNFG-EVADGARFV----QASQGALQ-GSGIATFSDRARDALRGG

GCCDSGAA-LFANRGLLSGLDRAPNARD------------ATPDHDDALRRAADLARVGL

AGTLRDAQMIA-A-DGA-RKRFSEI-DY-AG-QPAGYVAEPGEVVNYVENHDNPTLFDIL

ALKL--------P---------------ADTSADERARAQSLGVAFVAFSQGIAYYHAGI

ELLRSKSLDRNSFDSGDWFNRIDWSG---------Q-DNGFG-GGLPPASDN----GRDW

ALLEPVLS-N-DA-IK--PRPEQIAWMRAAFGDLLRIRRSSTLFRMREAREVHARLSFRN

V--G-PK--QNPRVIAAHIDGR--------G-YPGAGFRDVLHAINVAPEAQTLVMPEEA

--------GKPYLLHPVHRAPQA-A--DQR-AREARFASRD--GRLTVPGRTAVVFV---

-------

>UniRef90_A0A563EL37_998_1781 | Alpha-dextrin endo-1,6-alpha-glucosidase n=1 Tax=Lentzea sp. FXJ1.1311 TaxID=2591470 RepID=A0A56 | E_val=1.9e-166

------------DDLTKSKAIWVAKDLVVWD-VPV----------IAS-DGYRIEY----

-----------------GG-R------VLRLVPS-------TSPVPDRFPHLR--GKPVF

QV----------RGYAD-----GALREKLAAVHTD-----S----------SGA-IRHRT

GVQIAGVLDDRYAAA---AT---KLTYGP-------SF----D-H----------G----

-----------RASVRLWAPTA-RTVKLRLFDKPSG------------EPVKV-VELKRD

DA---SGSWSA------SG----N-----WK--DEYYQFEVTNWQR-----SI------V

VTDPYSVALAVN-----STHSQFADL---GDGRTMPGGWSG-------DV-ARGIGGAIT

ELHVRDFSINDQSVPAGERGTYKAFTHKD---------SVGMRHLAELAKAGMDTVHLLP

TFDIAT--IPEKRS---------------DQATPAC------------------------

---DLPSLP---ADSEQQQACVGAVAAKDGFNWGYDPFHYDVPEGSYAVAQ---DGWARS

REYREMVKSLHDNGNRVVVDVVYNHTAAHGD-APTSVLDKVVPGYYQRLN-LDGSVANST

C--CSNTATENAMMGKLVVDSVVRWARTYKVDGFRFDLMGHHPKANILAVRAALDALTVA

RDGVDGRKIGIYGEGWDFG-EVAGNARFE----QATQANMA-GTGVGTFSDRLRDAVRGG

GPFDDDP----RVQGIGSGLAGDLNGSP--------ANG----DVAARLSNYSDLVKLGM

AGNMAAYRFQS-T-AGP-VVSGRDV-PY-NG-APAGYTGAPGEAITYADAHDNETLFDAL

MYKL--------P---------------PGTSVADRVRMQQVALAPVLLGQGRPFVHAGT

EFLRSKSLDRNSFDSGDWFNPYDPSL---------R-DNGFG-RGLPPAADN----QSKW

PYARPLLG-T-AR--P--SSSSDMRTSLDGTLELLRIRRSSPLFSLGSLDLVQQKVSF--

-----PA--AEPGVIVMHVDDTVG------P-DADPARRGVLVVINPYPT----------

---------------------------D--------------------------------

-------

>UniRef90_A0A3S2TPB7_168_1011 | DUF3372 domain-containing protein n=2 Tax=Rubrivivax albus TaxID=2499835 RepID=A0A3S2TPB7_9BURK | E_val=3.3e-165

-----------------ARALWLDGRHLRWP-GA-----------KAG-GTVRLHHAGTG

Q-LRVVDG---RVA---GA-Q-----AVAALEDL-AAP--LPAEVEAAVRWAG--AGRTL

RLRDAD------AAELR-----RWLRGDLLLTLED-----S----------EGR-VSAHT

RVQLPRALDALYAPA---HA---LGDLGV-------QV----D-A----------T----

-----------GATMRVWAPTA-QTVALCLHA--PG------------APVRV-MPMQRD

DA---TGSWQA------RV-DG-V-----RD--G-FYTYLADLFVP-----GHG-RVRNR

VTDPYSVGLDAD-----SQRSWIGRL---DDPRTEPPGWRTQ--AAPPPP-RSMTDMVIY

ELHVRDFSRDDATVRPAWRGKYLGFTEPA---------SDGMRHLRALADAGLTDVHLLP

VFDLAT--VPERGC-----------------AVP--------------------------

---TIPRAA---PDSPEQQAAVAAVKDRDCYNWGYDPWHFNAPEGSFATDP--DDAPGRI

REFRAMVVALHAAGLRVGMDVVYNHTTAAGQ-HPKSVLDRVVPGYYHRLN-AQGEVERST

C--CDNTATEHAMMAKLMSDSVLLWARDHRIDGFRFDLMGHQPKAEMLAIRDRLRRET--

-----GRAVPLIGEGWNFG-EVADGARFV----QASQLELN-GSGIGTFSDRARDAIRGG

GAGDGGEA-QVTRKGYVNGG---------------------------ATAATADMVRVGL

AGSLRGLRMTT-H-DGG-ERALQDI-AYGGG-QPAGYVREPSEVVNYVENHDNQTLFDVN

VFKL--------P---------------PETTPDERARVQMLAAAINMFSQGVAYFHAGI

ETLRSKSMDRNSYDSGDWFNRVDWTL---------Q-DNFFG-TGLPPAWDN----EASW

PWMRPLLA-R-AAQIR--PRPQDIRFARDQFLDLLRIRASTSLLRLPSAAAIAQRLQFPN

T--G-PG--QVPGVVVGLLDGR--------G-LPDAGFATLAYVINVDTGVRTLNLPELA

--------GGGWALHPVHRAPGA-A--DAR-PKRAQLD-AQ--GRLAVPPRTAVVFV---

-------

>UniRef90_A0A285ING7_226_1092 | Pullulanase n=5 Tax=Rheinheimera TaxID=67575 RepID=A0A285ING7_9GAMM | E_val=2.5e-163

-------------------GHWLDTNTFIWN-ATAA---------DTA-PVVKLFHSADA

D-LEVNNN---DEL---EG-S------SIELT---PAE--LSDEQKALVPHLA--NAAAF

SVDFT-------ADEAK-----AMAKNQLALAAYN-----S----------DGE-LVAAS

YVQVAKVLDALYASSDNSAL---DASLGV-------HY----A-D----------G----

-----------NITAAVWGPTA-RSMTLKVYNDDKT------------VAAS--HPMSLD

EQ---TGVWSY------SA-AS-SA----LD--RKFYRYEVTVYHP-----LTRAVETIE

STDPYSVSVSTN-----GRYSQFVNL---ADADLAPEGWSER--TVP-VV-ANPEDIVIY

EGHVRDFSVRDMSTSEANRGKYLAFTEME---------SAPVSHLKKLADNGLTHFHVLP

LNDQAN--IEERANRRINLTDTVAELCALNAEAPVC----------------GVESDSAT

LLSLLEGYL---PYSDDAQALVESIRRIDGFNWGYDPHHFIAVEGSYASDP---EGAVRI

KEVRAMVKALNDIGLRVALDVVYNHTSSSGL-FDNSVFDKLVPGYYHRYNEVSGAIERST

C--CENTATEHKMFDKFIKDSLVILARDFGFNDFRFDIMGHHPKDNLLAAREAVRAV---

-----DANTYFYGEGWNFG-EVADNRLFT----QAKQQDMA-GSEVGTFNDRIREGVRSA

AIF--------------SGVNDDGN------------------------LAEQDIIRISL

AGTLKDYVLKN-Y-RDI-TGPASSA-NW-NG-QAAGYAEDPADIINYVSKHDNETLWDIL

QSRL--------P---------------ETLSVTERVRAQNMAIAIPLMSQGIPFLQMGG

DFLRSKSMDRDSYDSGDWFNYVDFTM---------Q-TNNWA-VGLPNAEKN----RDNW

DSIAGLFL-N-PQ-TQ--VSDTDIMFASDVFTEFLAIRSGSPLFRLTTADDVMERVGFHN

I--G-SN--QTQGVIVMSIDDGSG-----LT-DLDPMHDALVVMVNGTAQEQSHTVPTAA

----------GFDLHPMQQA--S-V--DSM-VSMASFTAGTDEGTFTVPAYSMAVFVKA-

-------

>UniRef90_UPI000C07D975_103_826 | pullulanase-type alpha-1,6-glucosidase n=1 Tax=Actinomyces minihominis TaxID=2002838 RepID=UP | E_val=9.1e-160

------------------------------------------------------------

------------------------------------------------------------

----------------------------------------------------GR-VLARS

GIQIARVLDDLYPG----AS---AETLGV-------GW----D-S----------G----

-----------RPILKLWAPTA-QNVQLRLGSG---------------PEAAL-VPAVRD

D----NGVWLV------EG-EA-D-----WE--DRPYLWEVRVYVP-----ALGSLVTNR

VTDPYSVGLSPD-----STKSVILNR---SRERWTPKGWGSA--RAA-KL-RHPAAQSIY

ELHVRDFSIWDSSVPEQVRGTYRAFLERD---------SAGIRALRNLVAAGITTLHLLP

TYDIATGSIPEIRS---------------EQRVPEV---EGIALLP----------ENEE

QLEKVSGWS---RSSRLQQGAVSEVANQDGFNWGYDPAHWMTPEGSYATED-NQYGGGRT

LEYREMIEALHGLGLCVVQDVVFNHTFGGGQ-CRDSVLDKIVPGYYHRLS-PAGEVETST

C--CPNVATEHLMAQKLMVDAILDAAVHYRIDGFRFDLMGHHSLQNMLAVRAALDALTVE

EDGVDGRSVYLYGEGWNFG-EVADDVLFI----QATQRNIG-GSGIGVFNDRLRDAVRGG

NPMDQNHS---ERQGFATGLLVDPNEAQNL-----GDKAARQ-TAQAELYRLMDLVKISL

IGAIRDYPLPL-S-DGSGTVLSQDI-NY-AG-QDAAFAEEPSECVNYVEAHDDESLYDSS

IWKL--------P---------------LTTSMEDRVRAQVLANATVALGQGVAFWASGT

ELMRSKSLDRDSYNSGDWFNAIDWTG---------Q-RSQFG-RGLPSAGRN----AEYW

SHMAPLLE-M-EQ-LR--PDAAAMAMCREQSLELLRTRANEPLLTLGSADEIRARLRFLP

T--G-EAGADRPGVIAMHIDGE--------------------------------------

----------------------GYE-----------------------------------

-------

>UniRef90_UPI00058D369A_885_1626 | pullulanase-type alpha-1,6-glucosidase n=1 Tax=Catelliglobosispora koreensis TaxID=129052 Rep | E_val=4.4e-156

-----------------SFAQLLDRSTLAWK-KAN----------PDG-KVYGLAVFPNG

G-ASIVDG---KLA---GT------FSTVAMTAK-PNG--LTEAQRERFPQLW--EYPTF

GLEIKK----------------SDLRGQVIATESD-----H----------TGK-LLSAT

GVQIAGAIDDHYAP----AA---SEKLGV-------IY----E-R----------N----

-----------QPTLKLWAPTA-HKVELELGD-------------------TV-VPMRLD

ER---TGVWSA------SG-KR-D-----WD--GKSYRYRVTTWQG-----------TRS

VTDPYSVALTPN-----STHSVIAQL---GAAKVE----------------KFDGRPHVQ

EVSVRDFSITDASVPAELRGTYGAFTV-D---------SAGTRHLKALAEAGVTHLHLLP

AFDFAT--IPELRS---------------DQAVPAC------------------------

---DLASLP---ANSPQQQECVDAVRARDGYNWGYDPLHYTVPEGGYAVNP---AGTARN

GEFKAMVDALHAKGLRVVMDVVYNHTSAP------SPLDSVVPGYYHRLA-ADGSIETST

C--CLNTAPENMMMGKLVVDSVVTWASAYGVDGFRFDLMGHHPKANMIAVREALDKLP--

----NGKSILLYGEGWNFG-EVANNARFV----QATQQNMA-GTGIGTFNDRLRDAVRGG

GPFDDDP----RQQGFASGL-----------------------GAGAKLSNYEDLIKVGL

TGNLADYKFTG-S-SGS-VVRGRDV-LY-NG-SPAGYTATPSEGVTYVDAHDNEILFDAL

AYKL------------------------TGVSPADRARMQVLALSTVVLGQGTGFVTFGS

DRLRSKSLDRNSYDSGDWFNAVEWDC--------AK-GNGFG-RGLPPAWDN----AGKW

QYAGPLLA-D-PS-LV--PSCADIALATARYQELLRIRKSSKLFALSTLDEVQRKVSFPV

T--G------TSGVITMVLRD---------------------------------------

---------------------------DRE------------------------------

-------

>UniRef90_A0A7L5YPG7_351_1155 | Pullulanase-type alpha-1,6-glucosidase n=1 Tax=Anaerolineae bacterium TaxID=2052143 RepID=A0A7L5 | E_val=9.9e-153

------------------------------------------------------------

--------------------A---------------------------------------

------------ADDAK-----HLLKGQVAVAGYD-----G----------GGA-RVDAT

RVQIQSALDALYAAN---AK---TQTLGV-------AY----S-G----------G----

-----------APSVKVWAPTA-KSVTLKRYATSAG------------AEVGS-HAMSLD

PA---SGVWSV------TG-DA-S-----WD--RQFYLFDVEVYVP-----SVDGVVHNL

VSDPYAVSLSQDGVATFDVRSQFVNL---ADADLKPAGWDSL--SKP-AL-VNFEDSVIY

EVHVRDFSANDSTVAAADRGKYTAFTYDGAGPHPNTTLSNGMSHLQQLQQAGLTHIHLLP

AFDIAS--VIEPAA---------------ERTGP--------------------------

---AVPVAP---RNSQDQQAAVGATRTTDSFNWGYDPFHYGLPEGSYSTNP---DGVQRI

LEFRDMVSALNQNGLRVVMDVVYNHTAASGQ-GDKSVLDRVVPGYYYRYD-ANGALYTTS

C--CDDTATEYEMMQKLMVDTVVRFATDYKVDGFRFDLMNFHTRQNMLDLQAAVNAVS--

------PQIYLYGEGWDFG-SAKEKGLTTCPNCYAQKYNMT-GAGIGLFNDIIRDAAHGG

YSEDPTG---IRKQGFINGLSYDWNGYS--------YNN----RNQSDLWDATDRLRSAL

RG--------------------SGT-DW-NG-QGAPFTDDPQEAVNYVEKHDNETLFDQN

IFKL--------PNGDGSGNPGWIGSGIPTTSMADRVRPQNMGQSIIGLAQGIPFFQMGS

DILRSKSLDRNSYDSGDWFNKVWWDK---------S-SNNFG-QGLPPAWDN----SSRW

PIMGPLLA-N-AG-LD--PSTANMNFAASHLRETLRIRQXPPLFRLTTEAEINARTAHYN

T--N-NS--Q-DALIVLRLSDEPA------P-DLDPNWENILVFFNANTLAQSVTIAGAN

----------GFTLHPLHTN--G-VDDDPVITGGATFNDAS--DTFTIPARTTAVFVSTQ

TLAP---

>UniRef90_A0A3C0HKX0_75_869 | DUF3372 domain-containing protein n=1 Tax=Xanthomonadaceae bacterium TaxID=1926873 RepID=A0A3C0H | E_val=2.3e-148

----------------HQRAAWLDATRLRWT-AP-----------ADA-ASVVLAVASTP

A-IEAGTG--VALG---GA-E-----RRHPLTRL-T----SPGEIDRRFSHIG---GDVF

ALD---------AAVPR-----AGWRAAMLVVALD-----A----------QGR-VLDAT

GIQNAGALDAVFAPA---AQ---SALLGA-------RI----D-A----------GAT--

-------AAPRSTRFALWAPTA-SRVSLCLFHDAVRDDEA-------PPTIEA-LPMRTD

HA---SGVWTR------TV-EA-D-----LR--GRRYAFLVDVVVP-----GVG-LVRNR

VTDPYSLGLDAD-----SQASVVLDL---DDPATQPEGWASD--RRPDTV-QAHTDMVIH

ELHIRDFSRDDDSVPEAHRGKYLAFTHPD---------TVGMRHLRALADAGLTDIHLLP

TYDLAT--VPERGC-----------------VTP--------------------------

---TLPESD--GPAGERVQAAIGAVRGRDCYNWGYDPWHFDVPEGSYATTP--DDAASRV

REFRAMVQALHQAGLRVGLDVVYNHTMASGQ-DRQSVLDRIVPGYYHRLD-AEGAVETST

C--CANTATEATMMAKLMIESAVIWARDYRVDSFRFDLMGHQPKAAMLALQRAVDEAT--

-----GRRIHLLGEGWNFG-EVQNGARFD----QAAQGVLN-GSGIATFSDRMRDAARGG

RAGDRGEA--LNAQGWLSGLHFAPNAAN--------AHRPEA-ETRAELRQAEALIRVGL

AGTLVDFETFD-A-DGV-VRPLREF-RY-GD-GPAGYASQPGEVVNYTENHDNLTLFDSN

ALRL--------P---------------LDTPMHERARLQVLGNALVLLAQGVGYLHAGQ

ELMRSKSLDRNSYDSGDAFNLIDWSG---------E-RSAFG-IGLPPAWDN----EDSW

PQMRPVLERG-PA-LA--PRAEDAGFVLEWTLALLRLRRDEPLLRLDSSDAVRAALRFHP

VE-G------EPGLVIGEL-----------------------------------------

------------------------------------------------------------

-------

>UniRef90_A0A3A2I349_518_1074 | Alpha-dextrin endo-1,6-alpha-glucosidase n=2 Tax=unclassified Vibrio TaxID=2614977 RepID=A0A3A2I | E_val=1.6e-144

------------------------------------------------------------

-----------SIC---GT-E---------------------------------------

------------------------------------------------------------

------------------------------------------------------------

------------------------------------------------------------

------------------------------------------------------------

------------------------------------------------------------

------------------------------------------------------------

--DDAK--VIED------------------------------------------------

---VLNRYD---PTTGAAQALMNDLRMLDSFNWGYDPFHYTVPEGSYATDP---NGSKRI

LEFREMVQATHKMDLKLVMDVVYNHTNASGV-NDKSVLDKIVPGYYHRLNVNTGGVENST

C--CDNTATENLMMGKLMVDSLLVWADDYKVDGFRFDLMGHQPKDVMVYALEQVRKI---

-----DPNTLFYGEGWDFG-EVANNARFD----QANQINMA-GTDIGTFSDRLRDAVRGG

SPFDGAGTGLRKNQGFGNAAY--PNELQ----------TGSQ-DEIDGALHNQDLVRLGM

AGNLADFILID-Y-TGE-TKLGKNV-DY-NG-APAGYTKAPSENISYVSKHDNQTLWDNN

AYKV--------D---------------AGTSSADRARMQSVSLSTVMLGQGIPFIHMGS

ELLRSKSMQRDSYDSGDWFNRVFFDG---------T-DNNWN-VGLPREDKD----GANW

ALIKTIIG-D-ST-TK--PDTTDIEQTKQQFLELLTIRSTSELFRLDTAQEVMNRVDFHN

V--G-QN--QVEGLIVMSIDDGTS----AGA-DLDQNYDAIVAVVNSTATEQSFKVEG--

--------ATSFELHNVQKN--S-M--DTT-VKGASF--SG--DTFTVPALTTAVFVQPQ

N------

>UniRef90_A0A2N1C8K5_220_1076 | Alpha-dextrin endo-1,6-alpha-glucosidase n=1 Tax=Colwellia sp. 75C3 TaxID=888425 RepID=A0A2N1C8K | E_val=7.9e-142

------------------AAHWIDSNTFAWN-VNF----------EQV-TSVKLHHSATA

D-IGASEDN-EDVI---TG-T------VVELT---EVD--LTDEQKSAAPLVA--DWPAF

TASWT-------VEEAK-----AITKNQSVLVGYN-----A----------EGV-AVAAT

YVQADLVLDDLYTNGEMDAD---EATLGV-------VY----D-N----------G----

-----------AINVSVWSPTA-QNLTLNVYNTAKE------------MVSS--HDMTED

SM---TGIWSY------AG-DA-S-----LD--RQFYRLALTIYHP-----QNKAIEEIE

STDPYSLSLSTN-----GEYSQFVNL---SDEDLQPEGWDGH--VVS-TI-ENYEDAVIY

EGHIRDFSALDESTSVENRGKFLAFTETE---------SAPMQHLQKLVANGLTHFHMLP

ANDIAT--IEEDADKLIDLDSTVLALCKLNFKAPPC----------------YQGIDDQT

LRSVFESYI---PFSNEAALLTEAMNDHDSFNWGYDPKHFNVPDGSYASNP---DGVSRI

KEMRAMIKSLHDIGLRVVLDVVYNHTNSSGL-WDNSVLDKFVPGYYHSRDVTTGAVLNGT

C--CSDTALEHRMMDKLMVDSLKQWTQQYQFDGFRFDIMSQGSKAQMLAARDAIQAI---

-----DADNHFYGEGW-----YKDARGFE----RADQENMA-GTEIATYNDRLRDGIRNA

TLFNNK-------------------------------------SDSDYPFEQQDIVKFGM

AGTLTDYVLKT-F-KGT-DVLGS---------ASGMYAKDPADIINYVSKHDNETLWDNL

QFNL--------S---------------PEMDNSERVRAQNISQAIVLLSQGIPFLQMGG

DFLRSKSLDRNTYDAGDWYNLVDFTF---------E-LNNWN-KGLPLDKGG----RTEA

ELVN-LAS-S-SS-SS--VALTDILFASNVFNEFLSIRSNSPLFRLTTATDIINRVGFHN

V--G-KN--QTQGLIVMSIDDGTN-----LT-DIDPNYDAVVVVVNGSDSEHTHTITSAS

----------GFTLHPLLAA--S-V--DGT-VASASFEQGTGEGSFTVPALTTAVFVK--

-------

>UniRef90_A0A4Q5QEK7_366_910 | Pullulanase-type alpha-1,6-glucosidase n=1 Tax=Gammaproteobacteria bacterium TaxID=1913989 RepID | E_val=2.7e-140

------------------------------------------------------------

--------------------Q---------------------------------------

------------------------------------------------------------

------------------------------------------------------------

------------------------------------------------------------

------------------------------------------------------------

-----------------SLRS---------------------------------------

------------------------------------------------------------

------------------------------------------------------------

---LLESLN---SRSGDAQAIIESLREQDNYNWGYDPFHYTVPQSSYAINP---EGSARI

IEFREMIAQLHNMGFKVIMDVVYNHTFAAGL-NEKSVLDKIVPNYYHRLHPVTGAIEQST

C--CNNTATEHVMMAKLMTDSLVVWARDYRINGFRFDLMGHQPKAVMLKARDAVRKI---

-----DPDTYFYGEGWNFG-EVENNKRFV----QSTQLELG-GTGIGTYTDRLRDAVRGG

SSMDSGKN-LRINQGIGNGLFVVPNESQ-----------SAD-NNKAKYLNYIDQIQIGL

AGNLAAFPLKN-A-VGK-EVVGSGV-DY-NG-SPAGYAKDPADTINYVSKHDNQTLWDNN

QYRI--------A---------------YSVSSADRVRMQALSLSYPLLAQGIPFLHMGC

EFLRSKSFLRDSYDYGDWFNAVDFSK---------Q-SNNYD-VGLPPAVKD----KDNW

PLIADVLK-K-SE-GRDHTTPAQIALSNNLFLDFIKIRSSSPLFRLRTADDIIKRVSFPN

S--G-EK--HQPGLVVMKIDNTDV-----NN-NLDPSTQQIIVVFNNGAVKQTFTYAD--

--------AKQFRLHPVQAK--G-S--DSL-VKTSS--ANT--KGFTVPAFTTAVFV---

-------
